# Development of genome-wide informative simple sequence repeat markers for large-scale genotyping applications in chickpea and development of web resource

**DOI:** 10.3389/fpls.2015.00645

**Published:** 2015-08-21

**Authors:** Swarup K. Parida, Mohit Verma, Santosh K. Yadav, Supriya Ambawat, Shouvik Das, Rohini Garg, Mukesh Jain

**Affiliations:** Functional and Applied Genomics Laboratory, National Institute of Plant Genome ResearchNew Delhi, India

**Keywords:** chickpea, genotyping, molecular diversity, polymorphism, population structure, simple sequence repeat

## Abstract

Development of informative polymorphic simple sequence repeat (SSR) markers at a genome-wide scale is essential for efficient large-scale genotyping applications. We identified genome-wide 1835 SSRs showing polymorphism between *desi* and *kabuli* chickpea. A total of 1470 polymorphic SSR markers from diverse coding and non-coding regions of the chickpea genome were developed. These physically mapped SSR markers exhibited robust amplification efficiency (73.9%) and high intra- and inter-specific polymorphic potential (63.5%), thereby suggesting their immense use in various genomics-assisted breeding applications. The SSR markers particularly derived from intergenic and intronic sequences revealed high polymorphic potential. Using the mapped SSR markers, a wider functional molecular diversity (16–94%, mean: 68%), and parentage- and cultivar-specific admixed domestication pattern and phylogenetic relationships in a structured population of *desi* and *kabuli* chickpea genotypes was evident. The intra-specific polymorphism (47.6%) and functional molecular diversity (65%) potential of polymorphic SSR markers developed in our study is much higher than that of previous documentations. Finally, we have developed a user-friendly web resource, Chickpea Microsatellite Database (CMsDB; http://www.nipgr.res.in/CMsDB.html), which provides public access to the data and results reported in this study. The developed informative SSR markers can serve as a resource for various genotyping applications, including genetic enhancement studies in chickpea.

## Introduction

In recent years, a significant progress has been made concerning the development of numerous genomic and transcript-derived simple sequence repeat (SSR) and single nucleotide polymorphism (SNP) markers at a genome-wide scale and their deployment in multi-dimensional genomics-assisted breeding applications in chickpea (Winter et al., 2000; Abbo et al., 2005; Millan et al., 2010; Nayak et al., 2010; Gujaria et al., 2011; Thudi et al., 2011; Gaur et al., 2012; Hiremath et al., 2012; Roorkiwal et al., 2013; Deokar et al., 2014; Jaganathan et al., 2014). This suggests that development, large-scale validation and use of functionally relevant informative sequence-based robust genetic markers revealing high intra-specific polymorphic potential are preferred in marker-assisted genetic enhancement studies of chickpea. Due to many inherent desirable genetic attributes, including abundance, co-dominant inheritance, reproducibility, multi-allelic nature and simpler genotyping potential (gel-based assay), SSRs have been considered as marker of choice in crop plants, including chickpea (Varshney et al., 2005, 2014; Parida et al., 2006; Sethy et al., 2006; Radhika et al., 2007; Upadhyaya et al., 2008; Nayak et al., 2010; Anuradha et al., 2011; Bharadwaj et al., 2011; Gujaria et al., 2011; Vadez et al., 2012).

Length polymorphism of SSR markers in the coding sequence (CDS) and non-CDS components [5′-untranslated regions (5′-UTRs), introns and 3′-untranslated regions (3′-UTRs)] of genes are known to affect transcription and translation, and may have significant consequences on gene function (Li et al., 2004). The expansion and contraction of SSR repeats in the 5′-UTRs, for instance, have significance in regulating many traits (Dresselhaus et al., 1999; Bao et al., 2002; Zhang et al., 2006). The length polymorphism in the functional domain of transcription factor genes, and alteration of secondary structure of proteins and functional domain sites have been proposed to control seed weight/seed size in chickpea (Kujur et al., 2013). These studies have suggested the utility of coding and non-CDS-based SSR markers for rapidly establishing marker-trait linkages and identifying genes/QTLs for many useful agronomic traits in crop plants. The SSR markers particularly derived from the non-CDS components of genes with moderate selection pressure are expected to reveal high intra-specific polymorphism in contrast to highly constrained CDS-based markers (Cho et al., 2000; Chabane et al., 2005; Parida et al., 2010; Kujur et al., 2013).

The utility of genome-wide identification of polymorphic SSR markers by comparing the genomic or transcript sequences between *indica* and *japonica* rice (Grover et al., 2007; Zhang et al., 2007), and *Setaria italica* and *S. viridis* (Zhang et al., 2014) as well as among chickpea genotypes (Agarwal et al., 2012; Jhanwar et al., 2012) in large-scale genotyping applications has been well demonstrated. A large chickpea genome (∼740 Mb) with narrow genetic base requires a huge number of such functionally relevant polymorphic SSR markers at a genome-wide scale for various applications in structural, functional, and applied genomics. With the availability of draft genome sequences of *desi* and *kabuli* chickpea (Jain et al., 2013; Varshney et al., 2013), it is now possible to mine informative SSR markers from coding and non-CDS components of genes in the two chickpea genomes. Recently, SSR markers identified from the *kabuli* genome have been made publicly accessible via a web resource, *Cicer arietinum* Microsatellite Database (CicArMiSatDB; Doddamani et al., 2014). The informativeness of such genome-wide SSR markers can further be enriched by identifying a subset of markers showing polymorphism between *desi* and *kabuli* chickpea in different sequence components of annotated genes.

Keeping all above in view, the present study was undertaken to mine and characterize SSR repeat-motifs in different coding and non-CDS components, and intergenic regions between *desi* and *kabuli* chickpea genomes and develop genome-wide polymorphic SSR markers. The developed markers were evaluated to determine their amplification and polymorphic potential, and assessment of functional molecular diversity and population genetic structure among *desi* and *kabuli* chickpea genotypes. In addition, we have developed an easy-to-use web resource for public access of the SSR data generated in this study. The development of informative markers would further expedite the process of construction of high resolution genetic map, and identification and mapping of genes/QTLs regulating important agronomic traits for genetic improvement of chickpea.

## Materials and Methods

### Discovery of SSRs in Chickpea Genomes

The *desi* (*Cicer arietinum* L. cv. ICC4958; Jain et al., 2013) and *kabuli* (*C. arietinum* L. cv. CDC Frontier; Varshney et al., 2013) chickpea genomes were obtained from Chickpea Genome Analysis Project^1^ and International Chickpea Genetics & Genomics Consortium^2^, respectively. These sequences were mined for SSRs using MISA (MIcroSAtellite^3^) following the criteria (at least six repeats of di-nucleotides and five repeats of tri- to hexa-nucleotides) as described earlier (Garg et al., 2011; Jhanwar et al., 2012). The perfect SSRs were further classified into hypervariable class I (≥20 bp) and potentially variable class II (12–20 bp) types according to length of repeat-motifs. The structural annotation of identified SSRs in different coding (CDS) and non-coding (5′-UTRs, introns and 3′-UTRs) sequence components of chickpea genes, and upstream regions (1000 bp) and intergenic regions was performed based on the available genome annotation. The putative function of SSRs containing gene sequences was determined based on their available functional annotation information.

### Development of Gene-Derived Polymorphic SSR Markers in Chickpea

The SSRs showing polymorphism between *desi* and *kabuli* chickpea were identified using the approach described by Zhang et al. (2007). In the first step, 250 bp flanking sequences on each side of the identified SSR motifs were retrieved from both the genomes and searched against each other via BLASTN. Only the sequences showing unique hit in the reciprocal BLAST results with an *E*-value cut-off of ≤1e-40 were retained. This resulted in the identification of 13327 orhtologous SSR loci in the two genomes. Further, we used custom designed perl script to identify and characterize the polymorphic SSR loci between *desi* and *kabuli* chickpea based on difference in number of repeat-units present, as described by Jhanwar et al. (2012). The forward and reverse primers from the genomic sequences of ICC 4958 flanking polymorphic SSR repeat-motifs were designed using the Primer3 tool at default parameters. The polymorphic SSR markers were physically mapped on eight chromosome pseudomolecules of chickpea according to their genomic location.

### Evaluation of Amplification Efficiency and Polymorphic Potential

A total of 341 selected polymorphic SSRs (showing ≥4-bp fragment length polymorphism between *desi* and *kabuli* chickpea) present in the coding and non-CDS components of genes, and intergenic regions were amplified via PCR using genomic DNA of 31 *desi* and 15 *kabuli* chickpea genotypes to evaluate their amplification and polymorphic potential. These *desi* and *kabuli* genotypes [collected from Indian Agricultural Research Institute (IARI), New Delhi and International Crops Research Institute for the Semi-Arid Tropics (ICRISAT), Hyderabad] with diverse useful yield contributing and stress tolerance traits, have been primarily utilized as contrasting parents in various cross-breeding varietal improvement programs for developing improved cultivars of chickpea in India. The touchdown thermal cycling profiling and standard constituents used for PCR amplification were as described previously (Agarwal et al., 2012; Jhanwar et al., 2012). The PCR products amplified by each SSR marker in the 46 chickpea genotypes were resolved on 3.5% metaphor agarose gel and their allele size and fragment length polymorphism was determined. The genotyping information of SSR markers was used to estimate the number of polymorphic alleles per marker locus, percent polymorphism and polymorphism information content (PIC) among genotypes using PowerMarker v3.51 (Liu and Muse, 2005). The polymorphic SSRs were positioned on the chickpea chromosomes according to their genomic coordinates using MapChart (v2.2).

### Functional Molecular Diversity and Population Structure Analysis

A total of 160 informative SSR markers were used for determining functional molecular diversity and establishing phylogenetic relationships among chickpea genotypes based on Nei’s genetic distance (Nei et al., 1983) by neighbor-joining (NJ) method (with 1,000 bootstrap replicates) using PowerMarker v3.51 and unrooted phylogenetic tree was constructed. For assessment of population structure, the SSR marker genotyping data were analyzed in STRUCTURE (Pritchard et al., 2000) with burn-in of 100000 iterations and run length of 1000000, following the method described by Kujur et al. (2013). The genetic variability (*F*_ST_) and degree of admixture within and between population groups at optimal *K* (population number) value was determined.

### Construction of Chickpea Microsatellite Database

We developed a user-friendly web resource, Chickpea Microsatellite Database (CMsDB), to provide browsable access to the SSR data. The web pages of CMsDB have been written using Perl-CGI on the Apache Tomcat (version 5.5.29) Web server application. The information regarding SSRs, their flanking sequences and primer details are cataloged in the MySql server (version 5.0.77). The database is currently hosted on Sun Workstation running CentOs (version 5.4) Linux operating system with two Intel Xeon quad core processors and 12 GB of random access memory. The database is compatible with various browsers like Internet Explorer, Mozilla Firefox, and Google Chrome.

## Results

### Frequency and Distribution of SSRs in *desi* and *kabuli* Chickpea Genomes

**FIGURE 1:**
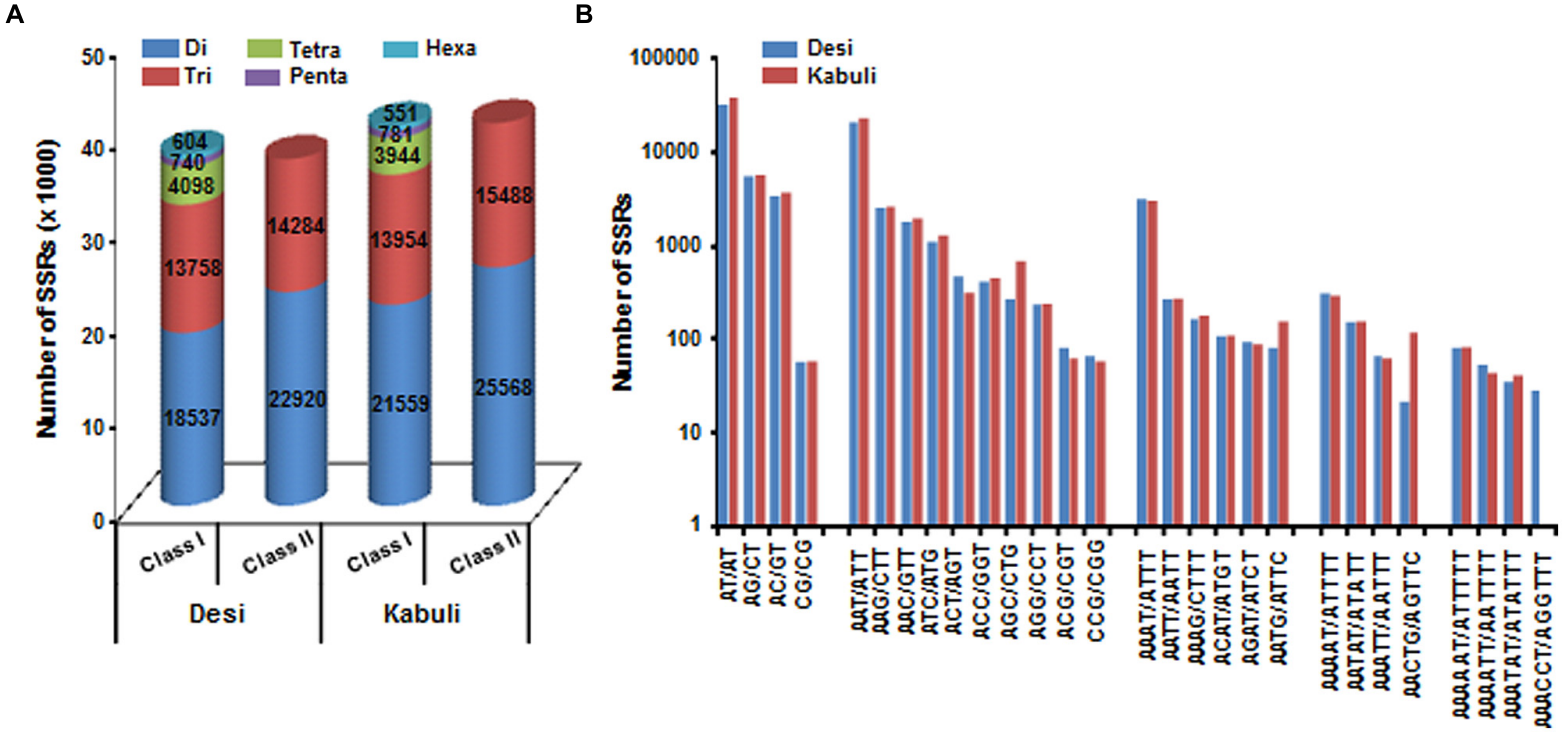
**Classification and frequency of simple sequence repeats (SSRs) identified in *desi* and *kabuli* chickpea genomes. (A)** The bar graph displays the number of SSRs of different types in long hypervariable class I (≥20 nt) and potentially variable class II (12–20 nt). **(B)** Frequency of major SSR motifs of different classes identified in *desi* and *kabuli* genomes.

**FIGURE 2:**
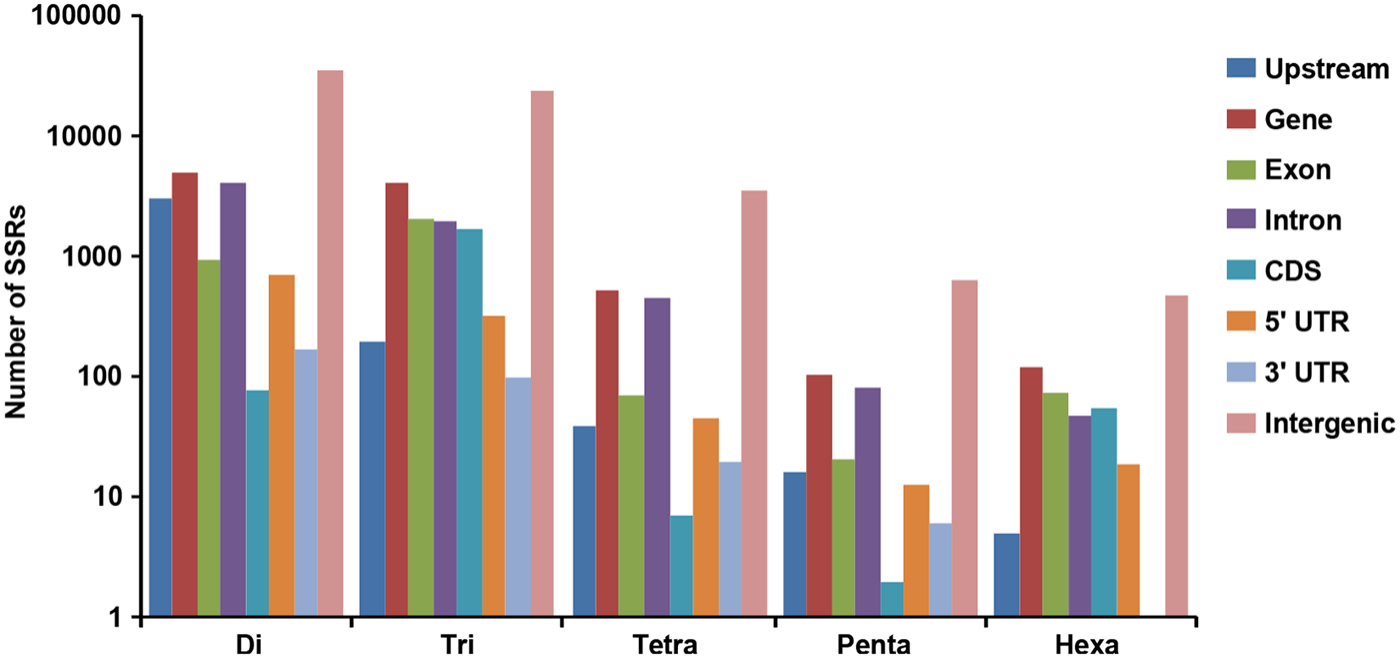
**Distribution of SSRs identified from *desi* and *kabuli* chickpea genomes in different regions of the chickpea genome.** The bar graph displays the number of SSRs of different classes located in different genomic features (various gene components and intergenic regions) of the chickpea genome.

A total of 519.8 and 522.3 Mb sequences of *desi* and *kabuli* chickpea genomes, respectively, were utilized for mining and characterization of SSR motifs. Based on these analyses, 74941 and 81845 perfect SSRs (excluding mono-nucleotides) were identified in *desi* and *kabuli* chickpea with an average density of 0.144 SSR/kb and 0.157 SSR/kb, respectively (**Table 1**). The overall frequency of compound SSRs identified in *desi* (2563, 0.005 SSRs/kb) and *kabuli* (2470, 0.004 SSRs/kb) chickpea was almost comparable with each other. In both the genomes, di-nucleotide repeat-motifs (*desi*: 41457, 55.3% and *kabuli*: 47127, 57.6%) were most prevalent followed by tri- and tetra-nucleotides (**Table 1**). In terms of proportion of total number of SSRs identified, the long hyper-variable class I repeats varied from 49.8% (40789) in *kabuli* to 50.3% (37737) in *desi* chickpea. The class I and class II di-nucleotide repeat-motifs were present in maximum fraction varying from 49.1 (18537) to 62.3% (25568) in *desi* and *kabuli* chickpea (**Figure 1A**). Next, the tri-nucleotide SSR repeat-motifs were most abundant (varied from 34.2 to 38.4%) in both class I and class II SSRs, while tetra-, penta-, and hexa-nucleotide motifs were completely absent in case of class II SSRs. The frequency of AT-rich di-nucleotide repeat-motifs (32354 SSRs in *desi*, 43.2% and 37977 SSRs in *kabuli*, 46.4%) was maximum in both *desi* and *kabuli* chickpea followed by AAT/ATT-rich tri-nucleotide SSRs (21113 SSRs, 28.2% in *desi* and 22003, 26.9% in *kabuli*) and AAAT/ATTT-rich tetra-nucleotide SSRs (3095, 4.1% and 2924, 3.6%) (**Figure 1B**). The structural annotation of identified SSRs in *desi* genome revealed their highest frequency in intergenic regions (64961 SSRs) followed by introns (6762), exons (3218), CDS (1816), and upstream regions (3289) (**Figure 2**). All five different classes of SSR repeat-motifs (di- to hexa-nucleotides) were predominant particularly in the intergenic regions as compared to various coding and non-CDS components of genes. However, within genes, the frequency of di- (4191), tetra- (462), and penta-nucleotide (84) SSR repeat-motifs were maximum in the intronic and upstream sequences, whereas tri- (2100) and hexa-nucleotide (76) motifs were abundant in the exons (**Figure 2**). Maximum number of tri-nucleotide SSR repeat-motifs was found in the CDS (1673 SSRs), whereas upstream regions (3029), and 5’- (703) and 3’-UTRs (168) were rich in di-nucleotide repeat-motifs.

**Table 1:** Summary of SSRs identified in *desi* and *kabuli* chickpea genomes.

Characteristic	*Desi*	*Kabuli*
Total number of sequences examined	181462	7135
Total size (bp) of examined sequences	519846222	522314610
Total number of identified SSRs	74941	81845
Number of SSRs containing sequences	18559	1769
Number of SSRs present in compound formation	2563	2470
Frequency of SSRs	0.144 SSR/kb	0.157 SSR/kb
Number (%) of di-nucleotides	41457 (55.3)	47127 (57.6)
Number (%) of tri-nucleotides	28042 (37.4)	29442 (36.0)
Number (%) of tetra-nucleotides	4098 (5.5)	3944 (4.8)
Number (%) of penta-nucleotides	740 (0.98)	781 (0.95)
Number (%) of hexa-nucleotides	604 (0.81)	551 (0.67)
Number (%) of class I SSRs	37737 (50.4)	40789 (49.8)
Number (%) of class II SSRs	37204 (49.6)	41056 (50.2)

### Density and Relative Abundance of Polymorphic SSRs

**FIGURE 3:**
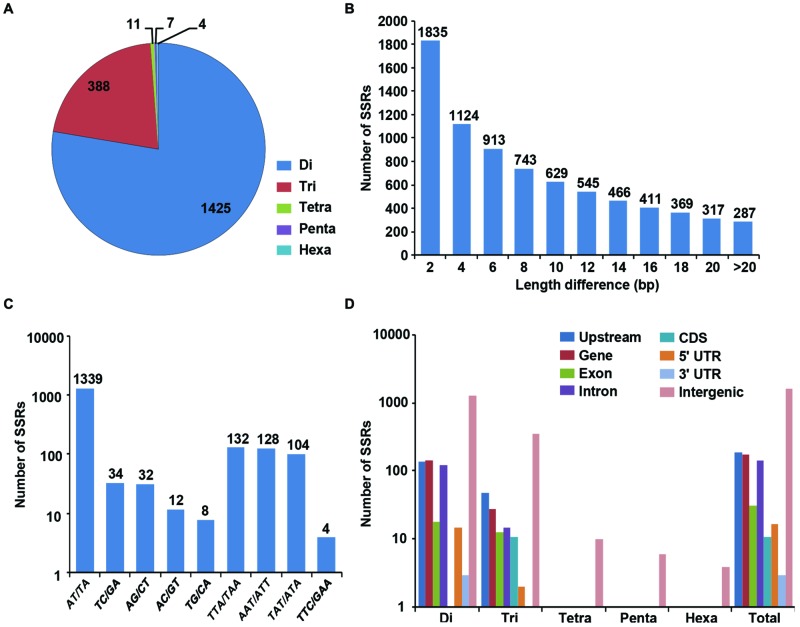
**Identification and analysis of polymorphic SSRs between *desi* and *kabuli* chickpea. (A)** Distribution of polymorphic SSRs in different classes. **(B)** Number of SSRs with different motif length (2 to >20) differences. **(C)** Frequency of selected (most abundant) motifs in the polymorphic SSRs. **(D)** Distribution of polymorphic SSRs in in different genomic features (various gene components and intergenic regions).

We identified 1835 genome-wide SSRs showing polymorphism between *desi* and *kabuli* chickpea based on their expansion/contraction of SSR repeat-length (ranged from 2 to >20 bp; **Figures 3A,B**). Among the polymorphic SSRs obtained, the di-nucleotide repeat-motifs (1425 SSRs, 77.6%) were most abundant followed by tri-nucleotide motifs (388, 21.1%) (**Figure 3B**). With the increase of SSR repeat-length variation, the number of SSRs showing polymorphism decreased, which indicates inverse correlation between fragment length polymorphism and frequency of polymorphic SSRs identified in *desi* and *kabuli* chickpea (**Figure 3A**). Maximum number of polymorphic SSRs showed ≥2-bp repeat-unit variation and 287 SSRs with >20-bp variation between *desi* and *kabuli* chickpea were identified. The AT/TA di-nucleotide repeats (1339, 73%) were maximum followed by TTA/TAA (132, 7.2%), AAT/ATT (128, 6.8%), and TAT/ATA (104, 5.7%) (**Figure 3C**). All the identified 1835 polymorphic SSRs were structurally annotated in intergenic regions, and coding and non-CDS components of genes. Maximum number of polymorphic SSR repeats were identified in the intergenic regions (1453 SSRs, 79.2%), (**Figure 3D**). The polymorphic SSRs identified within genes included highest number in the upstream sequences (190, 10.4%) followed by introns (143, 7.8%), exons (31, 1.7%) and minimum in 3′-UTRs (3, 0.16%) of genes. All classes of polymorphic SSR repeats including di- to hexa-nucleotide motifs were maximum in the intergenic sequences (**Figure 3D**). The di-nucleotide followed by tri-nucleotide SSR repeats were abundant in the upstream regions (139 di- and 49 tri-nucleotide) and intronic (126 di- and 15 tri-nucleotide) sequences of genes, whereas tri-nucleotide repeats were maximum in the coding regions. About 85% of the polymorphic SSRs were present on the eight chickpea chromosomes, whereas other 15% were located on the scaffolds of *kabuli* chickpea genome (Supplementary Figure S1). A complete list of polymorphic SSRs along with their motifs and genomic location in both genomes is given in the Supplementary Table S1.

The higher abundance of polymorphic SSRs specifically in the upstream regulatory regions and introns of chickpea genes is consistent with the previous reports in rice and *Arabidopsis* (Fujimori et al., 2003; Lawson and Zhang, 2006; Parida et al., 2009). The most frequent occurrences of di- and tri-nucleotide SSR repeats in the upstream regulatory regions of genes are expected. These SSR repeats can possibly facilitate or abolish binding sites of regulatory proteins and thus regulate gene expression (Yu et al., 2002; Lawson and Zhang, 2006; Parida et al., 2009). The presence of AT-rich di-nucleotide SSRs in the introns of chickpea genes is also comparable to that observed in the earlier studies of cereal genomes (Temnykh et al., 2001; Lawson and Zhang, 2006; Parida et al., 2009). The genome-wide identification and characterization of SSRs including those showing polymorphism between *desi* and *kabuli* revealed non-random and biased distribution across various genomic components.

### Development and Annotation of Polymorphic SSR Markers

**FIGURE 4:**
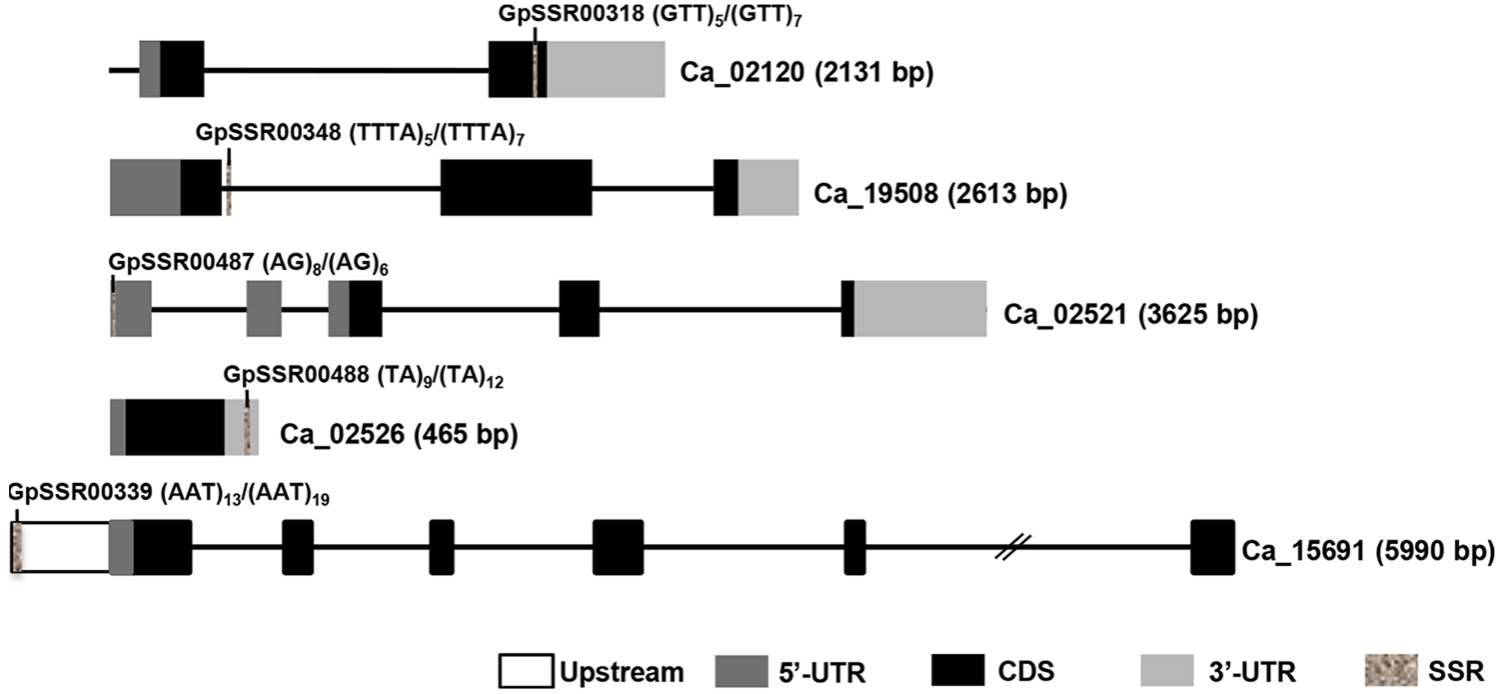
**Diagrammatic representation of polymorphic SSRs present in different components of chickpea genes.** Introns and exons are represented by lines and boxes, respectively. Different components of exons are represented in different shaded boxes. The SSR motifs present in different genic regions have been highlighted in gray texture. The motif and its frequency in both chickpea (*desi*/*kabuli*) types are also given. Gene ID and length (bp) are given on right side.

The forward and reverse primers from the flanking sequences of polymorphic 1835 SSR repeat-motifs were designed. The structural annotation and organization of selected SSR repeats located in different components of genes are presented in the **Figure 4**. We could design primers for 1470 (80.1%) polymorphic SSRs, which included 1151 (78.3%) in the intergenic regions, 150 (10.2%) in the upstream regulatory sequences, 125 (8.5%) in the introns, 31 (2.1%) in the exons, 17 (1.2%) in the 5′-UTRs, 11 (0.75%) in the CDS, and 3 (0.20%) in the 3′-UTRs (Supplementary Table S1). At least 85% of these genome-wide unique SSR markers were mapped on the chickpea chromosome pseudomolecules (Supplementary Figure S1). Maximum number of markers were mapped on chromosome 4 (224, 15.2%) followed by chromosome 5 (206, 14%), while minimum number of markers mapped on chromosome 8 (72, 4.9%).

### Amplification and Polymorphic Potential of Developed SSR Markers

**FIGURE 5:**
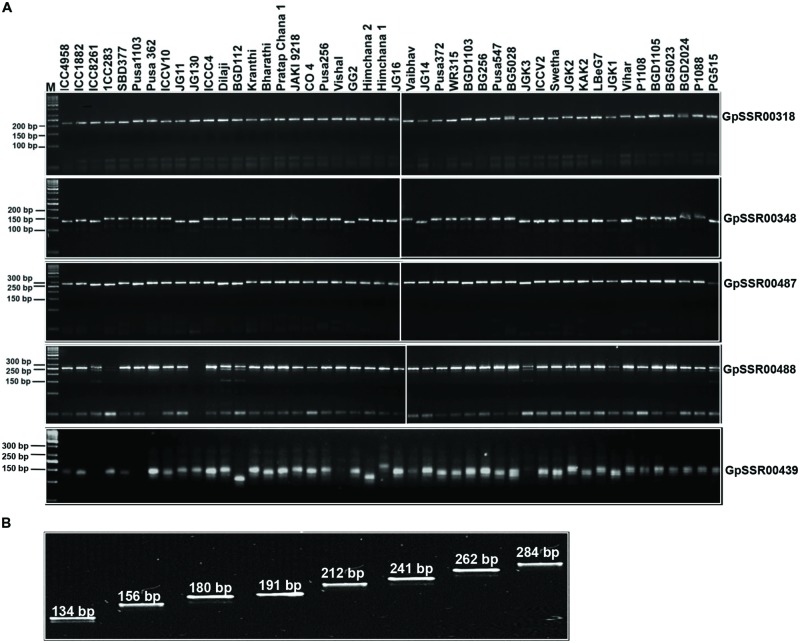
**Validation of amplification and polymorphic potential of selected SSRs and allelic variations in chickpea genotypes. (A)** Polymerase chain reaction based validation of amplification and polymorphic potential of selected SSRs in chickpea genotypes. Only five selected examples of SSRs have been represented. Representative gels showing PCR amplification of polymorphic SSRs (labeled on right side) validating the length polymorphism between *desi* and *kabuli* chickpea genotypes (genotype details are available in Supplementary Table S3). M, 50 bp DNA ladder as size standard. **(B)** Eight different representative allele types identified based on the fragment length polymorphism pattern of 160 SSR markers across *desi* and *kabuli* chickpea genotypes. The SSR marker-alleles are illustrated according to their lower to higher fragment size (bp).

We utilized 341 SSR markers (revealing ≥4-bp fragment length polymorphism between *desi* and *kabuli* chickpea) in total located in different components of genes (upstream, 5′-UTRs, CDS, introns and 3′-UTRs) and intergenic regions to evaluate their amplification efficiency as well as potential for detecting polymorphism among 31 *desi* and 15 *kabuli* chickpea genotypes (Supplementary Table S3). Two hundred fifty-two of the 341 markers gave successful PCR amplification in all 46 chickpea genotypes with an amplification success rate of 73.9% (Supplementary Table S2). One hundred sixty (63.5%) of 252 amplified markers showed polymorphism in at least two combinations of chickpea genotypes (**Figure 5A**). It included 130 (73.9%, mean PIC: 0.76) of 176 class I and 30 (39.5%, 0.69) of 76 class II SSR markers. The remaining 92 (36.5%) markers exhibited monomorphic amplification among chickpea genotypes used. A total number of 764 alleles were amplified by 160 polymorphic SSR markers with a mean allele number of 4.8. The number of alleles amplified per locus varied from 2 to 12. The PIC ranged from 0.23 to 0.86 with an average of 0.75, while gene diversity varied from 0.25 to 0.89 with a mean of 0.77 (Supplementary Table S2). The polymorphic potential of markers in different sequence components of genes and intergenic regions was analyzed in detail based on the percent polymorphism, PIC and polymorphic alleles amplified among chickpea genotypes. We were able to detect polymorphism in 55 (62.5%, allele number from 2–9 and PIC 0.74) of 88 markers derived from the different coding and non-CDS components of genes between *desi* and *kabuli* chickpea. The remaining 105 (64%, 2–12 and 0.63) of 164 markers derived from intergenic regions also showed polymorphism between the two chickpea types (Supplementary Table S2). Within genes, maximum potential of polymorphism was detected by the markers developed from intronic sequences (28 of 38 markers, 73.7%, allele number 2–9 and PIC 0.74) followed by 3′-UTRs (5 of 8 markers, 62.5%, 2–4 and 0.66), upstream (19 of 35 markers, 54.3%, 2–5 and 0.76) and 5′-UTRs (2 of 6 markers, 33.3%, 4–5 and 0.62) of genes (Supplementary Table S2, **Figure 5A**). Remarkably, 120 (47.6%, 2–10 and 0.62) and 107 (42.5%, 2–8 and 0.57) markers revealed polymorphism within *desi* and *kabuli* chickpea genotypes too, respectively. Overall, eight different representative allele types were detected based on fragment length polymorphism patterns of all the 160 SSR markers in 46 *desi* and *kabuli* genotypes (**Figure 5B**).

### Functional Molecular Diversity and Population Structure Among *desi* and *kabuli* Chickpea

**FIGURE 6:**
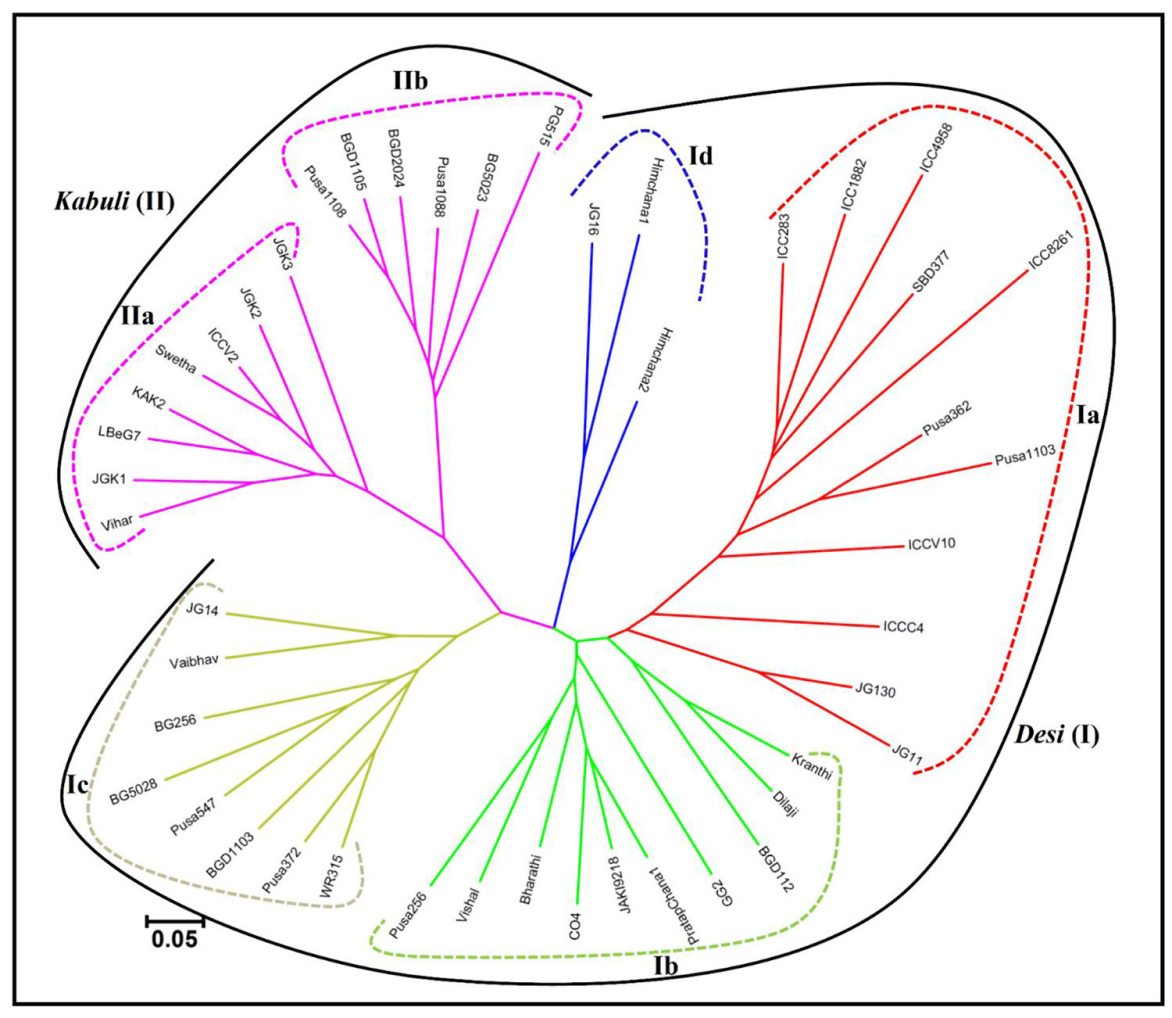
**Unrooted phylogenetic tree depicting the genetic relationships among *desi* and *kabuli* genotypes.** The tree was generated based on Nei’s genetic distance using genotyping data of 160 informative SSR markers in 31 *desi* and 15 *kabuli* chickpea genotypes. Molecular classification clearly differentiated genotypes into six different clusters, which corresponded to their cultivar-specific origin and parentage/known pedigree relationships.

The pair-wise distance matrix among 46 *desi* and *kabuli* chickpea genotypes based on genotyping information of 160 validated polymorphic SSR markers revealed a broad range of genetic distance that varied from 0.16 (*kabuli* cv. BGD1105 – *kabuli* cv. Pusa1088) to 0.94 (*desi* cv. Vishal – *desi* cv. ICC4958) with an average of 0.68. Maximum average genetic distance was observed particularly among the accessions belonging to *desi* chickpea (0.65) in contrast to that detected within *kabuli* (0.57). The phylogenetic relationship among 31 *desi* and 15 *kabuli* chickpea genotypes has been depicted in an unrooted dendrogram (**Figure 6**). This set of informative genome-wide physically mapped SSR markers (160) clearly discriminated all 46 genotypes from each other and resulted in definite *desi* and *kabuli* cultivar-specific groupings. Most of the *desi* and *kabuli* genotypes were grouped in separate clusters (I and II), which further corresponded well with their known pedigree relationships and parentage with slight deviations. However, *desi* genotypes included under cluster I were further grouped into four different sub-clusters (Ia, Ib, Ic, and Id), while *kabuli* genotypes belonging to cluster II classified into two different sub-clusters (IIa and IIb; **Figure 6**).

**FIGURE 7:**
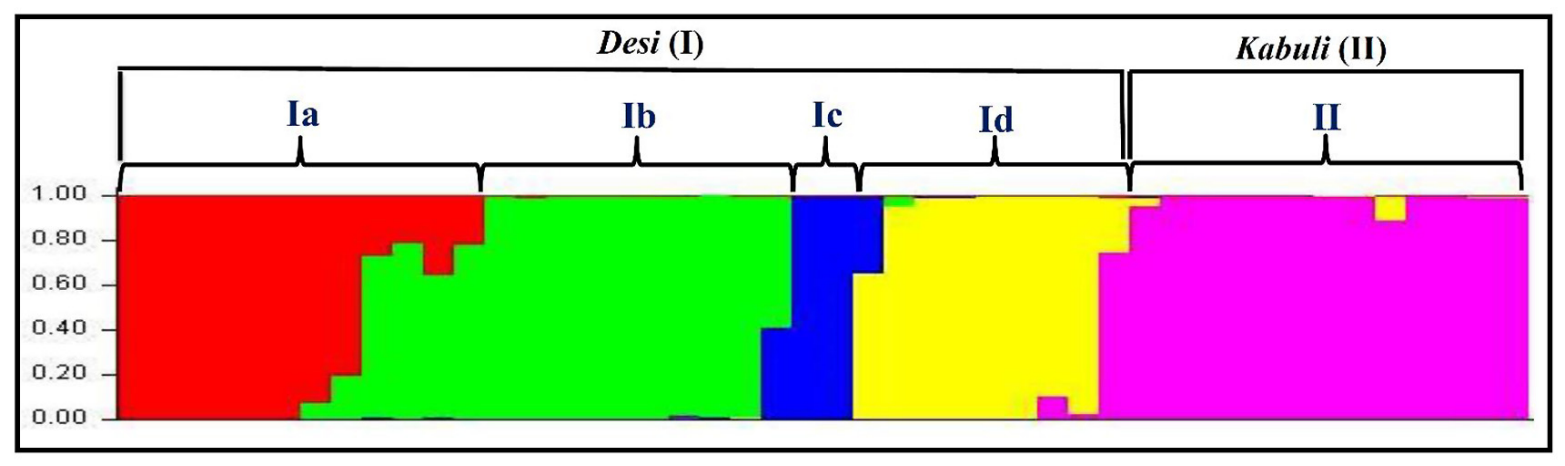
**Population genetic structure inferred best possible structure among *desi* and *kabuli* chickpea genotypes.** The genotyping data of 160 informative genome-wide SSR markers in 31 *desi* and 15 *kabuli* chickpea genotypes was used for this analysis. These mapped markers assigned 46 chickpea genotypes into five populations that majorly grouped accordingly by their cultivar-specific origin and parentage/pedigree relationships. The accessions represented by vertical bars along the horizontal axis were classified into *K* color segments based on their estimated membership fraction in each *K* cluster. Five diverse colors represent different population groups based on optimal population number *K* = 5.

The population genetic structure among 31 *desi* and 15 *kabuli* chickpea genotypes was determined using 160 validated polymorphic SSR markers with varying levels of population numbers (*K* = 2–10) with 20 replications. The optimization of *K* inferred that at *K* = 5, the average estimate of Ln P(D) across 20 independent replications plateaus and also best replicate giving maximum log likelihood values with sharp peak was obtained. All 46 chickpea genotypes were majorly classified into two distinct high resolution population groups (**Figure 7**). The population groups, I (31 *desi* and one *kabuli* chickpea) and II (14 *kabuli* chickpea) contained the genotypes mostly from *desi* and *kabuli* chickpea, respectively. The *desi* population group (I) was further classified into four sub-population groups; Ia (10 *desi* and one *kabuli* chickpea genotypes), Ib (10 *desi*), Ic (8 *desi*), and Id (3 *desi*) (**Figure 7**). The cultivar-specific classification and geographical origin of 46 chickpea genotypes belonging to all the five individual population groups are provided in the Supplementary Table S3. The population groupings obtained among 46 chickpea genotypes corresponded well with their origin and pedigree relationships/parentage. This was further consistent with the clustering patterns and genetic relationships as obtained by the NJ tree analysis. Further, molecular genetic variation among and within five populations was estimated using above 160 informative SSR markers. It revealed a wider level of quantitative genetic differentiation (*F*_ST_ varied from 0.16–0.91 with an average of 0.64) among five population groups. The genetic variation among the five population groups (mean *F*_ST_: 0.62) was higher than that estimated within populations (0.53). Higher molecular diversity of population group I (mean *F*_ST_: 0.86) as compared to group II (0.69) was evident. Within population groups I and II, maximum divergence was observed in population groups Ib (mean *F*_ST_: 0.83) and IIb (0.65), respectively. All the 46 chickpea genotypes clearly belonged to a structured population of five distinct groups in which about 74.2% of inferred ancestry of each group was derived from one of the model-based population and remaining ∼25.8% contained admixed ancestry. Maximum admixtures (20.3%) of the three *desi* population groups (Ib, Ic, and Id) with *kabuli* population (II) were observed.

### CMsDB: Features and Utility

**FIGURE 8:**
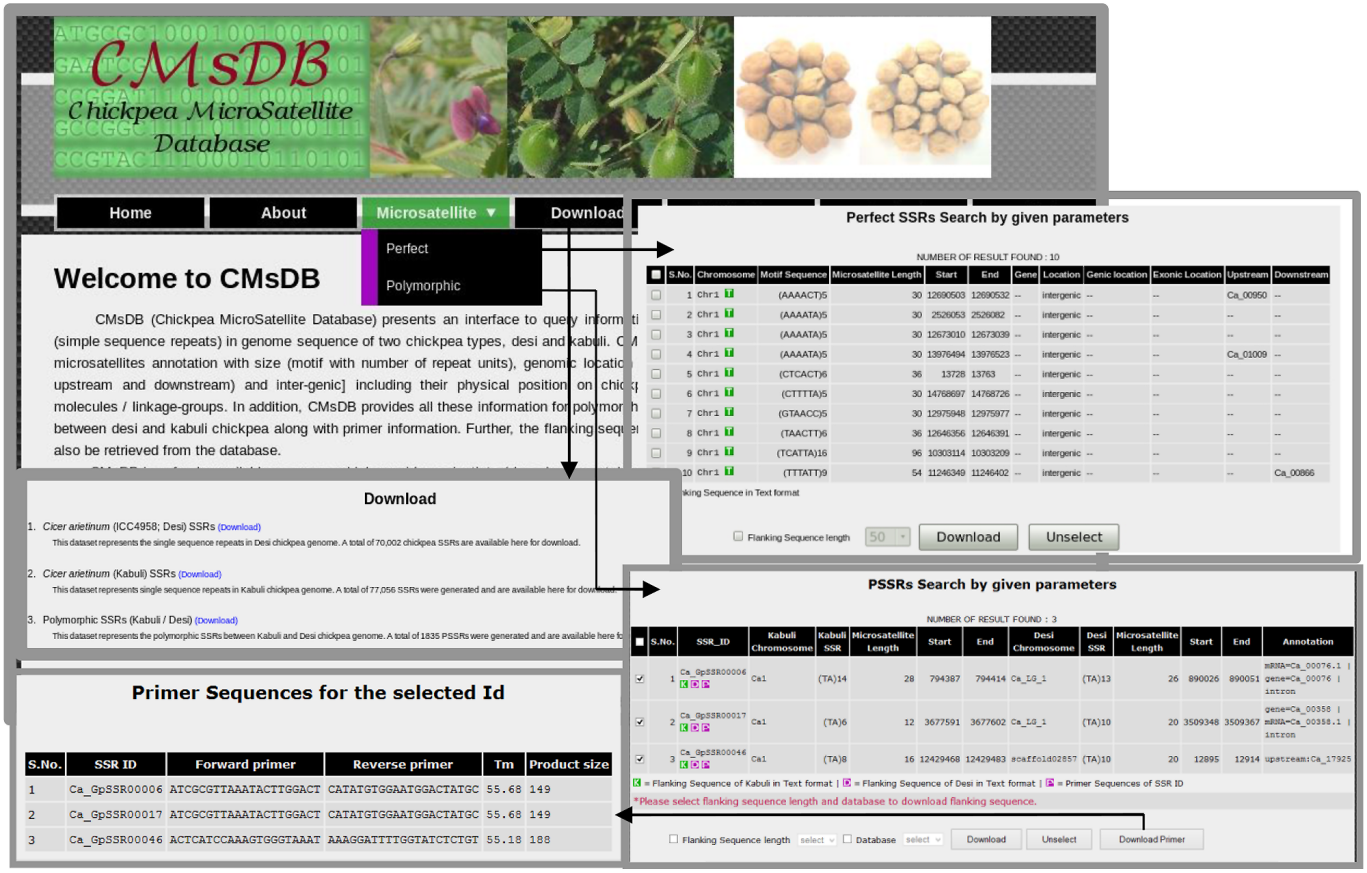
**Snapshots of the public web resource CMsDB showing its various utilities.** The snapshots were taken from the database webpages.

We developed a public data resource, CMsDB, to provide a searchable interface to the SSR data reported in this study. CMsDB is publicly available at http://www.nipgr.res.in/CMsDB.html. The database provides browsable access to all the SSRs identified in *desi* and *kabuli* chickpea types, and polymorphic SSRs between them. CMsDB can be used to retrieve SSRs in *desi* and *kabuli* genomes using various simple [genomic location (chromosome number and position) and genomic feature (genic and/or inter-genic)] and advanced [motif type (di- to hexa-nucleotide), motif sequence, repeat number and repeat unit length] search parameters. Multiple parameters can be combined also to search for a specific set of SSRs as per user requirement. The output lists all the SSRs meeting the user-selected parameters(s) in tabulated format along with various information, including SSR identifier, chromosome number, motif type and length, genomic location (start and end position in bp) and location in the genomic features (genic/intergenic, gene identifier and intron/exon/upstream sequence). An option for downloading the flanking sequences (50–250 bp) of individual/multiple SSRs has also been provided. Polymorphic SSRs between *desi*/*kabuli* can also be searched/retrieved using similar parameters. Further, CMsDB provides information on the primers designed for the polymorphic SSRs. In addition to download the flanking sequences (50–250 bp), an option for viewing/downloading the designed primers for individual/multiple polymorphic SSRs has also been provided. Whole datasets have also been made available for download for high-throughput genotyping applications. We aim to update the database as the new versions of *desi* and *kabuli* reference genome sequence data set(s) will become available for chickpea. **Figure 8** provides snapshots of various features and utilities of the CMsDB.

## Discussion

The development and large-scale validation of informative genome-wide SSR markers showing high intra-specific polymorphic potential among *desi* and *kabuli* genotypes is required for marker-assisted genetic improvement of chickpea. In this context, we discovered 74941 and 81845 SSRs from the *desi* and *kabuli* chickpea genomes, respectively, and inferred their frequency and genomic distribution in the intergenic regions and within protein-coding genes. Subsequently, 1835 polymorphic (based on SSR repeat-length variation) SSRs between *desi* and *kabuli* genomes from different coding and non-CDS components were identified and characterized. CMsDB provides an integrated web interface to search, browse and filter the SSRs in *desi* and *kabuli* chickpea genomes and polymorphic SSRs between them. Recently, the CicArMiSatDB comprising SSR markers identified from coding and non-coding regions of *kabuli* genome (Varshney et al., 2013) has been constructed (Doddamani et al., 2014). CMsDB developed in our study provides additional interfaces for genome-wide SSR markers from both *desi* and *kabuli* chickpea, and polymorphic SSR markers (derived from different coding and non-CDS components) between them, Thus, CMsDB will assist molecular breeders in rapid selection of gene-based polymorphic SSR markers for use in large-scale genotyping applications of chickpea.

Large-scale genome-wide SSR markers (designed from diverse coding, non-coding and intergenic regions) showing polymorphism between *desi* and *kabuli* chickpea developed in our study will serve as an immediate resource for mapping whole genome and targeted mapping of trait-specific genes/QTLs for marker-assisted genetic improvement in chickpea. These polymorphic SSR markers being derived from different coding and non-CDS components that regulate cellular and biological functions have significance in developing functional genetic markers for rapidly establishing marker-trait linkages and identification of genes/QTLs associated with important agronomic traits. The association of gene-based SSR markers based on their expansion/contraction of repeats with many traits of agricultural importance including seed weight in chickpea have been well studied (Kujur et al., 2013, 2014).

Higher (∼74%) amplification success rate of polymorphic SSR markers in chickpea genotypes suggested their immense use in various genotyping applications in chickpea. The remaining ∼26% of SSR markers derived mostly from the introns and intergenic regions of genes showed null amplification in chickpea genotypes. It could be due to insertion–deletions in the primer-binding sites of corresponding genomic sequences of *desi* and *kabuli* chickpea. This may also result from frequent association of transposable elements with the intronic and intergenic SSRs as previously documented in rice (Temnykh et al., 2001; Parida et al., 2009). The extent of intra-specific polymorphic potential (63.5%, 2–12 alleles and PIC: 0.75) detected in our study is much higher than that obtained previously using random SSR markers (∼35–40%; Sethy et al., 2006; Nayak et al., 2010; Bharadwaj et al., 2011; Gujaria et al., 2011; Hiremath et al., 2011; Kujur et al., 2013) and polymorphic SSR markers (50–60%, Hiremath et al., 2011; Agarwal et al., 2012). The maximum polymorphic potential (average 63% and mean PIC: 0.69) of SSR markers specifically derived from the intergenic, upstream regulatory regions and intronic sequences in chickpea agreed well with the previous reports in crop plants (Grover et al., 2007; Zhang et al., 2007; Parida et al., 2009, 2010). It suggested the utility of polymorphic SSRs derived from non-CDS component of genes in chickpea. The presence of abundant di- and tetra-nucleotide SSR repeats with their specific characteristics of showing high replication slippage than tri-nucleotide SSRs (more constrained by selective pressure), particularly in the upstream regulatory regions, introns and intergenic regions might be contributing to their high polymorphic potential. Among the identified polymorphic genome-wide SSR markers, the class I and non-coding gene sequence-derived SSR markers were found more informative and thus would have greater utility in rapid selection of polymorphic markers for efficient genotyping applications in chickpea. The SSR marker-based polymorphic potential (64.7%, PIC: 0.75) among *desi* and *kabuli* chickpea genotypes was higher (47.6%, PIC: 0.62) than that within *desi* or *kabuli* genotypes. Henceforth, the developed genome-wide SSR markers being more informative (in terms of high intra-specific polymorphic potential as well as functional significance) than SSR markers identified till now would be of immediate use in efficient large-scale genotyping applications in chickpea. Considering requirement of functional SSR markers showing high intra-specific polymorphism among *desi* and *kabuli* genotypes for chickpea marker-assisted genetic enhancement, the large-scale experimentally validated polymorphic SSR markers developed by us will be highly relevant.

A wider level of genetic differentiation (*F*_ST_ varied from 0.16 to 0.94 with a mean of 0.68) obtained among 46 chickpea genotypes belonging to five population groups was comparable/higher than the previously detected level (0.03–0.82) with the genomic and genic SSR markers (Sethy et al., 2006; Choudhary et al., 2009; Bharadwaj et al., 2011; Kujur et al., 2013). The higher molecular diversity between *desi* and *kabuli* population groups than that obtained within *desi* and *kabuli* populations is expected in a self-pollinated crop species like chickpea. Higher genetic differentiation within *desi* population in contrast to *kabuli* agreed well with earlier observations (Upadhyaya et al., 2008; Bharadwaj et al., 2011; Kujur et al., 2013). Therefore, wider molecular diversity and genetic base detected by genome-wide informative SSR markers would be much relevant in the selection of desirable plant types for varietal improvement in chickpea. The admixed ancestry (∼25.8%) among six populations might be due to their complex breeding history involving inter-crossing and introgression among *desi* and *kabuli* chickpea genotypes along with strong selection pressure and evolutionary bottlenecks during chickpea domestication. Maximum admixtures and close phylogenetic relationships between *desi* and *kabuli* populations is consistent with the earlier morphological, cytological and biochemical documentation and molecular studies using SSR markers (Abbo et al., 2003; Berger et al., 2003; Sethy et al., 2006; Upadhyaya et al., 2008; Bharadwaj et al., 2011; Jhanwar et al., 2012; Kujur et al., 2013). It is also supported with the commonly accepted presumption related to origination and domestication of *desi* and *kabuli* chickpea at archeological sites of South Eastern Turkey nearly about 10000 years ago (Abbo et al., 2003; Berger et al., 2003).

The distinctness and phylogenetic relationships established by informative genome-wide SSR markers in *desi* and *kabuli* chickpea genotypes belonging to five population groups are in accordance with their cultivar-specific origin and parentage/pedigree relationships. For instance, the genotypes classified under *desi* (31) and *kabuli* (15) population groups had distinct agro-morphological features that are commonly observed in *desi* (purple flower and small seed size with yellow brown to light brown colored seed coat) and *kabuli* (white flower and large seed size with beige colored seed coat) chickpea, respectively. The grouping of one *kabuli* chickpea genotype ICC 8261 (originated from Turkey) with the *desi* genotypes (originated from India) of population group Ia reflected more influence of its geographical origin rather than cultivar-specific classification. However, complex breeding history involving introgression, cross-breeding efforts and sequential evolutionary bottlenecks among *desi* genotypes possibly led to their clustering in four population sub-groups. The informative genome-wide SSR markers developed by us are significant in establishing distinctness and evolutionary relationships as well as assaying broader molecular diversity among *desi* and *kabuli* chickpea genotypes and therefore, will be useful for many applications in chickpea genetics, genomics and breeding.

## Conclusion

We developed a large set of polymorphic SSR markers between *desi* and *kabuli* chickpea from different coding and non-CDS components of genes and intergenic regions. These genome-wide physically mapped markers with relatively high experimental validation success rate and intra-/inter-specific polymorphic potential have immense utility in large-scale genotyping applications in chickpea. A wider molecular (functional) diversity including parentage- and cultivar-specific phylogenetic relationships assayed by these informative SSR markers in a structured *desi* and *kabuli* population suggested their significance in chickpea structural, functional and comparative genomics, and breeding. We anticipate that web resource CMsDB will be very useful to scientists/breeders to search, browse and query SSRs in chickpea to facilitate molecular breeding strategies in chickpea.

## Conflict of Interest Statement

The authors declare that the research was conducted in the absence of any commercial or financial relationships that could be construed as a potential conflict of interest.

## Footnotes

1 http://nipgr.res.in/CGAP/home.php

2 http://www.icrisat.org/gt-bt/ICGGC/GenomeManuscript.htm

3 http://pgrc.ipk-gatersleben.de/misa/
