# Supplementary material for: Development of genome-wide informative simple sequence repeat markers for large-scale genotyping applications in chickpea and development of web resource
Source: Front Plant Sci. 2015 Aug 21;6:645. doi: 10.3389/fpls.2015.00645 (PMC4543896; doi:10.3389/fpls.2015.00645)
Supplement: Supplementary file 1 [file Table_1.PDF]

Supplementary Table S1. List of 1835 polymorphic simple sequence repeats between desi and kabuli chickpea genome sequences.

	Desi						Kabuli								
**SSR ID**	Linkage group/Scaffold ID	SSR type	SSR motif	SSR length (bp)	Start position	Stop position	Linkage group/Scaffold ID	SSR type	SSR motif	SSR length (bp)	Start position	Stop position	**Annotation**	**Forward primer**	**Reverse primer**
Ca_GpSSR00001	Ca_LG_1	Dinucleotide	(AT)7	14	1675513	1675526	Ca1	Dinucleotide	(AT)6	12	251285	251296	Intergenic	ATCGCGTTAAATACTTGGACT	CATATGTGGAATGGACTATGC
Ca_GpSSR00002	Ca_LG_1	Dinucleotide	(AT)14	28	718312	718339	Ca1	Dinucleotide	(AT)12	24	623176	623199	upstream:Ca_00052;	ATCGCGTTAAATACTTGGACT	CATATGTGGAATGGACTATGC
Ca_GpSSR00003	Ca_LG_1	Dinucleotide	(AT)17	34	728566	728599	Ca1	Dinucleotide	(AT)22	44	633426	633469	Intergenic	TGAATGGAGTAAATGATTACTTG	TCGCATATAGACGGATAATTT
Ca_GpSSR00004	Ca_LG_1	Dinucleotide	(AT)11	22	732476	732497	Ca1	Dinucleotide	(AT)17	34	637347	637380	gene=Ca_00055;mRNA=Ca_00055.1;intron;		
Ca_GpSSR00005	Ca_LG_1	Dinucleotide	(AT)6	12	888361	888372	Ca1	Dinucleotide	(AT)10	20	792747	792766	mRNA=Ca_00076.1;gene=Ca_00076;intron;	ATCGCGTTAAATACTTGGACT	CATATGTGGAATGGACTATGC
Ca_GpSSR00006	Ca_LG_1	Dinucleotide	(TA)13	26	890026	890051	Ca1	Dinucleotide	(TA)14	28	794387	794414	mRNA=Ca_00076.1;gene=Ca_00076;intron;	ATCGCGTTAAATACTTGGACT	CATATGTGGAATGGACTATGC
Ca_GpSSR00007	Ca_LG_1	Trinucleotide	(AAT)13	39	932198	932236	Ca1	Trinucleotide	(AAT)14	42	830681	830722	mRNA=Ca_00076.1;gene=Ca_00076;intron;	TGACCAACTTTCCTTTTAAGAT	ATTGGAAAATTCTGGTAAAGC
Ca_GpSSR00008	Ca_LG_1	Trinucleotide	(ATA)11	33	1757091	1757123	Ca1	Trinucleotide	(ATA)20	60	939741	939800	upstream:Ca_00180;	TGACCAACTTTCCTTTTAAGAT	ATTGGAAAATTCTGGTAAAGC
Ca_GpSSR00009	Ca_LG_1	Dinucleotide	(AT)16	32	1907876	1907907	Ca1	Dinucleotide	(AT)10	20	1086807	1086826	mRNA=Ca_00199.1;gene=Ca_00199;intron;	TGAATGGAGTAAATGATTACTTG	TCGCATATAGACGGATAATTT
Ca_GpSSR00010	Ca_LG_1	Dinucleotide	(AG)7	14	2590893	2590906	Ca1	Dinucleotide	(AG)9	18	2129374	2129391	gene=Ca_00273;exon=Ca_00273.1.exon1;mRNA=Ca_	ATAGTGGACCCCACAATCCA	TCGCTGTTGTGTTTTTGCTT
Ca_GpSSR00011	scaffold04648	Dinucleotide	(AT)10	20	4461	4480	Ca1	Dinucleotide	(AT)11	22	2551220	2551241	Intergenic	CCCAATATGTTTTTCTTGTTC	GAGGTTCTGGTGATGAGTACA
Ca_GpSSR00012	Ca_LG_1	Dinucleotide	(AT)11	22	2087684	2087705	Ca1	Dinucleotide	(AT)12	24	2646660	2646683	Intergenic	ATCGCGTTAAATACTTGGACT	CATATGTGGAATGGACTATGC
Ca_GpSSR00013	scaffold06994	Dinucleotide	(TA)9	18	5034	5051	Ca1	Dinucleotide	(TA)11	22	2799358	2799379	Intergenic	ATCGCGTTAAATACTTGGACT	CATATGTGGAATGGACTATGC
Ca_GpSSR00014	scaffold00126	Dinucleotide	(AT)12	24	8420	8443	Ca1	Dinucleotide	(AT)21	42	3168778	3168819	Intergenic	AAAATTTCAATTCACGGTTG	CTTGCCAATTTATCATGTTTG
Ca_GpSSR00015	Ca_LG_1	Dinucleotide	(TA)23	46	3163612	3163657	Ca1	Dinucleotide	(TA)14	28	3286600	3286627	Intergenic	ATCGCGTTAAATACTTGGACT	CATATGTGGAATGGACTATGC
Ca_GpSSR00016	Ca_LG_1	Dinucleotide	(TA)9	18	3300402	3300419	Ca1	Dinucleotide	(TA)8	16	3475739	3475754	Intergenic	ATCGCGTTAAATACTTGGACT	CATATGTGGAATGGACTATGC
Ca_GpSSR00017	Ca_LG_1	Dinucleotide	(TA)10	20	3509348	3509367	Ca1	Dinucleotide	(TA)6	12	3677591	3677602	gene=Ca_00358;mRNA=Ca_00358.1;intron;	ATCGCGTTAAATACTTGGACT	CATATGTGGAATGGACTATGC
Ca_GpSSR00018	Ca_LG_1	Dinucleotide	(TA)7	14	3688112	3688125	Ca1	Dinucleotide	(TA)6	12	3865714	3865725	Intergenic	ATCGCGTTAAATACTTGGACT	CATATGTGGAATGGACTATGC
Ca_GpSSR00019	Ca_LG_3	Trinucleotide	(AAT)33	99	15350130	15350228	Ca1	Trinucleotide	(AAT)22	66	4262746	4262811	Intergenic		
Ca_GpSSR00020	Ca_LG_3	Trinucleotide	(TAA)6	18	15350247	15350264	Ca1	Trinucleotide	(TAA)5	15	4262836	4262850	Intergenic	CCGAATCACGCTTTAATTTAT	CAAACTGCAGTAGTGTTTATGG
Ca_GpSSR00021	scaffold12185	Dinucleotide	(AT)8	16	1	16	Ca1	Dinucleotide	(AT)18	36	4612205	4612240	Intergenic	TGTTATGTGGTGCATGTAAAA	TGTCAAATGCATGAAATGTT
Ca_GpSSR00022	scaffold02872	Dinucleotide	(TA)23	46	27985	28030	Ca1	Dinucleotide	(TA)19	38	4785169	4785206	Intergenic	AAAATTTCAATTCACGGTTG	CTTGCCAATTTATCATGTTTG
Ca_GpSSR00023	scaffold00341	Dinucleotide	(AT)12	24	16023	16046	Ca1	Dinucleotide	(AT)18	36	5270952	5270987	Intergenic	TTTGATTCCCTCGTTAAATTATGA	AAAGGCTAGATCAACAACGTACaA
Ca_GpSSR00024	scaffold00341	Dinucleotide	(TA)13	26	18750	18775	Ca1	Dinucleotide	(TA)12	24	5273612	5273635	Intergenic	ATCGCGTTAAATACTTGGACT	CATATGTGGAATGGACTATGC
Ca_GpSSR00025	scaffold00341	Trinucleotide	(TAA)6	18	158328	158345	Ca1	Trinucleotide	(TAA)5	15	5403076	5403090	Intergenic	CCGAATCACGCTTTAATTTAT	CAAACTGCAGTAGTGTTTATGG
Ca_GpSSR00026	scaffold00341	Dinucleotide	(TA)13	26	185756	185781	Ca1	Dinucleotide	(TA)14	28	5432164	5432191	Intergenic	ATCGCGTTAAATACTTGGACT	CATATGTGGAATGGACTATGC
Ca_GpSSR00027	scaffold18644	Dinucleotide	(AT)7	14	709	722	Ca1	Dinucleotide	(AT)6	12	6212979	6212990	Intergenic	ATTCTCTCGTGTGGTCAATTA	CATATTTACAAGCATGTTTTGC
Ca_GpSSR00028	Ca_LG_1	Dinucleotide	(AT)19	38	5529971	5530008	Ca1	Dinucleotide	(AT)9	18	6514446	6514463	upstream:Ca_00472;	ATCGCGTTAAATACTTGGACT	CATATGTGGAATGGACTATGC
Ca_GpSSR00029	Ca_LG_1	Dinucleotide	(AT)12	24	5594747	5594770	Ca1	Dinucleotide	(AT)11	22	6580403	6580424	Intergenic	ATCGCGTTAAATACTTGGACT	CATATGTGGAATGGACTATGC
Ca_GpSSR00030	scaffold03683	Trinucleotide	(TTA)14	42	6258	6299	Ca1	Trinucleotide	(TTA)21	63	6978418	6978480	gene=Ca_18538;mRNA=Ca_18538.1;intron;	GCTCTCGGGATTGACATCAT	TTCACATCATGAACAAAAGGAAA
Ca_GpSSR00031	scaffold03683	Dinucleotide	(AT)12	24	14500	14523	Ca1	Dinucleotide	(AT)11	22	6986678	6986699	Intergenic	CACAAAATAATACAAACCATGAG	GTCCATGTCTTCGTATAGGTG
Ca_GpSSR00032	scaffold07076	Dinucleotide	(AT)12	24	1	24	Ca1	Dinucleotide	(AT)10	20	7082140	7082159	Intergenic	ATCGCGTTAAATACTTGGACT	CATATGTGGAATGGACTATGC
Ca_GpSSR00033	scaffold05381	Dinucleotide	(AT)23	46	4528	4573	Ca1	Dinucleotide	(AT)21	42	7582814	7582855	Intergenic	AAAATTTCAATTCACGGTTG	CTTGCCAATTTATCATGTTTG
Ca_GpSSR00034	scaffold50499	Dinucleotide	(AT)19	38	654	691	Ca1	Dinucleotide	(AT)16	32	7953501	7953532	Intergenic		
Ca_GpSSR00035	scaffold15726	Trinucleotide	(TAT)11	33	2395	2427	Ca1	Trinucleotide	(TAT)29	87	8311802	8311888	Intergenic	CCTACGTGGACCCTCTTATTA	GCTTGTGTGGTTGATTTTATC
Ca_GpSSR00036	scaffold10946	Trinucleotide	(TTA)23	69	16	84	Ca1	Trinucleotide	(TTA)16	48	8328466	8328513	Intergenic	AATTAACCAACCAATCCTACG	GCTTGTGTGGTTGATTTTATC
Ca_GpSSR00037	Ca_LG_1	Trinucleotide	(TTA)8	24	2807885	2807908	Ca1	Trinucleotide	(TTA)7	21	8372333	8372353	upstream:Ca_00280;		
Ca_GpSSR00038	Ca_LG_1	Dinucleotide	(AT)11	22	2813153	2813174	Ca1	Dinucleotide	(AT)25	50	8377748	8377797	Intergenic	CTGTTACGTGCAATGGATGC	TCGGTATGACACAAAAATGTGA
Ca_GpSSR00039	Ca_LG_1	Dinucleotide	(TA)16	32	2981371	2981402	Ca1	Dinucleotide	(TA)6	12	8556463	8556474	Intergenic	TGAATGGAGTAAATGATTACTTG	TCGCATATAGACGGATAATTT
Ca_GpSSR00040	Ca_LG_1	Dinucleotide	(AT)10	20	13639938	13639957	Ca1	Dinucleotide	(AT)13	26	9758418	9758443	upstream:Ca_00971;	ATCGCGTTAAATACTTGGACT	CATATGTGGAATGGACTATGC
Ca_GpSSR00041	scaffold01972	Dinucleotide	(TA)6	12	45983	45994	Ca1	Dinucleotide	(TA)7	14	11072399	11072412	Intergenic	ATCGCGTTAAATACTTGGACT	CATATGTGGAATGGACTATGC
Ca_GpSSR00042	scaffold52833	Dinucleotide	(AT)10	20	631	650	Ca1	Dinucleotide	(AT)11	22	11162120	11162141	Intergenic	ATCGCGTTAAATACTTGGACT	CATATGTGGAATGGACTATGC
Ca_GpSSR00043	scaffold11096	Dinucleotide	(TA)8	16	2350	2365	Ca1	Dinucleotide	(TA)9	18	11557994	11558011	Intergenic	CTTAACTCTTGATTCCACCTCT	CCAAAAAGCTATGCTGACTTA
Ca_GpSSR00044	scaffold12020	Dinucleotide	(AT)6	12	283	294	Ca1	Dinucleotide	(AT)21	42	11633253	11633294	Intergenic	AAAATTTCAATTCACGGTTG	CTTGCCAATTTATCATGTTTG
Ca_GpSSR00045	Ca_LG_1	Dinucleotide	(AT)13	26	12006995	12007020	Ca1	Dinucleotide	(AT)10	20	12092744	12092763	upstream:Ca_00914;	ATCGCGTTAAATACTTGGACT	CATATGTGGAATGGACTATGC
Ca_GpSSR00046	scaffold02857	Dinucleotide	(TA)10	20	12895	12914	Ca1	Dinucleotide	(TA)8	16	12429468	12429483	upstream:Ca_17925;	ACTCATCCAAAGTGGGTAAAT	AAAGGATTTTGGTATCTCTGT
Ca_GpSSR00047	Ca_LG_1	Dinucleotide	(TA)29	58	11527398	11527455	Ca1	Dinucleotide	(TA)15	30	12645726	12645755	Intergenic		
Ca_GpSSR00048	scaffold01772	Dinucleotide	(AT)11	22	10428	10449	Ca1	Dinucleotide	(AT)12	24	12721903	12721926	Intergenic	CGAATTCAACTCATAACCAAT	TCATTGAAAAGATCGTCAAAC
Ca_GpSSR00049	scaffold53039	Dinucleotide	(TA)21	42	22	63	Ca1	Dinucleotide	(TA)8	16	13367333	13367348	Intergenic	ATCGCGTTAAATACTTGGACT	CATATGTGGAATGGACTATGC
Ca_GpSSR00050	Ca_LG_1	Dinucleotide	(TA)8	16	11064897	11064912	Ca1	Dinucleotide	(TA)14	28	13417844	13417871	Intergenic	ATCGCGTTAAATACTTGGACT	CATATGTGGAATGGACTATGC
Ca_GpSSR00051	scaffold00186	Dinucleotide	(TC)9	18	110519	110536	Ca1	Dinucleotide	(TC)10	20	13578764	13578783	exon=Ca_11189.1.exon1;gene=Ca_11189;5'-UTR=Ca_	CATCTGAGAAAAAGCTGAAAA	GTTTTCAAGGTCAAACTCCAT
Ca_GpSSR00052	scaffold62646	Dinucleotide	(AT)8	16	500	515	Ca1	Dinucleotide	(AT)22	44	14502774	14502817	Intergenic	AAAATTTCAATTCACGGTTG	CTTGCCAATTTATCATGTTTG
Ca_GpSSR00053	scaffold01841	Dinucleotide	(AT)7	14	7124	7137	Ca1	Dinucleotide	(AT)6	12	14779175	14779186	Intergenic	TTTGTAGTATAGCAATTTGGTGA	TCATTAGTGTCGTTTGTTGAA
Ca_GpSSR00054	scaffold113477	Dinucleotide	(AT)15	30	306	335	Ca1	Dinucleotide	(AT)16	32	14880860	14880891	Intergenic		
Ca_GpSSR00055	Ca_LG_1	Dinucleotide	(CT)7	14	10210848	10210861	Ca1	Dinucleotide	(CT)8	16	15022873	15022888	Intergenic	TCATACACAGAAAAGCAAACA	GGATGATATGGTATGGTTTGT
Ca_GpSSR00056	Ca_LG_1	Dinucleotide	(AT)8	16	10223659	10223674	Ca1	Dinucleotide	(AT)7	14	15037302	15037315	upstream:Ca_00758;	ATCGCGTTAAATACTTGGACT	CATATGTGGAATGGACTATGC
Ca_GpSSR00057	Ca_LG_1	Trinucleotide	(AAT)46	138	10080372	10080509	Ca1	Trinucleotide	(AAT)22	66	15237771	15237836	Intergenic		
Ca_GpSSR00058	Ca_LG_1	Trinucleotide	(TAA)43	129	9926791	9926919	Ca1	Trinucleotide	(TAA)19	57	15539756	15539812	Intergenic		
Ca_GpSSR00059	scaffold21907	Dinucleotide	(AT)13	26	83	108	Ca1	Dinucleotide	(AT)28	56	15542865	15542920	Intergenic	GACCTAGTCCGCGACTCAAC	AgAcCCaAACATGTCGGTaAA
Ca_GpSSR00060	Ca_LG_1	Dinucleotide	(AT)7	14	9952953	9952966	Ca1	Dinucleotide	(AT)6	12	15566516	15566527	Intergenic	ATCGCGTTAAATACTTGGACT	CATATGTGGAATGGACTATGC
Ca_GpSSR00061	Ca_LG_1	Dinucleotide	(AT)8	16	9968086	9968101	Ca1	Dinucleotide	(AT)9	18	15579151	15579168	Intergenic	ATCGCGTTAAATACTTGGACT	CATATGTGGAATGGACTATGC
Ca_GpSSR00062	scaffold01335	Dinucleotide	(AT)10	20	53353	53372	Ca1	Dinucleotide	(AT)11	22	15749343	15749364	Intergenic	ATTAGGCTACACCACATTCAA	TGAGATGAATCATGTAATAAGCA
Ca_GpSSR00063	scaffold07934	Dinucleotide	(AT)17	34	6963	6996	Ca1	Dinucleotide	(AT)12	24	15890821	15890844	Intergenic	ATCGCGTTAAATACTTGGACT	CATATGTGGAATGGACTATGC
Ca_GpSSR00064	scaffold00360	Trinucleotide	(TTA)14	42	145166	145207	Ca1	Trinucleotide	(TTA)17	51	15928923	15928973	Intergenic	CGACCATCGTCATATATTTTT	TTGTGTTTCCCAAATTTTTC
Ca_GpSSR00065	scaffold00360	Dinucleotide	(AT)11	22	167183	167204	Ca1	Dinucleotide	(AT)8	16	15951061	15951076	Intergenic	ATCGCGTTAAATACTTGGACT	CATATGTGGAATGGACTATGC
Ca_GpSSR00066	scaffold00360	Dinucleotide	(AT)10	20	181142	181161	Ca1	Dinucleotide	(AT)8	16	15971213	15971228	mRNA=Ca_12307.1;gene=Ca_12307;intron;	ATCGCGTTAAATACTTGGACT	CATATGTGGAATGGACTATGC
Ca_GpSSR00067	scaffold01760	Dinucleotide	(AG)16	32	1	32	Ca1	Dinucleotide	(AG)64	128	16108848	16108975	upstream:Ca_16396;		
Ca_GpSSR00068	scaffold03415	Dinucleotide	(TA)8	16	7031	7046	Ca1	Dinucleotide	(TA)26	52	16177047	16177098	Intergenic	CTTCATGTGAACTTATTTGTATG	GTGTATTTCATTTAGTCTCGAA
Ca_GpSSR00069	scaffold00968	Dinucleotide	(AT)8	16	654	669	Ca1	Dinucleotide	(AT)6	12	16199746	16199757	Intergenic	CGTCTTACCTCGTAAAAGTCA	TTTGTTATCTTGCCATTGAGT
Ca_GpSSR00070	scaffold00968	Trinucleotide	(TTA)6	18	17779	17796	Ca1	Trinucleotide	(TTA)7	21	16216790	16216810	Intergenic	TGTGTTATTGCAAACATTGTC	GCCAGCTCTATTCACAATATG
Ca_GpSSR00071	scaffold00968	Trinucleotide	(TTA)15	45	56074	56118	Ca1	Trinucleotide	(TTA)11	33	16244056	16244088	Intergenic	TGTGTTATTGCAAACATTGTC	GCCAGCTCTATTCACAATATG
Ca_GpSSR00072	scaffold00968	Dinucleotide	(AT)17	34	72718	72751	Ca1	Dinucleotide	(AT)18	36	16260714	16260749	upstream:Ca_14641;		
Ca_GpSSR00073	scaffold00205	Dinucleotide	(TA)25	50	150507	150556	Ca1	Dinucleotide	(TA)17	34	16440166	16440199	Intergenic		
Ca_GpSSR00074	scaffold02503	Dinucleotide	(TA)9	18	21957	21974	Ca1	Dinucleotide	(TA)8	16	16562075	16562090	Intergenic	TGAAAACAAAGAGAGGTTTCA	TGAGAATTATCTCTTTACGATAG
Ca_GpSSR00075	scaffold04014	Trinucleotide	(AAT)8	24	7922	7945	Ca1	Trinucleotide	(AAT)7	21	17208109	17208129	Intergenic	CAGAAATGCAGAACATAATCC	CATCGTCAAAGATCTCATCTC
Ca_GpSSR00076	scaffold04948	Trinucleotide	(TTA)29	87	8346	8432	Ca1	Trinucleotide	(TTA)21	63	17235425	17235487	Intergenic	CCTTTCCTTTTGATTAGGTTT	GGACTAATGACGTTAAAATCCA
Ca_GpSSR00077	scaffold04948	Dinucleotide	(AT)8	16	12268	12283	Ca1	Dinucleotide	(AT)27	54	17239312	17239365	Intergenic	TTAGATTGGATTCGCAGATAA	AGTGTTTTGTTGTATTTGTGGA
Ca_GpSSR00078	scaffold32683	Dinucleotide	(AT)19	38	4	41	Ca1	Dinucleotide	(AT)17	34	17277505	17277538	Intergenic		

Ca_GpSSR00079	scaffold06222	Dinucleotide	(AT)10	20	1	20	Ca1	Dinucleotide	(AT)7	14	17334909	17334922	Intergenic	ATCGCGTTAAATACTTGGACT	CATATGTGGAATGGACTATGC
Ca_GpSSR00080	scaffold04496	Dinucleotide	(TA)10	20	12652	12671	Ca1	Dinucleotide	(TA)11	22	17656897	17656918	Intergenic	ATCGCGTTAAATACTTGGACT	CATATGTGGAATGGACTATGC
Ca_GpSSR00081	Ca_LG_1	Dinucleotide	(AT)11	22	6783655	6783676	Ca1	Dinucleotide	(AT)16	32	17729267	17729298	Intergenic	TCATGCAACAGACGAATTGA	AACATGTTATAAAACGTAAGGCG
Ca_GpSSR00082	scaffold03025	Dinucleotide	(AT)11	22	3265	3286	Ca1	Dinucleotide	(AT)12	24	17920631	17920654	Intergenic	TTACTCACATCATTTCCCTTG	GAAATGTTTAGTTGCTCTCACA
Ca_GpSSR00083	scaffold00286	Dinucleotide	(AT)15	30	4055	4084	Ca1	Dinucleotide	(AT)10	20	18010520	18010539	Intergenic	ATCGCGTTAAATACTTGGACT	CATATGTGGAATGGACTATGC
Ca_GpSSR00084	scaffold00286	Dinucleotide	(TC)15	30	161986	162015	Ca1	Dinucleotide	(TC)13	26	18170639	18170664	Intergenic	ATTGCAAGTTTAGCCCTCTAT	AAAACATCTCACGTCATCATT
Ca_GpSSR00085	scaffold02877	Dinucleotide	(TA)8	16	17846	17861	Ca1	Dinucleotide	(TA)9	18	18444162	18444179	Intergenic	GGTGCTGGACAATTTATTTCT	CTCATGACTCCCAATCAAATA
Ca_GpSSR00086	scaffold08950	Dinucleotide	(AT)17	34	60	93	Ca1	Dinucleotide	(AT)18	36	18580998	18581033	Intergenic		
Ca_GpSSR00087	scaffold00725	Dinucleotide	(AT)10	20	86720	86739	Ca1	Dinucleotide	(AT)9	18	18966470	18966487	Intergenic	AATAAGTGAATGCTTCAATGTG	AATTTAAGCTTTTACCATCTCG
Ca_GpSSR00088	scaffold01070	Trinucleotide	(AAT)16	48	46674	46721	Ca1	Trinucleotide	(AAT)21	63	19020342	19020404	Intergenic		
Ca_GpSSR00089	scaffold00384	Dinucleotide	(TA)9	18	136054	136071	Ca1	Dinucleotide	(TA)8	16	19314928	19314943	Intergenic	TCATGTATTGTTTTGTCGAGTT	TCCAAAATATCTTGCTCTTCA
Ca_GpSSR00090	scaffold00795	Dinucleotide	(GA)11	22	52302	52323	Ca1	Dinucleotide	(GA)15	30	19550771	19550800	mRNA=Ca_14080.3;gene=Ca_14080;intron;	aGCGAAAATTCGAGAAACGA	ACTCCCCACCACtTTCCTCT
Ca_GpSSR00091	scaffold00795	Dinucleotide	(AG)10	20	73052	73071	Ca1	Dinucleotide	(AG)12	24	19574561	19574584	mRNA=Ca_14081.2;5'-UTR=Ca_14081.2.5'-UTR1;gene	CCAAAGCAAGAATTACACGC	CTTTACATTTACGCCCCCAA
Ca_GpSSR00092	scaffold00795	Dinucleotide	(TA)9	18	97023	97040	Ca1	Dinucleotide	(TA)15	30	19598101	19598130	Intergenic		
Ca_GpSSR00093	scaffold15263	Dinucleotide	(AT)6	12	2138	2149	Ca1	Dinucleotide	(AT)25	50	19631399	19631448	Intergenic	TCCTTTGTACTAAAGCAAACC	TAACATACGCGTTGCGAATA
Ca_GpSSR00094	scaffold00046	Dinucleotide	(AT)25	50	31876	31925	Ca1	Dinucleotide	(AT)29	58	19741393	19741450	Intergenic	TCTAACAAGTTGATCGTCAAAA	ATTGGCTACGATTTATTGTGT
Ca_GpSSR00095	scaffold00046	Dinucleotide	(AG)6	12	69018	69029	Ca1	Dinucleotide	(AG)7	14	19782647	19782660	gene=Ca_08747;5'-UTR=Ca_08747.1.5'-UTR1;exon=C	GTAAGGATTTCTAGGGACGTG	TTCGTCTTTCTTTTCTTTCTTC
Ca_GpSSR00096	scaffold00647	Dinucleotide	(AT)16	32	30709	30740	Ca1	Dinucleotide	(AT)26	52	20115911	20115962	Intergenic	CAATCAAGCTCGTATGGAACA	CATCAATTATTCAACTACCAAtTTTC
Ca_GpSSR00097	Ca_LG_8	Dinucleotide	(AT)10	20	7984111	7984130	Ca1	Dinucleotide	(AT)9	18	20182893	20182910	Intergenic	CCACTAGTATTATGGACAAATCA	GTGGTTTGCAAGGTTTTATATT
Ca_GpSSR00098	scaffold11992	Trinucleotide	(TAA)35	105	3872	3976	Ca1	Trinucleotide	(TAA)34	102	20243002	20243103	Intergenic		
Ca_GpSSR00099	Ca_LG_7	Trinucleotide	(TAT)11	33	2721252	2721284	Ca1	Trinucleotide	(TAT)10	30	20300791	20300820	upstream:Ca_07560;	AAGTTGTTCTTGTTGTTACCG	GAACATTTCTGCGAAAATAA
Ca_GpSSR00100	Ca_LG_7	Dinucleotide	(AT)12	24	2906256	2906279	Ca1	Dinucleotide	(AT)13	26	20494445	20494470	Intergenic	ATCGCGTTAAATACTTGGACT	CATATGTGGAATGGACTATGC
Ca_GpSSR00101	scaffold32514	Dinucleotide	(AT)10	20	733	752	Ca1	Dinucleotide	(AT)9	18	20534236	20534253	Intergenic	GATCAGCTCTTGTTTGATTTG	TGTCATTCATCACATTTATGG
Ca_GpSSR00102	scaffold09316	Dinucleotide	(AT)23	46	5579	5624	Ca1	Dinucleotide	(AT)12	24	20729491	20729514	Intergenic	ATCGCGTTAAATACTTGGACT	CATATGTGGAATGGACTATGC
Ca_GpSSR00103	scaffold01583	Dinucleotide	(AT)7	14	18737	18750	Ca1	Dinucleotide	(AT)8	16	20875711	20875726	Intergenic	GGATAACTTTCATTGCAACAG	ATATCTTGCTCCAATCAGCTT
Ca_GpSSR00104	Ca_LG_8	Trinucleotide	(TAA)12	36	8900588	8900623	Ca1	Trinucleotide	(TAA)13	39	21347593	21347631	Intergenic		
Ca_GpSSR00105	Ca_LG_8	Dinucleotide	(TA)14	28	9073186	9073213	Ca1	Dinucleotide	(TA)20	40	21511797	21511836	Intergenic	TTTACCGGTGTAAATGGAC	TGGTATCATGTGTTTGATGTG
Ca_GpSSR00106	Ca_LG_8	Trinucleotide	(TAT)28	84	9092309	9092392	Ca1	Trinucleotide	(TAT)17	51	21529812	21529862	Intergenic	CCTACGTGGACCCTCTTATTA	GCTTGTGTGGTTGATTTTATC
Ca_GpSSR00107	Ca_LG_1	Dinucleotide	(TA)8	16	7010541	7010556	Ca1	Dinucleotide	(TA)7	14	21820325	21820338	Intergenic	ATCGCGTTAAATACTTGGACT	CATATGTGGAATGGACTATGC
Ca_GpSSR00108	scaffold03076	Dinucleotide	(TA)12	24	31262	31285	Ca1	Dinucleotide	(TA)13	26	22002154	22002179	Intergenic	TCAATCTCTCACGCATTTTAC	TTACAGTTGAAAAGAGGGAAA
Ca_GpSSR00109	scaffold03076	Trinucleotide	(TTA)12	36	8849	8884	Ca1	Trinucleotide	(TTA)6	18	22024636	22024653	upstream:Ca_18127;		
Ca_GpSSR00110	scaffold03076	Dinucleotide	(TA)10	20	4347	4366	Ca1	Dinucleotide	(TA)14	28	22029012	22029039	Intergenic	TCAATCTCTCACGCATTTTAC	TTACAGTTGAAAAGAGGGAAA
Ca_GpSSR00111	scaffold92585	Dinucleotide	(TA)14	28	348	375	Ca1	Dinucleotide	(TA)21	42	22100596	22100637	Intergenic	AAAATTTCAATTCACGGTTG	CTTGCCAATTTATCATGTTTG
Ca_GpSSR00112	scaffold01916	Dinucleotide	(AT)8	16	12652	12667	Ca1	Dinucleotide	(AT)9	18	22189989	22190006	Intergenic	AAACAGCTTAGCACTTTGATG	CAACTAAAAAGAAAAGGAGTCTG
Ca_GpSSR00113	scaffold02568	Dinucleotide	(AT)28	56	15444	15499	Ca1	Dinucleotide	(AT)13	26	22226170	22226195	gene=Ca_17611;mRNA=Ca_17610.1;mRNA=Ca_1761	TCTTACCATCACGAAATGTTGC	GTTGAATGATAATTGGTCAGACTTT
Ca_GpSSR00114	Ca_LG_1	Trinucleotide	(TTA)25	75	7150246	7150320	Ca1	Trinucleotide	(TTA)12	36	22326881	22326916	Intergenic	TGTGACAAAaCAAaTAACGTGTCA	CGTCAAAGTTAACGCAGTTGAG
Ca_GpSSR00115	Ca_LG_1	Dinucleotide	(AT)18	36	7159798	7159833	Ca1	Dinucleotide	(AT)17	34	22335553	22335586	Intergenic		
Ca_GpSSR00116	scaffold08139	Trinucleotide	(AAT)15	45	6743	6787	Ca1	Trinucleotide	(AAT)18	54	22459728	22459781	Intergenic		
Ca_GpSSR00117	scaffold01234	Dinucleotide	(AT)10	20	5429	5448	Ca1	Dinucleotide	(AT)9	18	22757867	22757884	Intergenic	ATCGCGTTAAATACTTGGACT	CATATGTGGAATGGACTATGC
Ca_GpSSR00118	scaffold01480	Dinucleotide	(AT)10	20	13295	13314	Ca1	Dinucleotide	(AT)9	18	23304826	23304843	Intergenic	TAAAATTAGAACATGCCTCCA	GATTTTCCACCCTCTAATTGT
Ca_GpSSR00119	scaffold01480	Dinucleotide	(AT)11	22	14465	14486	Ca1	Dinucleotide	(AT)12	24	23305994	23306017	Intergenic	CCATCCTACAAACAAAGTTCA	ATAATTACACGACAACGCAAG
Ca_GpSSR00120	scaffold01704	Dinucleotide	(TA)15	30	89770	89799	Ca1	Dinucleotide	(TA)9	18	23573198	23573215	Intergenic	CAAAACCAAAAGTGTTGTAGG	CTCTTGATGTTGGTTTTTCTG
Ca_GpSSR00121	scaffold01704	Trinucleotide	(AAT)5	15	167393	167407	Ca1	Trinucleotide	(AAT)21	63	23641483	23641545	Intergenic	TGTGTTTCATGGGTCAGGTC	TCGtAAGtTTTTATTTATCAACGGG
Ca_GpSSR00122	scaffold03027	Dinucleotide	(TA)6	12	8781	8792	Ca1	Dinucleotide	(TA)7	14	23714877	23714890	Intergenic	GCACATGTGAGTGTGTGACTA	CTTCAAGATTTAGTCGGAAAA
Ca_GpSSR00123	Ca_LG_1	Dinucleotide	(AT)15	30	7928571	7928600	Ca1	Dinucleotide	(AT)17	34	24856704	24856737	Intergenic	TGAATGGAGTAAATGATTACTTG	TCGCATATAGACGGATAATTT
Ca_GpSSR00124	scaffold06223	Dinucleotide	(TA)11	22	2853	2874	Ca1	Dinucleotide	(TA)20	40	25306083	25306122	Intergenic	AAAATTTCAATTCACGGTTG	CTTGCCAATTTATCATGTTTG
Ca_GpSSR00125	scaffold00676	Dinucleotide	(AT)23	46	25043	25088	Ca1	Dinucleotide	(AT)28	56	25711490	25711545	Intergenic	TCTAACAAGTTGATCGTCAAAA	ATTGGCTACGATTTATTGTGT
Ca_GpSSR00126	scaffold05377	Dinucleotide	(AT)15	30	2341	2370	Ca1	Dinucleotide	(AT)16	32	26511146	26511177	Intergenic	TTTTATGAGCGTCCTCTTTTT	TAGGAGTAGCTTCTTGGAGTG
Ca_GpSSR00127	scaffold11428	Dinucleotide	(TA)10	20	1245	1264	Ca1	Dinucleotide	(TA)12	24	26577310	26577333	Intergenic	AATCACCACAATGATAAGGAA	CTTCTACCAATCAGTCCATTG
Ca_GpSSR00128	Ca_LG_2	Dinucleotide	(AT)18	36	5284151	5284186	Ca1	Dinucleotide	(AT)12	24	26624005	26624028	Intergenic	ATCGCGTTAAATACTTGGACT	CATATGTGGAATGGACTATGC
Ca_GpSSR00129	scaffold01670	Trinucleotide	(TAT)11	33	13773	13805	Ca1	Trinucleotide	(TAT)12	36	26945132	26945167	upstream:Ca_16256;	CAAATAAACCATTTCACCAA	TTCTAAGCATTCTTTTTCCT
Ca_GpSSR00130	scaffold01056	Trinucleotide	(TAA)49	147	24278	24424	Ca1	Trinucleotide	(TAA)13	39	28480343	28480381	downstream:Ca_09964;		
Ca_GpSSR00131	scaffold01056	Dinucleotide	(AT)28	56	189531	189586	Ca1	Dinucleotide	(AT)22	44	28705697	28705740	Intergenic	AAAATTTCAATTCACGGTTG	CTTGCCAATTTATCATGTTTG
Ca_GpSSR00132	scaffold09348	Dinucleotide	(AT)9	18	185922	185939	Ca1	Dinucleotide	(AT)10	20	29246695	29246714	Intergenic	GGTGTTGGCTCTATTTTTCTT	GTCGGAGGTAATGATGATTTT
Ca_GpSSR00133	scaffold01099	Dinucleotide	(AT)16	32	29357	29388	Ca1	Dinucleotide	(AT)26	52	30693052	30693103	gene=Ca_14952;mRNA=Ca_14952.1;intron;	TTAGAGAGGGAGAGTCCAAGT	TCACTCACGTGTATGTGTGAT
Ca_GpSSR00134	scaffold00845	Dinucleotide	(AT)16	32	38444	38475	Ca1	Dinucleotide	(AT)10	20	30828736	30828755	Intergenic	TCACACATTTTAACCTAACCGC	TGTGTGTGTGtGTTTGTGTGTG
Ca_GpSSR00135	scaffold00934	Dinucleotide	(AT)11	22	194455	194476	Ca1	Dinucleotide	(AT)6	12	31113752	31113763	Intergenic	ATCGCGTTAAATACTTGGACT	CATATGTGGAATGGACTATGC
Ca_GpSSR00136	scaffold02981	Dinucleotide	(TA)7	14	4692	4705	Ca1	Dinucleotide	(TA)6	12	32153738	32153749	Intergenic	TTTAGTTTAACGGGAGAATCC	TCCTCCATCTTGGGTAATTT
Ca_GpSSR00137	scaffold02300	Dinucleotide	(AT)12	24	37317	37340	Ca1	Dinucleotide	(AT)9	18	32897409	32897426	Intergenic	ATCGCGTTAAATACTTGGACT	CATATGTGGAATGGACTATGC
Ca_GpSSR00138	scaffold03175	Dinucleotide	(AT)12	24	1101	1124	Ca1	Dinucleotide	(AT)13	26	34191467	34191492	Intergenic	CTTTATTCTTTTGTGGTGACG	TCAAAGTACTAACCAGCGATT
Ca_GpSSR00139	scaffold84348	Dinucleotide	(AT)16	32	378	409	Ca1	Dinucleotide	(AT)18	36	34228060	34228095	Intergenic		
Ca_GpSSR00140	scaffold01687	Dinucleotide	(AT)14	28	10922	10949	Ca1	Dinucleotide	(AT)13	26	34293086	34293111	Intergenic	GTTTCTCTTTGCCAACTTTTC	GAAAAGCCTATGTGTCGTAAA
Ca_GpSSR00141	scaffold10990	Dinucleotide	(AT)7	14	55723	55736	Ca1	Dinucleotide	(AT)6	12	34747379	34747390	upstream:Ca_09604;	ATCGCGTTAAATACTTGGACT	CATATGTGGAATGGACTATGC
Ca_GpSSR00142	scaffold10990	Dinucleotide	(CT)9	18	56307	56324	Ca1	Dinucleotide	(CT)10	20	34747961	34747980	mRNA=Ca_09604.1;gene=Ca_09604;intron;	TTTCCGTAACAACACAAAAAC	CACTAGGTTTGAAAGTGGAAA
Ca_GpSSR00143	scaffold04517	Dinucleotide	(TA)20	40	13734	13773	Ca1	Dinucleotide	(TA)9	18	36306316	36306333	Intergenic	ATCGCGTTAAATACTTGGACT	CATATGTGGAATGGACTATGC
Ca_GpSSR00144	scaffold02542	Dinucleotide	(TA)8	16	40876	40891	Ca1	Dinucleotide	(TA)9	18	37293470	37293487	Intergenic	ATCGCGTTAAATACTTGGACT	CATATGTGGAATGGACTATGC
Ca_GpSSR00145	scaffold60246	Dinucleotide	(AT)9	18	490	507	Ca1	Dinucleotide	(AT)11	22	37424330	37424351	Intergenic	ATCGCGTTAAATACTTGGACT	CATATGTGGAATGGACTATGC
Ca_GpSSR00146	scaffold01007	Dinucleotide	(AT)26	52	43756	43807	Ca1	Dinucleotide	(AT)8	16	38750885	38750900	Intergenic	TCACAACAGTTGACATTATACTC	CGGAACAATCTCTCAAAAGTT
Ca_GpSSR00147	scaffold14328	Dinucleotide	(TA)20	40	45	84	Ca1	Dinucleotide	(TA)18	36	39038587	39038622	Intergenic		
Ca_GpSSR00148	scaffold02673	Dinucleotide	(AT)14	28	5120	5147	Ca1	Dinucleotide	(AT)12	24	39596654	39596677	Intergenic	GACACATGATAACTTAACACACG	GGAAGTAGATGAATGAATAATGG
Ca_GpSSR00149	scaffold01844	Trinucleotide	(ATT)25	75	51397	51471	Ca1	Trinucleotide	(ATT)20	60	40048662	40048721	Intergenic	ACATATCCTTTGTGAAAGTACAA	TTTTGTGTGTGTACATCTTTCA
Ca_GpSSR00150	scaffold01164	Dinucleotide	(AT)11	22	60769	60790	Ca1	Dinucleotide	(AT)10	20	40475885	40475904	Intergenic	CTTCTTGGAATGAAATCACAG	AGTGGTAAGAATTGGAAATGG
Ca_GpSSR00151	scaffold18197	Dinucleotide	(TA)21	42	1	42	Ca1	Dinucleotide	(TA)17	34	40927425	40927458	Intergenic	CTTGAGTTTCTTTTGCATTT	GTGGTTGTCTCTCTTCTTCCT
Ca_GpSSR00152	scaffold00206	Trinucleotide	(ATA)15	45	129608	129652	Ca1	Trinucleotide	(ATA)16	48	41025674	41025721	Intergenic		
Ca_GpSSR00153	scaffold00206	Dinucleotide	(AT)16	32	147017	147048	Ca1	Dinucleotide	(AT)15	30	41043358	41043387	Intergenic		
Ca_GpSSR00154	scaffold00206	Trinucleotide	(TAA)26	78	181997	182074	Ca1	Trinucleotide	(TAA)19	57	41069980	41070036	Intergenic	CCCTCAAGCAACCCATAAAT	TTGACACCATATGTGTTCTCCC
Ca_GpSSR00155	Ca_LG_1	Dinucleotide	(TA)7	14	9009356	9009369	Ca1	Dinucleotide	(TA)19	38	42003292	42003329	Intergenic	AAAATTTCAATTCACGGTTG	CTTGCCAATTTATCATGTTTG
Ca_GpSSR00156	scaffold08346	Dinucleotide	(AT)8	16	2156	2171	Ca1	Dinucleotide	(AT)7	14	42380092	42380105	Intergenic	ATCGCGTTAAATACTTGGACT	CATATGTGGAATGGACTATGC
Ca_GpSSR00157	scaffold04370	Dinucleotide	(AT)10	20	2065	2084	Ca1	Dinucleotide	(AT)11	22	42432937	42432958	Intergenic	CAGCAAAATTACATGAGTCAA	TGAATTGCACACTCATATCAA
Ca_GpSSR00158	Ca_LG_2	Dinucleotide	(TA)20	40	132909	132948	Ca1	Dinucleotide	(TA)9	18	43101961	43101978	Intergenic	ATCGCGTTAAATACTTGGACT	CATATGTGGAATGGACTATGC
Ca_GpSSR00159	Ca_LG_2	Dinucleotide	(TA)20	40	132656	132695	Ca1	Dinucleotide	(TA)17	34	43102414	43102447	Intergenic		

Ca_GpSSR00160	scaffold01182	Dinucleotide	(AT)6	12	38867	38878	Ca1	Dinucleotide	(AT)9	18	43396382	43396399	Intergenic	GGAAATATGTTTAAGGGGTGT	TTTATGCTTCTGATTCATTCC
Ca_GpSSR00161	scaffold01182	Dinucleotide	(AT)25	50	37708	37757	Ca1	Dinucleotide	(AT)10	20	43397528	43397547	Intergenic	ATCGCGTTAAATACTTGGACT	CATATGTGGAATGGACTATGC
Ca_GpSSR00162	scaffold03095	Dinucleotide	(AT)10	20	31103	31122	Ca1	Dinucleotide	(AT)11	22	43735532	43735553	Intergenic	ATAATGATGGCAAGATGATTG	TGTACTTAACGTGTCTATTTTGT
Ca_GpSSR00163	scaffold02589	Dinucleotide	(AT)12	24	15879	15902	Ca1	Dinucleotide	(AT)13	26	44080229	44080254	upstream:Ca_10025;	ATCGCGTTAAATACTTGGACT	CATATGTGGAATGGACTATGC
Ca_GpSSR00164	scaffold42336	Trinucleotide	(ATA)9	27	1	27	Ca1	Trinucleotide	(ATA)18	54	44102710	44102763	Intergenic		
Ca_GpSSR00165	scaffold02589	Trinucleotide	(TTA)5	15	41137	41151	Ca1	Trinucleotide	(TTA)6	18	44103854	44103871	Intergenic		
Ca_GpSSR00166	Ca_LG_6	Trinucleotide	(ATT)12	36	8851431	8851466	Ca1	Trinucleotide	(ATT)13	39	44556140	44556178	Intergenic		
Ca_GpSSR00167	scaffold03524	Trinucleotide	(ATT)11	33	1020	1052	Ca1	Trinucleotide	(ATT)5	15	45519153	45519167	Intergenic		
Ca_GpSSR00168	scaffold65483	Trinucleotide	(ATA)21	63	302	364	Ca1	Trinucleotide	(ATA)20	60	45570231	45570290	Intergenic		
Ca_GpSSR00169	scaffold08052	Dinucleotide	(AT)14	28	281	308	Ca1	Dinucleotide	(AT)15	30	46834641	46834670	Intergenic		
Ca_GpSSR00170	scaffold00448	Dinucleotide	(AT)12	24	63389	63412	Ca1	Dinucleotide	(AT)7	14	46991396	46991409	Intergenic	ATCGCGTTAAATACTTGGACT	CATATGTGGAATGGACTATGC
Ca_GpSSR00171	scaffold18266	Dinucleotide	(TA)18	36	1862	1897	Ca1	Dinucleotide	(TA)19	38	47266283	47266320	Intergenic	AAAATTTCAATTCACGGTTG	CTTGCCAATTTATCATGTTTG
Ca_GpSSR00172	scaffold05802	Dinucleotide	(AT)20	40	5731	5770	Ca1	Dinucleotide	(AT)19	38	47370417	47370454	Intergenic	AAAATTTCAATTCACGGTTG	CTTGCCAATTTATCATGTTTG
Ca_GpSSR00173	Ca_LG_1	Dinucleotide	(AT)16	32	9787142	9787173	Ca1	Dinucleotide	(AT)22	44	47415169	47415212	downstream:Ca_00721;	tGtCAcAAaCATGTAaGaCAGTtGTT	AAGATTTGGCTGGCTTCATC
Ca_GpSSR00174	scaffold01134	Trinucleotide	(TAT)19	57	11030	11086	Ca1	Trinucleotide	(TAT)26	78	47655104	47655181	Intergenic	ATTTGTAGGGATGTGAACCA	CATTGTTTTGAAAAGACTTGAAC
Ca_GpSSR00175	scaffold01134	Dinucleotide	(TC)7	14	58762	58775	Ca1	Dinucleotide	(TC)8	16	47699226	47699241	upstream:Ca_15045;	TTTTCTTCTCGGTAAAGATCC	TCCTCAAGTCTCTATTTATGAGC
Ca_GpSSR00176	scaffold01134	Dinucleotide	(AT)10	20	87553	87572	Ca1	Dinucleotide	(AT)9	18	47730181	47730198	Intergenic	AAGGGACGTTTACATAATTT	ACTTGACATTGCAAGGATATT
Ca_GpSSR00177	scaffold01134	Dinucleotide	(AT)11	22	89116	89137	Ca1	Dinucleotide	(AT)13	26	47731733	47731758	Intergenic	ATTTGTAGATGGTTGATGGTG	TTTCAGAAAATGACGATTAGG
Ca_GpSSR00178	Ca_LG_1	Dinucleotide	(AT)13	26	9284793	9284818	Ca1	Dinucleotide	(AT)12	24	47960007	47960030	Intergenic	ATCGCGTTAAATACTTGGACT	CATATGTGGAATGGACTATGC
Ca_GpSSR00179	scaffold07450	Trinucleotide	(TTA)7	21	638	658	Ca1	Trinucleotide	(TTA)5	15	48092389	48092403	Intergenic		
Ca_GpSSR00180	scaffold102782	Dinucleotide	(TA)16	32	48	79	Ca1	Dinucleotide	(TA)8	16	48206333	48206348	Intergenic	ATCGCGTTAAATACTTGGACT	CATATGTGGAATGGACTATGC
Ca_GpSSR00181	scaffold00368	Dinucleotide	(TA)10	20	77513	77532	Ca2	Dinucleotide	(TA)11	22	371547	371568	Intergenic	GATTTAATGGAGGGAAGATGT	CTTTTCCATTTTGTGAGGATT
Ca_GpSSR00182	scaffold00368	Dinucleotide	(TA)9	18	112025	112042	Ca2	Dinucleotide	(TA)10	20	408896	408915	Intergenic	GATTTAATGGAGGGAAGATGT	CTTTTCCATTTTGTGAGGATT
Ca_GpSSR00183	scaffold00368	Trinucleotide	(TAA)42	126	132481	132606	Ca2	Trinucleotide	(TAA)30	90	434661	434750	upstream:Ca_12327;		
Ca_GpSSR00184	scaffold03705	Dinucleotide	(AT)12	24	283	306	Ca2	Dinucleotide	(AT)11	22	1034777	1034798	Intergenic	TGACTAAAACGTTAAATGCAG	CCGTTAAACCGTATGACATT
Ca_GpSSR00185	Ca_LG_6	Dinucleotide	(AT)11	22	4288001	4288022	Ca2	Dinucleotide	(AT)10	20	1657272	1657291	upstream:Ca_06777;	TTTCTTCCTCATTTCAACAA	GAGTGACGTATTTTCTCAACTG
Ca_GpSSR00186	Ca_LG_6	Dinucleotide	(TA)11	22	3392951	3392972	Ca2	Dinucleotide	(TA)13	26	1835748	1835773	mRNA=Ca_06682.1;gene=Ca_06682;intron;	TTTCTTCCTCATTTCAACAA	GAGTGACGTATTTTCTCAACTG
Ca_GpSSR00187	Ca_LG_6	Dinucleotide	(TA)9	18	3580618	3580635	Ca2	Dinucleotide	(TA)10	20	2029936	2029955	Intergenic	TTTCTTCCTCATTTCAACAA	GAGTGACGTATTTTCTCAACTG
Ca_GpSSR00188	scaffold01150	Dinucleotide	(AT)11	22	1233	1254	Ca2	Dinucleotide	(AT)9	18	2433180	2433197	upstream:Ca_15086;	TGAAGCAATCTGATCTCTCAC	TGTTCTAAATCTCCAATGTCA
Ca_GpSSR00189	scaffold00558	Dinucleotide	(TC)9	18	38258	38275	Ca2	Dinucleotide	(TC)7	14	3033691	3033704	Intergenic	ACAAATTGAGAAGGGTCAAAT	TGAAGAAAAAGAGAGACATAGGA
Ca_GpSSR00190	scaffold00558	Dinucleotide	(AT)6	12	58677	58688	Ca2	Dinucleotide	(AT)7	14	3050932	3050945	upstream:Ca_13191;	TTGATTCCAATACAAATCACC	AAAGTGATTTTGCTGTTGTTC
Ca_GpSSR00191	scaffold00078	Dinucleotide	(TA)20	40	92569	92608	Ca2	Dinucleotide	(TA)25	50	3282129	3282178	Intergenic	ACAATTCCACTTAATCTTTGC	AATTAGCCTACAGACACACACA
Ca_GpSSR00192	scaffold11684	Dinucleotide	(AT)18	36	2	37	Ca2	Dinucleotide	(AT)15	30	3329817	3329846	Intergenic	TTTCTTCCTCATTTCAACAA	GAGTGACGTATTTTCTCAACTG
Ca_GpSSR00193	scaffold00078	Dinucleotide	(AT)11	22	337855	337876	Ca2	Dinucleotide	(AT)16	32	3531882	3531913	Intergenic	TTTCTTCCTCATTTCAACAA	GAGTGACGTATTTTCTCAACTG
Ca_GpSSR00194	Ca_LG_2	Dinucleotide	(AT)13	26	1974359	1974384	Ca2	Dinucleotide	(AT)10	20	4014196	4014215	Intergenic	TTTCTTCCTCATTTCAACAA	GAGTGACGTATTTTCTCAACTG
Ca_GpSSR00195	Ca_LG_2	Dinucleotide	(AT)19	38	1975086	1975123	Ca2	Dinucleotide	(AT)28	56	4014917	4014972	Intergenic	ACAATTCCACTTAATCTTTGC	AATTAGCCTACAGACACACACA
Ca_GpSSR00196	scaffold42178	Dinucleotide	(TA)16	32	692	723	Ca2	Dinucleotide	(TA)14	28	4023005	4023032	Intergenic	CGGTGGCTGTATTTTATTTTA	TGTAACGATTCAATTTCTACCA
Ca_GpSSR00197	scaffold01455	Dinucleotide	(AT)12	24	53170	53193	Ca2	Dinucleotide	(AT)11	22	4539195	4539216	Intergenic	TGCAAGACATTATCTAAAGTGG	ATTCTGTGTAAAGATCCATGC
Ca_GpSSR00198	scaffold12945	Dinucleotide	(AT)10	20	1304	1323	Ca2	Dinucleotide	(AT)8	16	4905078	4905093	Intergenic	TTTCTTCCTCATTTCAACAA	GAGTGACGTATTTTCTCAACTG
Ca_GpSSR00199	scaffold02025	Dinucleotide	(AT)15	30	38351	38380	Ca2	Dinucleotide	(AT)8	16	4978808	4978823	Intergenic	TTTCTTCCTCATTTCAACAA	GAGTGACGTATTTTCTCAACTG
Ca_GpSSR00200	scaffold00160	Dinucleotide	(AT)15	30	61726	61755	Ca2	Dinucleotide	(AT)12	24	5096453	5096476	Intergenic	TTTCTTCCTCATTTCAACAA	GAGTGACGTATTTTCTCAACTG
Ca_GpSSR00201	scaffold00160	Dinucleotide	(AT)21	42	150353	150394	Ca2	Dinucleotide	(AT)22	44	5190267	5190310	Intergenic		
Ca_GpSSR00202	scaffold10498	Dinucleotide	(AT)19	38	1	38	Ca2	Dinucleotide	(AT)7	14	5228837	5228850	Intergenic	TTTCTTCCTCATTTCAACAA	GAGTGACGTATTTTCTCAACTG
Ca_GpSSR00203	scaffold09389	Trinucleotide	(ATA)34	102	1335	1436	Ca2	Trinucleotide	(ATA)19	57	6035592	6035648	Intergenic		
Ca_GpSSR00204	scaffold91323	Dinucleotide	(AT)19	38	375	412	Ca2	Dinucleotide	(AT)17	34	6717838	6717871	Intergenic	TTTCTTCCTCATTTCAACAA	GAGTGACGTATTTTCTCAACTG
Ca_GpSSR00205	Ca_LG_2	Dinucleotide	(AT)25	50	2329773	2329822	Ca2	Dinucleotide	(AT)20	40	7011189	7011228	Intergenic		
Ca_GpSSR00206	scaffold00922	Dinucleotide	(AT)14	28	1655	1682	Ca2	Dinucleotide	(AT)12	24	7043444	7043467	Intergenic	TTTCTTCCTCATTTCAACAA	GAGTGACGTATTTTCTCAACTG
Ca_GpSSR00207	scaffold04360	Dinucleotide	(AT)8	16	11902	11917	Ca2	Dinucleotide	(AT)10	20	7056679	7056698	Intergenic	TATCAAGTTTAGGGTTTAGGG	CATCTAGAAGCTCAAAACCAA
Ca_GpSSR00208	scaffold04360	Dinucleotide	(TA)25	50	13020	13069	Ca2	Dinucleotide	(TA)6	12	7056841	7056852	Intergenic	TATCAAGTTTAGGGTTTAGGG	CATCTAGAAGCTCAAAACCAA
Ca_GpSSR00209	scaffold00541	Trinucleotide	(TTA)35	105	67182	67286	Ca2	Trinucleotide	(TTA)20	60	7620184	7620243	Intergenic	CCTTGCGTGAGTTTGACGTA	CATCGCGGTCATGTGaGTTA
Ca_GpSSR00210	Ca_LG_7	Trinucleotide	(TTA)20	60	1527838	1527897	Ca2	Trinucleotide	(TTA)21	63	7665876	7665938	Intergenic	TGATGTTCTCTGTCTTTCCTT	TCCATATGCAAGAGTAAAAGC
Ca_GpSSR00211	scaffold03135	Dinucleotide	(TA)10	20	23266	23285	Ca2	Dinucleotide	(TA)11	22	8159119	8159140	upstream:Ca_18165;	ATTTAACCTGCATATCAATCG	CCACACCTGCATTTACTCA
Ca_GpSSR00212	scaffold07987	Dinucleotide	(AT)12	24	2933	2956	Ca2	Dinucleotide	(AT)25	50	8175530	8175579	Intergenic	ACAATTCCACTTAATCTTTGC	AATTAGCCTACAGACACACACA
Ca_GpSSR00213	scaffold00166	Dinucleotide	(TA)7	14	48977	48990	Ca2	Dinucleotide	(TA)14	28	8239573	8239600	Intergenic	TTTTAACATCCAGAaGTAAAAGAAAa	ATcCCATAcACCgaCGACAT
Ca_GpSSR00214	Ca_LG_2	Dinucleotide	(TA)12	24	2711964	2711987	Ca2	Dinucleotide	(TA)11	22	8645959	8645980	Intergenic	TTTCTTCCTCATTTCAACAA	GAGTGACGTATTTTCTCAACTG
Ca_GpSSR00215	scaffold49661	Dinucleotide	(AT)7	14	22	35	Ca2	Dinucleotide	(AT)9	18	8647049	8647066	Intergenic	TTTCTTCCTCATTTCAACAA	GAGTGACGTATTTTCTCAACTG
Ca_GpSSR00216	scaffold06523	Dinucleotide	(AT)14	28	2560	2587	Ca2	Dinucleotide	(AT)11	22	9215801	9215822	Intergenic	CAAATATGAGATTCCATTCA	CGACTTCTCTTTTTCTGTTCA
Ca_GpSSR00217	scaffold00846	Trinucleotide	(AAT)15	45	26456	26500	Ca2	Trinucleotide	(AAT)11	33	9298700	9298732	mRNA=Ca_14257.1;gene=Ca_14257;intron;	TTGGTAGGAAGGTTGATCTGTTC	TGGCCCTAAATATTAGTATCATTTCAA
Ca_GpSSR00218	scaffold00846	Trinucleotide	(TAT)49	147	62065	62211	Ca2	Trinucleotide	(TAT)11	33	9329385	9329417	mRNA=Ca_14257.1;gene=Ca_14257;intron;		
Ca_GpSSR00219	scaffold00846	Dinucleotide	(AT)12	24	64932	64955	Ca2	Dinucleotide	(AT)14	28	9332068	9332095	mRNA=Ca_14257.1;gene=Ca_14257;intron;	TTTCTTCCTCATTTCAACAA	GAGTGACGTATTTTCTCAACTG
Ca_GpSSR00220	scaffold00846	Dinucleotide	(TA)21	42	101652	101693	Ca2	Dinucleotide	(TA)9	18	9370721	9370738	Intergenic	TTTCTTCCTCATTTCAACAA	GAGTGACGTATTTTCTCAACTG
Ca_GpSSR00221	scaffold00846	Dinucleotide	(AT)25	50	104003	104052	Ca2	Dinucleotide	(AT)16	32	9372804	9372835	Intergenic	TTTCTTCCTCATTTCAACAA	GAGTGACGTATTTTCTCAACTG
Ca_GpSSR00222	scaffold01854	Dinucleotide	(TA)11	22	50777	50798	Ca2	Dinucleotide	(TA)10	20	9520264	9520283	Intergenic	GCATCAAAACATCCCATAATA	AAGTCTTGACCTGAACCTTAAA
Ca_GpSSR00223	scaffold00719	Dinucleotide	(TA)11	22	43946	43967	Ca2	Dinucleotide	(TA)12	24	9689511	9689534	Intergenic	TTTCTTCCTCATTTCAACAA	GAGTGACGTATTTTCTCAACTG
Ca_GpSSR00224	Ca_LG_2	Dinucleotide	(TA)12	24	2464854	2464877	Ca2	Dinucleotide	(TA)9	18	9921390	9921407	Intergenic	TTTCTTCCTCATTTCAACAA	GAGTGACGTATTTTCTCAACTG
Ca_GpSSR00225	Ca_LG_2	Trinucleotide	(AAT)13	39	2515418	2515456	Ca2	Trinucleotide	(AAT)18	54	9970651	9970704	Intergenic		
Ca_GpSSR00226	Ca_LG_2	Dinucleotide	(AT)8	16	2538960	2538975	Ca2	Dinucleotide	(AT)9	18	9998184	9998201	Intergenic	ATTTGCACTCTTTGACTTTGA	TCAAATGTTTAACTCGTGGAC
Ca_GpSSR00227	Ca_LG_2	Dinucleotide	(TA)10	20	2550179	2550198	Ca2	Dinucleotide	(TA)7	14	10013936	10013949	Intergenic	ATTTGCACTCTTTGACTTTGA	TCAAATGTTTAACTCGTGGAC
Ca_GpSSR00228	Ca_LG_2	Dinucleotide	(AT)6	12	2573258	2573269	Ca2	Dinucleotide	(AT)7	14	10032702	10032715	upstream:Ca_01220;	ATTTGCACTCTTTGACTTTGA	TCAAATGTTTAACTCGTGGAC
Ca_GpSSR00229	Ca_LG_2	Dinucleotide	(TA)9	18	2626750	2626767	Ca2	Dinucleotide	(TA)22	44	10086460	10086503	Intergenic	ATTTGCACTCTTTGACTTTGA	TCAAATGTTTAACTCGTGGAC
Ca_GpSSR00230	Ca_LG_2	Dinucleotide	(AG)9	18	5381402	5381419	Ca2	Dinucleotide	(AG)10	20	10207705	10207724	mRNA=Ca_01400.1;gene=Ca_01400;intron;	CAAATTTGTTAGAGGAAACCA	CACCACCTCTTTCTATTTCAA
Ca_GpSSR00231	Ca_LG_2	Dinucleotide	(AT)7	14	5419221	5419234	Ca2	Dinucleotide	(AT)11	22	10240969	10240990	Intergenic	ATCTGGGGCCAAAAaGTAGG	CATTCAGCGACAAAAGGTCA
Ca_GpSSR00232	scaffold05933	Dinucleotide	(TA)12	24	5995	6018	Ca2	Dinucleotide	(TA)15	30	11044127	11044156	Intergenic	CAAAAGACATTTTCATCCTTTAG	AAAGCTAGGCTCAGACTTTTT
Ca_GpSSR00233	scaffold05933	Dinucleotide	(AT)15	30	6311	6340	Ca2	Dinucleotide	(AT)17	34	11044449	11044482	Intergenic	CAAAAGACATTTTCATCCTTTAG	AAAGCTAGGCTCAGACTTTTT
Ca_GpSSR00234	Ca_LG_2	Dinucleotide	(CT)6	12	5042612	5042623	Ca2	Dinucleotide	(CT)7	14	11718138	11718151	Intergenic	GTAAACGGGATTTTGACTCTT	GGTGGATTAGAGAGGCTAGTG
Ca_GpSSR00235	scaffold01447	Dinucleotide	(TA)8	16	35780	35795	Ca2	Dinucleotide	(TA)24	48	12003370	12003417	Intergenic	ACAATTCCACTTAATCTTTGC	AATTAGCCTACAGACACACACA
Ca_GpSSR00236	scaffold01166	Dinucleotide	(AT)14	28	63860	63887	Ca2	Dinucleotide	(AT)16	32	12558022	12558053	Intergenic	TTTCTTCCTCATTTCAACAA	GAGTGACGTATTTTCTCAACTG
Ca_GpSSR00237	scaffold01654	Dinucleotide	(AT)10	20	39914	39933	Ca2	Dinucleotide	(AT)9	18	13757791	13757808	upstream:Ca_16229;	ACAGAATTGTTCTTGTGAGACA	TTTCTCGTATGGGTAGTTTTG
Ca_GpSSR00238	scaffold00879	Dinucleotide	(AT)9	18	37908	37925	Ca2	Dinucleotide	(AT)10	20	14118072	14118091	Intergenic	GAAGGTCCGATAAGAGAAATC	TGGTGGTTACAATGTTTCTTC
Ca_GpSSR00239	scaffold05168	Dinucleotide	(AT)21	42	8566	8607	Ca2	Dinucleotide	(AT)19	38	14204210	14204247	Intergenic		
Ca_GpSSR00240	scaffold01244	Dinucleotide	(TA)16	32	71818	71849	Ca2	Dinucleotide	(TA)15	30	14246698	14246727	Intergenic	TCGTCTTTCTGAAACTTACAA	TCCAAAAGATGTAAACTAGAGTG

Ca_GpSSR00241	scaffold17215	Dinucleotide	(TA)20	40	21	60	Ca2	Dinucleotide	(TA)9	18	14283177	14283194	Intergenic	ACCCAATTTGGTTTCACTAA	TTGGACTTGAAAGCTTATTTC
Ca_GpSSR00242	scaffold01828	Dinucleotide	(TA)19	38	25978	26015	Ca2	Dinucleotide	(TA)22	44	14347637	14347680	Intergenic		
Ca_GpSSR00243	scaffold03204	Dinucleotide	(TA)9	18	120496	120513	Ca2	Dinucleotide	(TA)6	12	16293789	16293800	Intergenic	GAAATCTTGCCCAGTTAAAAT	AAAGTGTGGTTCATATCTCACAT
Ca_GpSSR00244	scaffold02129	Dinucleotide	(TA)10	20	48887	48906	Ca2	Dinucleotide	(TA)12	24	17913767	17913790	Intergenic	GTTTCAAAGCGATATCAAAATAG	CATGCAAGATGAGAAGCTAAT
Ca_GpSSR00245	scaffold01924	Dinucleotide	(CT)8	16	212764	212779	Ca2	Dinucleotide	(CT)9	18	18353412	18353429	upstream:Ca_09546		
Ca_GpSSR00246	scaffold04543	Dinucleotide	(AT)8	16	3359	3374	Ca2	Dinucleotide	(AT)9	18	18391803	18391820	Intergenic	TTTCTTCCTCATTTCAACAA	GAGTGACGTATTTTCTCAACTG
Ca_GpSSR00247	scaffold04790	Dinucleotide	(TA)24	48	12063	12110	Ca2	Dinucleotide	(TA)8	16	18676129	18676144	Intergenic	TTTCTTCCTCATTTCAACAA	GAGTGACGTATTTTCTCAACTG
Ca_GpSSR00248	Ca_LG_4	Dinucleotide	(AT)11	22	18542354	18542375	Ca2	Dinucleotide	(AT)10	20	18932387	18932406	exon=Ca_05024.1.exon1;gene=Ca_05024;5'-UTR=Ca_	TTTCTTCCTCATTTCAACAA	GAGTGACGTATTTTCTCAACTG
Ca_GpSSR00249	scaffold04261	Trinucleotide	(ATT)14	42	15733	15774	Ca2	Trinucleotide	(ATT)13	39	19196929	19196967	Intergenic	TCATCATCACCACCTTTTACT	GAATTCGCCATATAAACTGAA
Ca_GpSSR00250	scaffold00446	Dinucleotide	(AT)11	22	280881	280902	Ca2	Dinucleotide	(AT)10	20	20348650	20348669	upstream:Ca_09247;	CTCTGCGTTGTTTATTATAAGG	TGGTGAGTGGAAAAGTTAAAA
Ca_GpSSR00251	scaffold01399	Dinucleotide	(GA)8	16	44139	44154	Ca2	Dinucleotide	(GA)7	14	20897412	20897425	upstream:Ca_15714	TTGCCTAGTTGGCAGTAGTAG	ACTCTCAATTTGGATTCCATT
Ca_GpSSR00252	scaffold40371	Dinucleotide	(TA)8	16	659	674	Ca2	Dinucleotide	(TA)9	18	21266267	21266284	Intergenic	CTTGAGATAGTGGTAATATTTTG	TCTTTCTAATACCCACATTGAA
Ca_GpSSR00253	scaffold01219	Dinucleotide	(TA)16	32	81059	81090	Ca2	Dinucleotide	(TA)24	48	21641487	21641534	Intergenic	ACAATTCCACTTAATCTTTGC	AATTAGCCTACAGACACACACA
Ca_GpSSR00254	scaffold08601	Dinucleotide	(TA)22	44	6265	6308	Ca2	Dinucleotide	(TA)17	34	21735445	21735478	Intergenic	TTTCTTCCTCATTTCAACAA	GAGTGACGTATTTTCTCAACTG
Ca_GpSSR00255	scaffold01831	Dinucleotide	(AT)11	22	57628	57649	Ca2	Dinucleotide	(AT)12	24	22243313	22243336	Intergenic	TTTCTTCCTCATTTCAACAA	GAGTGACGTATTTTCTCAACTG
Ca_GpSSR00256	scaffold08095	Dinucleotide	(TA)11	22	3405	3426	Ca2	Dinucleotide	(TA)12	24	22337783	22337806	Intergenic	AGAGTCAACGAATCTCCACTT	TTTGGTCTTAAGATTGTTGGA
Ca_GpSSR00257	scaffold04802	Trinucleotide	(ATA)27	81	42917	42997	Ca2	Trinucleotide	(ATA)12	36	23011623	23011658	Intergenic		
Ca_GpSSR00258	scaffold04802	Dinucleotide	(TC)17	34	54068	54101	Ca2	Dinucleotide	(TC)18	36	23025138	23025173	mRNA=Ca_10445.1;gene=Ca_10445;intron;	TGCATCAAAGAAGGAAAGTTA	GGAAAGGAAGAAGAAAGAGAA
Ca_GpSSR00259	scaffold01786	Trinucleotide	(AAT)29	87	44809	44895	Ca2	Trinucleotide	(AAT)16	48	23668879	23668926	Intergenic		
Ca_GpSSR00260	scaffold02601	Dinucleotide	(AT)16	32	37240	37271	Ca2	Dinucleotide	(AT)15	30	23849488	23849517	Intergenic	AACCAATTTCTCACAACCATA	CTCGCCTTAATTATTTGTTT
Ca_GpSSR00261	scaffold00654	Dinucleotide	(TA)12	24	151014	151037	Ca2	Dinucleotide	(TA)11	22	23993905	23993926	Intergenic	ACGAATTTTCGTTAGTCTTT	GGGCCATAATTGAGATTTAAG
Ca_GpSSR00262	scaffold00654	Trinucleotide	(ATA)30	90	115861	115950	Ca2	Trinucleotide	(ATA)20	60	24135382	24135441	Intergenic		
Ca_GpSSR00263	scaffold02154	Dinucleotide	(AT)9	18	30042	30059	Ca2	Dinucleotide	(AT)10	20	25078729	25078748	Intergenic	CGCACTCTTCAGGTATTGTAT	TTTTTGTTTCGACACTGTAGC
Ca_GpSSR00264	Ca_LG_6	Dinucleotide	(TA)8	16	9506258	9506273	Ca2	Dinucleotide	(TA)9	18	25492803	25492820	Intergenic	TTTCTTCCTCATTTCAACAA	GAGTGACGTATTTTCTCAACTG
Ca_GpSSR00265	scaffold04397	Dinucleotide	(TA)12	24	1071	1094	Ca2	Dinucleotide	(TA)13	26	25593756	25593781	Intergenic	TTTCTTCCTCATTTCAACAA	GAGTGACGTATTTTCTCAACTG
Ca_GpSSR00266	scaffold00520	Dinucleotide	(TA)21	42	145447	145488	Ca2	Dinucleotide	(TA)26	52	25918163	25918214	Intergenic	ACAATTCCACTTAATCTTTGC	AATTAGCCTACAGACACACACA
Ca_GpSSR00267	scaffold17444	Dinucleotide	(AT)11	22	1413	1434	Ca2	Dinucleotide	(AT)10	20	26109347	26109366	Intergenic	AGTAGCTTTCAGTTCAACAGT	AAGAATTGTGATATGGGTAAGC
Ca_GpSSR00268	scaffold00297	Dinucleotide	(AT)19	38	18188	18225	Ca2	Dinucleotide	(AT)12	24	26470801	26470824	Intergenic	TTTCTTCCTCATTTCAACAA	GAGTGACGTATTTTCTCAACTG
Ca_GpSSR00269	scaffold03060	Dinucleotide	(AT)13	26	30476	30501	Ca2	Dinucleotide	(AT)12	24	26707706	26707729	Intergenic	ATATCACCACCATTAATGACA	AATTTTAGCGTAGTGTGTGGA
Ca_GpSSR00270	scaffold03060	Dinucleotide	(AT)12	24	30450	30473	Ca2	Dinucleotide	(AT)11	22	26707732	26707753	Intergenic	ATATCACCACCATTAATGACA	AATTTTAGCGTAGTGTGTGGA
Ca_GpSSR00271	scaffold00274	Dinucleotide	(AT)20	40	149602	149641	Ca2	Dinucleotide	(AT)16	32	27729660	27729691	Intergenic	TTTCTTCCTCATTTCAACAA	GAGTGACGTATTTTCTCAACTG
Ca_GpSSR00272	Ca_LG_2	Dinucleotide	(AT)13	26	3515543	3515568	Ca2	Dinucleotide	(AT)17	34	28157708	28157741	upstream:Ca_01286;	TcATTTTTGAATGTCCATGCTT	TCAATTTCTTAtACTTTTCGCAACA
Ca_GpSSR00273	scaffold23533	Dinucleotide	(TA)7	14	1	14	Ca2	Dinucleotide	(TA)22	44	28247278	28247321	Intergenic		
Ca_GpSSR00274	scaffold18923	Dinucleotide	(TA)11	22	1680	1701	Ca2	Dinucleotide	(TA)12	24	28846312	28846335	Intergenic	TTCTTTTTCTTTCCTGTCTTTC	GGAATATTGGCAGATCAACTA
Ca_GpSSR00275	scaffold58980	Dinucleotide	(AT)19	38	576	613	Ca2	Dinucleotide	(AT)7	14	29172414	29172427	Intergenic	TTTCTTCCTCATTTCAACAA	GAGTGACGTATTTTCTCAACTG
Ca_GpSSR00276	scaffold01604	Dinucleotide	(AT)13	26	41468	41493	Ca2	Dinucleotide	(AT)22	44	29315904	29315947	upstream:Ca_16130;		
Ca_GpSSR00277	scaffold22823	Dinucleotide	(AT)15	30	1406	1435	Ca2	Dinucleotide	(AT)18	36	29878213	29878248	Intergenic	TTTCTTCCTCATTTCAACAA	GAGTGACGTATTTTCTCAACTG
Ca_GpSSR00278	scaffold01369	Dinucleotide	(TA)18	36	1	36	Ca2	Dinucleotide	(TA)6	12	29878998	29879009	Intergenic	TTTCTTCCTCATTTCAACAA	GAGTGACGTATTTTCTCAACTG
Ca_GpSSR00279	scaffold01691	Dinucleotide	(AT)9	18	38836	38853	Ca2	Dinucleotide	(AT)12	24	30187524	30187547	Intergenic	CGAGTGAAATTGAAAATGAAC	CCTTGAAATTAATAGATCTCCAC
Ca_GpSSR00280	Ca_LG_2	Dinucleotide	(TA)15	30	3679983	3680012	Ca2	Dinucleotide	(TA)12	24	30291446	30291469	Intergenic	TTTCTTCCTCATTTCAACAA	GAGTGACGTATTTTCTCAACTG
Ca_GpSSR00281	Ca_LG_2	Dinucleotide	(AT)10	20	3785926	3785945	Ca2	Dinucleotide	(AT)9	18	30399985	30400002	Intergenic	ATTTGCACTCTTTGACTTTGA	TCAAATGTTTAACTCGTGGAC
Ca_GpSSR00282	Ca_LG_2	Dinucleotide	(AT)10	20	3816577	3816596	Ca2	Dinucleotide	(AT)11	22	30431127	30431148	Intergenic	ATTTGCACTCTTTGACTTTGA	TCAAATGTTTAACTCGTGGAC
Ca_GpSSR00283	scaffold02575	Dinucleotide	(AT)15	30	32460	32489	Ca2	Dinucleotide	(AT)21	42	30775309	30775350	Intergenic	GTTAAAATCTTTTACTGGCTTTG	AGAGAGAGAGAGAAGAACAACG
Ca_GpSSR00284	scaffold02476	Trinucleotide	(AAT)10	30	10536	10565	Ca2	Trinucleotide	(AAT)6	18	30847526	30847543	upstream:Ca_17495;	TTGTGTGGAGCTTAGGGTGTC	CCAATTAGTATATTGCTTCACAGACAA
Ca_GpSSR00285	scaffold02476	Dinucleotide	(TA)8	16	12599	12614	Ca2	Dinucleotide	(TA)17	34	30849550	30849583	Intergenic	GACCGAAATTTTAACCCTTGG	CGGACGGCATATCTAAGTTGA
Ca_GpSSR00286	scaffold00225	Trinucleotide	(AAT)19	57	202872	202928	Ca2	Trinucleotide	(AAT)29	87	30988922	30989008	Intergenic	TTGAAGATATATACATGAGCAAC	GGAGCTTTCTTTGATTTTTCT
Ca_GpSSR00287	scaffold01850	Dinucleotide	(AT)12	24	1205	1228	Ca2	Dinucleotide	(AT)13	26	31173307	31173332	Intergenic	CTTTTAGATAGGTGTAGCTTTGA	TCAAACCACATTCGTCTTTAT
Ca_GpSSR00288	scaffold01542	Trinucleotide	(TAT)12	36	51723	51758	Ca2	Trinucleotide	(TAT)34	102	31594661	31594762	upstream:Ca_16035;	TGATTCAACTAAAATAGGAGGA	TTCCAGCATTGACAATTACAT
Ca_GpSSR00289	scaffold85402	Trinucleotide	(ATT)6	18	241	258	Ca2	Trinucleotide	(ATT)7	21	31931259	31931279	Intergenic	AGTTAAGGGAATTTAGCCTTTT	CCATCTCCAATTTTGTTTTC
Ca_GpSSR00290	scaffold00420	Dinucleotide	(TA)11	22	83747	83768	Ca2	Dinucleotide	(TA)24	48	31986813	31986860	Intergenic	ACAATTCCACTTAATCTTTGC	AATTAGCCTACAGACACACACA
Ca_GpSSR00291	scaffold00420	Dinucleotide	(AT)8	16	43707	43722	Ca2	Dinucleotide	(AT)11	22	32032324	32032345	Intergenic	TTTCTTCCTCATTTCAACAA	GAGTGACGTATTTTCTCAACTG
Ca_GpSSR00292	scaffold41688	Dinucleotide	(TA)20	40	746	785	Ca2	Dinucleotide	(TA)14	28	32040141	32040168	Intergenic	TTTCTTCCTCATTTCAACAA	GAGTGACGTATTTTCTCAACTG
Ca_GpSSR00293	scaffold01901	Dinucleotide	(AT)7	14	35599	35612	Ca2	Dinucleotide	(AT)8	16	32241235	32241250	upstream:Ca_16632;	TTTCTTCCTCATTTCAACAA	GAGTGACGTATTTTCTCAACTG
Ca_GpSSR00294	scaffold02595	Dinucleotide	(TA)6	12	2245	2256	Ca2	Dinucleotide	(TA)7	14	32330521	32330534	Intergenic	ATAGGAAGCTCACAAACATTG	TTCTCATTCTCAAGCTGAATC
Ca_GpSSR00295	Ca_LG_2	Dinucleotide	(AT)6	12	6418959	6418970	Ca2	Dinucleotide	(AT)9	18	32334667	32334684	upstream:Ca_01491;	ATTTGCACTCTTTGACTTTGA	TCAAATGTTTAACTCGTGGAC
Ca_GpSSR00296	Ca_LG_2	Trinucleotide	(TTA)5	15	6529337	6529351	Ca2	Trinucleotide	(TTA)13	39	32478386	32478424	Intergenic	TGATGTTCTCTGTCTTTCCTT	TCCATATGCAAGAGTAAAAGC
Ca_GpSSR00297	Ca_LG_2	Dinucleotide	(TC)8	16	6872427	6872442	Ca2	Dinucleotide	(TC)9	18	32745344	32745361	gene=Ca_01523;mRNA=Ca_01523.2;intron;	TCTGGCAGATGAAGATGTAAC	TAACAGACCAAAAACTCCAAA
Ca_GpSSR00298	Ca_LG_2	Dinucleotide	(AT)18	36	6998435	6998470	Ca2	Dinucleotide	(AT)20	40	32873027	32873066	Intergenic		
Ca_GpSSR00299	Ca_LG_2	Dinucleotide	(AT)10	20	7163358	7163377	Ca2	Dinucleotide	(AT)9	18	33023523	33023540	Intergenic	ATTTGCACTCTTTGACTTTGA	TCAAATGTTTAACTCGTGGAC
Ca_GpSSR00300	Ca_LG_2	Dinucleotide	(AT)14	28	7170756	7170783	Ca2	Dinucleotide	(AT)18	36	33035031	33035066	Intergenic	TTTCTTCCTCATTTCAACAA	GAGTGACGTATTTTCTCAACTG
Ca_GpSSR00301	scaffold21244	Dinucleotide	(AT)9	18	1064	1081	Ca2	Dinucleotide	(AT)16	32	33171840	33171871	Intergenic	TTTCTTCCTCATTTCAACAA	GAGTGACGTATTTTCTCAACTG
Ca_GpSSR00302	Ca_LG_2	Trinucleotide	(AAT)43	129	7502281	7502409	Ca2	Trinucleotide	(AAT)32	96	33830163	33830258	Intergenic		
Ca_GpSSR00303	Ca_LG_2	Dinucleotide	(AT)16	32	7642719	7642750	Ca2	Dinucleotide	(AT)14	28	34235262	34235289	upstream:Ca_01603;	TTTCTTCCTCATTTCAACAA	GAGTGACGTATTTTCTCAACTG
Ca_GpSSR00304	scaffold02749	Dinucleotide	(TA)13	26	17152	17177	Ca2	Dinucleotide	(TA)12	24	34329918	34329941	Intergenic	ATATTTGCCCCTTAAGCTAGT	CACAAGACACAAAACAAAAGG
Ca_GpSSR00305	scaffold04168	Dinucleotide	(TA)13	26	5885	5910	Ca2	Dinucleotide	(TA)27	54	34464853	34464906	Intergenic	ACAATTCCACTTAATCTTTGC	AATTAGCCTACAGACACACACA
Ca_GpSSR00306	scaffold08771	Dinucleotide	(TA)16	32	7	38	Ca2	Dinucleotide	(TA)15	30	35031077	35031106	Intergenic	TTTCTTCCTCATTTCAACAA	GAGTGACGTATTTTCTCAACTG
Ca_GpSSR00307	scaffold08771	Dinucleotide	(AT)15	30	6108	6137	Ca2	Dinucleotide	(AT)7	14	35037307	35037320	Intergenic	TTTCTTCCTCATTTCAACAA	GAGTGACGTATTTTCTCAACTG
Ca_GpSSR00308	scaffold01478	Dinucleotide	(AT)8	16	72569	72584	Ca2	Dinucleotide	(AT)9	18	35073836	35073853	upstream:Ca_15889;	TTTCTTCCTCATTTCAACAA	GAGTGACGTATTTTCTCAACTG
Ca_GpSSR00309	Ca_LG_6	Dinucleotide	(TA)13	26	777404	777429	Ca2	Dinucleotide	(TA)20	40	35113441	35113480	Intergenic		
Ca_GpSSR00310	Ca_LG_6	Dinucleotide	(TA)9	18	820927	820944	Ca2	Dinucleotide	(TA)10	20	35158756	35158775	upstream:Ca_06502;	TTTCTTCCTCATTTCAACAA	GAGTGACGTATTTTCTCAACTG
Ca_GpSSR00311	Ca_LG_6	Trinucleotide	(ATA)27	81	822443	822523	Ca2	Trinucleotide	(ATA)8	24	35160276	35160299	Intergenic		
Ca_GpSSR00312	Ca_LG_6	Dinucleotide	(TA)10	20	843707	843726	Ca2	Dinucleotide	(TA)9	18	35180723	35180740	Intergenic	TTTCTTCCTCATTTCAACAA	GAGTGACGTATTTTCTCAACTG
Ca_GpSSR00313	Ca_LG_6	Trinucleotide	(AAT)6	18	865171	865188	Ca2	Trinucleotide	(AAT)19	57	35202765	35202821	Intergenic		
Ca_GpSSR00314	Ca_LG_6	Dinucleotide	(AT)17	34	744248	744281	Ca2	Dinucleotide	(AT)21	42	35332737	35332778	Intergenic		
Ca_GpSSR00315	Ca_LG_4	Trinucleotide	(AAT)8	24	3990821	3990844	Ca2	Trinucleotide	(AAT)5	15	35722047	35722061	upstream:Ca_04162;		
Ca_GpSSR00316	scaffold10005	Dinucleotide	(TA)16	32	2405	2436	Ca2	Dinucleotide	(TA)8	16	35816147	35816162	Intergenic	GCAAAGTGGAATTTGTGGGT	aAAACATCAATTTACCATCATTCA
Ca_GpSSR00317	Ca_LG_2	Dinucleotide	(TA)7	14	16657831	16657844	Ca2	Dinucleotide	(TA)8	16	35955087	35955102	Intergenic	ATTTGCACTCTTTGACTTTGA	TCAAATGTTTAACTCGTGGAC
Ca_GpSSR00318	Ca_LG_2	Trinucleotide	(GTT)5	15	16696532	16696546	Ca2	Trinucleotide	(GTT)7	21	35998700	35998720	mRNA=Ca_02120.1;exon=Ca_02120.1.exon2;CDS=Ca_	TGGCCCAATATTCAGATTCC	TCACCAAACCAGGTACTGCTC
Ca_GpSSR00319	Ca_LG_2	Trinucleotide	(ATT)7	21	16717405	16717425	Ca2	Trinucleotide	(ATT)8	24	36019004	36019027	Intergenic	TGATGTTCTCTGTCTTTCCTT	TCCATATGCAAGAGTAAAAGC
Ca_GpSSR00320	scaffold33774	Dinucleotide	(AT)12	24	977	1000	Ca2	Dinucleotide	(AT)19	38	36133422	36133459	Intergenic		
Ca_GpSSR00321	scaffold126914	Dinucleotide	(AT)7	14	285	298	Ca3	Dinucleotide	(AT)23	46	41407	41452	Intergenic	CAAATATGCAATAACCTTCCA	AAAAATCTATTTGGTGCTGGT

Ca_GpSSR00322	scaffold02122	Dinucleotide	(TA)7	14	9700	9713	Ca3	Dinucleotide	(TA)22	44	1264004	1264047	Intergenic	CAAATATGCAATAACCTTCCA	AAAAATCTATTTGGTGCTGGT
Ca_GpSSR00323	scaffold02501	Trinucleotide	(TAT)26	78	24880	24957	Ca3	Trinucleotide	(TAT)15	45	1386051	1386095	Intergenic		
Ca_GpSSR00324	scaffold01186	Dinucleotide	(TA)14	28	72962	72989	Ca3	Dinucleotide	(TA)12	24	1953128	1953151	Intergenic	GCATTTTGTTCTTTTCCTTCT	TCTTGCCTAAATAATGATCCA
Ca_GpSSR00325	scaffold02634	Trinucleotide	(TAT)12	36	39839	39874	Ca3	Trinucleotide	(TAT)11	33	2177060	2177092	Intergenic		
Ca_GpSSR00326	scaffold05856	Dinucleotide	(AT)23	46	10748	10793	Ca3	Dinucleotide	(AT)12	24	2444322	2444345	Intergenic	CAAATATGCAATAACCTTCCA	AAAAATCTATTTGGTGCTGGT
Ca_GpSSR00327	scaffold00321	Dinucleotide	(AT)9	18	88021	88038	Ca3	Dinucleotide	(AT)10	20	2886352	2886371	Intergenic	AAATTATCATGGTGACTCAA	ATGTGCTCCACAGTTTTTCTA
Ca_GpSSR00328	scaffold40121	Trinucleotide	(TTA)10	30	292	321	Ca3	Trinucleotide	(TTA)9	27	2962593	2962619	Intergenic	AATTAGAGGCAAACAAGAACC	ACACCTAATTTTGTCCGATTT
Ca_GpSSR00329	scaffold00321	Trinucleotide	(TTA)6	18	183106	183123	Ca3	Trinucleotide	(TTA)5	15	2985583	2985597	upstream:Ca_12030;	AAACTGATGTCATGGTCAAAG	TGTCTTCTTTCCACATCCTAA
Ca_GpSSR00330	Ca_LG_3	Dinucleotide	(AT)10	20	14009997	14010016	Ca3	Dinucleotide	(AT)15	30	3013495	3013524	Intergenic	AATCCACAAACACAACAAAAC	CCAATCAACTCATTGAGACC
Ca_GpSSR00331	scaffold03143	Dinucleotide	(TA)6	12	15908	15919	Ca3	Dinucleotide	(TA)16	32	3047940	3047971	Intergenic	TCCCTCAAGATAATATTTACCC	ATAGAGGTTGGGAATGTGTTT
Ca_GpSSR00332	Ca_LG_6	Dinucleotide	(TA)13	26	4302942	4302967	Ca3	Dinucleotide	(TA)16	32	3272994	3273025	upstream:Ca_06778;	CAAATATGCAATAACCTTCCA	AAAAATCTATTTGGTGCTGGT
Ca_GpSSR00333	scaffold03278	Dinucleotide	(TA)19	38	12681	12718	Ca3	Dinucleotide	(TA)18	36	3316147	3316182	mRNA=Ca_18276.1;gene=Ca_18276;intron;	CAAATATGCAATAACCTTCCA	AAAAATCTATTTGGTGCTGGT
Ca_GpSSR00334	scaffold03104	Dinucleotide	(AT)10	20	10290	10309	Ca3	Dinucleotide	(AT)24	48	3710819	3710866	Intergenic	AACAAGTGTCCGTTGAGAATA	TCCTCTGAGTTATGTCCTCCT
Ca_GpSSR00335	scaffold08235	Dinucleotide	(AT)8	16	938	953	Ca3	Dinucleotide	(AT)7	14	3838193	3838206	Intergenic	CAAATATGCAATAACCTTCCA	AAAAATCTATTTGGTGCTGGT
Ca_GpSSR00336	scaffold00617	Dinucleotide	(AT)8	16	82673	82688	Ca3	Dinucleotide	(AT)9	18	4180994	4181011	Intergenic	CAAATATGCAATAACCTTCCA	AAAAATCTATTTGGTGCTGGT
Ca_GpSSR00337	scaffold00617	Dinucleotide	(TA)14	28	166159	166186	Ca3	Dinucleotide	(TA)15	30	4332714	4332743	Intergenic	CAAATATGCAATAACCTTCCA	AAAAATCTATTTGGTGCTGGT
Ca_GpSSR00338	scaffold01392	Trinucleotide	(TAT)5	15	12016	12030	Ca3	Trinucleotide	(TAT)10	30	4561187	4561216	Intergenic		
Ca_GpSSR00339	scaffold01392	Trinucleotide	(AAT)13	39	47545	47583	Ca3	Trinucleotide	(AAT)19	57	4596676	4596732	upstream:Ca_15691;	GTGCACACGGGTTACACAGT	GTGCGGTGGTTGAAGGTAGT
Ca_GpSSR00340	scaffold01392	Trinucleotide	(TAT)12	36	75872	75907	Ca3	Trinucleotide	(TAT)14	42	4622795	4622836	Intergenic		
Ca_GpSSR00341	scaffold03057	Dinucleotide	(AT)14	28	25575	25602	Ca3	Dinucleotide	(AT)13	26	5239463	5239488	Intergenic	CAAATATGCAATAACCTTCCA	AAAAATCTATTTGGTGCTGGT
Ca_GpSSR00342	scaffold03057	Dinucleotide	(TA)9	18	29333	29350	Ca3	Dinucleotide	(TA)8	16	5243219	5243234	Intergenic	CAAATATGCAATAACCTTCCA	AAAAATCTATTTGGTGCTGGT
Ca_GpSSR00343	Ca_LG_3	Dinucleotide	(AT)23	46	14475055	14475100	Ca3	Dinucleotide	(AT)28	56	5431014	5431069	Intergenic	AATTTACCGCTGAAGAGTTTC	AGGGTAAACTTTAAACTCAAATG
Ca_GpSSR00344	scaffold19729	Dinucleotide	(TA)19	38	1672	1709	Ca3	Dinucleotide	(TA)17	34	5723135	5723168	Intergenic	CAAATATGCAATAACCTTCCA	AAAAATCTATTTGGTGCTGGT
Ca_GpSSR00345	scaffold00258	Dinucleotide	(AT)9	18	107332	107349	Ca3	Dinucleotide	(AT)10	20	6352792	6352811	Intergenic	GTGACACTCTAAACCCCAAA	TTTTGAGAAACGTGATATCCT
Ca_GpSSR00346	scaffold19952	Dinucleotide	(AT)12	24	1006	1029	Ca3	Dinucleotide	(AT)11	22	6375836	6375857	Intergenic	TTTTCCAACAATCTAAGCATC	TGTGATATAGTTTTGTACGCACT
Ca_GpSSR00347	scaffold23582	Dinucleotide	(TA)29	58	12	69	Ca3	Dinucleotide	(TA)22	44	6377157	6377200	Intergenic	CAAATATGCAATAACCTTCCA	AAAAATCTATTTGGTGCTGGT
Ca_GpSSR00348	scaffold05887	Tetranucleotide	(TTTA)5	20	8144	8163	Ca3	Tetranucleotide	(TTTA)7	28	6672423	6672450	gene=Ca_19508;mRNA=Ca_19508.1;intron;	CCGTCTCTGCTAAGATTGCC	AATGGGATTAATGGTGGTGAA
Ca_GpSSR00349	scaffold01393	Dinucleotide	(AT)12	24	52789	52812	Ca3	Dinucleotide	(AT)11	22	6814197	6814218	upstream:Ca_15697;	CAAATATGCAATAACCTTCCA	AAAAATCTATTTGGTGCTGGT
Ca_GpSSR00350	scaffold01393	Dinucleotide	(AT)8	16	52897	52912	Ca3	Dinucleotide	(AT)6	12	6814342	6814353	upstream:Ca_15697;	ATGTCCACCACTCAAAAATTA	TGAAAAGTAAAAAGCAACCA
Ca_GpSSR00351	scaffold03273	Dinucleotide	(TA)13	26	25982	26007	Ca3	Dinucleotide	(TA)12	24	6855598	6855621	Intergenic	GAGTCATTTGTGATTCGGTAG	TCCGTAAAAAGCACTTCAATA
Ca_GpSSR00352	scaffold60224	Trinucleotide	(ATT)14	42	510	551	Ca3	Trinucleotide	(ATT)11	33	6961515	6961547	Intergenic		
Ca_GpSSR00353	scaffold08839	Dinucleotide	(TA)6	12	1	12	Ca3	Dinucleotide	(TA)15	30	7409720	7409749	Intergenic	CAAATATGCAATAACCTTCCA	AAAAATCTATTTGGTGCTGGT
Ca_GpSSR00354	scaffold01039	Dinucleotide	(AT)11	22	104943	104964	Ca3	Dinucleotide	(AT)10	20	8560112	8560131	upstream:Ca_09953;	AAAATATGAGGTTTTGGTTCG	CGGTTATTTGATGAATTGTTG
Ca_GpSSR00355	scaffold01529	Dinucleotide	(TA)7	14	64206	64219	Ca3	Dinucleotide	(TA)6	12	8814496	8814507	Intergenic	TGTCATCACTCCCTAAAACTG	TCATCACCCTTTATTTCATCA
Ca_GpSSR00356	scaffold26172	Trinucleotide	(TTA)25	75	142	216	Ca3	Trinucleotide	(TTA)14	42	9133095	9133136	Intergenic	CATCATGGATTTCAAAGAGAG	TGAAACTTTAAGTGTTTGCTTC
Ca_GpSSR00357	scaffold00726	Tetranucleotide	(TTTA)8	32	101734	101765	Ca3	Tetranucleotide	(TTTA)6	24	9241047	9241070	Intergenic	GGATCACATGTGGTATTTAGCC	GCACATGTTCACCGACATTC
Ca_GpSSR00358	scaffold02763	Dinucleotide	(AT)11	22	19825	19846	Ca3	Dinucleotide	(AT)10	20	9389985	9390004	Intergenic	CAAATATGCAATAACCTTCCA	AAAAATCTATTTGGTGCTGGT
Ca_GpSSR00359	scaffold02763	Trinucleotide	(AAT)12	36	31063	31098	Ca3	Trinucleotide	(AAT)15	45	9401230	9401274	Intergenic		
Ca_GpSSR00360	scaffold08694	Dinucleotide	(TA)13	26	31449	31474	Ca3	Dinucleotide	(TA)18	36	9561961	9561996	Intergenic	AGGAAGATGAGGTCTAAGTGG	GAGCCCATACTTCCTCTAAGT
Ca_GpSSR00361	scaffold02564	Trinucleotide	(TAT)47	141	45894	46034	Ca3	Trinucleotide	(TAT)34	102	9762104	9762205	Intergenic		
Ca_GpSSR00362	scaffold02071	Dinucleotide	(TA)24	48	13548	13595	Ca3	Dinucleotide	(TA)16	32	10108962	10108993	Intergenic	CAAATATGCAATAACCTTCCA	AAAAATCTATTTGGTGCTGGT
Ca_GpSSR00363	scaffold02031	Dinucleotide	(TA)12	24	12977	13000	Ca3	Dinucleotide	(TA)14	28	10299423	10299450	gene=Ca_16817;mRNA=Ca_16817.1;intron;	GGATGTGTGCAATTGGTATAA	GGGAGGTCATTATATTATTCTTT
Ca_GpSSR00364	scaffold02254	Dinucleotide	(TA)17	34	37669	37702	Ca3	Dinucleotide	(TA)21	42	10488016	10488057	Intergenic	CAAATATGCAATAACCTTCCA	AAAAATCTATTTGGTGCTGGT
Ca_GpSSR00365	scaffold17712	Dinucleotide	(AT)11	22	53	74	Ca3	Dinucleotide	(AT)25	50	10541967	10542016	Intergenic		
Ca_GpSSR00366	scaffold02294	Dinucleotide	(TA)17	34	34354	34387	Ca3	Dinucleotide	(TA)14	28	11163961	11163988	Intergenic	CAAATATGCAATAACCTTCCA	AAAAATCTATTTGGTGCTGGT
Ca_GpSSR00367	scaffold04225	Trinucleotide	(TTA)5	15	3042	3056	Ca3	Trinucleotide	(TTA)7	21	11250531	11250551	Intergenic	CATCATGGATTTCAAAGAGAG	TGAAACTTTAAGTGTTTGCTTC
Ca_GpSSR00368	scaffold01152	Dinucleotide	(AT)20	40	19640	19679	Ca3	Dinucleotide	(AT)23	46	11430132	11430177	Intergenic	AGTTAGAGGGCTAATTAAATGCT	TATACATCGCGCTATCTTCAT
Ca_GpSSR00369	scaffold01152	Dinucleotide	(AT)14	28	67683	67710	Ca3	Dinucleotide	(AT)16	32	11478030	11478061	Intergenic	AGTTAGAGGGCTAATTAAATGCT	TATACATCGCGCTATCTTCAT
Ca_GpSSR00370	scaffold07350	Dinucleotide	(AT)13	26	5176	5201	Ca3	Dinucleotide	(AT)15	30	11652167	11652196	Intergenic	TTTCACTATTGTCGGATGAAT	TTGAAAACACCTCAACTTTT
Ca_GpSSR00371	scaffold00772	Trinucleotide	(AAT)15	45	89080	89124	Ca3	Trinucleotide	(AAT)17	51	11701006	11701056	Intergenic	GCCCACGATATCCCctATTT	GGGAGAGCAAAGAAGAAAGG
Ca_GpSSR00372	scaffold03342	Dinucleotide	(AT)11	22	27717	27738	Ca3	Dinucleotide	(AT)10	20	13647077	13647096	Intergenic	TTCTCCTTCGTCTCCTTTATT	TCTATTGTGATTGATGGTTGA
Ca_GpSSR00373	scaffold00236	Dinucleotide	(AT)14	28	229036	229063	Ca3	Dinucleotide	(AT)13	26	13697651	13697676	Intergenic	ATTTTAAACAAGGCCTTTCA	GAAATCTAATTAGCCTTATCACT
Ca_GpSSR00374	scaffold01688	Trinucleotide	(ATA)20	60	51583	51642	Ca3	Trinucleotide	(ATA)19	57	14383865	14383921	Intergenic	AAAACACACGTAGTTTTTGGA	GCGAGAACATACATCTCACAT
Ca_GpSSR00375	scaffold02359	Dinucleotide	(AT)12	24	1856	1879	Ca3	Dinucleotide	(AT)14	28	14450921	14450948	Intergenic	ACTCATCTCCCACATATACCC	ACATGTTCAATGCTCATTAGG
Ca_GpSSR00376	scaffold03173	Dinucleotide	(TA)17	34	10148	10181	Ca3	Dinucleotide	(TA)23	46	14763435	14763480	Intergenic	AGCGCTATGTGACCTCTTTAT	TTAACTGATCACCCCAATATG
Ca_GpSSR00377	scaffold11433	Trinucleotide	(TAA)11	33	900	932	Ca3	Trinucleotide	(TAA)10	30	14764341	14764370	Intergenic	CTTTCTTCTATTTGTTTCGGTAA	AATTGAACCAATAAAACACG
Ca_GpSSR00378	scaffold02521	Dinucleotide	(AT)12	24	35428	35451	Ca3	Dinucleotide	(AT)15	30	15422425	15422454	Intergenic	CAAATATGCAATAACCTTCCA	AAAAATCTATTTGGTGCTGGT
Ca_GpSSR00379	scaffold00482	Dinucleotide	(TA)6	12	55593	55604	Ca3	Dinucleotide	(TA)14	28	15601971	15601998	Intergenic	AACAACGGTATGAAAAACTCA	GTGAATGGCTAGAATGATTTG
Ca_GpSSR00380	scaffold23143	Dinucleotide	(TA)15	30	34	63	Ca3	Dinucleotide	(TA)14	28	16296327	16296354	Intergenic	CAAATATGCAATAACCTTCCA	AAAAATCTATTTGGTGCTGGT
Ca_GpSSR00381	Ca_LG_3	Dinucleotide	(AT)13	26	12984678	12984703	Ca3	Dinucleotide	(AT)9	18	16563840	16563857	gene=Ca_03057;mRNA=Ca_03057.1;intron;	TGCAATAGTACAAAatAaTTGTAaTCc	tTTGTTTGtTTGACACATTGCT
Ca_GpSSR00382	scaffold09429	Trinucleotide	(TAT)15	45	3923	3967	Ca3	Trinucleotide	(TAT)13	39	16643956	16643994	Intergenic	CCCTTCCTCTTTTTCTTTTCT	AATAAAACATCCCTCTTTAGCA
Ca_GpSSR00383	scaffold00246	Trinucleotide	(TAA)29	87	151828	151914	Ca3	Trinucleotide	(TAA)6	18	16728236	16728253	upstream:Ca_11540;	TTTGTTAACCATTGGATTGAG	CATCCACTGACCAAAAATAAA
Ca_GpSSR00384	scaffold06582	Dinucleotide	(AT)15	30	2834	2863	Ca3	Dinucleotide	(AT)22	44	16735265	16735308	Intergenic	GTCCAATAAACTCAACCAAAA	ATACAGGGGATCGAAATCTT
Ca_GpSSR00385	scaffold55202	Trinucleotide	(ATT)49	147	439	585	Ca3	Trinucleotide	(ATT)43	129	17012109	17012237	Intergenic		
Ca_GpSSR00386	scaffold01196	Dinucleotide	(TA)16	32	26122	26153	Ca3	Dinucleotide	(TA)6	12	17064570	17064581	Intergenic	CAAATATGCAATAACCTTCCA	AAAAATCTATTTGGTGCTGGT
Ca_GpSSR00387	scaffold03408	Dinucleotide	(AT)7	14	17016	17029	Ca3	Dinucleotide	(AT)15	30	17474698	17474727	Intergenic	CAAATATGCAATAACCTTCCA	AAAAATCTATTTGGTGCTGGT
Ca_GpSSR00388	scaffold00499	Dinucleotide	(AT)10	20	24209	24228	Ca3	Dinucleotide	(AT)11	22	17739153	17739174	Intergenic	TTCCTTCTTTCCTTTTCTTTC	TGGTTTGAGAAGTTATGAGTGA
Ca_GpSSR00389	scaffold00499	Dinucleotide	(AT)16	32	24844	24875	Ca3	Dinucleotide	(AT)18	36	17739797	17739832	Intergenic	CACCCTAATTTTGGAATCTTT	TTGCTACTTCATTTTACCATCA
Ca_GpSSR00390	scaffold00499	Dinucleotide	(AT)21	42	97951	97992	Ca3	Dinucleotide	(AT)16	32	17819481	17819512	Intergenic	CACCCTAATTTTGGAATCTTT	TTGCTACTTCATTTTACCATCA
Ca_GpSSR00391	scaffold00675	Dinucleotide	(AT)10	20	19071	19090	Ca3	Dinucleotide	(AT)9	18	17978670	17978687	Intergenic	AACGAATTAACTCACATCCAT	TTCTACTCAAGAGCCAACAAC
Ca_GpSSR00392	scaffold00675	Dinucleotide	(AT)19	38	23505	23542	Ca3	Dinucleotide	(AT)14	28	17982996	17983023	Intergenic	CAAATATGCAATAACCTTCCA	AAAAATCTATTTGGTGCTGGT
Ca_GpSSR00393	scaffold00675	Dinucleotide	(TA)13	26	56244	56269	Ca3	Dinucleotide	(TA)15	30	18015979	18016008	Intergenic	CAAATATGCAATAACCTTCCA	AAAAATCTATTTGGTGCTGGT
Ca_GpSSR00394	scaffold00675	Dinucleotide	(AT)12	24	83645	83668	Ca3	Dinucleotide	(AT)15	30	18045479	18045508	Intergenic	CAAATATGCAATAACCTTCCA	AAAAATCTATTTGGTGCTGGT
Ca_GpSSR00395	scaffold06136	Dinucleotide	(CT)7	14	3477	3490	Ca3	Dinucleotide	(CT)8	16	18311713	18311728	Intergenic		
Ca_GpSSR00396	scaffold00761	Dinucleotide	(GT)6	12	32355	32366	Ca3	Dinucleotide	(GT)7	14	19156139	19156152	Intergenic	ATGATTAGCTGCAAAATAGGC	TTTTGTCCCTCCTTTCATAA
Ca_GpSSR00397	scaffold42371	Trinucleotide	(TAT)7	21	708	728	Ca3	Trinucleotide	(TAT)6	18	19610530	19610547	Intergenic		
Ca_GpSSR00398	Ca_LG_3	Dinucleotide	(AT)8	16	13800310	13800325	Ca3	Dinucleotide	(AT)7	14	19776383	19776396	Intergenic	AATCCACAAACACAACAAAAC	CCAATCAACTCATTGAGACC
Ca_GpSSR00399	scaffold111852	Dinucleotide	(TA)13	26	311	336	Ca3	Dinucleotide	(TA)11	22	20053411	20053432	Intergenic	CAAATATGCAATAACCTTCCA	AAAAATCTATTTGGTGCTGGT
Ca_GpSSR00400	scaffold32929	Dinucleotide	(TA)18	36	8	43	Ca3	Dinucleotide	(TA)8	16	20272661	20272676	Intergenic	CAAATATGCAATAACCTTCCA	AAAAATCTATTTGGTGCTGGT
Ca_GpSSR00401	scaffold02646	Trinucleotide	(AAT)10	30	10978	11007	Ca3	Trinucleotide	(AAT)13	39	20537462	20537500	Intergenic	TTGCCTACTTTTCATGAGTTTG	TCTCGTTTAAAATCAATTATTCCG
Ca_GpSSR00402	scaffold00718	Tetranucleotide	(TTAA)8	32	32797	32828	Ca3	Tetranucleotide	(TTAA)9	36	20813329	20813364	Intergenic	TCGTCATATTTAGGATTTGGA	TTTGGCATAGTTAGTAATGTCC

Ca_GpSSR00403	scaffold00718	Dinucleotide	(AT)12	24	76254	76277	Ca3	Dinucleotide	(AT)18	36	20857080	20857115	Intergenic	TGTGTCTTAGTATCGATTTCA	TTGTCAACTAATGACAACCATT
Ca_GpSSR00404	scaffold00718	Dinucleotide	(TA)17	34	78691	78724	Ca3	Dinucleotide	(TA)12	24	20859498	20859521	Intergenic	TGTGTCTTAGTATCGATTTCA	TTGTCAACTAATGACAACCATT
Ca_GpSSR00405	Ca_LG_3	Dinucleotide	(TA)14	28	12228989	12229016	Ca3	Dinucleotide	(TA)10	20	20947307	20947326	Intergenic	CAAATATGCAATAACCTTCCA	AAAAATCTATTTGGTGCTGGT
Ca_GpSSR00406	Ca_LG_3	Dinucleotide	(TA)11	22	12227011	12227032	Ca3	Dinucleotide	(TA)10	20	20949287	20949306	Intergenic	AATCCACAAACACAACAAAAC	CCAATCAACTCATTGAGACC
Ca_GpSSR00407	Ca_LG_3	Dinucleotide	(TA)13	26	12053446	12053471	Ca3	Dinucleotide	(TA)21	42	21125541	21125582	Intergenic	GAGCACCAATTAAAGAGGGC	GGCATTTAAAACATAAATCCCAA
Ca_GpSSR00408	scaffold77392	Dinucleotide	(AT)20	40	444	483	Ca3	Dinucleotide	(AT)17	34	21159893	21159926	Intergenic	CAAATATGCAATAACCTTCCA	AAAAATCTATTTGGTGCTGGT
Ca_GpSSR00409	Ca_LG_3	Trinucleotide	(TAT)16	48	11580342	11580389	Ca3	Trinucleotide	(TAT)17	51	21481671	21481721	Intergenic		
Ca_GpSSR00410	Ca_LG_3	Trinucleotide	(TAT)28	84	11603350	11603433	Ca3	Trinucleotide	(TAT)16	48	21503440	21503487	Intergenic		
Ca_GpSSR00411	Ca_LG_3	Trinucleotide	(TAT)20	60	11849609	11849668	Ca3	Trinucleotide	(TAT)19	57	21760134	21760190	Intergenic		
Ca_GpSSR00412	Ca_LG_3	Dinucleotide	(TA)10	20	11857779	11857798	Ca3	Dinucleotide	(TA)11	22	21767899	21767920	Intergenic	AATCCACAAACACAACAAAAC	CCAATCAACTCATTGAGACC
Ca_GpSSR00413	Ca_LG_3	Trinucleotide	(AAT)11	33	12239480	12239512	Ca3	Trinucleotide	(AAT)10	30	21910993	21911022	Intergenic	AATCTGACCATAAGTTGACGA	AGAACTATTACTGCATACCTCCA
Ca_GpSSR00414	scaffold00288	Dinucleotide	(AT)11	22	200892	200913	Ca3	Dinucleotide	(AT)12	24	22287301	22287324	gene=Ca_11842;mRNA=Ca_11842.1;intron;	GTAGCTTTTGCCCTAAAAATC	GTTCTACCGCAACATTAAGAA
Ca_GpSSR00415	scaffold06173	Dinucleotide	(TA)15	30	2648	2677	Ca3	Dinucleotide	(TA)13	26	22422347	22422372	Intergenic	CATGTTTCGTGATTACTACTTCA	TGCTGTTCATATTATTGGTGA
Ca_GpSSR00416	scaffold71035	Dinucleotide	(AT)7	14	436	449	Ca3	Dinucleotide	(AT)12	24	22439660	22439683	Intergenic	TATGCAACCATGGCTATAACT	TAGATTTTTCCTGATGAGTCG
Ca_GpSSR00417	scaffold05715	Dinucleotide	(AT)13	26	10213	10238	Ca3	Dinucleotide	(AT)18	36	22478436	22478471	Intergenic	CAAATATGCAATAACCTTCCA	AAAAATCTATTTGGTGCTGGT
Ca_GpSSR00418	scaffold23738	Trinucleotide	(AAT)18	54	1263	1316	Ca3	Trinucleotide	(AAT)19	57	22651901	22651957	Intergenic	TTAAGCTCAAACAAACGAATC	ACCTTTTTAGACAATAGATGTCA
Ca_GpSSR00419	Ca_LG_3	Trinucleotide	(ATA)7	21	6257604	6257624	Ca3	Trinucleotide	(ATA)5	15	22850419	22850433	upstream:Ca_02650;	TCATCTCCTGCTCCAGTATTA	TTGTTGAGGTCACTCACTTCT
Ca_GpSSR00420	scaffold11189	Dinucleotide	(AT)7	14	759	772	Ca3	Dinucleotide	(AT)34	68	22930707	22930774	Intergenic	TcTCACAAGGATGGGAACAA	ATTAtCCAGGTTgACGCTGC
Ca_GpSSR00421	Ca_LG_3	Trinucleotide	(ATT)9	27	12843457	12843483	Ca3	Trinucleotide	(ATT)8	24	23219722	23219745	Intergenic		
Ca_GpSSR00422	scaffold00903	Dinucleotide	(AT)10	20	32968	32987	Ca3	Dinucleotide	(AT)12	24	23512634	23512657	gene=Ca_14436;mRNA=Ca_14436.1;intron;	GGTATTTCGTCAACAACAAAA	AGAGAACTTAAGCCATTGAGC
Ca_GpSSR00423	scaffold00903	Dinucleotide	(AT)9	18	11342	11359	Ca3	Dinucleotide	(AT)12	24	23530918	23530941	Intergenic	TCGTCAACGAAAATCCTATTA	TTAAGCGGTGGAGCTATAAAT
Ca_GpSSR00424	scaffold00348	Trinucleotide	(AAT)9	27	73617	73643	Ca3	Trinucleotide	(AAT)10	30	23619758	23619787	Intergenic		
Ca_GpSSR00425	scaffold00348	Dinucleotide	(TC)7	14	75784	75797	Ca3	Dinucleotide	(TC)36	72	23621929	23622000	upstream:Ca_12222;	ATAACAGGCTCTCACTCCTCT	CAAAACAAGGAATGCAGAATA
Ca_GpSSR00426	Ca_LG_3	Dinucleotide	(TA)17	34	11157400	11157433	Ca3	Dinucleotide	(TA)16	32	24035594	24035625	Intergenic	CAAATATGCAATAACCTTCCA	AAAAATCTATTTGGTGCTGGT
Ca_GpSSR00427	Ca_LG_3	Dinucleotide	(TA)18	36	11159660	11159695	Ca3	Dinucleotide	(TA)16	32	24037477	24037508	Intergenic	CAAATATGCAATAACCTTCCA	AAAAATCTATTTGGTGCTGGT
Ca_GpSSR00428	Ca_LG_3	Trinucleotide	(AAT)20	60	11236544	11236603	Ca3	Trinucleotide	(AAT)21	63	24112136	24112198	upstream:Ca_02924;	TTAAGCTCAAACAAACGAATC	ACCTTTTTAGACAATAGATGTCA
Ca_GpSSR00429	Ca_LG_3	Dinucleotide	(TA)13	26	11290602	11290627	Ca3	Dinucleotide	(TA)11	22	24156924	24156945	Intergenic	CAAATATGCAATAACCTTCCA	AAAAATCTATTTGGTGCTGGT
Ca_GpSSR00430	scaffold135233	Dinucleotide	(AT)7	14	259	272	Ca3	Dinucleotide	(AT)12	24	24387026	24387049	Intergenic	CAAATATGCAATAACCTTCCA	AAAAATCTATTTGGTGCTGGT
Ca_GpSSR00431	scaffold01547	Dinucleotide	(AT)13	26	68376	68401	Ca3	Dinucleotide	(AT)14	28	24789267	24789294	Intergenic	TGCAAGGTAAAATCGTAGAAG	TATGTACATGTGATGGTGGTG
Ca_GpSSR00432	scaffold00619	Trinucleotide	(TAT)5	15	3	17	Ca3	Trinucleotide	(TAT)19	57	24809699	24809755	Intergenic		
Ca_GpSSR00433	scaffold00270	Dinucleotide	(AT)16	32	29180	29211	Ca3	Dinucleotide	(AT)22	44	25181723	25181766	Intergenic	GACCCTACGACTAATTGAAAAA	TCGGAGAATATGAGAATGAGA
Ca_GpSSR00434	Ca_LG_3	Dinucleotide	(AT)14	28	10049346	10049373	Ca3	Dinucleotide	(AT)17	34	25215268	25215301	Intergenic	CAAATATGCAATAACCTTCCA	AAAAATCTATTTGGTGCTGGT
Ca_GpSSR00435	scaffold00679	Dinucleotide	(TA)10	20	100016	100035	Ca3	Dinucleotide	(TA)11	22	25937656	25937677	Intergenic	CAAATATGCAATAACCTTCCA	AAAAATCTATTTGGTGCTGGT
Ca_GpSSR00436	scaffold03883	Dinucleotide	(AT)12	24	23137	23160	Ca3	Dinucleotide	(AT)10	20	26020459	26020478	Intergenic	CGCCCCACTGTTAATTTT	AATTGAATTTTTCCACTCCAC
Ca_GpSSR00437	Ca_LG_3	Dinucleotide	(AT)16	32	9885008	9885039	Ca3	Dinucleotide	(AT)6	12	26388749	26388760	Intergenic	TGATTTAATTATCGATGTGAGTTTCAA	TTGAGTTAATGAAACAAATAAGTCGAA
Ca_GpSSR00438	scaffold03779	Dinucleotide	(TA)8	16	19271	19286	Ca3	Dinucleotide	(TA)14	28	27573782	27573809	Intergenic	ATTTCTTATGGgCATGGgTG	GAATAGTCAAAGCGATAAAATAAATGA
Ca_GpSSR00439	scaffold00201	Dinucleotide	(TA)13	26	142773	142798	Ca3	Dinucleotide	(TA)12	24	27725675	27725698	Intergenic	CAAATATGCAATAACCTTCCA	AAAAATCTATTTGGTGCTGGT
Ca_GpSSR00440	scaffold00201	Dinucleotide	(TA)13	26	243409	243434	Ca3	Dinucleotide	(TA)8	16	27812739	27812754	Intergenic	CAAATATGCAATAACCTTCCA	AAAAATCTATTTGGTGCTGGT
Ca_GpSSR00441	Ca_LG_3	Trinucleotide	(AAT)10	30	8889359	8889388	Ca3	Trinucleotide	(AAT)8	24	27949909	27949932	Intergenic	AATCTGACCATAAGTTGACGA	AGAACTATTACTGCATACCTCCA
Ca_GpSSR00442	Ca_LG_3	Dinucleotide	(AT)10	20	8906044	8906063	Ca3	Dinucleotide	(AT)9	18	27966590	27966607	Intergenic	AATCCACAAACACAACAAAAC	CCAATCAACTCATTGAGACC
Ca_GpSSR00443	scaffold08338	Dinucleotide	(AT)7	14	4536	4549	Ca3	Dinucleotide	(AT)12	24	28035978	28036001	Intergenic	ACCATCTCCAACGTAAAGATT	GATCGCGTATTACCAAAATAA
Ca_GpSSR00444	Ca_LG_3	Dinucleotide	(AT)9	18	8996672	8996689	Ca3	Dinucleotide	(AT)8	16	28051428	28051443	Intergenic	AATCCACAAACACAACAAAAC	CCAATCAACTCATTGAGACC
Ca_GpSSR00445	Ca_LG_3	Dinucleotide	(TA)13	26	8546278	8546303	Ca3	Dinucleotide	(TA)14	28	28932041	28932068	upstream:Ca_02776;	CAAATATGCAATAACCTTCCA	AAAAATCTATTTGGTGCTGGT
Ca_GpSSR00446	scaffold00077	Dinucleotide	(TA)8	16	238643	238658	Ca3	Dinucleotide	(TA)6	12	30420824	30420835	Intergenic	CAAATATGCAATAACCTTCCA	AAAAATCTATTTGGTGCTGGT
Ca_GpSSR00447	scaffold00077	Trinucleotide	(AAT)16	48	310112	310159	Ca3	Trinucleotide	(AAT)14	42	30491265	30491306	Intergenic		
Ca_GpSSR00448	scaffold00077	Dinucleotide	(AT)10	20	360596	360615	Ca3	Dinucleotide	(AT)9	18	30546285	30546302	Intergenic	CAAATATGCAATAACCTTCCA	AAAAATCTATTTGGTGCTGGT
Ca_GpSSR00449	scaffold13233	Trinucleotide	(TAT)16	48	2486	2533	Ca3	Trinucleotide	(TAT)17	51	30586755	30586805	Intergenic	AGTTTACTATTTTCCCCTTTC	TCACAAATTCTCCAAACTGAT
Ca_GpSSR00450	scaffold02596	Dinucleotide	(AT)20	40	10890	10929	Ca3	Dinucleotide	(AT)16	32	30634653	30634684	Intergenic	ACACACACACATAACACACGTA	GGATAAGAGAAAGAACCCTTG
Ca_GpSSR00451	Ca_LG_3	Dinucleotide	(AT)15	30	7054559	7054588	Ca3	Dinucleotide	(AT)9	18	30841155	30841172	upstream:Ca_02730;	CAAATATGCAATAACCTTCCA	AAAAATCTATTTGGTGCTGGT
Ca_GpSSR00452	Ca_LG_3	Dinucleotide	(AT)11	22	7042247	7042268	Ca3	Dinucleotide	(AT)13	26	30852578	30852603	Intergenic	AATCCACAAACACAACAAAAC	CCAATCAACTCATTGAGACC
Ca_GpSSR00453	Ca_LG_3	Dinucleotide	(TA)8	16	6953286	6953301	Ca3	Dinucleotide	(TA)18	36	30941968	30942003	Intergenic	AATCCACAAACACAACAAAAC	CCAATCAACTCATTGAGACC
Ca_GpSSR00454	scaffold00729	Dinucleotide	(TA)7	14	90636	90649	Ca3	Dinucleotide	(TA)8	16	31128980	31128995	Intergenic	AAGCTGAGGTAACATCGATTC	TACAAATTCCACGTGATCTTC
Ca_GpSSR00455	scaffold69776	Dinucleotide	(AT)22	44	486	529	Ca3	Dinucleotide	(AT)12	24	31259694	31259717	Intergenic	CAAATATGCAATAACCTTCCA	AAAAATCTATTTGGTGCTGGT
Ca_GpSSR00456	Ca_LG_3	Dinucleotide	(AT)17	34	6784924	6784957	Ca3	Dinucleotide	(AT)18	36	31293319	31293354	Intergenic	CAAATATGCAATAACCTTCCA	AAAAATCTATTTGGTGCTGGT
Ca_GpSSR00457	Ca_LG_3	Dinucleotide	(TA)7	14	6717732	6717745	Ca3	Dinucleotide	(TA)6	12	31361082	31361093	mRNA=Ca_02697.1;gene=Ca_02697;intron;	CAAATATGCAATAACCTTCCA	AAAAATCTATTTGGTGCTGGT
Ca_GpSSR00458	Ca_LG_3	Dinucleotide	(TA)9	18	6598132	6598149	Ca3	Dinucleotide	(TA)14	28	31474732	31474759	Intergenic	TCAAAGGCAATTTTGATTGAA	TTTTTGATAGTCGCAGGAcCTT
Ca_GpSSR00459	Ca_LG_3	Dinucleotide	(TA)12	24	6355894	6355917	Ca3	Dinucleotide	(TA)11	22	31540732	31540753	gene=Ca_02661;mRNA=Ca_02661.1;intron;	CAAATATGCAATAACCTTCCA	AAAAATCTATTTGGTGCTGGT
Ca_GpSSR00460	Ca_LG_3	Dinucleotide	(AT)8	16	6378639	6378654	Ca3	Dinucleotide	(AT)13	26	31576728	31576753	Intergenic	AATCCACAAACACAACAAAAC	CCAATCAACTCATTGAGACC
Ca_GpSSR00461	scaffold00198	Dinucleotide	(TA)10	20	4434	4453	Ca3	Dinucleotide	(TA)26	52	31735989	31736040	mRNA=Ca_11252.1;gene=Ca_11252;intron;	ACACAAAACACACACACACAC	CAACTTGTCGACGATGTATAA
Ca_GpSSR00462	scaffold00198	Dinucleotide	(TA)9	18	21316	21333	Ca3	Dinucleotide	(TA)8	16	31754643	31754658	Intergenic	CAAATATGCAATAACCTTCCA	AAAAATCTATTTGGTGCTGGT
Ca_GpSSR00463	scaffold00198	Dinucleotide	(TA)12	24	22060	22083	Ca3	Dinucleotide	(TA)11	22	31755385	31755406	Intergenic	CAAATATGCAATAACCTTCCA	AAAAATCTATTTGGTGCTGGT
Ca_GpSSR00464	scaffold00198	Dinucleotide	(AT)10	20	252116	252135	Ca3	Dinucleotide	(AT)11	22	31982547	31982568	downstream:Ca_11272;	CAAATATGCAATAACCTTCCA	AAAAATCTATTTGGTGCTGGT
Ca_GpSSR00465	Ca_LG_5	Dinucleotide	(AT)8	16	16100890	16100905	Ca3	Dinucleotide	(AT)25	50	32394131	32394180	Intergenic	CTTCTTTACATCTGCTCTCCA	GCGCAAAATTCCTTTTAGTAT
Ca_GpSSR00466	scaffold01422	Dinucleotide	(AT)15	30	5086	5115	Ca3	Dinucleotide	(AT)18	36	32399896	32399931	Intergenic	AACTTGCATACTTATGCCTTG	TTTCAATGTACTTCACCGTTT
Ca_GpSSR00467	scaffold07745	Trinucleotide	(AAT)12	36	7163	7198	Ca3	Trinucleotide	(AAT)13	39	32589346	32589384	Intergenic		
Ca_GpSSR00468	scaffold08026	Dinucleotide	(AT)6	12	2236	2247	Ca3	Dinucleotide	(AT)7	14	32996407	32996420	Intergenic	CAAAAAGGTTTTGATTGTCCT	GCAAGAACGACGTGATAATAG
Ca_GpSSR00469	scaffold02065	Dinucleotide	(AT)19	38	1806	1843	Ca3	Dinucleotide	(AT)14	28	33301076	33301103	Intergenic	CAAATATGCAATAACCTTCCA	AAAAATCTATTTGGTGCTGGT
Ca_GpSSR00470	scaffold01300	Trinucleotide	(TAA)14	42	42091	42132	Ca3	Trinucleotide	(TAA)15	45	33424356	33424400	Intergenic	AAATGACCAAAATAGCCAACT	GACTTAACATCAATTTCTTACCA
Ca_GpSSR00471	scaffold01346	Trinucleotide	(ATA)21	63	56446	56508	Ca3	Trinucleotide	(ATA)20	60	33733175	33733234	Intergenic		
Ca_GpSSR00472	scaffold00767	Dinucleotide	(TA)27	54	52777	52830	Ca3	Dinucleotide	(TA)19	38	33953817	33953854	Intergenic	CAAATATGCAATAACCTTCCA	AAAAATCTATTTGGTGCTGGT
Ca_GpSSR00473	scaffold67063	Dinucleotide	(TA)9	18	148	165	Ca3	Dinucleotide	(TA)10	20	33954194	33954213	Intergenic	TGTAGATATGCTTCCCTCTCTT	TTTCCTATCCATTAACCATGA
Ca_GpSSR00474	scaffold67063	Dinucleotide	(TA)11	22	331	352	Ca3	Dinucleotide	(TA)10	20	33954379	33954398	Intergenic	TGTAGATATGCTTCCCTCTCTT	TTTCCTATCCATTAACCATGA
Ca_GpSSR00475	scaffold14097	Dinucleotide	(AT)20	40	1	40	Ca3	Dinucleotide	(AT)15	30	34430333	34430362	Intergenic	CAAATATGCAATAACCTTCCA	AAAAATCTATTTGGTGCTGGT
Ca_GpSSR00476	scaffold27121	Trinucleotide	(ATA)34	102	1112	1213	Ca3	Trinucleotide	(ATA)32	96	34495626	34495721	Intergenic		
Ca_GpSSR00477	Ca_LG_3	Dinucleotide	(TA)18	36	4824557	4824592	Ca3	Dinucleotide	(TA)21	42	35051582	35051623	upstream:Ca_02586;	CAAATATGCAATAACCTTCCA	AAAAATCTATTTGGTGCTGGT
Ca_GpSSR00478	Ca_LG_1	Dinucleotide	(AT)6	12	5257855	5257866	Ca3	Dinucleotide	(AT)8	16	35365461	35365476	Intergenic	CAAATATGCAATAACCTTCCA	AAAAATCTATTTGGTGCTGGT
Ca_GpSSR00479	Ca_LG_1	Dinucleotide	(AT)13	26	5252128	5252153	Ca3	Dinucleotide	(AT)7	14	35371166	35371179	Intergenic	CAAATATGCAATAACCTTCCA	AAAAATCTATTTGGTGCTGGT
Ca_GpSSR00480	scaffold00089	Dinucleotide	(AT)6	12	91723	91734	Ca3	Dinucleotide	(AT)8	16	35686184	35686199	Intergenic	CAAATATGCAATAACCTTCCA	AAAAATCTATTTGGTGCTGGT
Ca_GpSSR00481	Ca_LG_3	Trinucleotide	(TTA)25	75	3805284	3805358	Ca3	Trinucleotide	(TTA)33	99	35840378	35840476	upstream:Ca_02474;	TtAattttgTTTTTGttCTTtcgTt	GAGAAGGAGGCTGAGCAAAA
Ca_GpSSR00482	Ca_LG_3	Dinucleotide	(TA)7	14	3823442	3823455	Ca3	Dinucleotide	(TA)8	16	35859166	35859181	Intergenic	CAAATATGCAATAACCTTCCA	AAAAATCTATTTGGTGCTGGT
Ca_GpSSR00483	Ca_LG_3	Dinucleotide	(AT)8	16	3907503	3907518	Ca3	Dinucleotide	(AT)11	22	35943336	35943357	Intergenic	CGAACACTGATGCCAAACAA	AAAATGTATTTTATGAGTTTGGCCTAT

Ca_GpSSR00484	Ca_LG_3	Dinucleotide	(GA)8	16	4006341	4006356	Ca3	Dinucleotide	(GA)7	14	36037713	36037726	mRNA=Ca_02498.2;gene=Ca_02498;intron;	AGTTTTGAGGGTGCTTCTTAT	CAGTCCTCTACCACTTCACAC
Ca_GpSSR00485	Ca_LG_3	Dinucleotide	(AT)16	32	4152478	4152509	Ca3	Dinucleotide	(AT)12	24	36184022	36184045	Intergenic	CAAATATGCAATAACCTTCCA	AAAAATCTATTTGGTGCTGGT
Ca_GpSSR00486	Ca_LG_3	Dinucleotide	(GA)8	16	4188189	4188204	Ca3	Dinucleotide	(GA)9	18	36218739	36218756	mRNA=Ca_02520.1;gene=Ca_02520;intron;	AGTTTTGAGGGTGCTTCTTAT	CAGTCCTCTACCACTTCACAC
Ca_GpSSR00487	Ca_LG_3	Dinucleotide	(AG)8	16	4194481	4194496	Ca3	Dinucleotide	(AG)6	12	36225055	36225066	mRNA=Ca_02521.1;gene=Ca_02521;exon=Ca_02521.	TGAAATTCAGCAACGAAATGA	cCCAGTAACCGCTCTCTCTG
Ca_GpSSR00488	Ca_LG_3	Dinucleotide	(TA)9	18	4217827	4217844	Ca3	Dinucleotide	(TA)12	24	36248881	36248904	mRNA=Ca_02526.1;3'-UTR=Ca_02526.1.3'-UTR1;gene	GCAGGGgcTATATGGATTTG	GCATGAATTCAGCAGAAGAAAA
Ca_GpSSR00489	Ca_LG_3	Dinucleotide	(CT)8	16	4298387	4298402	Ca3	Dinucleotide	(CT)9	18	36329058	36329075	gene=Ca_02535;mRNA=Ca_02535.1;intron;	CAACTCTCAACTCCCTAATCC	AGTGAGTGAGGTAGGGAGAAA
Ca_GpSSR00490	Ca_LG_3	Trinucleotide	(ACC)5	15	4366997	4367011	Ca3	Trinucleotide	(ACC)6	18	36397764	36397781	mRNA=Ca_02544.2;exon=Ca_02544.2.exon3;CDS=Ca_	CAACATCAATTCATTCCAAGT	AGAGGTCTTGAAGAGGATCTG
Ca_GpSSR00491	scaffold01341	Dinucleotide	(AT)19	38	8088	8125	Ca3	Dinucleotide	(AT)10	20	36484834	36484853	Intergenic	CAAATATGCAATAACCTTCCA	AAAAATCTATTTGGTGCTGGT
Ca_GpSSR00492	scaffold01341	Dinucleotide	(TA)17	34	77063	77096	Ca3	Dinucleotide	(TA)6	12	36554091	36554102	Intergenic	CAAATATGCAATAACCTTCCA	AAAAATCTATTTGGTGCTGGT
Ca_GpSSR00493	scaffold07075	Dinucleotide	(TA)8	16	5587	5602	Ca3	Dinucleotide	(TA)13	26	36740349	36740374	Intergenic	CAAATATGCAATAACCTTCCA	AAAAATCTATTTGGTGCTGGT
Ca_GpSSR00494	scaffold76366	Dinucleotide	(TA)15	30	455	484	Ca3	Dinucleotide	(TA)12	24	36764988	36765011	Intergenic	CAAATATGCAATAACCTTCCA	AAAAATCTATTTGGTGCTGGT
Ca_GpSSR00495	scaffold00394	Dinucleotide	(AT)10	20	72997	73016	Ca3	Dinucleotide	(AT)6	12	37620448	37620459	Intergenic	GTGGGCTCTTCCAATAATACT	ATTTTCTTAAGGTTCGAGTGG
Ca_GpSSR00496	scaffold07873	Pentanucleotide	(CCATA)6	30	6639	6668	Ca3	Pentanucleotide	(CCATA)5	25	37663389	37663413	Intergenic	TTTTCTTCCCTGACACAGTTA	AGATACCGCAGCATGTATTTA
Ca_GpSSR00497	scaffold00159	Trinucleotide	(ATT)45	135	158858	158992	Ca3	Trinucleotide	(ATT)21	63	37881773	37881835	Intergenic		
Ca_GpSSR00498	Ca_LG_3	Dinucleotide	(TA)15	30	3559952	3559981	Ca3	Dinucleotide	(TA)13	26	38096031	38096056	Intergenic	CAAATATGCAATAACCTTCCA	AAAAATCTATTTGGTGCTGGT
Ca_GpSSR00499	Ca_LG_3	Dinucleotide	(AT)15	30	3313388	3313417	Ca3	Dinucleotide	(AT)11	22	38406108	38406129	Intergenic	TTTTtCTCCCTTGACTAATTGATTTT	TGGTTGATTTGGTAACATTTTTCA
Ca_GpSSR00500	Ca_LG_3	Dinucleotide	(AT)19	38	2692579	2692616	Ca3	Dinucleotide	(AT)16	32	38784891	38784922	upstream:Ca_02356;	CAAATATGCAATAACCTTCCA	AAAAATCTATTTGGTGCTGGT
Ca_GpSSR00501	scaffold06011	Dinucleotide	(TA)19	38	10347	10384	Ca4	Dinucleotide	(TA)11	22	323025	323046	Intergenic	AGAAAATCAGAAGTTGCATT	AAAGTTTGGTGGTGAGTTTTT
Ca_GpSSR00502	scaffold08110	Dinucleotide	(TA)11	22	1	22	Ca4	Dinucleotide	(TA)19	38	717706	717743	Intergenic		
Ca_GpSSR00503	scaffold39089	Dinucleotide	(TA)20	40	840	879	Ca4	Dinucleotide	(TA)11	22	817007	817028	Intergenic	AGAAAATCAGAAGTTGCATT	AAAGTTTGGTGGTGAGTTTTT
Ca_GpSSR00504	Ca_LG_4	Trinucleotide	(ATA)21	63	18982147	18982209	Ca4	Trinucleotide	(ATA)13	39	1104792	1104830	Intergenic	TCACTTTTACATTACACAcAAGGATt	CATGTCTTTTGATATACTACCTCCACA
Ca_GpSSR00505	Ca_LG_4	Dinucleotide	(AT)14	28	19003323	19003350	Ca4	Dinucleotide	(AT)33	66	1126522	1126587	Intergenic		
Ca_GpSSR00506	scaffold05698	Dinucleotide	(AT)9	18	4046	4063	Ca4	Dinucleotide	(AT)8	16	1592014	1592029	Intergenic	AGAAAATCAGAAGTTGCATT	AAAGTTTGGTGGTGAGTTTTT
Ca_GpSSR00507	scaffold19337	Dinucleotide	(TA)8	16	1746	1761	Ca4	Dinucleotide	(TA)10	20	1627334	1627353	Intergenic	AGAAAATCAGAAGTTGCATT	AAAGTTTGGTGGTGAGTTTTT
Ca_GpSSR00508	scaffold39578	Dinucleotide	(AT)17	34	838	871	Ca4	Dinucleotide	(AT)22	44	1732272	1732315	Intergenic	GGTGTCAAAAAGGTATAAAGGGG	CCTCGTGTCATTATTATATTTAACGGT
Ca_GpSSR00509	Ca_LG_4	Trinucleotide	(AAT)14	42	18361599	18361640	Ca4	Trinucleotide	(AAT)5	15	1863084	1863098	Intergenic		
Ca_GpSSR00510	Ca_LG_4	Trinucleotide	(TAT)6	18	18363535	18363552	Ca4	Trinucleotide	(TAT)5	15	1864782	1864796	gene=Ca_05007;mRNA=Ca_05007.1;intron;		
Ca_GpSSR00511	Ca_LG_4	Dinucleotide	(TA)8	16	18432941	18432956	Ca4	Dinucleotide	(TA)17	34	1927537	1927570	Intergenic	ACGATATCGACATTTTGTCA	CGTACATATGCACCGAAAT
Ca_GpSSR00512	Ca_LG_4	Dinucleotide	(TA)25	50	18464251	18464300	Ca4	Dinucleotide	(TA)8	16	1962951	1962966	Intergenic	TTAAAGACCCTTTTTGGAATC	TCAATCTTTTACTCTCGCAAT
Ca_GpSSR00513	Ca_LG_4	Dinucleotide	(AT)10	20	18680223	18680242	Ca4	Dinucleotide	(AT)8	16	2221335	2221350	Intergenic	AGAAAATCAGAAGTTGCATT	AAAGTTTGGTGGTGAGTTTTT
Ca_GpSSR00514	Ca_LG_4	Dinucleotide	(AT)8	16	17957278	17957293	Ca4	Dinucleotide	(AT)9	18	2714317	2714334	Intergenic	AGAAAATCAGAAGTTGCATT	AAAGTTTGGTGGTGAGTTTTT
Ca_GpSSR00515	scaffold13487	Dinucleotide	(AT)7	14	1	14	Ca4	Dinucleotide	(AT)20	40	2833839	2833878	Intergenic		
Ca_GpSSR00516	scaffold08065	Dinucleotide	(TA)10	20	175	194	Ca4	Dinucleotide	(TA)9	18	3203026	3203043	Intergenic	AGAAAATCAGAAGTTGCATT	AAAGTTTGGTGGTGAGTTTTT
Ca_GpSSR00517	scaffold58946	Dinucleotide	(AT)23	46	570	615	Ca4	Dinucleotide	(AT)32	64	3313522	3313585	Intergenic		
Ca_GpSSR00518	scaffold00263	Dinucleotide	(AT)12	24	32642	32665	Ca4	Dinucleotide	(AT)10	20	3695492	3695511	upstream:Ca_11658;	CTAAGTTTTAACTCAATTGTCAC	TCTTTCTTGTACCCTTCTTCG
Ca_GpSSR00519	scaffold00263	Dinucleotide	(AT)9	18	224482	224499	Ca4	Dinucleotide	(AT)27	54	3961170	3961223	Intergenic	ACATAGCTCTGGACCAAAAA	TTCATAACACTCTCCCTTGAA
Ca_GpSSR00520	scaffold01962	Dinucleotide	(AT)14	28	51358	51385	Ca4	Dinucleotide	(AT)7	14	4014739	4014752	Intergenic	AGAAAATCAGAAGTTGCATT	AAAGTTTGGTGGTGAGTTTTT
Ca_GpSSR00521	Ca_LG_4	Dinucleotide	(TA)10	20	16984989	16985008	Ca4	Dinucleotide	(TA)16	32	4062811	4062842	mRNA=Ca_04950.1;gene=Ca_04950;intron;	AATGACGCAGGTTCAACACA	ATGTCATTATGTTGCATTAAGATCTAC
Ca_GpSSR00522	Ca_LG_4	Dinucleotide	(TA)6	12	16958079	16958090	Ca4	Dinucleotide	(TA)7	14	4089875	4089888	3'-UTR=Ca_04949.1.3'-UTR1;exon=Ca_04949.1.exon6	AGATGTACAATCTTTTGAGACAA	GCTGCAAAATGGTACATAGAC
Ca_GpSSR00523	Ca_LG_4	Dinucleotide	(TA)9	18	16887902	16887919	Ca4	Dinucleotide	(TA)8	16	4161931	4161946	Intergenic	AGAAAATCAGAAGTTGCATT	AAAGTTTGGTGGTGAGTTTTT
Ca_GpSSR00524	Ca_LG_4	Dinucleotide	(TA)6	12	16879703	16879714	Ca4	Dinucleotide	(TA)8	16	4171523	4171538	Intergenic	AGATGTACAATCTTTTGAGACAA	GCTGCAAAATGGTACATAGAC
Ca_GpSSR00525	scaffold13064	Dinucleotide	(AT)17	34	3392	3425	Ca4	Dinucleotide	(AT)8	16	4266247	4266262	Intergenic	AGAAAATCAGAAGTTGCATT	AAAGTTTGGTGGTGAGTTTTT
Ca_GpSSR00526	scaffold76751	Trinucleotide	(TAT)60	180	260	439	Ca4	Trinucleotide	(TAT)18	54	4266684	4266737	Intergenic	CATTCCTTCAGCATTTCTTTT	CGTAGAATTTACCGTTGTTCT
Ca_GpSSR00527	Ca_LG_4	Dinucleotide	(AT)11	22	16754699	16754720	Ca4	Dinucleotide	(AT)23	46	4295796	4295841	Intergenic	TCTTCACGCTCCCTACAAAA	TGAGCGTTATAGGGGAGAAAAA
Ca_GpSSR00528	Ca_LG_4	Dinucleotide	(TA)8	16	20289870	20289885	Ca4	Dinucleotide	(TA)33	66	4358440	4358505	Intergenic		
Ca_GpSSR00529	Ca_LG_4	Trinucleotide	(TAA)5	15	20448344	20448358	Ca4	Trinucleotide	(TAA)14	42	4516085	4516126	Intergenic	CTACGTTTCACAATATTTTCGTT	ACCCCTTTTCTCTTTCTTCTA
Ca_GpSSR00530	Ca_LG_4	Dinucleotide	(AT)7	14	20461749	20461762	Ca4	Dinucleotide	(AT)9	18	4521077	4521094	Intergenic	AGAAAATCAGAAGTTGCATT	AAAGTTTGGTGGTGAGTTTTT
Ca_GpSSR00531	Ca_LG_4	Dinucleotide	(AT)7	14	20465320	20465333	Ca4	Dinucleotide	(AT)9	18	4523885	4523902	Intergenic	AGAAAATCAGAAGTTGCATT	AAAGTTTGGTGGTGAGTTTTT
Ca_GpSSR00532	Ca_LG_4	Dinucleotide	(TA)8	16	20468524	20468539	Ca4	Dinucleotide	(TA)6	12	4527174	4527185	Intergenic	AGAAAATCAGAAGTTGCATT	AAAGTTTGGTGGTGAGTTTTT
Ca_GpSSR00533	Ca_LG_4	Dinucleotide	(TA)11	22	20468878	20468899	Ca4	Dinucleotide	(TA)17	34	4527521	4527554	upstream:Ca_05096;	ACGATATCGACATTTTGTCA	CGTACATATGCACCGAAAT
Ca_GpSSR00534	Ca_LG_4	Dinucleotide	(AT)9	18	20533227	20533244	Ca4	Dinucleotide	(AT)6	12	4594656	4594667	mRNA=Ca_05103.1;exon=Ca_05103.1.exon1;5'-UTR=	TGATCGATTGCTCCTCCTTT	ACCCATCATCCCCACTCATA
Ca_GpSSR00535	Ca_LG_4	Trinucleotide	(AAT)40	120	20535959	20536078	Ca4	Trinucleotide	(AAT)18	54	4597377	4597430	Intergenic	AAAATGGTAGGTCATTCATTACT	TACTCTTTCCGCCACAATAA
Ca_GpSSR00536	Ca_LG_4	Dinucleotide	(AG)9	18	20788615	20788632	Ca4	Dinucleotide	(AG)12	24	4842166	4842189	5'-UTR=Ca_05130.1.5'-UTR1;mRNA=Ca_05130.1;exor	TTATCCCGTAAGCCTCGCTA	CCACGTCGGTTCTCTTTGAT
Ca_GpSSR00537	Ca_LG_4	Dinucleotide	(TA)10	20	20822750	20822769	Ca4	Dinucleotide	(TA)8	16	4874271	4874286	gene=Ca_05132;mRNA=Ca_05132.1;intron;	AGAAAATCAGAAGTTGCATT	AAAGTTTGGTGGTGAGTTTTT
Ca_GpSSR00538	Ca_LG_4	Trinucleotide	(ATT)7	21	20834957	20834977	Ca4	Trinucleotide	(ATT)6	18	4891738	4891755	gene=Ca_05132;mRNA=Ca_05132.1;intron;	AAAATAAAAGCTAACGGTCA	TCGAAGTTGTGTCAAACTGTA
Ca_GpSSR00539	Ca_LG_4	Dinucleotide	(AT)12	24	20998932	20998955	Ca4	Dinucleotide	(AT)10	20	5040392	5040411	Intergenic	AGAAAATCAGAAGTTGCATT	AAAGTTTGGTGGTGAGTTTTT
Ca_GpSSR00540	Ca_LG_4	Dinucleotide	(TA)17	34	21147662	21147695	Ca4	Dinucleotide	(TA)21	42	5190023	5190064	Intergenic		
Ca_GpSSR00541	Ca_LG_4	Dinucleotide	(AT)9	18	16136927	16136944	Ca4	Dinucleotide	(AT)11	22	5307746	5307767	Intergenic	AGAAAATCAGAAGTTGCATT	AAAGTTTGGTGGTGAGTTTTT
Ca_GpSSR00542	Ca_LG_4	Dinucleotide	(AT)12	24	16332808	16332831	Ca4	Dinucleotide	(AT)11	22	5499795	5499816	Intergenic	AGAAAATCAGAAGTTGCATT	AAAGTTTGGTGGTGAGTTTTT
Ca_GpSSR00543	Ca_LG_4	Dinucleotide	(TA)22	44	16002374	16002417	Ca4	Dinucleotide	(TA)20	40	5655210	5655249	upstream:Ca_04858;	ATCATTTCCTTCGGTTCATC	TTCTTTTCTTCCTAAAAGGACA
Ca_GpSSR00544	Ca_LG_4	Dinucleotide	(AT)10	20	15835227	15835246	Ca4	Dinucleotide	(AT)9	18	5818842	5818859	Intergenic	AGAAAATCAGAAGTTGCATT	AAAGTTTGGTGGTGAGTTTTT
Ca_GpSSR00545	scaffold02410	Trinucleotide	(TTA)7	21	7931	7951	Ca4	Trinucleotide	(TTA)8	24	7442176	7442199	mRNA=Ca_17394.1;gene=Ca_17394;exon=Ca_17394.	GTAGAAAAGAGAGGACGAACC	AAGACACCATCTTCCCTTTAC
Ca_GpSSR00546	scaffold02410	Trinucleotide	(TAT)43	129	16189	16317	Ca4	Trinucleotide	(TAT)23	69	7450500	7450568	mRNA=Ca_17396.1;gene=Ca_17396;intron;	GTGTAATTTTTGGGGTGATTT	ACAAGACATATACTACGCACCA
Ca_GpSSR00547	scaffold00322	Dinucleotide	(AT)15	30	113976	114005	Ca4	Dinucleotide	(AT)14	28	7584273	7584300	Intergenic	CAAATAACGTTCTCCGTTTTA	CCAATATTGCAAATGTAAAC
Ca_GpSSR00548	scaffold00322	Dinucleotide	(AT)9	18	136154	136171	Ca4	Dinucleotide	(AT)18	36	7603956	7603991	Intergenic	GCCTAAAATCATAAAACCAAAG	ATGCAAGGGTTATTTTATGTC
Ca_GpSSR00549	scaffold01349	Dinucleotide	(TA)9	18	53634	53651	Ca4	Dinucleotide	(TA)10	20	8033857	8033876	Intergenic	CGTGAGTTGACTCATTTTCAT	AAACAGAAATTGTTCCTCACTT
Ca_GpSSR00550	scaffold38781	Dinucleotide	(TA)22	44	1	44	Ca4	Dinucleotide	(TA)8	16	8375076	8375091	Intergenic	AGAAAATCAGAAGTTGCATT	AAAGTTTGGTGGTGAGTTTTT
Ca_GpSSR00551	Ca_LG_4	Dinucleotide	(AT)6	12	12472219	12472230	Ca4	Dinucleotide	(AT)7	14	9130580	9130593	Intergenic	AGATGTACAATCTTTTGAGACAA	GCTGCAAAATGGTACATAGAC
Ca_GpSSR00552	Ca_LG_4	Dinucleotide	(TC)10	20	12035781	12035800	Ca4	Dinucleotide	(TC)11	22	9736708	9736729	gene=Ca_04734;mRNA=Ca_04734.1;intron;	ACCATTTATGAATTACGGTCA	CACACACACATTCACTCTCAA
Ca_GpSSR00553	Ca_LG_4	Dinucleotide	(CA)7	14	12048983	12048996	Ca4	Dinucleotide	(CA)6	12	9750684	9750695	Intergenic		
Ca_GpSSR00554	Ca_LG_4	Dinucleotide	(AT)10	20	12220272	12220291	Ca4	Dinucleotide	(AT)9	18	9923456	9923473	Intergenic	AGAAAATCAGAAGTTGCATT	AAAGTTTGGTGGTGAGTTTTT
Ca_GpSSR00555	Ca_LG_4	Trinucleotide	(TTA)12	36	12261648	12261683	Ca4	Trinucleotide	(TTA)14	42	9966046	9966087	Intergenic		
Ca_GpSSR00556	Ca_LG_4	Dinucleotide	(TA)9	18	12332683	12332700	Ca4	Dinucleotide	(TA)7	14	10047177	10047190	gene=Ca_04760;mRNA=Ca_04760.1;intron;	AGAAAATCAGAAGTTGCATT	AAAGTTTGGTGGTGAGTTTTT
Ca_GpSSR00557	Ca_LG_4	Dinucleotide	(AT)10	20	11754522	11754541	Ca4	Dinucleotide	(AT)22	44	10517113	10517156	Intergenic		
Ca_GpSSR00558	Ca_LG_4	Dinucleotide	(AT)9	18	11805331	11805348	Ca4	Dinucleotide	(AT)10	20	10568477	10568496	Intergenic	AGAAAATCAGAAGTTGCATT	AAAGTTTGGTGGTGAGTTTTT
Ca_GpSSR00559	Ca_LG_4	Dinucleotide	(AT)7	14	16368737	16368750	Ca4	Dinucleotide	(AT)13	26	10729912	10729937	Intergenic	CTTCTTTATTCGCAGTACCAA	GGGCTTCTGTGACACTATAAA
Ca_GpSSR00560	Ca_LG_4	Dinucleotide	(AT)11	22	16377652	16377673	Ca4	Dinucleotide	(AT)18	36	10738319	10738354	Intergenic	GCCTAAAATCATAAAACCAAAG	ATGCAAGGGTTATTTTATGTC
Ca_GpSSR00561	Ca_LG_4	Dinucleotide	(AC)8	16	16525870	16525885	Ca4	Dinucleotide	(AC)9	18	10885133	10885150	Intergenic	TATTGGCAAAAGACTTCATGT	TTCCTCTTTTGGGATACAACT
Ca_GpSSR00562	Ca_LG_4	Dinucleotide	(AT)9	18	16526326	16526343	Ca4	Dinucleotide	(AT)8	16	10885588	10885603	Intergenic	AGAAAATCAGAAGTTGCATT	AAAGTTTGGTGGTGAGTTTTT
Ca_GpSSR00563	Ca_LG_4	Dinucleotide	(AT)9	18	11359990	11360007	Ca4	Dinucleotide	(AT)8	16	11011624	11011639	Intergenic	AGAAAATCAGAAGTTGCATT	AAAGTTTGGTGGTGAGTTTTT
Ca_GpSSR00564	Ca_LG_4	Dinucleotide	(AT)7	14	11365225	11365238	Ca4	Dinucleotide	(AT)6	12	11017174	11017185	Intergenic	AGAAAATCAGAAGTTGCATT	AAAGTTTGGTGGTGAGTTTTT

Ca_GpSSR00565	scaffold109052	Dinucleotide	(TA)21	42	296	337	Ca4	Dinucleotide	(TA)29	58	11148659	11148716	Intergenic		
Ca_GpSSR00566	scaffold00823	Dinucleotide	(TA)12	24	71967	71990	Ca4	Dinucleotide	(TA)13	26	11586183	11586208	Intergenic	CTTCTTTATTCGCAGTACCAA	GGGCTTCTGTGACACTATAAA
Ca_GpSSR00567	scaffold00823	Dinucleotide	(AT)16	32	25440	25471	Ca4	Dinucleotide	(AT)13	26	11642882	11642907	Intergenic	CTTCTTTATTCGCAGTACCAA	GGGCTTCTGTGACACTATAAA
Ca_GpSSR00568	Ca_LG_3	Trinucleotide	(TAT)58	174	3027986	3028159	Ca4	Trinucleotide	(TAT)18	54	11669951	11670004	upstream:Ca_02396;	CATTCCTTCAGCATTTCTTTT	CGTAGAATTTACCGTTGTTCT
Ca_GpSSR00569	Ca_LG_3	Dinucleotide	(AT)16	32	3141497	3141528	Ca4	Dinucleotide	(AT)14	28	11782740	11782767	Intergenic	TTTGCAATCAGTAGGTGAAAT	TAGTTTTCAAGGTGGTAGAGC
Ca_GpSSR00570	Ca_LG_4	Trinucleotide	(TAA)23	69	11059656	11059724	Ca4	Trinucleotide	(TAA)20	60	11973134	11973193	Intergenic		
Ca_GpSSR00571	scaffold00110	Dinucleotide	(TA)9	18	23268	23285	Ca4	Dinucleotide	(TA)8	16	12440404	12440419	Intergenic	GGTGTTTTGGTTTTGATGTAT	TTCCCTTCTACTAAATTTTCCA
Ca_GpSSR00572	scaffold15282	Dinucleotide	(TA)13	26	2550	2575	Ca4	Dinucleotide	(TA)17	34	12451999	12452032	upstream:Ca_20611;	ACGATATCGACATTTTGTCA	CGTACATATGCACCGAAAT
Ca_GpSSR00573	scaffold01289	Dinucleotide	(TA)6	12	31469	31480	Ca4	Dinucleotide	(TA)7	14	12510824	12510837	Intergenic	ACGATGGTCAAAGACACTAAT	CCAAAGAATTATCACATCTTCA
Ca_GpSSR00574	scaffold00690	Trinucleotide	(TAA)17	51	11781	11831	Ca4	Trinucleotide	(TAA)20	60	12613373	12613432	Intergenic	AAGTCAGAAGGTCATACTCAAA	TGGTCTCCAATTATACAGAAAA
Ca_GpSSR00575	scaffold02712	Trinucleotide	(TTA)16	48	5231	5278	Ca4	Trinucleotide	(TTA)15	45	12639363	12639407	Intergenic	TTAAATGGCCCAGACTTAAA	TTTGATGTCGTGTGTGATAGA
Ca_GpSSR00576	scaffold02712	Dinucleotide	(AT)11	22	10747	10768	Ca4	Dinucleotide	(AT)9	18	12644878	12644895	upstream:Ca_17776;	AGAAAATCAGAAGTTGCATT	AAAGTTTGGTGGTGAGTTTTT
Ca_GpSSR00577	scaffold02712	Dinucleotide	(AT)14	28	24774	24801	Ca4	Dinucleotide	(AT)9	18	12659406	12659423	Intergenic	AGAAAATCAGAAGTTGCATT	AAAGTTTGGTGGTGAGTTTTT
Ca_GpSSR00578	Ca_LG_4	Trinucleotide	(ATA)12	36	10552513	10552548	Ca4	Trinucleotide	(ATA)18	54	12681613	12681666	Intergenic	CCAAGCTATAGTGCAATCATCGT	AGGAGGCCTTTGAAAAATCA
Ca_GpSSR00579	Ca_LG_4	Dinucleotide	(TA)7	14	10621458	10621471	Ca4	Dinucleotide	(TA)8	16	12748854	12748869	gene=Ca_04609;mRNA=Ca_04609.1;intron;	AGATGTACAATCTTTTGAGACAA	GCTGCAAAATGGTACATAGAC
Ca_GpSSR00580	Ca_LG_4	Dinucleotide	(AT)15	30	10710704	10710733	Ca4	Dinucleotide	(AT)16	32	12836742	12836773	upstream:Ca_04614;	TCTTATCACTGAATTGACACA	CAAACTTCACCAATACCACAT
Ca_GpSSR00581	Ca_LG_4	Dinucleotide	(AT)7	14	10796590	10796603	Ca4	Dinucleotide	(AT)11	22	12929424	12929445	Intergenic	AGAAAATCAGAAGTTGCATT	AAAGTTTGGTGGTGAGTTTTT
Ca_GpSSR00582	scaffold03073	Dinucleotide	(AT)12	24	106610	106633	Ca4	Dinucleotide	(AT)11	22	12940887	12940908	Intergenic	ATTGTGTAACTGTGCGTCGTA	TAACATTGACCAAAAAGCAGT
Ca_GpSSR00583	Ca_LG_4	Dinucleotide	(TA)14	28	10865571	10865598	Ca4	Dinucleotide	(TA)12	24	12999106	12999129	Intergenic	CTTCTTTATTCGCAGTACCAA	GGGCTTCTGTGACACTATAAA
Ca_GpSSR00584	Ca_LG_4	Dinucleotide	(AT)14	28	10114854	10114881	Ca4	Dinucleotide	(AT)15	30	13467815	13467844	Intergenic	AACGTGTGAGGAATCAAGATA	GGATTCGGAATATCCATTTTA
Ca_GpSSR00585	Ca_LG_4	Dinucleotide	(AT)13	26	9692930	9692955	Ca4	Dinucleotide	(AT)11	22	14000970	14000991	Intergenic	AGAAAATCAGAAGTTGCATT	AAAGTTTGGTGGTGAGTTTTT
Ca_GpSSR00586	scaffold12647	Dinucleotide	(AT)10	20	2183	2202	Ca4	Dinucleotide	(AT)11	22	14487984	14488005	Intergenic	AGAAAATCAGAAGTTGCATT	AAAGTTTGGTGGTGAGTTTTT
Ca_GpSSR00587	scaffold14163	Dinucleotide	(AT)21	42	2894	2935	Ca4	Dinucleotide	(AT)11	22	14755594	14755615	Intergenic	AGAAAATCAGAAGTTGCATT	AAAGTTTGGTGGTGAGTTTTT
Ca_GpSSR00588	Ca_LG_4	Dinucleotide	(AT)21	42	8395710	8395751	Ca4	Dinucleotide	(AT)15	30	14821172	14821201	Intergenic	ATCATTTCCTTCGGTTCATC	TTCTTTTCTTCCTAAAAGGACA
Ca_GpSSR00589	Ca_LG_4	Trinucleotide	(TTA)53	159	8427095	8427253	Ca4	Trinucleotide	(TTA)30	90	14846175	14846264	Intergenic		
Ca_GpSSR00590	scaffold18728	Dinucleotide	(TA)8	16	1821	1836	Ca4	Dinucleotide	(TA)9	18	15019027	15019044	Intergenic	AGAAAATCAGAAGTTGCATT	AAAGTTTGGTGGTGAGTTTTT
Ca_GpSSR00591	Ca_LG_4	Dinucleotide	(TA)8	16	9027366	9027381	Ca4	Dinucleotide	(TA)9	18	15473255	15473272	upstream:Ca_04478;	AGAAAATCAGAAGTTGCATT	AAAGTTTGGTGGTGAGTTTTT
Ca_GpSSR00592	Ca_LG_4	Dinucleotide	(AT)14	28	9100638	9100665	Ca4	Dinucleotide	(AT)11	22	15544198	15544219	Intergenic	AGAAAATCAGAAGTTGCATT	AAAGTTTGGTGGTGAGTTTTT
Ca_GpSSR00593	Ca_LG_4	Dinucleotide	(AT)17	34	8874342	8874375	Ca4	Dinucleotide	(AT)6	12	15924759	15924770	Intergenic	AGAAAATCAGAAGTTGCATT	AAAGTTTGGTGGTGAGTTTTT
Ca_GpSSR00594	Ca_LG_4	Dinucleotide	(TA)10	20	8902940	8902959	Ca4	Dinucleotide	(TA)9	18	15951555	15951572	Intergenic	AGAAAATCAGAAGTTGCATT	AAAGTTTGGTGGTGAGTTTTT
Ca_GpSSR00595	scaffold00935	Trinucleotide	(AAG)5	15	67065	67079	Ca4	Trinucleotide	(AAG)6	18	16077646	16077663	gene=Ca_14517;mRNA=Ca_14517.1;intron;	CCATAAAATGAGAAAGGAAAAG	TGAGCCTACTATTCTCATTGG
Ca_GpSSR00596	scaffold00935	Dinucleotide	(AT)6	12	81588	81599	Ca4	Dinucleotide	(AT)9	18	16086488	16086505	Intergenic	TGACGGCCTTTTAATTTTT	TGCTAGATATGAATGGGTCAT
Ca_GpSSR00597	scaffold00935	Dinucleotide	(TA)10	20	95081	95100	Ca4	Dinucleotide	(TA)14	28	16103326	16103353	Intergenic	TGACGGCCTTTTAATTTTT	TGCTAGATATGAATGGGTCAT
Ca_GpSSR00598	scaffold03454	Dinucleotide	(AG)9	18	10758	10775	Ca4	Dinucleotide	(AG)10	20	16173107	16173126	gene=Ca_18386;mRNA=Ca_18386.1;intron;	AACAACTAGTAGTGGCTTCCA	GGGAAGGTCTATGAGTGTAGG
Ca_GpSSR00599	scaffold00657	Dinucleotide	(TA)12	24	31144	31167	Ca4	Dinucleotide	(TA)24	48	16273661	16273708	Intergenic		
Ca_GpSSR00600	scaffold08911	Dinucleotide	(TA)18	36	1	36	Ca4	Dinucleotide	(TA)16	32	16704731	16704762	Intergenic	ACGATATCGACATTTTGTCA	CGTACATATGCACCGAAAT
Ca_GpSSR00601	Ca_LG_4	Dinucleotide	(AT)9	18	21661216	21661233	Ca4	Dinucleotide	(AT)22	44	17021597	17021640	Intergenic		
Ca_GpSSR00602	Ca_LG_4	Dinucleotide	(AT)14	28	21639011	21639038	Ca4	Dinucleotide	(AT)8	16	17043303	17043318	Intergenic	AGAAAATCAGAAGTTGCATT	AAAGTTTGGTGGTGAGTTTTT
Ca_GpSSR00603	Ca_LG_4	Dinucleotide	(AT)10	20	7748875	7748894	Ca4	Dinucleotide	(AT)26	52	17341313	17341364	Intergenic	AAAACATCATGTGCTGGTGC	CAGCTCATCCTCCTACTGTGG
Ca_GpSSR00604	Ca_LG_4	Dinucleotide	(TA)6	12	7681073	7681084	Ca4	Dinucleotide	(TA)10	20	17395507	17395526	mRNA=Ca_04367.1;gene=Ca_04367;intron;	tCAAAGGTTGAGAACGAGTCC	CAACTTTCATTTTCTCACGAAGA
Ca_GpSSR00605	Ca_LG_4	Trinucleotide	(CAT)5	15	8003332	8003346	Ca4	Trinucleotide	(CAT)6	18	17769896	17769913	gene=Ca_04394;mRNA=Ca_04394.1;exon=Ca_04394.	AGTTATGAATGTGAGGCAATG	AACCCAATAAGAAAAATGGAG
Ca_GpSSR00606	scaffold22996	Dinucleotide	(AT)21	42	1	42	Ca4	Dinucleotide	(AT)6	12	18163274	18163285	upstream:Ca_20829;	AGAAAGGTTTCACTACCCAAG	TCACTAGCCCTATTGTGTGAG
Ca_GpSSR00607	scaffold03151	Dinucleotide	(TA)13	26	6240	6265	Ca4	Dinucleotide	(TA)11	22	18201340	18201361	Intergenic	CTGGACAAGATCAGGTGAGTA	GCAAAAATTTCCTAGATACACC
Ca_GpSSR00608	scaffold00943	Trinucleotide	(ATT)7	21	23716	23736	Ca4	Trinucleotide	(ATT)6	18	18415670	18415687	Intergenic	AAAATAAAAGCTAACGGTCA	TCGAAGTTGTGTCAAACTGTA
Ca_GpSSR00609	scaffold00943	Dinucleotide	(TA)10	20	38367	38386	Ca4	Dinucleotide	(TA)9	18	18430227	18430244	downstream:Ca_14551;	TGAGAGTGAGGTATTTTGGTG	CTCCTGTAGACAAAATTCGTG
Ca_GpSSR00610	Ca_LG_4	Dinucleotide	(TA)7	14	7215697	7215710	Ca4	Dinucleotide	(TA)8	16	18582758	18582773	Intergenic	AGATGTACAATCTTTTGAGACAA	GCTGCAAAATGGTACATAGAC
Ca_GpSSR00611	Ca_LG_7	Trinucleotide	(TTA)9	27	291636	291662	Ca4	Trinucleotide	(TTA)8	24	18821700	18821723	Intergenic		
Ca_GpSSR00612	Ca_LG_7	Trinucleotide	(TTA)17	51	394604	394654	Ca4	Trinucleotide	(TTA)18	54	18919762	18919815	upstream:Ca_07409;		
Ca_GpSSR00613	Ca_LG_7	Dinucleotide	(TA)8	16	450058	450073	Ca4	Dinucleotide	(TA)7	14	18980290	18980303	upstream:Ca_07417;	AGAAAATCAGAAGTTGCATT	AAAGTTTGGTGGTGAGTTTTT
Ca_GpSSR00614	scaffold01605	Dinucleotide	(AT)12	24	60719	60742	Ca4	Dinucleotide	(AT)11	22	19322387	19322408	Intergenic	GGAAGTTAAAATTGTCTGCAA	TGTCATAGAGAGAAGATGAAATG
Ca_GpSSR00615	scaffold03734	Dinucleotide	(AT)7	14	11977	11990	Ca4	Dinucleotide	(AT)10	20	19417048	19417067	Intergenic	AGAGGGATAATTCTGCTTGTC	GGTATACCATCGTTCAGGAG
Ca_GpSSR00616	scaffold00669	Dinucleotide	(AT)20	40	7023	7062	Ca4	Dinucleotide	(AT)18	36	20002735	20002770	Intergenic	GCCTAAAATCATAAAACCAAAG	ATGCAAGGGTTATTTTATGTC
Ca_GpSSR00617	scaffold00669	Trinucleotide	(ATA)29	87	18822	18908	Ca4	Trinucleotide	(ATA)7	21	20009819	20009839	upstream:Ca_13630;	TCTGATAAATCAATCACAAGC	TTACAATTGCAGCTTAAAACG
Ca_GpSSR00618	Ca_LG_4	Dinucleotide	(AT)12	24	5602176	5602199	Ca4	Dinucleotide	(AT)10	20	20464791	20464810	Intergenic	AGAAAATCAGAAGTTGCATT	AAAGTTTGGTGGTGAGTTTTT
Ca_GpSSR00619	scaffold12694	Dinucleotide	(AT)16	32	3363	3394	Ca4	Dinucleotide	(AT)11	22	20884614	20884635	Intergenic	tCATTGTTtCTGAAAATGAAGGAT	CAGAAAATAGAAACGGAAAATTCG
Ca_GpSSR00620	scaffold17969	Dinucleotide	(TA)9	18	1931	1948	Ca4	Dinucleotide	(TA)10	20	21055269	21055288	Intergenic	AGAAAATCAGAAGTTGCATT	AAAGTTTGGTGGTGAGTTTTT
Ca_GpSSR00621	scaffold02573	Trinucleotide	(TAA)10	30	42357	42386	Ca4	Trinucleotide	(TAA)34	102	21113604	21113705	Intergenic	CTTACACGATTTTCTGTGGAC	GCTCAAAAGTTGTAAAACATGA
Ca_GpSSR00622	scaffold00585	Dinucleotide	(AT)8	16	110379	110394	Ca4	Dinucleotide	(AT)9	18	21134199	21134216	Intergenic	AGAAAATCAGAAGTTGCATT	AAAGTTTGGTGGTGAGTTTTT
Ca_GpSSR00623	scaffold00585	Dinucleotide	(TA)10	20	114711	114730	Ca4	Dinucleotide	(TA)11	22	21138579	21138600	Intergenic	AAACATCGACTTTCTGAAACA	CATTTCACATTGAAGTGCTTT
Ca_GpSSR00624	scaffold02396	Trinucleotide	(AAT)12	36	3982	4017	Ca4	Trinucleotide	(AAT)6	18	21257918	21257935	Intergenic	CAACTCCCTCCAATCTTTATT	CATTCCTTGTTGAAATTTCTCT
Ca_GpSSR00625	scaffold00703	Trinucleotide	(TAT)27	81	113509	113589	Ca4	Trinucleotide	(TAT)18	54	21528401	21528454	Intergenic	CATTCCTTCAGCATTTCTTTT	CGTAGAATTTACCGTTGTTCT
Ca_GpSSR00626	scaffold06307	Trinucleotide	(ATA)21	63	5817	5879	Ca4	Trinucleotide	(ATA)19	57	21728404	21728460	Intergenic	TTCAAAGACTGAAAAGAAGAAAG	GTTATGGTTTTTGTTGTTTGG
Ca_GpSSR00627	scaffold00511	Dinucleotide	(TA)12	24	126554	126577	Ca4	Dinucleotide	(TA)9	18	21770744	21770761	gene=Ca_12980;mRNA=Ca_12980.1;intron;	AATTTGCATCTATCTCAAGAACT	CGTTCTTTTAATACTGATTTGAC
Ca_GpSSR00628	scaffold00295	Dinucleotide	(TA)14	28	118053	118080	Ca4	Dinucleotide	(TA)15	30	22280621	22280650	Intergenic	TTTCATCGATCTTGATCACTT	GTGTCAATAAATGGAACAAATG
Ca_GpSSR00629	scaffold01186	Dinucleotide	(AT)11	22	1853	1874	Ca4	Dinucleotide	(AT)8	16	22781195	22781210	Intergenic	TGTAATTTGAGTGCAAACTAGG	TTCTTGTTATCCTCTTGACACA
Ca_GpSSR00630	scaffold01186	Dinucleotide	(TA)7	14	12234	12247	Ca4	Dinucleotide	(TA)6	12	22794522	22794533	Intergenic	TGTAATTTGAGTGCAAACTAGG	TTCTTGTTATCCTCTTGACACA
Ca_GpSSR00631	scaffold06944	Dinucleotide	(AT)16	32	118785	118816	Ca4	Dinucleotide	(AT)8	16	23302563	23302578	Intergenic	AGAAAATCAGAAGTTGCATT	AAAGTTTGGTGGTGAGTTTTT
Ca_GpSSR00632	scaffold55055	Dinucleotide	(AT)7	14	642	655	Ca4	Dinucleotide	(AT)19	38	23722051	23722088	Intergenic		
Ca_GpSSR00633	scaffold00690	Dinucleotide	(TA)18	36	63692	63727	Ca4	Dinucleotide	(TA)10	20	24106872	24106891	Intergenic	AGAAAATCAGAAGTTGCATT	AAAGTTTGGTGGTGAGTTTTT
Ca_GpSSR00634	scaffold00826	Dinucleotide	(AT)12	24	39510	39533	Ca4	Dinucleotide	(AT)9	18	24682088	24682105	Intergenic	AGAAAATCAGAAGTTGCATT	AAAGTTTGGTGGTGAGTTTTT
Ca_GpSSR00635	scaffold00826	Dinucleotide	(AT)12	24	113029	113052	Ca4	Dinucleotide	(AT)14	28	24748228	24748255	Intergenic	TTTGCAATCAGTAGGTGAAAT	TAGTTTTCAAGGTGGTAGAGC
Ca_GpSSR00636	scaffold02554	Dinucleotide	(AT)8	16	13261	13276	Ca4	Dinucleotide	(AT)7	14	25228842	25228855	mRNA=Ca_17597.1;gene=Ca_17597;intron;	GCAAGTTCATGGAGACACTTA	CCAGCTTGGTTAATCATGTAAT
Ca_GpSSR00637	scaffold04250	Dinucleotide	(AT)6	12	17051	17062	Ca4	Dinucleotide	(AT)7	14	25700961	25700974	Intergenic	ATACAAAATGCAACCACTGAG	GAACATCTTGAACTCCAAAAA
Ca_GpSSR00638	scaffold00622	Dinucleotide	(AT)10	20	30973	30992	Ca4	Dinucleotide	(AT)11	22	26366196	26366217	Intergenic	AGAAAATCAGAAGTTGCATT	AAAGTTTGGTGGTGAGTTTTT
Ca_GpSSR00639	scaffold03575	Dinucleotide	(AT)12	24	24937	24960	Ca4	Dinucleotide	(AT)10	20	26908403	26908422	Intergenic	TACAATCTTCACGACTTGACC	ATAGATTACGGCCATTTTTGT
Ca_GpSSR00640	scaffold03575	Trinucleotide	(TTA)42	126	26252	26377	Ca4	Trinucleotide	(TTA)18	54	26909715	26909768	upstream:Ca_18467;		
Ca_GpSSR00641	Ca_LG_4	Trinucleotide	(TAA)37	111	5895990	5896100	Ca4	Trinucleotide	(TAA)6	18	27313204	27313221	Intergenic	TAAATCAATCTACACCGCAAT	ATATGGGACAAGATGATGAGA
Ca_GpSSR00642	scaffold10216	Dinucleotide	(AT)25	50	1081	1130	Ca4	Dinucleotide	(AT)7	14	27363338	27363351	Intergenic	TGTCAACCTTAATGTTTTGGAA	TTgGGCTATTAGTCTTCCTATTCAA
Ca_GpSSR00643	scaffold04448	Dinucleotide	(AT)9	18	5050	5067	Ca4	Dinucleotide	(AT)8	16	27617105	27617120	gene=Ca_18942;mRNA=Ca_18942.1;intron;	TTACAACAATCACTCCGAAAT	GAGCTCGAAAAACTTTTAAGG
Ca_GpSSR00644	scaffold04448	Dinucleotide	(TA)19	38	5777	5814	Ca4	Dinucleotide	(TA)8	16	27617346	27617361	gene=Ca_18942;mRNA=Ca_18942.1;intron;	TTACAACAATCACTCCGAAAT	GAGCTCGAAAAACTTTTAAGG
Ca_GpSSR00645	Ca_LG_4	Dinucleotide	(AT)9	18	5998005	5998022	Ca4	Dinucleotide	(AT)12	24	27796697	27796720	mRNA=Ca_04270.1;gene=Ca_04270;intron;	AGAAAATCAGAAGTTGCATT	AAAGTTTGGTGGTGAGTTTTT

Ca_GpSSR00646	scaffold02387	Dinucleotide	(AT)8	16	40583	40598	Ca4	Dinucleotide	(AT)9	18	28060299	28060316	Intergenic	CAATCTTAATTTCTCCCCAAA	AGGAACCTTGTAACTTTGGTT
Ca_GpSSR00647	scaffold02387	Dinucleotide	(GA)7	14	41885	41898	Ca4	Dinucleotide	(GA)11	22	28061216	28061237	upstream:Ca_17358;	TGTTGGTGTTGAAATCACATA	GTCACTGTAAACAAACGGTGT
Ca_GpSSR00648	Ca_LG_4	Dinucleotide	(AT)13	26	5673428	5673453	Ca4	Dinucleotide	(AT)25	50	28087405	28087454	upstream:Ca_04258;	TTTGCATTGAGATTCATTGTTTTT	AGCGAGGATCGAGACTAAACa
Ca_GpSSR00649	scaffold00167	Dinucleotide	(TA)12	24	203749	203772	Ca4	Dinucleotide	(TA)8	16	28591976	28591991	upstream:Ca_08849;	AGAAAATCAGAAGTTGCATT	AAAGTTTGGTGGTGAGTTTTT
Ca_GpSSR00650	scaffold00167	Dinucleotide	(AT)13	26	206989	207014	Ca4	Dinucleotide	(AT)8	16	28595213	28595228	Intergenic	AGAAAATCAGAAGTTGCATT	AAAGTTTGGTGGTGAGTTTTT
Ca_GpSSR00651	scaffold01139	Dinucleotide	(AT)17	34	8865	8898	Ca4	Dinucleotide	(AT)16	32	28792982	28793013	upstream:Ca_10016;	ACGATATCGACATTTTGTCA	CGTACATATGCACCGAAAT
Ca_GpSSR00652	scaffold02124	Trinucleotide	(ATT)10	30	79909	79938	Ca4	Trinucleotide	(ATT)13	39	28832973	28833011	Intergenic	GCAAGATTTTTCCACTCAAAT	GTACAAAAATTGAAGACCCATAA
Ca_GpSSR00653	scaffold00248	Trinucleotide	(AAT)6	18	22841	22858	Ca4	Trinucleotide	(AAT)7	21	28913642	28913662	Intergenic	TGTTCCTGATATTTTGCTGAT	CGTACTCAAATACGTTAACTTTG
Ca_GpSSR00654	scaffold00248	Trinucleotide	(TAT)11	33	62270	62302	Ca4	Trinucleotide	(TAT)9	27	28939194	28939220	upstream:Ca_09001;	CACCCATGCCTTATAGATACA	TTTAAATTGAGTTCACGTAGTCT
Ca_GpSSR00655	scaffold00248	Dinucleotide	(AT)9	18	160687	160704	Ca4	Dinucleotide	(AT)8	16	29038167	29038182	downstream:Ca_09003;	GACTGTAGAGGAGCACTGATG	GCACACAACAACAAAATTTATC
Ca_GpSSR00656	scaffold04775	Dinucleotide	(AG)9	18	12904	12921	Ca4	Dinucleotide	(AG)8	16	29157884	29157899	upstream:Ca_19091;	TGGAATTTCAAGTTGGTTTT	TATCTCCACTCCTCTCTCCTC
Ca_GpSSR00657	scaffold01858	Trinucleotide	(ATA)20	60	17232	17291	Ca4	Trinucleotide	(ATA)19	57	29358105	29358161	Intergenic		
Ca_GpSSR00658	scaffold00723	Dinucleotide	(AT)7	14	25779	25792	Ca4	Dinucleotide	(AT)8	16	29685561	29685576	mRNA=Ca_09626.1;gene=Ca_09626;intron;	GATCCCTCTATCCCATTTTTA	CTTAAATGTAACATCCCCAAA
Ca_GpSSR00659	scaffold00723	Dinucleotide	(AT)10	20	145661	145680	Ca4	Dinucleotide	(AT)14	28	29767265	29767292	Intergenic	GGGAGAGAAGGAAACTCATT	GATCCAACGAAAATCTGAAAT
Ca_GpSSR00660	scaffold71268	Dinucleotide	(AT)20	40	473	512	Ca4	Dinucleotide	(AT)9	18	29907950	29907967	Intergenic	AGAAAATCAGAAGTTGCATT	AAAGTTTGGTGGTGAGTTTTT
Ca_GpSSR00661	Ca_LG_4	Dinucleotide	(TA)12	24	6167585	6167608	Ca4	Dinucleotide	(TA)6	12	30087569	30087580	Intergenic	AGAAAATCAGAAGTTGCATT	AAAGTTTGGTGGTGAGTTTTT
Ca_GpSSR00662	scaffold02278	Trinucleotide	(ATT)5	15	195789	195803	Ca4	Trinucleotide	(ATT)18	54	30189770	30189823	Intergenic		
Ca_GpSSR00663	scaffold02278	Dinucleotide	(AC)7	14	278841	278854	Ca4	Dinucleotide	(AC)8	16	30330119	30330134	Intergenic	TGGCTAGATTACATAGTGCTTG	AGTCATCGATATTTTCCGATA
Ca_GpSSR00664	scaffold02278	Dinucleotide	(AT)10	20	328881	328900	Ca4	Dinucleotide	(AT)12	24	30372963	30372986	Intergenic	AATGTACATAGTTCATTTGCTG	CATAAATCCAACCTCAAAATG
Ca_GpSSR00665	scaffold02278	Dinucleotide	(AT)7	14	329708	329721	Ca4	Dinucleotide	(AT)9	18	30373373	30373390	Intergenic	AATGTACATAGTTCATTTGCTG	CATAAATCCAACCTCAAAATG
Ca_GpSSR00666	scaffold02278	Trinucleotide	(AAT)28	84	381203	381286	Ca4	Trinucleotide	(AAT)13	39	30412466	30412504	Intergenic	TGTTGACACCTAATTTTATTCG	CGAAAACACTTGTGATTGTTA
Ca_GpSSR00667	scaffold02278	Trinucleotide	(CAG)7	21	384267	384287	Ca4	Trinucleotide	(CAG)6	18	30415086	30415103	gene=Ca_09072;CDS=Ca_09072.1.cds1;mRNA=Ca_09	AATCAAAATCGGTGTTACTCA	CTTTCTTGTTCAGCTCTTTCA
Ca_GpSSR00668	scaffold03864	Trinucleotide	(TTA)6	18	22307	22324	Ca4	Trinucleotide	(TTA)5	15	30500285	30500299	Intergenic	GGAGAAGAAGAAGATGGAATG	CAATCTTGGTCCTCCTATTTT
Ca_GpSSR00669	Ca_LG_4	Dinucleotide	(TA)11	22	6374008	6374029	Ca4	Dinucleotide	(TA)9	18	30776004	30776021	Intergenic	AGAAAATCAGAAGTTGCATT	AAAGTTTGGTGGTGAGTTTTT
Ca_GpSSR00670	Ca_LG_4	Dinucleotide	(AT)15	30	6388898	6388927	Ca4	Dinucleotide	(AT)18	36	30791928	30791963	Intergenic	TCTTATCACTGAATTGACACA	CAAACTTCACCAATACCACAT
Ca_GpSSR00671	scaffold58838	Dinucleotide	(AT)18	36	581	616	Ca4	Dinucleotide	(AT)12	24	31259146	31259169	Intergenic	AGAAAATCAGAAGTTGCATT	AAAGTTTGGTGGTGAGTTTTT
Ca_GpSSR00672	scaffold16708	Dinucleotide	(AT)8	16	126	141	Ca4	Dinucleotide	(AT)25	50	31260052	31260101	Intergenic	CcACCAAAAtCCGTCAACTT	TGTTCCATCATAtTTTcAACCG
Ca_GpSSR00673	scaffold05983	Dinucleotide	(GA)53	106	5704	5809	Ca4	Dinucleotide	(GA)15	30	31448290	31448319	Intergenic	TAGAGGAAGCGTCGAATTTAC	TTCTCCCCCACTCTATTTTAC
Ca_GpSSR00674	scaffold00440	Trinucleotide	(TTA)6	18	164648	164665	Ca4	Trinucleotide	(TTA)5	15	31547980	31547994	Intergenic	GGAGAAGAAGAAGATGGAATG	CAATCTTGGTCCTCCTATTTT
Ca_GpSSR00675	scaffold01082	Dinucleotide	(TA)17	34	44663	44696	Ca4	Dinucleotide	(TA)16	32	31633215	31633246	Intergenic	ACGATATCGACATTTTGTCA	CGTACATATGCACCGAAAT
Ca_GpSSR00676	scaffold01082	Dinucleotide	(TA)8	16	58681	58696	Ca4	Dinucleotide	(TA)7	14	31647089	31647102	gene=Ca_14915;mRNA=Ca_14915.1;intron;	GGAATTGTGACAAGAATCGTA	GATTTGAAAGATTTTTCCTCTCT
Ca_GpSSR00677	scaffold00936	Dinucleotide	(AT)15	30	64220	64249	Ca4	Dinucleotide	(AT)10	20	31856392	31856411	Intergenic	AGAAAATCAGAAGTTGCATT	AAAGTTTGGTGGTGAGTTTTT
Ca_GpSSR00678	scaffold00876	Dinucleotide	(GT)8	16	51889	51904	Ca4	Dinucleotide	(GT)7	14	32138549	32138562	Intergenic	TAGGGTGGATGTTAATCATTG	TCCTACTTCATTTTCAACCAT
Ca_GpSSR00679	scaffold00510	Dinucleotide	(TA)12	24	47250	47273	Ca4	Dinucleotide	(TA)10	20	32548489	32548508	Intergenic	TATTGCAAGAGCTTCGTATTC	TGATGCTCATCATTTAGTCTTT
Ca_GpSSR00680	scaffold06455	Tetranucleotide	(TATT)6	24	1544	1567	Ca4	Tetranucleotide	(TATT)5	20	32587040	32587059	Intergenic	CTTTGGTGTCTTCAAATATGC	AAAGATGATGTGTTGGTGTGT
Ca_GpSSR00681	scaffold01385	Trinucleotide	(TTC)9	27	3678	3704	Ca4	Trinucleotide	(TTC)7	21	32654906	32654926	Intergenic	TGACTTCCAAAAGAATGAAGA	CAGAAGCAGAAAGAGTGAAAA
Ca_GpSSR00682	scaffold11568	Trinucleotide	(TTA)5	15	401	415	Ca4	Trinucleotide	(TTA)7	21	32663517	32663537	Intergenic	TGTTGGTTTTGTTTTCTTCTC	AATAAAATGTCAAGCCCAAA
Ca_GpSSR00683	scaffold01385	Dinucleotide	(TA)10	20	43293	43312	Ca4	Dinucleotide	(TA)7	14	32707242	32707255	Intergenic	CACTTTGTAATAAAACTCATTCC	GCATGATGAATACGCAATCTA
Ca_GpSSR00684	scaffold03736	Dinucleotide	(TA)8	16	7571	7586	Ca4	Dinucleotide	(TA)6	12	32819274	32819285	Intergenic	AAAAATAAGTGGGAGAGAGAA	TATTGACTTCAATGTCCCTTC
Ca_GpSSR00685	Ca_LG_4	Dinucleotide	(TA)18	36	6630434	6630469	Ca4	Dinucleotide	(TA)20	40	34073713	34073752	Intergenic		
Ca_GpSSR00686	scaffold01247	Dinucleotide	(CT)7	14	4643	4656	Ca4	Dinucleotide	(CT)9	18	34309140	34309157	5'-UTR=Ca_10102.1.5'-UTR1;gene=Ca_10102;mRNA=	ACATCCGGTTACCCCTCTCT	CGTAACGGTTTTCTTCACGC
Ca_GpSSR00687	scaffold03261	Dinucleotide	(TA)6	12	13821	13832	Ca4	Dinucleotide	(TA)8	16	34921996	34922011	Intergenic	AAACAAAAGTCATTGTTATCCTC	CAAAAATGTTAGACTCGGATT
Ca_GpSSR00688	scaffold09501	Dinucleotide	(AT)12	24	2887	2910	Ca4	Dinucleotide	(AT)10	20	35445773	35445792	Intergenic	CAACATAGTTCGGATTTCTCC	CGAATCCGTTTCACAAGTAAT
Ca_GpSSR00689	scaffold02835	Dinucleotide	(AT)6	12	119574	119585	Ca4	Dinucleotide	(AT)8	16	35764869	35764884	Intergenic	GCTTGTCCAAATATGTCCTTA	AAAAGTCAATGCTCTGAAGAA
Ca_GpSSR00690	Ca_LG_7	Dinucleotide	(AG)7	14	1753446	1753459	Ca4	Dinucleotide	(AG)8	16	36458303	36458318	Intergenic	CATATCATATCATGCCCTGAC	TTTGTACAAGTGGGTTTTCTC
Ca_GpSSR00691	Ca_LG_7	Dinucleotide	(AC)9	18	1755599	1755616	Ca4	Dinucleotide	(AC)8	16	36460460	36460475	gene=Ca_07502;mRNA=Ca_07502.2;intron;	ACTTCAAAATAGAGGCCATTC	GGTGTGTCTCAATTCAGTGTT
Ca_GpSSR00692	Ca_LG_7	Trinucleotide	(ATT)6	18	1848134	1848151	Ca4	Trinucleotide	(ATT)5	15	36564657	36564671	upstream:Ca_07508;		
Ca_GpSSR00693	Ca_LG_4	Dinucleotide	(TA)6	12	4863426	4863437	Ca4	Dinucleotide	(TA)23	46	36627420	36627465	Intergenic	AGATGTACAATCTTTTGAGACAA	GCTGCAAAATGGTACATAGAC
Ca_GpSSR00694	Ca_LG_4	Dinucleotide	(AT)7	14	4881993	4882006	Ca4	Dinucleotide	(AT)6	12	36642951	36642962	gene=Ca_04220;mRNA=Ca_04220.1;intron;	AGAAAATCAGAAGTTGCATT	AAAGTTTGGTGGTGAGTTTTT
Ca_GpSSR00695	Ca_LG_4	Dinucleotide	(AT)9	18	4916967	4916984	Ca4	Dinucleotide	(AT)11	22	36680990	36681011	Intergenic	AGAAAATCAGAAGTTGCATT	AAAGTTTGGTGGTGAGTTTTT
Ca_GpSSR00696	Ca_LG_4	Dinucleotide	(AT)22	44	4939195	4939238	Ca4	Dinucleotide	(AT)23	46	36712037	36712082	Intergenic	ATCATTTCCTTCGGTTCATC	TTCTTTTCTTCCTAAAAGGACA
Ca_GpSSR00697	scaffold00495	Trinucleotide	(CTT)6	18	41233	41250	Ca4	Trinucleotide	(CTT)7	21	36807815	36807835	5'-UTR=Ca_12890.1.5'-UTR1;exon=Ca_12890.1.exon1	TTTCTTTGAAAGTTTCCCACT	CAAAAATTCCCTAGGAACAG
Ca_GpSSR00698	scaffold00495	Trinucleotide	(CAC)8	24	76627	76650	Ca4	Trinucleotide	(CAC)7	21	36842783	36842803	Intergenic	CTAAAGAATGGAATTGGGATT	CTCGTTTGTTTGCTCTATTGT
Ca_GpSSR00699	scaffold00495	Dinucleotide	(AT)9	18	121129	121146	Ca4	Dinucleotide	(AT)12	24	36887863	36887886	gene=Ca_12895;mRNA=Ca_12895.1;intron;	GACTAGTCCAACATTTGACTTTTCTT	TCACATAAGTGGTCCTGCCA
Ca_GpSSR00700	scaffold17208	Dinucleotide	(AT)13	26	2049	2074	Ca4	Dinucleotide	(AT)8	16	36969368	36969383	Intergenic	AGAAAATCAGAAGTTGCATT	AAAGTTTGGTGGTGAGTTTTT
Ca_GpSSR00701	scaffold00331	Dinucleotide	(TC)8	16	18495	18510	Ca4	Dinucleotide	(TC)7	14	37259902	37259915	mRNA=Ca_12084.1;gene=Ca_12084;intron;	TTGGACCTATAGCTTTCTTCA	TGCGTGATATGAGATGTGTTA
Ca_GpSSR00702	scaffold00331	Dinucleotide	(AG)7	14	40845	40858	Ca4	Dinucleotide	(AG)8	16	37280085	37280100	exon=Ca_12084.1.exon1;5'-UTR=Ca_12084.1.5'-UTR1	CTAACCAGTGTCGAAACAAAC	TCTCTAAATCCACCACTTTCA
Ca_GpSSR00703	scaffold02277	Dinucleotide	(TA)6	12	32178	32189	Ca4	Dinucleotide	(TA)9	18	37494002	37494019	Intergenic	AGAAAATCAGAAGTTGCATT	AAAGTTTGGTGGTGAGTTTTT
Ca_GpSSR00704	scaffold01413	Dinucleotide	(AT)6	12	13091	13102	Ca4	Dinucleotide	(AT)7	14	37581783	37581796	gene=Ca_10228;mRNA=Ca_10228.1;intron;	TGAAATAAAAGCAAGAAGTGG	ATCCTTTACCATTTCATCTGG
Ca_GpSSR00705	scaffold01413	Dinucleotide	(CT)8	16	23717	23732	Ca4	Dinucleotide	(CT)7	14	37608002	37608015	exon=Ca_10229.1.exon1;gene=Ca_10229;mRNA=Ca_	TTCTCCTCTTTCATTTCACAA	AAGAGGACTGTGCCTAGTTTC
Ca_GpSSR00706	scaffold01006	Dinucleotide	(AT)11	22	34128	34149	Ca4	Dinucleotide	(AT)14	28	37789660	37789687	Intergenic	TGGTGGACACTCTAAGACAAT	CATAGCAAAATTGAACAGAATG
Ca_GpSSR00707	scaffold01006	Dinucleotide	(TA)6	12	44287	44298	Ca4	Dinucleotide	(TA)24	48	37799177	37799224	Intergenic	AAATTTTCACATGACTTGGTG	GAAAAATATTCATCGAACACG
Ca_GpSSR00708	scaffold00491	Dinucleotide	(TA)9	18	146082	146099	Ca4	Dinucleotide	(TA)8	16	37817076	37817091	mRNA=Ca_12868.1;gene=Ca_12868;intron;	AGAAAATCAGAAGTTGCATT	AAAGTTTGGTGGTGAGTTTTT
Ca_GpSSR00709	scaffold00423	Dinucleotide	(TA)7	14	24842	24855	Ca4	Dinucleotide	(TA)12	24	38232543	38232566	Intergenic	CAACGACAAATCGACAATGG	CGAATTCGACAAAACTACAATAAATG
Ca_GpSSR00710	scaffold01706	Dinucleotide	(TA)13	26	51498	51523	Ca4	Dinucleotide	(TA)14	28	38376340	38376367	Intergenic	CGCTACTTGTTTACTCCAAAA	TAATCATAAATGGGTTTCACG
Ca_GpSSR00711	scaffold01706	Dinucleotide	(TA)7	14	61058	61071	Ca4	Dinucleotide	(TA)6	12	38385903	38385914	Intergenic	GAATTGCTTAGGTTTTTAGCC	CATTGAATTCGAGAAACACTC
Ca_GpSSR00712	scaffold00930	Dinucleotide	(TA)7	14	26824	26837	Ca4	Dinucleotide	(TA)6	12	38473893	38473904	gene=Ca_14499;mRNA=Ca_14499.1;intron;	TGGTATGGCTGCTATATGAAT	TGGGCCTCTAAGTCTTTTATT
Ca_GpSSR00713	scaffold00930	Dinucleotide	(TA)7	14	53855	53868	Ca4	Dinucleotide	(TA)6	12	38503645	38503656	mRNA=Ca_14501.1;gene=Ca_14501;intron;	TGGTATGGCTGCTATATGAAT	TGGGCCTCTAAGTCTTTTATT
Ca_GpSSR00714	scaffold15561	Dinucleotide	(AT)6	12	683	694	Ca4	Dinucleotide	(AT)12	24	38524592	38524615	Intergenic	CGTTCATTAACAAAACAATCAAC	TGAACCTAATGTTCTTTAACTCA
Ca_GpSSR00715	Ca_LG_4	Dinucleotide	(TA)16	32	4607604	4607635	Ca4	Dinucleotide	(TA)11	22	38674437	38674458	gene=Ca_04202;mRNA=Ca_04202.1;intron;	CACCAAGCCACCcTTATGAC	TGGGATGACTGCTATTCCAA
Ca_GpSSR00716	Ca_LG_4	Dinucleotide	(AT)10	20	4611897	4611916	Ca4	Dinucleotide	(AT)8	16	38678558	38678573	gene=Ca_04202;mRNA=Ca_04202.1;intron;	AGAAAATCAGAAGTTGCATT	AAAGTTTGGTGGTGAGTTTTT
Ca_GpSSR00717	Ca_LG_4	Trinucleotide	(TGC)6	18	4613667	4613684	Ca4	Trinucleotide	(TGC)5	15	38680325	38680339	gene=Ca_04202;mRNA=Ca_04202.1;exon=Ca_04202.	CACATGTCCAATACCACCTAT	ATCAACAGCATTCTCAACAAC
Ca_GpSSR00718	Ca_LG_4	Tetranucleotide	(TATT)5	20	4645602	4645621	Ca4	Tetranucleotide	(TATT)6	24	38712506	38712529	upstream:Ca_04203;	TAATTTTGTGGATCTGGCTTA	GGTTTGTGAGTCATGGTTTTA
Ca_GpSSR00719	Ca_LG_4	Dinucleotide	(AT)8	16	4697060	4697075	Ca4	Dinucleotide	(AT)7	14	38766115	38766128	Intergenic	AGAAAATCAGAAGTTGCATT	AAAGTTTGGTGGTGAGTTTTT
Ca_GpSSR00720	Ca_LG_4	Dinucleotide	(TA)8	16	4703375	4703390	Ca4	Dinucleotide	(TA)10	20	38772350	38772369	upstream:Ca_04206;	AGAAAATCAGAAGTTGCATT	AAAGTTTGGTGGTGAGTTTTT
Ca_GpSSR00721	Ca_LG_4	Trinucleotide	(AAT)17	51	4750412	4750462	Ca4	Trinucleotide	(AAT)5	15	38818820	38818834	mRNA=Ca_04209.1;gene=Ca_04209;intron;		
Ca_GpSSR00722	Ca_LG_4	Trinucleotide	(ATT)8	24	4764707	4764730	Ca4	Trinucleotide	(ATT)15	45	38829589	38829633	upstream:Ca_04210;		
Ca_GpSSR00723	scaffold01372	Dinucleotide	(TA)12	24	29391	29414	Ca4	Dinucleotide	(TA)9	18	39116041	39116058	upstream:Ca_15623;	GCAAACTATAATTCAAAAGCAC	TCTTGTTTGTTCAGGAAAGAG
Ca_GpSSR00724	scaffold03500	Dinucleotide	(AT)9	18	25134	25151	Ca4	Dinucleotide	(AT)25	50	39270659	39270708	upstream:Ca_18418;	ATAATACCAGCCCAAATCTGT	CGTCAATGTTGAACTTCTTTT
Ca_GpSSR00725	scaffold00364	Dinucleotide	(TA)13	26	11085	11110	Ca4	Dinucleotide	(TA)18	36	39775286	39775321	Intergenic		
Ca_GpSSR00726	scaffold17537	Dinucleotide	(TA)16	32	1978	2009	Ca4	Dinucleotide	(TA)10	20	40608786	40608805	Intergenic	AGAAAATCAGAAGTTGCATT	AAAGTTTGGTGGTGAGTTTTT

Ca_GpSSR00727	scaffold01851	Dinucleotide	(AT)6	12	40550	40561	Ca4	Dinucleotide	(AT)7	14	40614743	40614756	Intergenic	ATGGCGACTTCTTGAAATAAT	ACATTGTCCAAATGTTACAGG
Ca_GpSSR00728	Ca_LG_4	Dinucleotide	(AT)12	24	3824144	3824167	Ca4	Dinucleotide	(AT)21	42	40760314	40760355	Intergenic	TgCCcTTTtAaGgTTtGTTTG	TGATTTCTCATTTTAAACGGtTTTT
Ca_GpSSR00729	Ca_LG_4	Dinucleotide	(AT)19	38	3825049	3825086	Ca4	Dinucleotide	(AT)11	22	40761337	40761358	Intergenic	AGAAAATCAGAAGTTGCATT	AAAGTTTGGTGGTGAGTTTTT
Ca_GpSSR00730	Ca_LG_4	Dinucleotide	(TA)20	40	3853392	3853431	Ca4	Dinucleotide	(TA)8	16	40789270	40789285	Intergenic	AGAAAATCAGAAGTTGCATT	AAAGTTTGGTGGTGAGTTTTT
Ca_GpSSR00731	Ca_LG_4	Trinucleotide	(TTA)7	21	3902260	3902280	Ca4	Trinucleotide	(TTA)13	39	40838056	40838094	upstream:Ca_04151;		
Ca_GpSSR00732	scaffold01081	Pentanucleotide	(TAATA)7	35	63544	63578	Ca4	Pentanucleotide	(TAATA)44	220	40920226	40920445	mRNA=Ca_14912.1;gene=Ca_14912;intron;	TGATCATAGGAAATAACTCAAGA	GTTGCTTTTCCATCCTTTTTA
Ca_GpSSR00733	Ca_LG_4	Trinucleotide	(TTA)15	45	7331223	7331267	Ca4	Trinucleotide	(TTA)23	69	41261011	41261079	Intergenic		
Ca_GpSSR00734	scaffold05070	Trinucleotide	(ATT)27	81	10535	10615	Ca4	Trinucleotide	(ATT)20	60	41654606	41654665	Intergenic		
Ca_GpSSR00735	Ca_LG_4	Dinucleotide	(AT)8	16	3406867	3406882	Ca4	Dinucleotide	(AT)9	18	41708778	41708795	Intergenic	AGAAAATCAGAAGTTGCATT	AAAGTTTGGTGGTGAGTTTTT
Ca_GpSSR00736	Ca_LG_4	Dinucleotide	(AT)12	24	3370477	3370500	Ca4	Dinucleotide	(AT)11	22	41748174	41748195	upstream:Ca_04118	AGAAAATCAGAAGTTGCATT	AAAGTTTGGTGGTGAGTTTTT
Ca_GpSSR00737	scaffold43344	Dinucleotide	(AT)17	34	1	34	Ca4	Dinucleotide	(AT)18	36	43419688	43419723	Intergenic	GCCTAAAATCATAAAACCAAAG	ATGCAAGGGTTATTTTATGTC
Ca_GpSSR00738	scaffold03544	Dinucleotide	(TA)11	22	18214	18235	Ca4	Dinucleotide	(TA)7	14	43437682	43437695	upstream:Ca_18440;	CCAAAAGGTTACGTTCAAAAAGA	GCTTTTTGCATTTGTTTCCAA
Ca_GpSSR00739	scaffold00953	Dinucleotide	(AT)22	44	58999	59042	Ca4	Dinucleotide	(AT)20	40	43678585	43678624	downstream:Ca_14603;		
Ca_GpSSR00740	Ca_LG_4	Dinucleotide	(AT)17	34	944822	944855	Ca4	Dinucleotide	(AT)14	28	43895698	43895725	Intergenic	TTTGCAATCAGTAGGTGAAAT	TAGTTTTCAAGGTGGTAGAGC
Ca_GpSSR00741	Ca_LG_4	Dinucleotide	(AT)17	34	1024068	1024101	Ca4	Dinucleotide	(AT)18	36	43981124	43981159	upstream:Ca_03955;	GCCTAAAATCATAAAACCAAAG	ATGCAAGGGTTATTTTATGTC
Ca_GpSSR00742	Ca_LG_4	Dinucleotide	(TA)15	30	1057355	1057384	Ca4	Dinucleotide	(TA)11	22	44015358	44015379	upstream:Ca_03957;	AGAAAATCAGAAGTTGCATT	AAAGTTTGGTGGTGAGTTTTT
Ca_GpSSR00743	Ca_LG_4	Trinucleotide	(TTA)7	21	2871745	2871765	Ca4	Trinucleotide	(TTA)8	24	44057616	44057639	mRNA=Ca_04080.2;gene=Ca_04080;intron;		
Ca_GpSSR00744	Ca_LG_4	Dinucleotide	(AT)10	20	2420679	2420698	Ca4	Dinucleotide	(AT)11	22	45196422	45196443	Intergenic	AGAAAATCAGAAGTTGCATT	AAAGTTTGGTGGTGAGTTTTT
Ca_GpSSR00745	scaffold20485	Dinucleotide	(TA)18	36	1	36	Ca4	Dinucleotide	(TA)16	32	45441613	45441644	Intergenic	ACGATATCGACATTTTGTCA	CGTACATATGCACCGAAAT
Ca_GpSSR00746	scaffold00710	Dinucleotide	(TA)23	46	89185	89230	Ca4	Dinucleotide	(TA)9	18	45581584	45581601	Intergenic	AGAAAATCAGAAGTTGCATT	AAAGTTTGGTGGTGAGTTTTT
Ca_GpSSR00747	scaffold01013	Dinucleotide	(TA)15	30	57191	57220	Ca4	Dinucleotide	(TA)16	32	45713859	45713890	Intergenic	TGTATGCTTACCATAAGTTTCTT	TTGTTAGAAAGTTCTTGTTTGGT
Ca_GpSSR00748	scaffold04580	Dinucleotide	(TA)11	22	17663	17684	Ca4	Dinucleotide	(TA)12	24	45740249	45740272	Intergenic	TCATAACCGACCCATGTATAA	GAATGATGTTTCCGTCTAATG
Ca_GpSSR00749	scaffold00259	Dinucleotide	(AT)16	32	189743	189774	Ca4	Dinucleotide	(AT)12	24	45811549	45811572	Intergenic	AGACGTCCTTAGGGgATTTTT	TTTAAGGaAGTCGCCTTTGC
Ca_GpSSR00750	scaffold01793	Dinucleotide	(AT)8	16	5485	5500	Ca4	Dinucleotide	(AT)18	36	46377600	46377635	upstream:Ca_16441;	GCCTAAAATCATAAAACCAAAG	ATGCAAGGGTTATTTTATGTC
Ca_GpSSR00751	scaffold27754	Dinucleotide	(TA)21	42	4	45	Ca4	Dinucleotide	(TA)22	44	46383956	46383999	Intergenic		
Ca_GpSSR00752	scaffold08083	Dinucleotide	(AT)12	24	4111	4134	Ca4	Dinucleotide	(AT)15	30	46625203	46625232	Intergenic	AACGTGTGAGGAATCAAGATA	GGATTCGGAATATCCATTTTA
Ca_GpSSR00753	scaffold03157	Dinucleotide	(AT)11	22	22132	22153	Ca4	Dinucleotide	(AT)20	40	46805382	46805421	Intergenic		
Ca_GpSSR00754	Ca_LG_4	Dinucleotide	(AT)10	20	11218999	11219018	Ca4	Dinucleotide	(AT)11	22	47306164	47306185	mRNA=Ca_04662.1;gene=Ca_04662;intron;	AGAAAATCAGAAGTTGCATT	AAAGTTTGGTGGTGAGTTTTT
Ca_GpSSR00755	scaffold48034	Dinucleotide	(AT)16	32	1	32	Ca4	Dinucleotide	(AT)24	48	47891453	47891500	Intergenic		
Ca_GpSSR00756	scaffold00343	Dinucleotide	(AT)22	44	148582	148625	Ca4	Dinucleotide	(AT)23	46	47891943	47891988	upstream:Ca_12188;		
Ca_GpSSR00757	scaffold02287	Dinucleotide	(AT)15	30	45452	45481	Ca4	Dinucleotide	(AT)16	32	48042401	48042432	mRNA=Ca_17224.1;gene=Ca_17224;intron;	ACGATATCGACATTTTGTCA	CGTACATATGCACCGAAAT
Ca_GpSSR00758	Ca_LG_4	Dinucleotide	(AT)17	34	4770	4803	Ca4	Dinucleotide	(AT)24	48	48120529	48120576	Intergenic		
Ca_GpSSR00759	Ca_LG_4	Trinucleotide	(ATT)18	54	81060	81113	Ca4	Trinucleotide	(ATT)17	51	48197647	48197697	Intergenic		
Ca_GpSSR00760	Ca_LG_4	Dinucleotide	(AT)11	22	193470	193491	Ca4	Dinucleotide	(AT)12	24	48317504	48317527	Intergenic	AGAAAATCAGAAGTTGCATT	AAAGTTTGGTGGTGAGTTTTT
Ca_GpSSR00761	scaffold09025	Dinucleotide	(AT)14	28	12	39	Ca4	Dinucleotide	(AT)16	32	48660045	48660076	Intergenic	ACGATATCGACATTTTGTCA	CGTACATATGCACCGAAAT
Ca_GpSSR00762	scaffold00351	Hexanucleotide	(ATAGTA)19	114	127986	128099	Ca4	Hexanucleotide	(ATAGTA)11	66	49097626	49097691	Intergenic	AAAAGTATGAGCAACGCACT	TCTAAAAAGACTACATCCTAACA
Ca_GpSSR00763	scaffold00351	Dinucleotide	(TA)16	32	166714	166745	Ca4	Dinucleotide	(TA)20	40	49142217	49142256	Intergenic		
Ca_GpSSR00764	Ca_LG_8	Dinucleotide	(AT)14	28	231831	231858	Ca5	Dinucleotide	(AT)15	30	105148	105177	gene=Ca_07993;mRNA=Ca_07993.1;intron;	TTTACCGGTGTAAATGGAC	TGGTATCATGTGTTTGATGTG
Ca_GpSSR00765	Ca_LG_8	Dinucleotide	(AG)11	22	306000	306021	Ca5	Dinucleotide	(AG)36	72	182413	182484	upstream:Ca_08001;	GGTGAAAATCAAAAGTAGGAGA	GGTATTCCTTCCATCCTCTT
Ca_GpSSR00766	scaffold04668	Dinucleotide	(TA)12	24	765	788	Ca5	Dinucleotide	(TA)13	26	864810	864835	Intergenic	TGGAAACAATCTCCAATCTTA	GTTGGAGTCATGTTTTTCATC
Ca_GpSSR00767	scaffold36618	Trinucleotide	(TAA)15	45	302	346	Ca5	Trinucleotide	(TAA)11	33	927604	927636	Intergenic	CATTTTACCTTATTAGGAGTTGA	AGACATTTTTAACCGTTCCTT
Ca_GpSSR00768	scaffold02375	Dinucleotide	(AT)11	22	22802	22823	Ca5	Dinucleotide	(AT)10	20	1239626	1239645	Intergenic	GTTGATTTTGAATAGGTGTCG	TTAAAATAAAGGACCAACTCG
Ca_GpSSR00769	scaffold02361	Dinucleotide	(AT)15	30	2442	2471	Ca5	Dinucleotide	(AT)7	14	1305258	1305271	Intergenic	CATGCAACCTTCTTTTCAA	AGACATGGTAGATTAAAAGTGTG
Ca_GpSSR00770	scaffold05350	Dinucleotide	(AT)22	44	8401	8444	Ca5	Dinucleotide	(AT)9	18	1433021	1433038	Intergenic	CATGCAACCTTCTTTTCAA	AGACATGGTAGATTAAAAGTGTG
Ca_GpSSR00771	scaffold19329	Trinucleotide	(TTA)7	21	730	750	Ca5	Trinucleotide	(TTA)6	18	2101144	2101161	Intergenic	CGAAAACACTTGTGATTGTTA	ACACCTAATTTTGTCCGATTT
Ca_GpSSR00772	scaffold71205	Dinucleotide	(TA)13	26	7	32	Ca5	Dinucleotide	(TA)14	28	2577648	2577675	Intergenic	GTCCCAAAGCAATTTATTATC	CACTGCCACCGTATTATTAAA
Ca_GpSSR00773	scaffold00741	Dinucleotide	(TA)18	36	33647	33682	Ca5	Dinucleotide	(TA)16	32	3103863	3103894	Intergenic	GTCCCAAAGCAATTTATTATC	CACTGCCACCGTATTATTAAA
Ca_GpSSR00774	scaffold02355	Dinucleotide	(AT)11	22	35422	35443	Ca5	Dinucleotide	(AT)10	20	3235898	3235917	Intergenic	TTGGTGGTGAACTAGGATAAC	ATACAAGAACGAGGAACACAA
Ca_GpSSR00775	scaffold02571	Dinucleotide	(TA)21	42	29718	29759	Ca5	Dinucleotide	(TA)6	12	3875374	3875385	Intergenic	CATGCAACCTTCTTTTCAA	AGACATGGTAGATTAAAAGTGTG
Ca_GpSSR00776	scaffold01710	Trinucleotide	(TAT)5	15	26523	26537	Ca5	Trinucleotide	(TAT)6	18	4654654	4654671	Intergenic		
Ca_GpSSR00777	scaffold55125	Trinucleotide	(AAT)23	69	501	569	Ca5	Trinucleotide	(AAT)20	60	4860801	4860860	Intergenic		
Ca_GpSSR00778	scaffold55125	Trinucleotide	(TAA)8	24	633	656	Ca5	Trinucleotide	(TAA)7	21	4860923	4860943	Intergenic	TGGAGTGAAGAGGTAGAGACA	TTAAAATCTGAGGCCTTTTT
Ca_GpSSR00779	scaffold00673	Dinucleotide	(AT)16	32	87725	87756	Ca5	Dinucleotide	(AT)23	46	5043272	5043317	Intergenic	GTCCCAAAGCAATTTATTATC	CACTGCCACCGTATTATTAAA
Ca_GpSSR00780	scaffold00673	Dinucleotide	(TA)15	30	88088	88117	Ca5	Dinucleotide	(TA)20	40	5043499	5043538	Intergenic	ACATCATCTGGTGGACAAATA	ACATGCTAATCTACTGCTTGTT
Ca_GpSSR00781	Ca_LG_8	Dinucleotide	(TA)18	36	10358461	10358496	Ca5	Dinucleotide	(TA)16	32	5222323	5222354	Intergenic	GTCCCAAAGCAATTTATTATC	CACTGCCACCGTATTATTAAA
Ca_GpSSR00782	scaffold05608	Dinucleotide	(TA)18	36	2634	2669	Ca5	Dinucleotide	(TA)17	34	5225523	5225556	Intergenic	GTCCCAAAGCAATTTATTATC	CACTGCCACCGTATTATTAAA
Ca_GpSSR00783	scaffold01058	Dinucleotide	(AT)9	18	76518	76535	Ca5	Dinucleotide	(AT)20	40	5394906	5394945	Intergenic	GTCCCAAAGCAATTTATTATC	CACTGCCACCGTATTATTAAA
Ca_GpSSR00784	scaffold01912	Dinucleotide	(TA)22	44	46262	46305	Ca5	Dinucleotide	(TA)7	14	5804707	5804720	Intergenic	CATGCAACCTTCTTTTCAA	AGACATGGTAGATTAAAAGTGTG
Ca_GpSSR00785	scaffold03269	Dinucleotide	(AT)23	46	27028	27073	Ca5	Dinucleotide	(AT)6	12	5888599	5888610	Intergenic	GGGACCTATTTACACACTACG	TCACACGAGTTGACTTAACAA
Ca_GpSSR00786	scaffold03730	Trinucleotide	(CCA)5	15	38043	38057	Ca5	Trinucleotide	(CCA)8	24	6286658	6286681	mRNA=Ca_09570.1;exon=Ca_09570.1.exon5;gene=Ca	GCCCCTcTTCCACCaTTCtA	GAGGTGGGGCCTTAGACATC
Ca_GpSSR00787	scaffold03730	Trinucleotide	(ATT)5	15	84198	84212	Ca5	Trinucleotide	(ATT)6	18	6320568	6320585	Intergenic	AGAGAGAGAGGAAATGGAAAA	GGGTATACTCAAATTTTGTCCT
Ca_GpSSR00788	scaffold16375	Dinucleotide	(AT)24	48	2205	2252	Ca5	Dinucleotide	(AT)8	16	6371319	6371334	Intergenic	CATGCAACCTTCTTTTCAA	AGACATGGTAGATTAAAAGTGTG
Ca_GpSSR00789	scaffold01324	Dinucleotide	(AT)11	22	34286	34307	Ca5	Dinucleotide	(AT)6	12	6941912	6941923	Intergenic	CATGCAACCTTCTTTTCAA	AGACATGGTAGATTAAAAGTGTG
Ca_GpSSR00790	scaffold01324	Dinucleotide	(CA)6	12	66298	66309	Ca5	Dinucleotide	(CA)7	14	6974563	6974576	Intergenic	TGAAGACCTATAGATGTTTGGTT	ATGATGAGATATGCACGTTTG
Ca_GpSSR00791	scaffold04922	Dinucleotide	(AT)19	38	11222	11259	Ca5	Dinucleotide	(AT)12	24	7376223	7376246	Intergenic	GTCCCAAAGCAATTTATTATC	CACTGCCACCGTATTATTAAA
Ca_GpSSR00792	scaffold26239	Trinucleotide	(ATA)47	141	1112	1252	Ca5	Trinucleotide	(ATA)29	87	7392411	7392497	Intergenic		
Ca_GpSSR00793	scaffold06887	Dinucleotide	(AT)8	16	1	16	Ca5	Dinucleotide	(AT)12	24	7866389	7866412	Intergenic	GTCCCAAAGCAATTTATTATC	CACTGCCACCGTATTATTAAA
Ca_GpSSR00794	scaffold02788	Dinucleotide	(AT)14	28	68263	68290	Ca5	Dinucleotide	(AT)22	44	7882818	7882861	Intergenic	AAGTGGAGGTTGAAAAGCCC	ACGCAATTTAACGTTCCCaA
Ca_GpSSR00795	scaffold01042	Trinucleotide	(AAT)5	15	96064	96078	Ca5	Trinucleotide	(AAT)15	45	8840304	8840348	Intergenic	TAATAAACAAAGCCTCAAGCA	TGAACTCTTCTTGCATGTAACT
Ca_GpSSR00796	scaffold02149	Trinucleotide	(TAA)33	99	12525	12623	Ca5	Trinucleotide	(TAA)26	78	8865654	8865731	Intergenic	TGGAGTGAAGAGGTAGAGACA	TTAAAATCTGAGGCCTTTTT
Ca_GpSSR00797	scaffold01959	Dinucleotide	(AT)13	26	31999	32024	Ca5	Dinucleotide	(AT)8	16	8949986	8950001	mRNA=Ca_16717.1;gene=Ca_16717;intron;	TTATTGGGGACATAATTAGCA	TCAAAAACTTCAACATCCAAG
Ca_GpSSR00798	scaffold01258	Dinucleotide	(AT)14	28	50930	50957	Ca5	Dinucleotide	(AT)13	26	9057998	9058023	Intergenic	GTCCCAAAGCAATTTATTATC	CACTGCCACCGTATTATTAAA
Ca_GpSSR00799	Ca_LG_5	Dinucleotide	(AT)16	32	15438916	15438947	Ca5	Dinucleotide	(AT)12	24	9708217	9708240	Intergenic	AGAGTTGTCAATGGATCGGA	TCGCTTTTGCAAATGACAAC
Ca_GpSSR00800	scaffold01538	Dinucleotide	(AT)9	18	49987	50004	Ca5	Dinucleotide	(AT)8	16	10173276	10173291	Intergenic	GACGAACGACCACTCTCTC	GAGAGAGAGGGAGAGAAAGAA
Ca_GpSSR00801	scaffold13591	Dinucleotide	(TA)15	30	1	30	Ca5	Dinucleotide	(TA)8	16	10458493	10458508	Intergenic	CATGCAACCTTCTTTTCAA	AGACATGGTAGATTAAAAGTGTG
Ca_GpSSR00802	scaffold13591	Dinucleotide	(TC)11	22	664	685	Ca5	Dinucleotide	(TC)12	24	10459142	10459165	Intergenic	TTCTTGGATTTGTTGTTGAGT	TAAACAATGGAGTGTTTTGGA
Ca_GpSSR00803	scaffold02942	Dinucleotide	(TA)9	18	29079	29096	Ca5	Dinucleotide	(TA)10	20	11116405	11116424	Intergenic	GCAACCGTCCCTCTAGTAAT	AATGCGGTTTAAGTTTTCG
Ca_GpSSR00804	scaffold01063	Dinucleotide	(TA)7	14	54827	54840	Ca5	Dinucleotide	(TA)8	16	11208969	11208984	gene=Ca_14872;mRNA=Ca_14872.1;intron;	CATGCAACCTTCTTTTCAA	AGACATGGTAGATTAAAAGTGTG
Ca_GpSSR00805	scaffold01063	Dinucleotide	(AT)21	42	44865	44906	Ca5	Dinucleotide	(AT)24	48	11218908	11218955	gene=Ca_14872;mRNA=Ca_14872.1;intron;	TTAGTCACTAGTTGTGTTGTCAC	TCGTAATGAGAAATCCTGAAA
Ca_GpSSR00806	scaffold01097	Dinucleotide	(AT)17	34	32269	32302	Ca5	Dinucleotide	(AT)23	46	11328143	11328188	Intergenic	GTCCCAAAGCAATTTATTATC	CACTGCCACCGTATTATTAAA
Ca_GpSSR00807	scaffold21717	Trinucleotide	(TAA)32	96	793	888	Ca5	Trinucleotide	(TAA)34	102	11828661	11828762	Intergenic	CACAACAAAGAGAGAAACCAA	TGTTTTTAACGGTTCCTTTT

Ca_GpSSR00808	scaffold03611	Trinucleotide	(ATA)8	24	5247	5270	Ca5	Trinucleotide	(ATA)7	21	11886998	11887018	Intergenic		
Ca_GpSSR00809	scaffold00779	Dinucleotide	(AT)9	18	80630	80647	Ca5	Dinucleotide	(AT)10	20	12042079	12042098	gene=Ca_14020;mRNA=Ca_14020.1;intron;	CAATCCTTTGTTGGAGTTAAA	GTGTTGATATCAGCTCCACAT
Ca_GpSSR00810	scaffold02730	Trinucleotide	(AAT)35	105	19482	19586	Ca5	Trinucleotide	(AAT)20	60	12292181	12292240	Intergenic	GGGGAAATTTTCATATAGAGC	AAAATCAGACCTTGTCCTTTT
Ca_GpSSR00811	scaffold00195	Dinucleotide	(AT)18	36	195459	195494	Ca5	Dinucleotide	(AT)17	34	12390053	12390086	Intergenic	GTCCCAAAGCAATTTATTATC	CACTGCCACCGTATTATTAAA
Ca_GpSSR00812	scaffold02711	Trinucleotide	(AAT)35	105	33934	34038	Ca5	Trinucleotide	(AAT)26	78	12969948	12970025	upstream:Ca_10495;		
Ca_GpSSR00813	scaffold02275	Trinucleotide	(AAT)30	90	17172	17261	Ca5	Trinucleotide	(AAT)24	72	13120133	13120204	Intergenic	GCGACCTCGCACTAGAACTT	GACTCCCAATTTCCTTCCCT
Ca_GpSSR00814	scaffold02275	Dinucleotide	(AT)11	22	22599	22620	Ca5	Dinucleotide	(AT)15	30	13125542	13125571	Intergenic	GTCCCAAAGCAATTTATTATC	CACTGCCACCGTATTATTAAA
Ca_GpSSR00815	scaffold02728	Dinucleotide	(TA)24	48	15287	15334	Ca5	Dinucleotide	(TA)20	40	13826424	13826463	Intergenic	GTCCCAAAGCAATTTATTATC	CACTGCCACCGTATTATTAAA
Ca_GpSSR00816	scaffold05664	Dinucleotide	(AT)7	14	11475	11488	Ca5	Dinucleotide	(AT)8	16	14192500	14192515	Intergenic	ACCTATGTGTTTGCCTTAGTG	TTTTTAGCGCTATAGTTAGCAA
Ca_GpSSR00817	scaffold01652	Dinucleotide	(AT)21	42	60947	60988	Ca5	Dinucleotide	(AT)7	14	15496297	15496310	Intergenic	AATAAATTAGGGCATCTCGAC	TGAAATGCTAGTTAGGGATGA
Ca_GpSSR00818	scaffold04414	Dinucleotide	(TA)9	18	4666	4683	Ca5	Dinucleotide	(TA)8	16	16126633	16126648	mRNA=Ca_18930.1;gene=Ca_18930;intron;	CAAAAGCATTGAAATGAGAGT	TTTATACCGCTTTCACTCGTA
Ca_GpSSR00819	scaffold03890	Trinucleotide	(AAT)21	63	22883	22945	Ca5	Trinucleotide	(AAT)18	54	16470096	16470149	Intergenic	TTGAACGTTATATCTTAGATTCG	GAAATAGAGAAAATCCCTACTCT
Ca_GpSSR00820	scaffold01744	Dinucleotide	(TA)17	34	45683	45716	Ca5	Dinucleotide	(TA)15	30	16540432	16540461	Intergenic	GTATAATAGCCATGCACAAAAA	GGTTAAAGGATCCAATCATTC
Ca_GpSSR00821	scaffold01744	Dinucleotide	(AT)14	28	46043	46070	Ca5	Dinucleotide	(AT)16	32	16540620	16540651	Intergenic	GTATAATAGCCATGCACAAAAA	GGTTAAAGGATCCAATCATTC
Ca_GpSSR00822	scaffold01633	Dinucleotide	(AT)12	24	31002	31025	Ca5	Dinucleotide	(AT)11	22	16881791	16881812	Intergenic	TTTCAAAATTACTGCCCTACT	CTTCAAAATTTATGTGGTTGG
Ca_GpSSR00823	scaffold00857	Trinucleotide	(TTA)24	72	65131	65202	Ca5	Trinucleotide	(TTA)27	81	17774911	17774991	Intergenic	tGTGTGTGGACGTGTGATTG	TTCGTTTACCAATAcCAAtTTTCa
Ca_GpSSR00824	scaffold05660	Trinucleotide	(TAA)21	63	6876	6938	Ca5	Trinucleotide	(TAA)25	75	17797031	17797105	Intergenic	CACATAGGTCAAACCCAATAA	ACTCACTCCTTAAGTGGAAGC
Ca_GpSSR00825	scaffold08467	Dinucleotide	(AT)11	22	5357	5378	Ca5	Dinucleotide	(AT)10	20	17819562	17819581	Intergenic	CACACAAAGTGAGATGTACCA	TGTAAGCGAAAATAAAGTTGTG
Ca_GpSSR00826	Ca_LG_2	Dinucleotide	(TA)13	26	16272516	16272541	Ca5	Dinucleotide	(TA)12	24	18003818	18003841	Intergenic	GTCCCAAAGCAATTTATTATC	CACTGCCACCGTATTATTAAA
Ca_GpSSR00827	Ca_LG_2	Trinucleotide	(TAA)15	45	16284604	16284648	Ca5	Trinucleotide	(TAA)12	36	18015101	18015136	Intergenic	GCCCGTCTAGAGAAACATCTA	GATTGGATGTATGTGCATTTT
Ca_GpSSR00828	scaffold11200	Dinucleotide	(AT)19	38	39	76	Ca5	Dinucleotide	(AT)13	26	18058126	18058151	Intergenic	GTCCCAAAGCAATTTATTATC	CACTGCCACCGTATTATTAAA
Ca_GpSSR00829	scaffold06357	Dinucleotide	(TA)18	36	6070	6105	Ca5	Dinucleotide	(TA)15	30	18612827	18612856	Intergenic	GTCCCAAAGCAATTTATTATC	CACTGCCACCGTATTATTAAA
Ca_GpSSR00830	scaffold02689	Dinucleotide	(TA)24	48	15448	15495	Ca5	Dinucleotide	(TA)7	14	18905071	18905084	Intergenic	CATGCAACCTTCTTTTCAA	AGACATGGTAGATTAAAAGTGTG
Ca_GpSSR00831	Ca_LG_5	Dinucleotide	(TA)7	14	14217008	14217021	Ca5	Dinucleotide	(TA)9	18	19640669	19640686	Intergenic	CTTCTTTACATCTGCTCTCCA	GCGCAAAATTCCTTTTAGTAT
Ca_GpSSR00832	Ca_LG_5	Dinucleotide	(AT)19	38	14320433	14320470	Ca5	Dinucleotide	(AT)24	48	19863173	19863220	Intergenic	GTCCCAAAGCAATTTATTATC	CACTGCCACCGTATTATTAAA
Ca_GpSSR00833	Ca_LG_5	Dinucleotide	(AT)10	20	14359224	14359243	Ca5	Dinucleotide	(AT)11	22	19904232	19904253	Intergenic	CTTCTTTACATCTGCTCTCCA	GCGCAAAATTCCTTTTAGTAT
Ca_GpSSR00834	Ca_LG_5	Trinucleotide	(TAA)8	24	15138160	15138183	Ca5	Trinucleotide	(TAA)10	30	20176966	20176995	Intergenic	AACGCTTTCCTACCAATTTTT	GGAGGAAGTCGTTAACAAAAGA
Ca_GpSSR00835	scaffold03002	Dinucleotide	(TA)7	14	34331	34344	Ca5	Dinucleotide	(TA)6	12	20546658	20546669	Intergenic	ACCTTCCTCTTTTTGATTTTC	AAAAGAAGTGCAAGACACAAG
Ca_GpSSR00836	scaffold01618	Trinucleotide	(AAT)13	39	17434	17472	Ca5	Trinucleotide	(AAT)14	42	20759181	20759222	Intergenic	TTTCATGTTGTAGATGAGATCAA	TACAAGTTGCAACCCAATTAT
Ca_GpSSR00837	scaffold01618	Trinucleotide	(ATT)8	24	42128	42151	Ca5	Trinucleotide	(ATT)19	57	20784629	20784685	Intergenic	CCAAACTAAATATTGCAAAC	GCACAGGACAATAAAATGAAC
Ca_GpSSR00838	scaffold00785	Dinucleotide	(AT)11	22	46436	46457	Ca5	Dinucleotide	(AT)14	28	22612293	22612320	Intergenic	AAAACCTTTGGGAAATCG	GGAAATTTAAGTTCGAAATGC
Ca_GpSSR00839	scaffold14763	Dinucleotide	(AT)15	30	2642	2671	Ca5	Dinucleotide	(AT)13	26	22723105	22723130	Intergenic	GTCCCAAAGCAATTTATTATC	CACTGCCACCGTATTATTAAA
Ca_GpSSR00840	scaffold00215	Dinucleotide	(AT)8	16	192380	192395	Ca5	Dinucleotide	(AT)6	12	23725211	23725222	Intergenic	AGTTTTCGATTGATGCAAAT	CATAATTGAGGTATGTTATTCCA
Ca_GpSSR00841	scaffold00924	Dinucleotide	(TA)11	22	35798	35819	Ca5	Dinucleotide	(TA)12	24	24045722	24045745	Intergenic	TTGGAAAGAAGTCATTCGTTA	TGGTTTATAATTGCCGTAAGA
Ca_GpSSR00842	scaffold00924	Dinucleotide	(TA)8	16	40614	40629	Ca5	Dinucleotide	(TA)9	18	24050542	24050559	Intergenic	TTGGAAAGAAGTCATTCGTTA	TGGTTTATAATTGCCGTAAGA
Ca_GpSSR00843	scaffold00924	Trinucleotide	(ATT)24	72	65927	65998	Ca5	Trinucleotide	(ATT)20	60	24073883	24073942	Intergenic	CCAAACTAAATATTGCAAAC	GCACAGGACAATAAAATGAAC
Ca_GpSSR00844	scaffold00408	Dinucleotide	(TA)7	14	29807	29820	Ca5	Dinucleotide	(TA)25	50	24525862	24525911	Intergenic	GTCCCAAAGCAATTTATTATC	CACTGCCACCGTATTATTAAA
Ca_GpSSR00845	scaffold01817	Dinucleotide	(TA)27	54	19298	19351	Ca5	Dinucleotide	(TA)11	22	24880237	24880258	Intergenic	CAAATCCCATTATATCCATCA	AACCATTTTGTGTATGAAGGT
Ca_GpSSR00846	scaffold01428	Dinucleotide	(TA)10	20	23868	23887	Ca5	Dinucleotide	(TA)12	24	25132631	25132654	Intergenic	GTCCCAAAGCAATTTATTATC	CACTGCCACCGTATTATTAAA
Ca_GpSSR00847	Ca_LG_5	Dinucleotide	(TA)8	16	14115634	14115649	Ca5	Dinucleotide	(TA)9	18	25364006	25364023	Intergenic	CTTCTTTACATCTGCTCTCCA	GCGCAAAATTCCTTTTAGTAT
Ca_GpSSR00848	scaffold56007	Trinucleotide	(TTA)16	48	544	591	Ca5	Trinucleotide	(TTA)10	30	25427042	25427071	Intergenic	CCTCGGAGTTACATAATACAAAT	TGTATCTTAACAACTCCAAGATT
Ca_GpSSR00849	scaffold00225	Dinucleotide	(AT)16	32	81661	81692	Ca5	Dinucleotide	(AT)21	42	25756857	25756898	Intergenic	TtATATAAAaTTCGATATGCAGGATCT	tCTcAATTCaAACTTCCAcTCAA
Ca_GpSSR00850	scaffold00312	Dinucleotide	(AT)19	38	201799	201836	Ca5	Dinucleotide	(AT)17	34	25902949	25902982	Intergenic	GTCCCAAAGCAATTTATTATC	CACTGCCACCGTATTATTAAA
Ca_GpSSR00851	Ca_LG_5	Trinucleotide	(ATA)16	48	13965236	13965283	Ca5	Trinucleotide	(ATA)13	39	26159872	26159910	Intergenic		
Ca_GpSSR00852	Ca_LG_5	Trinucleotide	(TAT)14	42	14028154	14028195	Ca5	Trinucleotide	(TAT)16	48	26213472	26213519	Intergenic		
Ca_GpSSR00853	Ca_LG_5	Trinucleotide	(TTA)37	111	14038871	14038981	Ca5	Trinucleotide	(TTA)30	90	26224427	26224516	Intergenic		
Ca_GpSSR00854	scaffold02068	Dinucleotide	(AT)19	38	14945	14982	Ca5	Dinucleotide	(AT)6	12	26256880	26256891	Intergenic	CATGCAACCTTCTTTTCAA	AGACATGGTAGATTAAAAGTGTG
Ca_GpSSR00855	scaffold02068	Trinucleotide	(TTA)21	63	17591	17653	Ca5	Trinucleotide	(TTA)22	66	26260311	26260376	Intergenic	GTTTATTAATCCCTCCTTTTTCT	TTGATCGTGTAACGCTTAAAT
Ca_GpSSR00856	scaffold04553	Dinucleotide	(AT)21	42	10912	10953	Ca5	Dinucleotide	(AT)26	52	26316866	26316917	Intergenic	GTCCCAAAGCAATTTATTATC	CACTGCCACCGTATTATTAAA
Ca_GpSSR00857	scaffold00809	Dinucleotide	(AT)10	20	4834	4853	Ca5	Dinucleotide	(AT)9	18	26329316	26329333	Intergenic	GACATGATGGCAATAAGGTAA	CACATTGTGAGATAACCGTTC
Ca_GpSSR00858	scaffold00809	Dinucleotide	(GA)8	16	47863	47878	Ca5	Dinucleotide	(GA)7	14	26374447	26374460	5'-UTR=Ca_14131.1.5'-UTR1;mRNA=Ca_14131.1;gene	CCATATTGGACGAACCTTT	CTCTTCAACTTCTCAGCTTCA
Ca_GpSSR00859	Ca_LG_5	Trinucleotide	(TCA)7	21	13582150	13582170	Ca5	Trinucleotide	(TCA)8	24	26833071	26833094	exon=Ca_06351.1.exon1;mRNA=Ca_06351.1;gene=Ca	AAAATAGGTGCAGAGAAAGGT	GTGGCACTTTTGCTAATTTT
Ca_GpSSR00860	scaffold00702	Dinucleotide	(TA)18	36	34989	35024	Ca5	Dinucleotide	(TA)16	32	26907451	26907482	Intergenic	GTCCCAAAGCAATTTATTATC	CACTGCCACCGTATTATTAAA
Ca_GpSSR00861	scaffold15808	Dinucleotide	(AT)8	16	2387	2402	Ca5	Dinucleotide	(AT)14	28	27249907	27249934	Intergenic	GTCCCAAAGCAATTTATTATC	CACTGCCACCGTATTATTAAA
Ca_GpSSR00862	scaffold00642	Dinucleotide	(TA)6	12	54926	54937	Ca5	Dinucleotide	(TA)7	14	27365405	27365418	Intergenic	TTTCTCCCTTGTTTGTCATC	ACCAAACCACTACCTGAAAAT
Ca_GpSSR00863	scaffold00642	Dinucleotide	(TA)9	18	75104	75121	Ca5	Dinucleotide	(TA)11	22	27389758	27389779	Intergenic	TTTCTCCCTTGTTTGTCATC	ACCAAACCACTACCTGAAAAT
Ca_GpSSR00864	scaffold02127	Dinucleotide	(AT)7	14	5563	5576	Ca5	Dinucleotide	(AT)8	16	27603144	27603159	upstream:Ca_16956;	TTTTATCGGAAACGTTATTG	AGAAAATCTGAATCTGCAACA
Ca_GpSSR00865	scaffold02127	Dinucleotide	(TA)11	22	13391	13412	Ca5	Dinucleotide	(TA)10	20	27609540	27609559	Intergenic	TTAGGGATTTTGTTAAGTGGA	TGTATACCCAAAAGGAGTTAAGA
Ca_GpSSR00866	scaffold01148	Dinucleotide	(AT)13	26	17224	17249	Ca5	Dinucleotide	(AT)20	40	27846628	27846667	Intergenic	GTCCCAAAGCAATTTATTATC	CACTGCCACCGTATTATTAAA
Ca_GpSSR00867	scaffold00441	Dinucleotide	(AT)7	14	103669	103682	Ca5	Dinucleotide	(AT)8	16	28012408	28012423	upstream:Ca_09236;	TTTTCCTTTTCCTTCCTTCTA	TTAATGCGTTACGAGTGAAAT
Ca_GpSSR00868	scaffold00978	Dinucleotide	(TA)11	22	23039	23060	Ca5	Dinucleotide	(TA)25	50	28109108	28109157	Intergenic	TAGGGTAATTTGGTGTTTTT	TGCAATTTATGTTACTCTCATGT
Ca_GpSSR00869	scaffold00978	Dinucleotide	(AT)9	18	49512	49529	Ca5	Dinucleotide	(AT)8	16	28136432	28136447	Intergenic	TAGGGTAATTTGGTGTTTTT	TGCAATTTATGTTACTCTCATGT
Ca_GpSSR00870	scaffold07913	Dinucleotide	(TA)10	20	3918	3937	Ca5	Dinucleotide	(TA)9	18	28224949	28224966	Intergenic	AAAATATATCGACTGGATTGTTG	GAATATTTGTCAATCGGAAGA
Ca_GpSSR00871	scaffold02013	Dinucleotide	(TA)9	18	46143	46160	Ca5	Dinucleotide	(TA)10	20	28241838	28241857	Intergenic	TCGAAATAGAAGAAGACACACA	ACGGTTAAGAAGTTGAATGGT
Ca_GpSSR00872	scaffold62771	Dinucleotide	(TA)15	30	553	582	Ca5	Dinucleotide	(TA)16	32	28366917	28366948	Intergenic	GTCCCAAAGCAATTTATTATC	CACTGCCACCGTATTATTAAA
Ca_GpSSR00873	scaffold00113	Dinucleotide	(TA)7	14	99053	99066	Ca5	Dinucleotide	(TA)8	16	28870695	28870710	Intergenic	CATGCAACCTTCTTTTCAA	AGACATGGTAGATTAAAAGTGTG
Ca_GpSSR00874	scaffold00113	Dinucleotide	(AT)16	32	42357	42388	Ca5	Dinucleotide	(AT)8	16	28927562	28927577	Intergenic	CATGCAACCTTCTTTTCAA	AGACATGGTAGATTAAAAGTGTG
Ca_GpSSR00875	scaffold28810	Dinucleotide	(TA)9	18	581	598	Ca5	Dinucleotide	(TA)10	20	29190002	29190021	Intergenic	CTCTATGGCATGGTGTAAAAT	CTATATCCCTACGTCTCATGC
Ca_GpSSR00876	scaffold00825	Dinucleotide	(TA)18	36	15701	15736	Ca5	Dinucleotide	(TA)19	38	29394830	29394867	Intergenic	GTCCCAAAGCAATTTATTATC	CACTGCCACCGTATTATTAAA
Ca_GpSSR00877	scaffold03538	Dinucleotide	(TA)9	18	18060	18077	Ca5	Dinucleotide	(TA)8	16	29418888	29418903	Intergenic	CATTGACATGTAAACCTCAAAA	TGAGTCAAATGAGGTGAACTT
Ca_GpSSR00878	scaffold00245	Trinucleotide	(TAA)13	39	8372	8410	Ca5	Trinucleotide	(TAA)14	42	29450061	29450102	Intergenic	GTACTTCAATTTTGTCCAATCA	CAGCCAGTGAAACCAATATAC
Ca_GpSSR00879	scaffold00245	Dinucleotide	(TA)12	24	29026	29049	Ca5	Dinucleotide	(TA)7	14	29470732	29470745	Intergenic	CATGCAACCTTCTTTTCAA	AGACATGGTAGATTAAAAGTGTG
Ca_GpSSR00880	scaffold00245	Dinucleotide	(TA)9	18	38632	38649	Ca5	Dinucleotide	(TA)15	30	29479495	29479524	Intergenic	CTCGTACTGTGAGAAAACCTcG	AGCTTGCAAATGCGATTCTT
Ca_GpSSR00881	scaffold00245	Dinucleotide	(TA)17	34	51882	51915	Ca5	Dinucleotide	(TA)24	48	29492793	29492840	Intergenic	GTCCCAAAGCAATTTATTATC	CACTGCCACCGTATTATTAAA
Ca_GpSSR00882	scaffold00245	Trinucleotide	(ATA)21	63	117729	117791	Ca5	Trinucleotide	(ATA)18	54	29568805	29568858	upstream:Ca_11528;	TTTTTCTTTTCCATAATGTGCG	CACCGGTTCAATTTTCGATT
Ca_GpSSR00883	scaffold00245	Dinucleotide	(AT)13	26	138837	138862	Ca5	Dinucleotide	(AT)15	30	29592861	29592890	upstream:Ca_11530;	TGGCTCTCAAATCATATTCAT	TGTGCATGGATATTTTGTTATC
Ca_GpSSR00884	scaffold00245	Dinucleotide	(AT)9	18	209162	209179	Ca5	Dinucleotide	(AT)8	16	29663444	29663459	upstream:Ca_11535;	CATGCAACCTTCTTTTCAA	AGACATGGTAGATTAAAAGTGTG
Ca_GpSSR00885	scaffold08630	Dinucleotide	(TA)14	28	1210	1237	Ca5	Dinucleotide	(TA)12	24	29691490	29691513	Intergenic	TGGGTCGTAAGCTAAGTTTTT	TGATCCGACCAGAGTTAAATA
Ca_GpSSR00886	scaffold03274	Dinucleotide	(AT)16	32	12228	12259	Ca5	Dinucleotide	(AT)17	34	29735775	29735808	Intergenic	GTCCCAAAGCAATTTATTATC	CACTGCCACCGTATTATTAAA
Ca_GpSSR00887	scaffold01241	Dinucleotide	(CA)8	16	46349	46364	Ca5	Dinucleotide	(CA)9	18	29954908	29954925	Intergenic	TTCTCGTTAAGGAATGAAGTCT	GTTATGGTCGGTGTCGTAATA
Ca_GpSSR00888	scaffold01241	Trinucleotide	(TAT)11	33	70721	70753	Ca5	Trinucleotide	(TAT)12	36	29978018	29978053	upstream:Ca_15307;		

Ca_GpSSR00889	scaffold07887	Dinucleotide	(TA)11	22	3553	3574	Ca5	Dinucleotide	(TA)15	30	30114887	30114916	Intergenic	TATAAACTCTTTCCGCTCACC	ACCTTTTTAGTTGGCTACCAT
Ca_GpSSR00890	scaffold01734	Dinucleotide	(TA)9	18	14401	14418	Ca5	Dinucleotide	(TA)8	16	30338195	30338210	Intergenic	AGAACATCTTATTTGGACAAG	TATGTAAATCCCAACACGAAG
Ca_GpSSR00891	scaffold06201	Dinucleotide	(TA)14	28	843	870	Ca5	Dinucleotide	(TA)9	18	30610219	30610236	Intergenic	AAATGAGTGGTAGAAATGAACA	AGAGGACGAACTTGATACTATT
Ca_GpSSR00892	Ca_LG_5	Dinucleotide	(AT)13	26	1360543	1360568	Ca5	Dinucleotide	(AT)12	24	31056193	31056216	Intergenic	CTTCTTTACATCTGCTCTCCA	GCGCAAAATTCCTTTTAGTAT
Ca_GpSSR00893	Ca_LG_5	Dinucleotide	(AT)20	40	1404766	1404805	Ca5	Dinucleotide	(AT)14	28	31101286	31101313	upstream:Ca_05304;	GTCCCAAAGCAATTTATTATC	CACTGCCACCGTATTATTAAA
Ca_GpSSR00894	Ca_LG_5	Dinucleotide	(TA)9	18	1559295	1559312	Ca5	Dinucleotide	(TA)10	20	31256886	31256905	Intergenic	CTTCTTTACATCTGCTCTCCA	GCGCAAAATTCCTTTTAGTAT
Ca_GpSSR00895	Ca_LG_5	Trinucleotide	(TTA)14	42	1589000	1589041	Ca5	Trinucleotide	(TTA)15	45	31286653	31286697	Intergenic		
Ca_GpSSR00896	scaffold07375	Dinucleotide	(AC)7	14	937	950	Ca5	Dinucleotide	(AC)8	16	31489306	31489321	gene=Ca_19887;mRNA=Ca_19887.1;intron;	AATTGTTGATACTGCATGGTG	AGTTTTGTTTTTGAAAGGTACAG
Ca_GpSSR00897	Ca_LG_5	Dinucleotide	(AT)17	34	1796552	1796585	Ca5	Dinucleotide	(AT)13	26	31503890	31503915	upstream:Ca_05343;	GTCCCAAAGCAATTTATTATC	CACTGCCACCGTATTATTAAA
Ca_GpSSR00898	Ca_LG_5	Dinucleotide	(AT)10	20	1955721	1955740	Ca5	Dinucleotide	(AT)9	18	31663883	31663900	downstream:Ca_05358;	CTTCTTTACATCTGCTCTCCA	GCGCAAAATTCCTTTTAGTAT
Ca_GpSSR00899	scaffold01357	Dinucleotide	(AT)10	20	6903	6922	Ca5	Dinucleotide	(AT)16	32	31901747	31901778	Intergenic	TCGTGTACTGTCTCATCCTTC	GAATACGTGATCGTTTATGGA
Ca_GpSSR00900	scaffold02357	Dinucleotide	(AT)9	18	16779	16796	Ca5	Dinucleotide	(AT)11	22	31930750	31930771	Intergenic	AGGGAGAAAGATGAAGAAAAA	AAATTTCTTGAGGTTGGAGTC
Ca_GpSSR00901	scaffold06965	Dinucleotide	(AT)10	20	2678	2697	Ca5	Dinucleotide	(AT)9	18	31960995	31961012	Intergenic	TCCACGGTTCTTTTACAGATA	CTTTTTCAAAAGCATCTCTTAAC
Ca_GpSSR00902	Ca_LG_5	Dinucleotide	(TA)11	22	2317836	2317857	Ca5	Dinucleotide	(TA)9	18	32360829	32360846	upstream:Ca_05382;	CTTCTTTACATCTGCTCTCCA	GCGCAAAATTCCTTTTAGTAT
Ca_GpSSR00903	Ca_LG_5	Trinucleotide	(ATA)10	30	2345966	2345995	Ca5	Trinucleotide	(ATA)12	36	32388361	32388396	Intergenic	TTGAGGACATGCATATGTTTT	TCTCAAGATAAAACTCTCGATT
Ca_GpSSR00904	Ca_LG_5	Dinucleotide	(TC)9	18	2592222	2592239	Ca5	Dinucleotide	(TC)24	48	32627922	32627969	gene=Ca_05419;mRNA=Ca_05419.1;intron;	TTCAGGATTTGCTTGATGGA	CATAACCGCATAAGGGCATT
Ca_GpSSR00905	Ca_LG_5	Dinucleotide	(TG)9	18	2634079	2634096	Ca5	Dinucleotide	(TG)8	16	32673384	32673399	mRNA=Ca_05427.1;gene=Ca_05427;intron;	GAAACAATATCTCCTGCTTCA	TGCCAGTAAGTGCAAGATAAT
Ca_GpSSR00906	Ca_LG_5	Dinucleotide	(AT)15	30	2663535	2663564	Ca5	Dinucleotide	(AT)14	28	32704939	32704966	Intergenic	GTCCCAAAGCAATTTATTATC	CACTGCCACCGTATTATTAAA
Ca_GpSSR00907	Ca_LG_5	Dinucleotide	(AT)21	42	2783303	2783344	Ca5	Dinucleotide	(AT)9	18	32879761	32879778	upstream:Ca_05437;	CATGCAACCTTCTTTTCAA	AGACATGGTAGATTAAAAGTGTG
Ca_GpSSR00908	Ca_LG_5	Dinucleotide	(AT)12	24	2822411	2822434	Ca5	Dinucleotide	(AT)11	22	32915713	32915734	Intergenic	CTTCTTTACATCTGCTCTCCA	GCGCAAAATTCCTTTTAGTAT
Ca_GpSSR00909	scaffold04182	Trinucleotide	(TTA)34	102	8047	8148	Ca5	Trinucleotide	(TTA)21	63	33049556	33049618	Intergenic		
Ca_GpSSR00910	Ca_LG_5	Trinucleotide	(GAC)5	15	3087188	3087202	Ca5	Trinucleotide	(GAC)6	18	33336008	33336025	Intergenic	CTATTTGAGTTTCACCCCTTT	GTTGTGGTGGTTCACTATCAT
Ca_GpSSR00911	Ca_LG_5	Dinucleotide	(AT)18	36	3138651	3138686	Ca5	Dinucleotide	(AT)14	28	33384809	33384836	Intergenic	GTCCCAAAGCAATTTATTATC	CACTGCCACCGTATTATTAAA
Ca_GpSSR00912	scaffold46278	Dinucleotide	(AT)13	26	677	702	Ca5	Dinucleotide	(AT)11	22	33662321	33662342	Intergenic	GTCCCAAAGCAATTTATTATC	CACTGCCACCGTATTATTAAA
Ca_GpSSR00913	scaffold22151	Dinucleotide	(AT)14	28	1	28	Ca5	Dinucleotide	(AT)26	52	33855413	33855464	Intergenic	GTCCCAAAGCAATTTATTATC	CACTGCCACCGTATTATTAAA
Ca_GpSSR00914	Ca_LG_5	Dinucleotide	(TA)11	22	3500799	3500820	Ca5	Dinucleotide	(TA)16	32	34084354	34084385	upstream:Ca_05505;	CTTCTTTACATCTGCTCTCCA	GCGCAAAATTCCTTTTAGTAT
Ca_GpSSR00915	Ca_LG_5	Dinucleotide	(AT)11	22	3517750	3517771	Ca5	Dinucleotide	(AT)10	20	34101600	34101619	Intergenic	CTTCTTTACATCTGCTCTCCA	GCGCAAAATTCCTTTTAGTAT
Ca_GpSSR00916	scaffold121098	Trinucleotide	(TAT)32	96	217	312	Ca5	Trinucleotide	(TAT)19	57	34382742	34382798	Intergenic		
Ca_GpSSR00917	Ca_LG_4	Dinucleotide	(TA)7	14	8086803	8086816	Ca5	Dinucleotide	(TA)12	24	34466075	34466098	Intergenic	AGATGTACAATCTTTTGAGACAA	GCTGCAAAATGGTACATAGAC
Ca_GpSSR00918	scaffold27627	Trinucleotide	(ATA)13	39	341	379	Ca5	Trinucleotide	(ATA)12	36	34466519	34466554	Intergenic		
Ca_GpSSR00919	Ca_LG_4	Dinucleotide	(AT)14	28	8170593	8170620	Ca5	Dinucleotide	(AT)13	26	34549657	34549682	Intergenic	GTCCCAAAGCAATTTATTATC	CACTGCCACCGTATTATTAAA
Ca_GpSSR00920	Ca_LG_4	Trinucleotide	(AAT)15	45	8243439	8243483	Ca5	Trinucleotide	(AAT)11	33	34620470	34620502	Intergenic		
Ca_GpSSR00921	Ca_LG_4	Dinucleotide	(TA)7	14	8278820	8278833	Ca5	Dinucleotide	(TA)6	12	34656068	34656079	Intergenic	AGATGTACAATCTTTTGAGACAA	GCTGCAAAATGGTACATAGAC
Ca_GpSSR00922	Ca_LG_4	Trinucleotide	(ATA)22	66	8321447	8321512	Ca5	Trinucleotide	(ATA)11	33	34698064	34698096	upstream:Ca_04428;		
Ca_GpSSR00923	scaffold07358	Dinucleotide	(TA)7	14	2404	2417	Ca5	Dinucleotide	(TA)11	22	35031824	35031845	Intergenic	TGCGTAAGAGAGGTATATGGA	GGGAGTCAGCACCATTTAT
Ca_GpSSR00924	Ca_LG_5	Dinucleotide	(AT)9	18	4380108	4380125	Ca5	Dinucleotide	(AT)10	20	35157633	35157652	upstream:Ca_05525;	CTTCTTTACATCTGCTCTCCA	GCGCAAAATTCCTTTTAGTAT
Ca_GpSSR00925	Ca_LG_5	Trinucleotide	(ATA)28	84	4426508	4426591	Ca5	Trinucleotide	(ATA)29	87	35204595	35204681	upstream:Ca_05531;		
Ca_GpSSR00926	Ca_LG_5	Trinucleotide	(AAT)56	168	4571757	4571924	Ca5	Trinucleotide	(AAT)12	36	35348713	35348748	Intergenic		
Ca_GpSSR00927	Ca_LG_5	Dinucleotide	(TA)8	16	4606957	4606972	Ca5	Dinucleotide	(TA)7	14	35383408	35383421	mRNA=Ca_05552.1;3'-UTR=Ca_05552.1.3'-UTR1;gene	CTTCTTTACATCTGCTCTCCA	GCGCAAAATTCCTTTTAGTAT
Ca_GpSSR00928	scaffold08685	Dinucleotide	(AT)16	32	5	36	Ca5	Dinucleotide	(AT)7	14	35492915	35492928	Intergenic	CATGCAACCTTCTTTTCAA	AGACATGGTAGATTAAAAGTGTG
Ca_GpSSR00929	scaffold08685	Dinucleotide	(AT)15	30	2186	2215	Ca5	Dinucleotide	(AT)12	24	35495080	35495103	Intergenic	GTCCCAAAGCAATTTATTATC	CACTGCCACCGTATTATTAAA
Ca_GpSSR00930	Ca_LG_5	Dinucleotide	(AT)7	14	4669159	4669172	Ca5	Dinucleotide	(AT)6	12	35602928	35602939	mRNA=Ca_05557.1;gene=Ca_05557;intron;	CTTCTTTACATCTGCTCTCCA	GCGCAAAATTCCTTTTAGTAT
Ca_GpSSR00931	Ca_LG_5	Trinucleotide	(AAT)35	105	4673759	4673863	Ca5	Trinucleotide	(AAT)20	60	35607530	35607589	Intergenic	TCATCTGATCACTAATACACCAA	GAAGAAAAAGAACCATTTGACT
Ca_GpSSR00932	Ca_LG_5	Dinucleotide	(TA)9	18	4834792	4834809	Ca5	Dinucleotide	(TA)11	22	35770233	35770254	upstream:Ca_05583;	CTTCTTTACATCTGCTCTCCA	GCGCAAAATTCCTTTTAGTAT
Ca_GpSSR00933	Ca_LG_5	Dinucleotide	(AT)11	22	4880574	4880595	Ca5	Dinucleotide	(AT)10	20	35815982	35816001	Intergenic	CTTCTTTACATCTGCTCTCCA	GCGCAAAATTCCTTTTAGTAT
Ca_GpSSR00934	Ca_LG_5	Dinucleotide	(TA)6	12	4949032	4949043	Ca5	Dinucleotide	(TA)7	14	35886784	35886797	gene=Ca_05597;mRNA=Ca_05597.1;intron;	GCTTTCAAAACATATCAACAAG	CCATCTACGTTGTCTCAGGTA
Ca_GpSSR00935	scaffold00189	Dinucleotide	(TA)9	18	253981	253998	Ca5	Dinucleotide	(TA)10	20	35973136	35973155	Intergenic	ACATGAGCGCATAAGTTTTTA	CACGGAAACAACGATTTTA
Ca_GpSSR00936	scaffold00158	Dinucleotide	(AT)18	36	152844	152879	Ca5	Dinucleotide	(AT)17	34	36503696	36503729	upstream:Ca_11074;	GTCCCAAAGCAATTTATTATC	CACTGCCACCGTATTATTAAA
Ca_GpSSR00937	Ca_LG_5	Dinucleotide	(TA)17	34	5609103	5609136	Ca5	Dinucleotide	(TA)10	20	36723236	36723255	Intergenic	TGCGTAAGAGAGGTATATGGA	GGGAGTCAGCACCATTTAT
Ca_GpSSR00938	Ca_LG_5	Dinucleotide	(TA)26	52	5771063	5771114	Ca5	Dinucleotide	(TA)13	26	36873482	36873507	Intergenic	GTCCCAAAGCAATTTATTATC	CACTGCCACCGTATTATTAAA
Ca_GpSSR00939	Ca_LG_5	Dinucleotide	(TA)11	22	5771679	5771700	Ca5	Dinucleotide	(TA)12	24	36874049	36874072	Intergenic	CTTCTTTACATCTGCTCTCCA	GCGCAAAATTCCTTTTAGTAT
Ca_GpSSR00940	scaffold00885	Dinucleotide	(TA)9	18	26099	26116	Ca5	Dinucleotide	(TA)10	20	37056418	37056437	Intergenic	CGATTAGAGATCAATGCAGAG	GATGGTAGAATTTTGTGTCCA
Ca_GpSSR00941	scaffold00885	Dinucleotide	(TA)11	22	78902	78923	Ca5	Dinucleotide	(TA)13	26	37108496	37108521	upstream:Ca_14389;	GTCCCAAAGCAATTTATTATC	CACTGCCACCGTATTATTAAA
Ca_GpSSR00942	Ca_LG_5	Dinucleotide	(AT)8	16	5206845	5206860	Ca5	Dinucleotide	(AT)9	18	37460032	37460049	mRNA=Ca_05622.1;gene=Ca_05622;intron;	CTTCTTTACATCTGCTCTCCA	GCGCAAAATTCCTTTTAGTAT
Ca_GpSSR00943	Ca_LG_5	Trinucleotide	(TTA)7	21	5265718	5265738	Ca5	Trinucleotide	(TTA)5	15	37521123	37521137	Intergenic	CTTGGTGTGATCTTCACGGA	AGACAAAAACGGGCTAGTTAAA
Ca_GpSSR00944	Ca_LG_5	Dinucleotide	(TA)21	42	6186593	6186634	Ca5	Dinucleotide	(TA)18	36	37704365	37704400	Intergenic	GTCCCAAAGCAATTTATTATC	CACTGCCACCGTATTATTAAA
Ca_GpSSR00945	Ca_LG_5	Dinucleotide	(AT)11	22	6226185	6226206	Ca5	Dinucleotide	(AT)12	24	38157188	38157211	Intergenic	CTTCTTTACATCTGCTCTCCA	GCGCAAAATTCCTTTTAGTAT
Ca_GpSSR00946	Ca_LG_7	Dinucleotide	(AT)10	20	2381510	2381529	Ca5	Dinucleotide	(AT)11	22	38903078	38903099	Intergenic	GTCCCAAAGCAATTTATTATC	CACTGCCACCGTATTATTAAA
Ca_GpSSR00947	scaffold08723	Dinucleotide	(TA)16	32	6075	6106	Ca5	Dinucleotide	(TA)22	44	38941088	38941131	upstream:Ca_20095;	GTCCCAAAGCAATTTATTATC	CACTGCCACCGTATTATTAAA
Ca_GpSSR00948	Ca_LG_5	Dinucleotide	(AT)14	28	11740620	11740647	Ca5	Dinucleotide	(AT)18	36	39188155	39188190	Intergenic	GTCCCAAAGCAATTTATTATC	CACTGCCACCGTATTATTAAA
Ca_GpSSR00949	Ca_LG_5	Dinucleotide	(AT)10	20	11706240	11706259	Ca5	Dinucleotide	(AT)11	22	39222389	39222410	mRNA=Ca_06213.1;gene=Ca_06213;intron;	CTTCTTTACATCTGCTCTCCA	GCGCAAAATTCCTTTTAGTAT
Ca_GpSSR00950	scaffold06162	Dinucleotide	(AT)15	30	9899	9928	Ca5	Dinucleotide	(AT)26	52	39407800	39407851	Intergenic	GTCCCAAAGCAATTTATTATC	CACTGCCACCGTATTATTAAA
Ca_GpSSR00951	Ca_LG_5	Dinucleotide	(AT)9	18	11489066	11489083	Ca5	Dinucleotide	(AT)11	22	39449602	39449623	Intergenic	CTTCTTTACATCTGCTCTCCA	GCGCAAAATTCCTTTTAGTAT
Ca_GpSSR00952	Ca_LG_5	Dinucleotide	(AT)13	26	11577667	11577692	Ca5	Dinucleotide	(AT)15	30	39537233	39537262	gene=Ca_06203;mRNA=Ca_06203.1;intron;	CTTCTTTACATCTGCTCTCCA	GCGCAAAATTCCTTTTAGTAT
Ca_GpSSR00953	scaffold10319	Dinucleotide	(TA)10	20	2086	2105	Ca5	Dinucleotide	(TA)12	24	39630429	39630452	Intergenic	GGGCATTTTAGTCATTTTACC	GGATACACATGGTGTGCTACT
Ca_GpSSR00954	Ca_LG_5	Dinucleotide	(AT)11	22	6600947	6600968	Ca5	Dinucleotide	(AT)10	20	39815598	39815617	gene=Ca_05751;mRNA=Ca_05751.1;intron;	CTTCTTTACATCTGCTCTCCA	GCGCAAAATTCCTTTTAGTAT
Ca_GpSSR00955	Ca_LG_5	Dinucleotide	(TA)23	46	6579859	6579904	Ca5	Dinucleotide	(TA)25	50	39836174	39836223	upstream:Ca_05749;	GTCCCAAAGCAATTTATTATC	CACTGCCACCGTATTATTAAA
Ca_GpSSR00956	scaffold00748	Dinucleotide	(TA)7	14	79787	79800	Ca5	Dinucleotide	(TA)8	16	40512626	40512641	Intergenic	GGGTATTTTAGACATTTCTATTC	GAAAGAGGAAGACGAGAGAG
Ca_GpSSR00957	scaffold00748	Dinucleotide	(TA)22	44	111999	112042	Ca5	Dinucleotide	(TA)25	50	40544921	40544970	gene=Ca_13902;mRNA=Ca_13902.1;intron;	TGCTAAAGTAGCAAGCCCaCAAA	cGGaAgAAGATgGTgaAGATG
Ca_GpSSR00958	Ca_LG_5	Trinucleotide	(ATT)6	18	10989647	10989664	Ca5	Trinucleotide	(ATT)5	15	40661717	40661731	gene=Ca_06155;mRNA=Ca_06155.1;intron;	CCAAACTAAATATTGCAAAC	GCACAGGACAATAAAATGAAC
Ca_GpSSR00959	Ca_LG_5	Dinucleotide	(AT)12	24	11059774	11059797	Ca5	Dinucleotide	(AT)11	22	40732157	40732178	Intergenic	CTTCTTTACATCTGCTCTCCA	GCGCAAAATTCCTTTTAGTAT
Ca_GpSSR00960	Ca_LG_5	Dinucleotide	(TA)18	36	11151147	11151182	Ca5	Dinucleotide	(TA)7	14	40819373	40819386	Intergenic	CATGCAACCTTCTTTTCAA	AGACATGGTAGATTAAAAGTGTG
Ca_GpSSR00961	scaffold00815	Dinucleotide	(AT)12	24	96590	96613	Ca5	Dinucleotide	(AT)10	20	41219553	41219572	Intergenic	TGCGTAAGAGAGGTATATGGA	GGGAGTCAGCACCATTTAT
Ca_GpSSR00962	Ca_LG_5	Dinucleotide	(TA)15	30	10584753	10584782	Ca5	Dinucleotide	(TA)16	32	41829928	41829959	Intergenic	GTCCCAAAGCAATTTATTATC	CACTGCCACCGTATTATTAAA
Ca_GpSSR00963	scaffold01290	Dinucleotide	(AT)14	28	56853	56880	Ca5	Dinucleotide	(AT)16	32	42035943	42035974	Intergenic	GTCCCAAAGCAATTTATTATC	CACTGCCACCGTATTATTAAA
Ca_GpSSR00964	Ca_LG_2	Dinucleotide	(AT)11	22	17175679	17175700	Ca5	Dinucleotide	(AT)12	24	42108270	42108293	Intergenic	ATTTGCACTCTTTGACTTTGA	TCAAATGTTTAACTCGTGGAC
Ca_GpSSR00965	Ca_LG_2	Dinucleotide	(TA)19	38	17189885	17189922	Ca5	Dinucleotide	(TA)17	34	42123377	42123410	Intergenic	GTCCCAAAGCAATTTATTATC	CACTGCCACCGTATTATTAAA
Ca_GpSSR00966	scaffold01965	Dinucleotide	(TC)7	14	7371	7384	Ca5	Dinucleotide	(TC)6	12	42279877	42279888	mRNA=Ca_16729.1;gene=Ca_16729;intron;	CAAGAACACTTGCAGTAAACC	ACAAGTGTGTGTGCGTGTA
Ca_GpSSR00967	scaffold02133	Trinucleotide	(TTA)15	45	10969	11013	Ca5	Trinucleotide	(TTA)16	48	42420569	42420616	Intergenic		
Ca_GpSSR00968	scaffold02133	Trinucleotide	(TTA)15	45	10922	10966	Ca5	Trinucleotide	(TTA)19	57	42420620	42420676	Intergenic		
Ca_GpSSR00969	Ca_LG_5	Dinucleotide	(TA)7	14	7612987	7613000	Ca5	Dinucleotide	(TA)20	40	42847034	42847073	mRNA=Ca_05848.1;gene=Ca_05848;intron;	CTTCTTTACATCTGCTCTCCA	GCGCAAAATTCCTTTTAGTAT

Ca_GpSSR00970	scaffold14993	Dinucleotide	(TA)21	42	2646	2687	Ca5	Dinucleotide	(TA)7	14	42914747	42914760	Intergenic	CATGCAACCTTCTTTTCAA	AGACATGGTAGATTAAAAGTGTG
Ca_GpSSR00971	Ca_LG_5	Dinucleotide	(AT)9	18	7947853	7947870	Ca5	Dinucleotide	(AT)8	16	43206162	43206177	upstream:Ca_05883;	CTTCTTTACATCTGCTCTCCA	GCGCAAAATTCCTTTTAGTAT
Ca_GpSSR00972	Ca_LG_5	Dinucleotide	(AT)10	20	7983757	7983776	Ca5	Dinucleotide	(AT)13	26	43242240	43242265	Intergenic	TTTTtGTTACAACCATTTTTGGAT	GAAGCtATTTTGAAtTCTTTTGTTAAA
Ca_GpSSR00973	Ca_LG_5	Dinucleotide	(TA)7	14	8108264	8108277	Ca5	Dinucleotide	(TA)6	12	43365611	43365622	Intergenic	CTTCTTTACATCTGCTCTCCA	GCGCAAAATTCCTTTTAGTAT
Ca_GpSSR00974	Ca_LG_5	Dinucleotide	(AT)20	40	8219412	8219451	Ca5	Dinucleotide	(AT)9	18	43474700	43474717	upstream:Ca_05914;	CATGCAACCTTCTTTTCAA	AGACATGGTAGATTAAAAGTGTG
Ca_GpSSR00975	scaffold09195	Dinucleotide	(AT)10	20	3664	3683	Ca5	Dinucleotide	(AT)8	16	43551687	43551702	downstream:Ca_20167;	ACGCCACTCTTTTGTTATTTT	CCAACTCATAAACACGTAACAC
Ca_GpSSR00976	Ca_LG_5	Trinucleotide	(AAT)23	69	8384037	8384105	Ca5	Trinucleotide	(AAT)18	54	43627432	43627485	Intergenic	TGGTTTTtAACAAAAaTATGTGGC	TCCCTCTTAAATTGATGGCAA
Ca_GpSSR00977	scaffold00073	Trinucleotide	(TTA)9	27	169455	169481	Ca5	Trinucleotide	(TTA)12	36	44475841	44475876	upstream:Ca_10661;		
Ca_GpSSR00978	Ca_LG_5	Dinucleotide	(AT)11	22	9818919	9818940	Ca5	Dinucleotide	(AT)10	20	45350530	45350549	upstream:Ca_06065;	CTTCTTTACATCTGCTCTCCA	GCGCAAAATTCCTTTTAGTAT
Ca_GpSSR00979	scaffold05658	Dinucleotide	(TA)20	40	1	40	Ca5	Dinucleotide	(TA)29	58	45381820	45381877	Intergenic	GTCCCAAAGCAATTTATTATC	CACTGCCACCGTATTATTAAA
Ca_GpSSR00980	Ca_LG_5	Dinucleotide	(AT)10	20	10024504	10024523	Ca5	Dinucleotide	(AT)14	28	45557068	45557095	Intergenic	TTCAAATGGAAAAAGGTAATGAAA	CCTATGAATTTTTCATGATATCCaA
Ca_GpSSR00981	Ca_LG_5	Dinucleotide	(TA)18	36	10038254	10038289	Ca5	Dinucleotide	(TA)17	34	45569593	45569626	Intergenic	GTCCCAAAGCAATTTATTATC	CACTGCCACCGTATTATTAAA
Ca_GpSSR00982	Ca_LG_5	Dinucleotide	(AT)8	16	10072741	10072756	Ca5	Dinucleotide	(AT)26	52	45603419	45603470	Intergenic	CTTCTTTACATCTGCTCTCCA	GCGCAAAATTCCTTTTAGTAT
Ca_GpSSR00983	Ca_LG_5	Dinucleotide	(TA)11	22	10085899	10085920	Ca5	Dinucleotide	(TA)12	24	45616615	45616638	Intergenic	CTTCTTTACATCTGCTCTCCA	GCGCAAAATTCCTTTTAGTAT
Ca_GpSSR00984	Ca_LG_5	Dinucleotide	(AT)14	28	9673854	9673881	Ca5	Dinucleotide	(AT)30	60	45960550	45960609	Intergenic		
Ca_GpSSR00985	Ca_LG_5	Dinucleotide	(TA)11	22	9680660	9680681	Ca5	Dinucleotide	(TA)10	20	45967396	45967415	Intergenic	CTTCTTTACATCTGCTCTCCA	GCGCAAAATTCCTTTTAGTAT
Ca_GpSSR00986	Ca_LG_5	Trinucleotide	(TTA)34	102	8575149	8575250	Ca5	Trinucleotide	(TTA)28	84	46235643	46235726	Intergenic		
Ca_GpSSR00987	Ca_LG_5	Trinucleotide	(TAT)17	51	9264554	9264604	Ca5	Trinucleotide	(TAT)19	57	46725919	46725975	Intergenic		
Ca_GpSSR00988	scaffold00580	Dinucleotide	(AT)18	36	18618	18653	Ca5	Dinucleotide	(AT)22	44	46995516	46995559	Intergenic	AATGCAACATTATGACGTAGG	CACTATTTAAACCAATGGACAA
Ca_GpSSR00989	scaffold30400	Dinucleotide	(TA)22	44	1049	1092	Ca5	Dinucleotide	(TA)8	16	47064295	47064310	Intergenic	CATGCAACCTTCTTTTCAA	AGACATGGTAGATTAAAAGTGTG
Ca_GpSSR00990	scaffold09576	Dinucleotide	(AT)18	36	7	42	Ca5	Dinucleotide	(AT)15	30	47119032	47119061	Intergenic	GTCCCAAAGCAATTTATTATC	CACTGCCACCGTATTATTAAA
Ca_GpSSR00991	Ca_LG_5	Dinucleotide	(TA)12	24	7322109	7322132	Ca5	Dinucleotide	(TA)10	20	47386244	47386263	Intergenic	CTTCTTTACATCTGCTCTCCA	GCGCAAAATTCCTTTTAGTAT
Ca_GpSSR00992	Ca_LG_5	Dinucleotide	(TA)11	22	7020694	7020715	Ca5	Dinucleotide	(TA)12	24	47689134	47689157	upstream:Ca_05793;	CTTCTTTACATCTGCTCTCCA	GCGCAAAATTCCTTTTAGTAT
Ca_GpSSR00993	scaffold48385	Dinucleotide	(AT)20	40	690	729	Ca6	Dinucleotide	(AT)19	38	30810	30847	Intergenic		
Ca_GpSSR00994	scaffold29683	Dinucleotide	(TA)20	40	1	40	Ca6	Dinucleotide	(TA)21	42	31511	31552	Intergenic		
Ca_GpSSR00995	Ca_LG_6	Dinucleotide	(TA)9	18	1847503	1847520	Ca6	Dinucleotide	(TA)11	22	443926	443947	Intergenic		
Ca_GpSSR00996	Ca_LG_6	Dinucleotide	(TA)12	24	1887260	1887283	Ca6	Dinucleotide	(TA)11	22	483614	483635	gene=Ca_06566;mRNA=Ca_06566.1;intron;		
Ca_GpSSR00997	Ca_LG_6	Dinucleotide	(AT)13	26	2022244	2022269	Ca6	Dinucleotide	(AT)23	46	620795	620840	Intergenic	CTAGAGACGTTTAGGTGGACA	GGCAATACCACCATCTAAGTA
Ca_GpSSR00998	Ca_LG_6	Dinucleotide	(TA)13	26	2074269	2074294	Ca6	Dinucleotide	(TA)11	22	665722	665743	Intergenic		
Ca_GpSSR00999	Ca_LG_6	Dinucleotide	(TA)19	38	2393396	2393433	Ca6	Dinucleotide	(TA)17	34	909790	909823	gene=Ca_06606;intron;		
Ca_GpSSR01000	Ca_LG_6	Dinucleotide	(AT)9	18	2154664	2154681	Ca6	Dinucleotide	(AT)8	16	1156232	1156247	Intergenic		
Ca_GpSSR01001	scaffold58094	Pentanucleotide	(AATAT)19	95	491	585	Ca6	Pentanucleotide	(AATAT)18	90	2375568	2375657	Intergenic		
Ca_GpSSR01002	scaffold18055	Dinucleotide	(AT)16	32	21	52	Ca6	Dinucleotide	(AT)19	38	2762482	2762519	Intergenic		
Ca_GpSSR01003	Ca_LG_6	Dinucleotide	(AT)6	12	3984494	3984505	Ca6	Dinucleotide	(AT)23	46	2902039	2902084	Intergenic	AAAGGTCGAACTTGGAGTTAC	GAATTAAGGAGCCAAGACAAC
Ca_GpSSR01004	Ca_LG_6	Dinucleotide	(AT)7	14	3925718	3925731	Ca6	Dinucleotide	(AT)9	18	2960469	2960486	Intergenic	AAAGGTCGAACTTGGAGTTAC	GAATTAAGGAGCCAAGACAAC
Ca_GpSSR01005	Ca_LG_5	Dinucleotide	(AT)10	20	337493	337512	Ca6	Dinucleotide	(AT)11	22	3073675	3073696	Intergenic	CTTCTTTACATCTGCTCTCCA	GCGCAAAATTCCTTTTAGTAT
Ca_GpSSR01006	Ca_LG_5	Dinucleotide	(TA)16	32	354884	354915	Ca6	Dinucleotide	(TA)11	22	3102481	3102502	Intergenic		
Ca_GpSSR01007	scaffold02632	Dinucleotide	(AT)12	24	8801	8824	Ca6	Dinucleotide	(AT)13	26	3184080	3184105	Intergenic	GTTCAGGTGCATTTTTAATGA	AGATGTTTTGCCAGATGTTTA
Ca_GpSSR01008	scaffold02632	Trinucleotide	(AAT)5	15	17252	17266	Ca6	Trinucleotide	(AAT)6	18	3192536	3192553	upstream:Ca_17683;	GGATAGGCCCAAAATATGTAA	GAAAGAGCTGCACTGAGATAA
Ca_GpSSR01009	scaffold00122	Dinucleotide	(AT)7	14	112360	112373	Ca6	Dinucleotide	(AT)25	50	3579475	3579524	Intergenic	TGGAGAATATTATGTGACCTGA	AGCCTTACTTCAAAAATTGGT
Ca_GpSSR01010	Ca_LG_6	Dinucleotide	(TA)12	24	2897917	2897940	Ca6	Dinucleotide	(TA)14	28	4171420	4171447	Intergenic		
Ca_GpSSR01011	Ca_LG_6	Dinucleotide	(TA)16	32	3281618	3281649	Ca6	Dinucleotide	(TA)18	36	4339493	4339528	mRNA=Ca_06672.1;gene=Ca_06672;intron;		
Ca_GpSSR01012	scaffold00032	Trinucleotide	(AAT)6	18	9485	9502	Ca6	Trinucleotide	(AAT)16	48	4784625	4784672	Intergenic	GAAATCGAAGATGATTGACAC	AAGAAAAGGGAAAAGTTGGTT
Ca_GpSSR01013	scaffold00032	Dinucleotide	(TA)9	18	48550	48567	Ca6	Dinucleotide	(TA)8	16	4827705	4827720	Intergenic	TTGGCTCTTAAGAAGAAGTGA	CGGGCAGTTTGTATTTTATAG
Ca_GpSSR01014	scaffold00032	Dinucleotide	(TA)8	16	420686	420701	Ca6	Dinucleotide	(TA)9	18	5191856	5191873	Intergenic		
Ca_GpSSR01015	scaffold00032	Dinucleotide	(TA)11	22	550166	550187	Ca6	Dinucleotide	(TA)9	18	5322407	5322424	mRNA=Ca_10647.1;gene=Ca_10647;intron;	ACTTATGAATGCCAATAAAGC	GTTTGGCCCCACTTGCTAT
Ca_GpSSR01016	Ca_LG_6	Dinucleotide	(AT)9	18	4571350	4571367	Ca6	Dinucleotide	(AT)10	20	5469397	5469416	Intergenic		
Ca_GpSSR01017	Ca_LG_6	Dinucleotide	(AT)18	36	11447254	11447289	Ca6	Dinucleotide	(AT)14	28	5596082	5596109	Intergenic		
Ca_GpSSR01018	Ca_LG_6	Dinucleotide	(AT)9	18	11249455	11249472	Ca6	Dinucleotide	(AT)7	14	5797959	5797972	Intergenic		
Ca_GpSSR01019	Ca_LG_6	Dinucleotide	(AT)11	22	10946387	10946408	Ca6	Dinucleotide	(AT)14	28	6113644	6113671	mRNA=Ca_07336.1;gene=Ca_07336;intron;	TCAAATCAACGTTGCAAAAA	GTGCGATCGTACATAACCCT
Ca_GpSSR01020	scaffold00582	Trinucleotide	(TTA)25	75	60330	60404	Ca6	Trinucleotide	(TTA)23	69	6333208	6333276	upstream:Ca_13285;	CTGAAAATGGAAAATCAATCAG	TCAACCTAATAAGAGAATGAACA
Ca_GpSSR01021	scaffold00925	Dinucleotide	(AT)9	18	40698	40715	Ca6	Dinucleotide	(AT)11	22	6985127	6985148	Intergenic	CCATTGAACATATTTCACTTCA	TTTGAAATGTCCCAATTGTC
Ca_GpSSR01022	Ca_LG_6	Trinucleotide	(TAT)13	39	5752145	5752183	Ca6	Trinucleotide	(TAT)12	36	7634600	7634635	Intergenic	GACGATTCTCCATAACTGTGA	ACAAAGGTTGTGTTTCCTAAA
Ca_GpSSR01023	Ca_LG_6	Trinucleotide	(TAT)12	36	5752187	5752222	Ca6	Trinucleotide	(TAT)13	39	7634639	7634677	Intergenic	GACGATTCTCCATAACTGTGA	ACAAAGGTTGTGTTTCCTAAA
Ca_GpSSR01024	Ca_LG_6	Dinucleotide	(AT)13	26	5756686	5756711	Ca6	Dinucleotide	(AT)25	50	7639034	7639083	gene=Ca_06880;mRNA=Ca_06880.1;intron;	AACCACATTTGTTGTCGCTG	CAAAATAAATGAAGGAGAGAATAAAGC
Ca_GpSSR01025	Ca_LG_6	Dinucleotide	(TG)6	12	5762499	5762510	Ca6	Dinucleotide	(TG)7	14	7644877	7644890	mRNA=Ca_06881.1;exon=Ca_06881.1.exon1;5'-UTR=	TCTGTTCCTTTGAGAATTGAA	CATTTCTGGCAGTACAAAGAG
Ca_GpSSR01026	scaffold02514	Dinucleotide	(AT)19	38	12	49	Ca6	Dinucleotide	(AT)12	24	7660930	7660953	Intergenic	GATACCTCATTAGATGAATGAAA	CACCCTTGAATACGGTAAAA
Ca_GpSSR01027	Ca_LG_6	Dinucleotide	(TA)11	22	5784448	5784469	Ca6	Dinucleotide	(TA)10	20	7670668	7670687	upstream:Ca_06883;		
Ca_GpSSR01028	Ca_LG_6	Dinucleotide	(AT)16	32	5843570	5843601	Ca6	Dinucleotide	(AT)25	50	7733096	7733145	Intergenic	TGAAAACATCTAAATTTCTACCG	CAGCTAACAATTAAACCTCCA
Ca_GpSSR01029	Ca_LG_6	Trinucleotide	(TTA)13	39	5914495	5914533	Ca6	Trinucleotide	(TTA)14	42	7800997	7801038	Intergenic		
Ca_GpSSR01030	Ca_LG_6	Trinucleotide	(AAT)14	42	6020547	6020588	Ca6	Trinucleotide	(AAT)17	51	7901085	7901135	Intergenic	TGAATGACAATTTTAACAAATGGA	TTTCTTCTATGTCTTTTGTGTGTCAA
Ca_GpSSR01031	Ca_LG_6	Dinucleotide	(AT)10	20	6069088	6069107	Ca6	Dinucleotide	(AT)9	18	7949812	7949829	Intergenic		
Ca_GpSSR01032	scaffold03805	Dinucleotide	(TA)16	32	21081	21112	Ca6	Dinucleotide	(TA)12	24	7969856	7969879	Intergenic	TCAATAGTGTGTTTTGTGTAAGAGATT	TgGATATTAtTTgGtCAtGtTTTGA
Ca_GpSSR01033	Ca_LG_6	Dinucleotide	(AT)9	18	6115060	6115077	Ca6	Dinucleotide	(AT)10	20	8006059	8006078	upstream:Ca_06922;		
Ca_GpSSR01034	scaffold00463	Dinucleotide	(AT)16	32	56350	56381	Ca6	Dinucleotide	(AT)21	42	8074938	8074979	Intergenic	AGTCTTGTGCAATAAATGTTGA	TACTTACGACCGACGTCAAA
Ca_GpSSR01035	Ca_LG_6	Dinucleotide	(AT)7	14	6223497	6223510	Ca6	Dinucleotide	(AT)8	16	8364912	8364927	Intergenic	AAAGGTCGAACTTGGAGTTAC	GAATTAAGGAGCCAAGACAAC
Ca_GpSSR01036	scaffold08721	Trinucleotide	(ATT)53	159	3774	3932	Ca6	Trinucleotide	(ATT)16	48	8770847	8770894	upstream:Ca_20094;		
Ca_GpSSR01037	scaffold01603	Dinucleotide	(AT)16	32	35546	35577	Ca6	Dinucleotide	(AT)21	42	8789650	8789691	Intergenic		
Ca_GpSSR01038	scaffold01603	Dinucleotide	(AT)16	32	36182	36213	Ca6	Dinucleotide	(AT)19	38	8790848	8790885	Intergenic		
Ca_GpSSR01039	Ca_LG_2	Dinucleotide	(AT)23	46	17076053	17076098	Ca6	Dinucleotide	(AT)19	38	8876651	8876688	Intergenic		
Ca_GpSSR01040	Ca_LG_7	Dinucleotide	(TA)8	16	545886	545901	Ca6	Dinucleotide	(TA)9	18	8928644	8928661	upstream:Ca_07423;		
Ca_GpSSR01041	Ca_LG_7	Trinucleotide	(TTA)10	30	595314	595343	Ca6	Trinucleotide	(TTA)12	36	8978016	8978051	Intergenic		
Ca_GpSSR01042	Ca_LG_7	Dinucleotide	(AT)11	22	609638	609659	Ca6	Dinucleotide	(AT)10	20	8992025	8992044	Intergenic		
Ca_GpSSR01043	scaffold01131	Dinucleotide	(AT)7	14	36172	36185	Ca6	Dinucleotide	(AT)6	12	9436355	9436366	Intergenic		
Ca_GpSSR01044	Ca_LG_6	Dinucleotide	(AT)10	20	6615531	6615550	Ca6	Dinucleotide	(AT)9	18	9665234	9665251	Intergenic		
Ca_GpSSR01045	Ca_LG_6	Dinucleotide	(TA)9	18	6752543	6752560	Ca6	Dinucleotide	(TA)29	58	9795289	9795346	Intergenic	TTTGTTGAATAGGTCTTGTTTTGAA	CTGATGTGGGAAAATTCCAG
Ca_GpSSR01046	Ca_LG_6	Dinucleotide	(TC)9	18	6793081	6793098	Ca6	Dinucleotide	(TC)8	16	9834493	9834508	gene=Ca_06979;mRNA=Ca_06979.1;intron;	TCCAGATTCGTCTTCTCTGTA	AGGTGAAGATGAAGAGCTACC
Ca_GpSSR01047	Ca_LG_6	Dinucleotide	(TA)15	30	6807371	6807400	Ca6	Dinucleotide	(TA)16	32	9848916	9848947	Intergenic		
Ca_GpSSR01048	Ca_LG_6	Dinucleotide	(TA)12	24	6831734	6831757	Ca6	Dinucleotide	(TA)6	12	9877757	9877768	Intergenic		
Ca_GpSSR01049	scaffold00157	Dinucleotide	(AT)23	46	23949	23994	Ca6	Dinucleotide	(AT)7	14	9927325	9927338	gene=Ca_11034;mRNA=Ca_11034.1;intron;	CCTCTTTTCACACTTCCTTTT	ATTTCTGATATGGGTCACTCC
Ca_GpSSR01050	scaffold00157	Dinucleotide	(AT)9	18	46164	46181	Ca6	Dinucleotide	(AT)8	16	9950510	9950525	Intergenic		

Ca_GpSSR01051	scaffold00157	Dinucleotide	(AT)8	16	67435	67450	Ca6	Dinucleotide	(AT)10	20	9980048	9980067	Intergenic		
Ca_GpSSR01052	scaffold08987	Dinucleotide	(AT)23	46	1	46	Ca6	Dinucleotide	(AT)13	26	10049765	10049790	Intergenic		
Ca_GpSSR01053	scaffold00157	Dinucleotide	(AT)16	32	169250	169281	Ca6	Dinucleotide	(AT)19	38	10104632	10104669	Intergenic		
Ca_GpSSR01054	Ca_LG_6	Trinucleotide	(TTA)6	18	6879397	6879414	Ca6	Trinucleotide	(TTA)7	21	10142489	10142509	Intergenic		
Ca_GpSSR01055	Ca_LG_6	Trinucleotide	(TTA)5	15	6986058	6986072	Ca6	Trinucleotide	(TTA)6	18	10249636	10249653	mRNA=Ca_07001.2;gene=Ca_07001;intron;		
Ca_GpSSR01056	Ca_LG_6	Dinucleotide	(AT)8	16	7007560	7007575	Ca6	Dinucleotide	(AT)11	22	10271556	10271577	Intergenic		
Ca_GpSSR01057	scaffold00192	Dinucleotide	(AT)9	18	38508	38525	Ca6	Dinucleotide	(AT)26	52	10369596	10369647	upstream:Ca_11212;		
Ca_GpSSR01058	scaffold00192	Trinucleotide	(TTA)8	24	89439	89462	Ca6	Trinucleotide	(TTA)6	18	10416723	10416740	Intergenic		
Ca_GpSSR01059	scaffold00192	Dinucleotide	(AT)7	14	98123	98136	Ca6	Dinucleotide	(AT)6	12	10424381	10424392	Intergenic		
Ca_GpSSR01060	scaffold00192	Dinucleotide	(AC)8	16	116902	116917	Ca6	Dinucleotide	(AC)7	14	10443149	10443162	gene=Ca_11221;mRNA=Ca_11221.1;intron;	GTTTCACGAAAGATTGTTCAG	GCTACCCAGAGAAAAACCTAT
Ca_GpSSR01061	scaffold00192	Dinucleotide	(AT)13	26	214154	214179	Ca6	Dinucleotide	(AT)17	34	10551167	10551200	mRNA=Ca_11230.1;gene=Ca_11230;intron;	CACCACCTAGAGATGATAAAACT	GTGTCAAATTTACGTTTTCA
Ca_GpSSR01062	scaffold10995	Dinucleotide	(AT)8	16	2768	2783	Ca6	Dinucleotide	(AT)7	14	10564543	10564556	Intergenic	GATGATATGCATCCAAGTTTT	TCACAAACTTCTTGAAAAACAC
Ca_GpSSR01063	Ca_LG_6	Trinucleotide	(TAA)18	54	4835874	4835927	Ca6	Trinucleotide	(TAA)17	51	10716214	10716264	upstream:Ca_06829;		
Ca_GpSSR01064	Ca_LG_6	Dinucleotide	(TA)11	22	4849106	4849127	Ca6	Dinucleotide	(TA)10	20	10729448	10729467	Intergenic		
Ca_GpSSR01065	scaffold141681	Dinucleotide	(TA)14	28	233	260	Ca6	Dinucleotide	(TA)13	26	10948209	10948234	Intergenic		
Ca_GpSSR01066	scaffold58402	Dinucleotide	(AT)19	38	1	38	Ca6	Dinucleotide	(AT)26	52	10961707	10961758	Intergenic		
Ca_GpSSR01067	Ca_LG_6	Trinucleotide	(ATA)29	87	7442254	7442340	Ca6	Trinucleotide	(ATA)19	57	11038836	11038892	Intergenic	GAGCTCAATAATTGGATTAGATTTATG	TGGTACAAAATACCAGGATCAA
Ca_GpSSR01068	scaffold01465	Trinucleotide	(AAT)8	24	33413	33436	Ca6	Trinucleotide	(AAT)7	21	11358290	11358310	Intergenic	GAAATCGAAGATGATTGACAC	AAGAAAAGGGAAAAGTTGGTT
Ca_GpSSR01069	scaffold02329	Dinucleotide	(AT)11	22	18357	18378	Ca6	Dinucleotide	(AT)10	20	11484093	11484112	Intergenic	GCTCTTTAATGATGAATCAAAAC	GCTTAGCAAATCCAAAAATCT
Ca_GpSSR01070	scaffold09587	Dinucleotide	(TA)8	16	5134	5149	Ca6	Dinucleotide	(TA)7	14	11500818	11500831	Intergenic	AATCACATCACTCACTTCTCAA	TTGAACTTAGGTTGGCTAGAA
Ca_GpSSR01071	scaffold00364	Dinucleotide	(AT)7	14	141179	141192	Ca6	Dinucleotide	(AT)15	30	11933023	11933052	Intergenic		
Ca_GpSSR01072	scaffold01886	Tetranucleotide	(TTCT)19	76	6987	7062	Ca6	Tetranucleotide	(TTCT)15	60	12269011	12269070	Intergenic	GCCCAGTCTCTCCCTCTTTC	ACGTCACTCCCTTCCACAAA
Ca_GpSSR01073	scaffold00804	Dinucleotide	(TA)11	22	115088	115109	Ca6	Dinucleotide	(TA)7	14	12634831	12634844	Intergenic	AACCGTTACTCTAACCATTCA	TTGGAAAGTGTATCAAACGAT
Ca_GpSSR01074	scaffold20415	Dinucleotide	(TA)8	16	1587	1602	Ca6	Dinucleotide	(TA)7	14	12775089	12775102	Intergenic		
Ca_GpSSR01075	Ca_LG_6	Trinucleotide	(TGC)6	18	7893464	7893481	Ca6	Trinucleotide	(TGC)5	15	12908240	12908254	mRNA=Ca_07074.1;gene=Ca_07074;exon=Ca_07074.	GAGACAGAGAATTGGACTGAA	GGTGAAAATCAGAATCATCAG
Ca_GpSSR01076	Ca_LG_6	Dinucleotide	(TC)8	16	7917964	7917979	Ca6	Dinucleotide	(TC)7	14	12935212	12935225	upstream:Ca_07076;	TCCAGATTCGTCTTCTCTGTA	AGGTGAAGATGAAGAGCTACC
Ca_GpSSR01077	Ca_LG_6	Dinucleotide	(TA)8	16	7941242	7941257	Ca6	Dinucleotide	(TA)7	14	12958421	12958434	upstream:Ca_07078;		
Ca_GpSSR01078	Ca_LG_6	Dinucleotide	(TA)9	18	7985866	7985883	Ca6	Dinucleotide	(TA)10	20	13003026	13003045	upstream:Ca_07083;		
Ca_GpSSR01079	scaffold11772	Dinucleotide	(AT)20	40	1	40	Ca6	Dinucleotide	(AT)33	66	13354839	13354904	Intergenic		
Ca_GpSSR01080	Ca_LG_6	Dinucleotide	(TA)6	12	8715974	8715985	Ca6	Dinucleotide	(TA)26	52	13550243	13550294	upstream:Ca_07136;	AAAGGTCGAACTTGGAGTTAC	GAATTAAGGAGCCAAGACAAC
Ca_GpSSR01081	scaffold10777	Dinucleotide	(TA)12	24	1	24	Ca6	Dinucleotide	(TA)10	20	13675433	13675452	Intergenic		
Ca_GpSSR01082	Ca_LG_7	Dinucleotide	(AT)9	18	730971	730988	Ca6	Dinucleotide	(AT)8	16	14164386	14164401	Intergenic		
Ca_GpSSR01083	scaffold25641	Dinucleotide	(AT)7	14	1	14	Ca6	Dinucleotide	(AT)17	34	14260814	14260847	Intergenic		
Ca_GpSSR01084	Ca_LG_6	Dinucleotide	(AT)18	36	10727121	10727156	Ca6	Dinucleotide	(AT)25	50	14786648	14786697	upstream:Ca_07317;	TGAAAACATCTAAATTTCTACCG	CAGCTAACAATTAAACCTCCA
Ca_GpSSR01085	scaffold06215	Dinucleotide	(AT)13	26	759	784	Ca6	Dinucleotide	(AT)17	34	15073083	15073116	upstream:Ca_19605;	TTTCGATAGTGTCAAATGAA	ACGGGTAGAAGAAAAAGAGTC
Ca_GpSSR01086	scaffold00608	Dinucleotide	(AT)19	38	82669	82706	Ca6	Dinucleotide	(AT)21	42	15892065	15892106	Intergenic	TCCTAGTGTGAAATCAAGGTG	GTGGAGCACCATCTAACAGTA
Ca_GpSSR01087	scaffold02744	Dinucleotide	(AT)13	26	3710	3735	Ca6	Dinucleotide	(AT)14	28	16049988	16050015	Intergenic		
Ca_GpSSR01088	Ca_LG_6	Dinucleotide	(AT)15	30	8448845	8448874	Ca6	Dinucleotide	(AT)12	24	16339488	16339511	Intergenic		
Ca_GpSSR01089	Ca_LG_6	Trinucleotide	(TAT)21	63	8487238	8487300	Ca6	Trinucleotide	(TAT)17	51	16372440	16372490	Intergenic	TTCTGCTTTCCAACCAAaGA	AGTCAAAGTGAATCCGTGATGA
Ca_GpSSR01090	Ca_LG_6	Dinucleotide	(AT)15	30	8512479	8512508	Ca6	Dinucleotide	(AT)12	24	16397769	16397792	upstream:Ca_07118;	CAtGcGAaTttAGgAagaGAGA	aGACATGGAGAGACCAAACAA
Ca_GpSSR01091	Ca_LG_6	Dinucleotide	(TA)6	12	10491425	10491436	Ca6	Dinucleotide	(TA)30	60	16527609	16527668	Intergenic	AAAGGTCGAACTTGGAGTTAC	GAATTAAGGAGCCAAGACAAC
Ca_GpSSR01092	Ca_LG_6	Dinucleotide	(AT)10	20	10440926	10440945	Ca6	Dinucleotide	(AT)9	18	16582791	16582808	gene=Ca_07287;mRNA=Ca_07287.1;intron;		
Ca_GpSSR01093	Ca_LG_6	Dinucleotide	(AT)12	24	10214331	10214354	Ca6	Dinucleotide	(AT)10	20	16596952	16596971	upstream:Ca_07262;		
Ca_GpSSR01094	Ca_LG_6	Dinucleotide	(AT)11	22	10329778	10329799	Ca6	Dinucleotide	(AT)10	20	16710890	16710909	upstream:Ca_07274;		
Ca_GpSSR01095	Ca_LG_2	Dinucleotide	(AT)20	40	13727583	13727622	Ca6	Dinucleotide	(AT)9	18	16909469	16909486	gene=Ca_01888;mRNA=Ca_01888.1;intron;		
Ca_GpSSR01096	scaffold05938	Dinucleotide	(AT)19	38	1	38	Ca6	Dinucleotide	(AT)10	20	16938428	16938447	Intergenic		
Ca_GpSSR01097	scaffold00918	Dinucleotide	(TA)12	24	101615	101638	Ca6	Dinucleotide	(TA)10	20	16990147	16990166	Intergenic	TTCTTCGTTGTTGTCTACCTC	GTTCAACCTGATTTCTTCCTT
Ca_GpSSR01098	scaffold00918	Dinucleotide	(TA)13	26	44439	44464	Ca6	Dinucleotide	(TA)18	36	17051356	17051391	Intergenic	TTCTTCGTTGTTGTCTACCTC	GTTCAACCTGATTTCTTCCTT
Ca_GpSSR01099	scaffold00918	Dinucleotide	(TA)7	14	9548	9561	Ca6	Dinucleotide	(TA)9	18	17084796	17084813	gene=Ca_14454;mRNA=Ca_14454.1;intron;	TTCTTCGTTGTTGTCTACCTC	GTTCAACCTGATTTCTTCCTT
Ca_GpSSR01100	scaffold08791	Trinucleotide	(TTA)18	54	3269	3322	Ca6	Trinucleotide	(TTA)30	90	17249296	17249385	Intergenic	GACCATCAATCACAGAGAGAA	TTTATTACGTACCCACTAGACAA
Ca_GpSSR01101	scaffold82223	Dinucleotide	(AT)21	42	412	453	Ca6	Dinucleotide	(AT)12	24	17301512	17301535	Intergenic		
Ca_GpSSR01102	scaffold00526	Trinucleotide	(TTA)26	78	150872	150949	Ca6	Trinucleotide	(TTA)18	54	17385162	17385215	Intergenic	TTCTCTTTTTCCCATTAATCC	CATCCCTCGTTAGCATAAATA
Ca_GpSSR01103	Ca_LG_6	Dinucleotide	(AT)8	16	6314907	6314922	Ca6	Dinucleotide	(AT)7	14	17503156	17503169	gene=Ca_06937;mRNA=Ca_06937.1;intron;		
Ca_GpSSR01104	scaffold00157	Dinucleotide	(AT)10	20	204801	204820	Ca6	Dinucleotide	(AT)12	24	17580706	17580729	Intergenic		
Ca_GpSSR01105	scaffold00262	Dinucleotide	(AT)21	42	10324	10365	Ca6	Dinucleotide	(AT)19	38	17901357	17901394	upstream:Ca_11635;		
Ca_GpSSR01106	scaffold36986	Trinucleotide	(TAT)5	15	907	921	Ca6	Trinucleotide	(TAT)15	45	17934034	17934078	Intergenic	GACGATTCTCCATAACTGTGA	ACAAAGGTTGTGTTTCCTAAA
Ca_GpSSR01107	scaffold01878	Dinucleotide	(TA)11	22	30788	30809	Ca6	Dinucleotide	(TA)10	20	17966625	17966644	Intergenic		
Ca_GpSSR01108	scaffold39172	Dinucleotide	(TA)8	16	801	816	Ca6	Dinucleotide	(TA)6	12	18175012	18175023	Intergenic		
Ca_GpSSR01109	Ca_LG_2	Dinucleotide	(AT)9	18	15041589	15041606	Ca6	Dinucleotide	(AT)8	16	18207035	18207050	mRNA=Ca_01995.2;gene=Ca_01995;intron;	ATTTGCACTCTTTGACTTTGA	TCAAATGTTTAACTCGTGGAC
Ca_GpSSR01110	Ca_LG_2	Dinucleotide	(AT)9	18	14882003	14882020	Ca6	Dinucleotide	(AT)10	20	18368643	18368662	Intergenic	ATTTGCACTCTTTGACTTTGA	TCAAATGTTTAACTCGTGGAC
Ca_GpSSR01111	scaffold07242	Dinucleotide	(TA)10	20	5503	5522	Ca6	Dinucleotide	(TA)12	24	18423211	18423234	Intergenic	TATATCGCGGGATGTATTTTA	AATGCTCAAGTGCTATGAAAA
Ca_GpSSR01112	scaffold49486	Trinucleotide	(ATA)23	69	17	85	Ca6	Trinucleotide	(ATA)19	57	18458254	18458310	Intergenic		
Ca_GpSSR01113	scaffold00266	Dinucleotide	(AT)15	30	122274	122303	Ca6	Dinucleotide	(AT)16	32	18540093	18540124	Intergenic		
Ca_GpSSR01114	Ca_LG_2	Dinucleotide	(AT)10	20	15362893	15362912	Ca6	Dinucleotide	(AT)11	22	19063000	19063021	Intergenic	ATTTGCACTCTTTGACTTTGA	TCAAATGTTTAACTCGTGGAC
Ca_GpSSR01115	Ca_LG_2	Dinucleotide	(AT)12	24	14364118	14364141	Ca6	Dinucleotide	(AT)11	22	19351210	19351231	Intergenic		
Ca_GpSSR01116	Ca_LG_2	Dinucleotide	(AT)11	22	14452258	14452279	Ca6	Dinucleotide	(AT)18	36	19439669	19439704	upstream:Ca_01945;	ATTTGCACTCTTTGACTTTGA	TCAAATGTTTAACTCGTGGAC
Ca_GpSSR01117	scaffold03622	Trinucleotide	(AAT)28	84	11728	11811	Ca6	Trinucleotide	(AAT)22	66	19583902	19583967	Intergenic	AATAGTTTCGGAATTTCCTTG	GTTAGTTAGTTAGGTTTTCGTTT
Ca_GpSSR01118	Ca_LG_2	Dinucleotide	(TA)11	22	14688427	14688448	Ca6	Dinucleotide	(TA)10	20	19680353	19680372	Intergenic	ATTTGCACTCTTTGACTTTGA	TCAAATGTTTAACTCGTGGAC
Ca_GpSSR01119	Ca_LG_2	Pentanucleotide	(ATATT)38	190	14722440	14722629	Ca6	Pentanucleotide	(ATATT)11	55	19718572	19718626	Intergenic		
Ca_GpSSR01120	scaffold00811	Trinucleotide	(TTA)21	63	158684	158746	Ca6	Trinucleotide	(TTA)35	105	19971945	19972049	Intergenic		
Ca_GpSSR01121	Ca_LG_2	Dinucleotide	(TA)12	24	13971642	13971665	Ca6	Dinucleotide	(TA)6	12	20958755	20958766	Intergenic		
Ca_GpSSR01122	Ca_LG_2	Trinucleotide	(ATA)18	54	14015310	14015363	Ca6	Trinucleotide	(ATA)21	63	21006929	21006991	Intergenic		
Ca_GpSSR01123	Ca_LG_2	Dinucleotide	(AT)12	24	14077816	14077839	Ca6	Dinucleotide	(AT)14	28	21071110	21071137	Intergenic		
Ca_GpSSR01124	scaffold44937	Dinucleotide	(AT)23	46	735	780	Ca6	Dinucleotide	(AT)28	56	21291882	21291937	Intergenic	AGCACTTGTTAACGTTTTCTT	GTCAATCTATTGTGATGTTTTTG
Ca_GpSSR01125	scaffold19366	Dinucleotide	(TA)14	28	1727	1754	Ca6	Dinucleotide	(TA)7	14	21374770	21374783	Intergenic		
Ca_GpSSR01126	scaffold00233	Dinucleotide	(TA)10	20	117468	117487	Ca6	Dinucleotide	(TA)9	18	21605986	21606003	gene=Ca_08986;mRNA=Ca_08986.1;intron;		
Ca_GpSSR01127	scaffold00277	Dinucleotide	(AT)15	30	208838	208867	Ca6	Dinucleotide	(AT)18	36	21739442	21739477	Intergenic	AAACCACAATCAAAATGTGAC	AGATTCTAATAGAGGAAAACAAG
Ca_GpSSR01128	scaffold00277	Dinucleotide	(TA)12	24	208318	208341	Ca6	Dinucleotide	(TA)13	26	21739974	21739999	Intergenic	AAACCACAATCAAAATGTGAC	AGATTCTAATAGAGGAAAACAAG
Ca_GpSSR01129	scaffold00277	Dinucleotide	(AT)16	32	141930	141961	Ca6	Dinucleotide	(AT)14	28	21815345	21815372	Intergenic	AAACCACAATCAAAATGTGAC	AGATTCTAATAGAGGAAAACAAG
Ca_GpSSR01130	scaffold00277	Dinucleotide	(AT)8	16	136137	136152	Ca6	Dinucleotide	(AT)22	44	21817312	21817355	Intergenic	AAGCTTTGTGAAAGGGATATT	CGCTTACAAAATTTGCTAGTTA
Ca_GpSSR01131	scaffold00277	Dinucleotide	(TA)27	54	33574	33627	Ca6	Dinucleotide	(TA)17	34	21922928	21922961	Intergenic		

Ca_GpSSR01132	scaffold00277	Dinucleotide	(AT)16	32	27618	27649	Ca6	Dinucleotide	(AT)6	12	21928555	21928566	Intergenic	AAACCACAATCAAAATGTGAC	AGATTCTAATAGAGGAAAACAAG
Ca_GpSSR01133	scaffold00850	Dinucleotide	(AT)16	32	90023	90054	Ca6	Dinucleotide	(AT)19	38	22078094	22078131	upstream:Ca_14276;	CGCATATACTTCCTCAATACAA	AGAGAAGTTTTATGAACATTTGC
Ca_GpSSR01134	scaffold09107	Dinucleotide	(AT)6	12	3712	3723	Ca6	Dinucleotide	(AT)12	24	22163951	22163974	Intergenic	CAGTGACATTTCATTTTGTCTCC	TCGAGATTAGTTTGTTTATTCTTGGA
Ca_GpSSR01135	scaffold46650	Trinucleotide	(TAT)7	21	149	169	Ca6	Trinucleotide	(TAT)6	18	22167121	22167138	Intergenic	GACGATTCTCCATAACTGTGA	ACAAAGGTTGTGTTTCCTAAA
Ca_GpSSR01136	scaffold00173	Dinucleotide	(AT)11	22	6670	6691	Ca6	Dinucleotide	(TA)10	20	22477291	22477310	Intergenic	TTGTCACTTGTGTTTTCTTGA	TCACTAACCCATAAACAATGC
Ca_GpSSR01137	scaffold16760	Trinucleotide	(TTA)29	87	1939	2025	Ca6	Trinucleotide	(TTA)25	75	22733499	22733573	Intergenic	GCAAGTGAATGTGAAGTAGAAA	GAGCGTGTACTATACAACTACTG
Ca_GpSSR01138	Ca_LG_5	Dinucleotide	(AT)10	20	12566895	12566914	Ca6	Dinucleotide	(AT)13	26	22824829	22824854	Intergenic	CTTCTTTACATCTGCTCTCCA	GCGCAAAATTCCTTTTAGTAT
Ca_GpSSR01139	scaffold00173	Dinucleotide	(TA)12	24	103666	103689	Ca6	Dinucleotide	(TA)11	22	22899311	22899332	Intergenic		
Ca_GpSSR01140	scaffold00173	Dinucleotide	(AT)9	18	167748	167765	Ca6	Dinucleotide	(AT)8	16	22962832	22962847	Intergenic	GTACCGGGGTATAGTGAAAAT	CACTAAGCTGGTTTGTGATTC
Ca_GpSSR01141	scaffold00173	Trinucleotide	(ATA)23	69	208703	208771	Ca6	Trinucleotide	(ATA)19	57	23003198	23003254	upstream:Ca_11178;	AAGATACCAAACCTTTCCTTG	GTGTTAATGAAGGGGTATTTTT
Ca_GpSSR01142	scaffold03330	Dinucleotide	(TA)17	34	4019	4052	Ca6	Dinucleotide	(TA)21	42	23086414	23086455	Intergenic		
Ca_GpSSR01143	Ca_LG_2	Dinucleotide	(AT)9	18	13185166	13185183	Ca6	Dinucleotide	(AT)18	36	23107468	23107503	Intergenic	ATTTGCACTCTTTGACTTTGA	TCAAATGTTTAACTCGTGGAC
Ca_GpSSR01144	scaffold00782	Dinucleotide	(TA)23	46	17638	17683	Ca6	Dinucleotide	(TA)18	36	23439829	23439864	Intergenic		
Ca_GpSSR01145	scaffold01520	Trinucleotide	(AAT)5	15	42829	42843	Ca6	Trinucleotide	(AAT)16	48	23501072	23501119	Intergenic	GAAATCGAAGATGATTGACAC	AAGAAAAGGGAAAAGTTGGTT
Ca_GpSSR01146	scaffold00481	Dinucleotide	(TA)9	18	61602	61619	Ca6	Dinucleotide	(TA)10	20	23817223	23817242	Intergenic	ACCATCAACTGATGTATCCAC	GGTTTGAATGAGAGATAAAATTG
Ca_GpSSR01147	scaffold00296	Dinucleotide	(AT)10	20	72675	72694	Ca6	Dinucleotide	(AT)21	42	24249940	24249981	Intergenic		
Ca_GpSSR01148	scaffold00296	Dinucleotide	(AT)8	16	175960	175975	Ca6	Dinucleotide	(AT)9	18	24359507	24359524	Intergenic		
Ca_GpSSR01149	scaffold00296	Trinucleotide	(TTA)18	54	185825	185878	Ca6	Trinucleotide	(TTA)20	60	24368115	24368174	Intergenic	CTTAATTTCCCAAAAATCACC	TGACTCGTGTGTTGACACTTA
Ca_GpSSR01150	scaffold00296	Trinucleotide	(ATT)5	15	207458	207472	Ca6	Trinucleotide	(ATT)6	18	24390114	24390131	Intergenic		
Ca_GpSSR01151	scaffold01394	Dinucleotide	(TA)17	34	47366	47399	Ca6	Dinucleotide	(TA)12	24	24459207	24459230	Intergenic	AATCACACCACAAATTTTGAC	TGCAGTAAAGTCCATCTTAGG
Ca_GpSSR01152	scaffold01170	Trinucleotide	(TAT)18	54	20480	20533	Ca6	Trinucleotide	(TAT)15	45	24608315	24608359	Intergenic	GACGATTCTCCATAACTGTGA	ACAAAGGTTGTGTTTCCTAAA
Ca_GpSSR01153	scaffold00703	Dinucleotide	(AT)7	14	21154	21167	Ca6	Dinucleotide	(AT)8	16	26460342	26460357	Intergenic	CTCCGTTTGGAAGGATTT	GCCTTATTCAGAAGAAAACCA
Ca_GpSSR01154	Ca_LG_2	Dinucleotide	(TC)10	20	8340012	8340031	Ca6	Dinucleotide	(TC)8	16	26986245	26986260	5'-UTR=Ca_01665.1.5'-UTR1;mRNA=Ca_01665.1;exon	TCTGGCAGATGAAGATGTAAC	TAACAGACCAAAAACTCCAAA
Ca_GpSSR01155	Ca_LG_2	Trinucleotide	(ATT)22	66	8394979	8395044	Ca6	Trinucleotide	(ATT)21	63	27050310	27050372	Intergenic		
Ca_GpSSR01156	Ca_LG_2	Dinucleotide	(AT)11	22	8611652	8611673	Ca6	Dinucleotide	(AT)25	50	27273612	27273661	Intergenic	ATTTGCACTCTTTGACTTTGA	TCAAATGTTTAACTCGTGGAC
Ca_GpSSR01157	scaffold00555	Dinucleotide	(AT)12	24	130008	130031	Ca6	Dinucleotide	(AT)10	20	27773203	27773222	Intergenic		
Ca_GpSSR01158	scaffold00555	Dinucleotide	(AT)12	24	95048	95071	Ca6	Dinucleotide	(AT)9	18	27808765	27808782	Intergenic		
Ca_GpSSR01159	Ca_LG_5	Dinucleotide	(AT)9	18	15598005	15598022	Ca6	Dinucleotide	(AT)10	20	28188244	28188263	Intergenic	CTTCTTTACATCTGCTCTCCA	GCGCAAAATTCCTTTTAGTAT
Ca_GpSSR01160	scaffold00490	Dinucleotide	(AT)15	30	85504	85533	Ca6	Dinucleotide	(AT)16	32	28632280	28632311	Intergenic		
Ca_GpSSR01161	scaffold00490	Dinucleotide	(AT)9	18	132681	132698	Ca6	Dinucleotide	(AT)10	20	28683624	28683643	Intergenic		
Ca_GpSSR01162	scaffold00606	Dinucleotide	(AT)11	22	113104	113125	Ca6	Dinucleotide	(AT)12	24	28935910	28935933	Intergenic	GTATTCCTGAAATGCGACA	CTCGGTTTATCCTCAAATTCT
Ca_GpSSR01163	scaffold00606	Dinucleotide	(AT)13	26	97625	97650	Ca6	Dinucleotide	(AT)12	24	28949518	28949541	mRNA=Ca_13375.1;gene=Ca_13375;intron;	GTATTCCTGAAATGCGACA	CTCGGTTTATCCTCAAATTCT
Ca_GpSSR01164	scaffold73268	Trinucleotide	(ATT)32	96	411	506	Ca6	Trinucleotide	(ATT)18	54	28966841	28966894	Intergenic		
Ca_GpSSR01165	scaffold01145	Dinucleotide	(TA)14	28	41780	41807	Ca6	Dinucleotide	(TA)19	38	29128099	29128136	Intergenic	AATTGTGGGAATGGAGTAAAT	CGTATATAGGCGGATAATTCA
Ca_GpSSR01166	scaffold01145	Dinucleotide	(AT)7	14	82568	82581	Ca6	Dinucleotide	(AT)6	12	29174757	29174768	Intergenic	AATTGTGGGAATGGAGTAAAT	CGTATATAGGCGGATAATTCA
Ca_GpSSR01167	scaffold00666	Dinucleotide	(AT)11	22	108520	108541	Ca6	Dinucleotide	(AT)8	16	29313257	29313272	Intergenic	AGCTGGCTCATTTACTCTTTT	CCCTCCCTAGGTTCTTTTTA
Ca_GpSSR01168	scaffold00666	Dinucleotide	(AT)15	30	103848	103877	Ca6	Dinucleotide	(AT)14	28	29317329	29317356	Intergenic	AGCTGGCTCATTTACTCTTTT	CCCTCCCTAGGTTCTTTTTA
Ca_GpSSR01169	scaffold00167	Dinucleotide	(AT)18	36	87309	87344	Ca6	Dinucleotide	(AT)23	46	29947310	29947355	Intergenic	CTAGAGACGTTTAGGTGGACA	GGCAATACCACCATCTAAGTA
Ca_GpSSR01170	scaffold03352	Dinucleotide	(AC)10	20	932	951	Ca6	Dinucleotide	(AC)9	18	29991078	29991095	Intergenic	AGAGTACCCATGATTGATCTTT	CTTTTGCATTCGATTCTTTT
Ca_GpSSR01171	scaffold01041	Dinucleotide	(AT)7	14	11979	11992	Ca6	Dinucleotide	(AT)6	12	30054258	30054269	Intergenic		
Ca_GpSSR01172	scaffold44025	Dinucleotide	(AT)16	32	676	707	Ca6	Dinucleotide	(AT)24	48	30106548	30106595	Intergenic	CTAGAGACGTTTAGGTGGACA	GGCAATACCACCATCTAAGTA
Ca_GpSSR01173	scaffold03171	Dinucleotide	(TA)11	22	21735	21756	Ca6	Dinucleotide	(TA)25	50	30333189	30333238	Intergenic	TTACCCCTTGTTCTATAATTATTTTGT	TCACCACAATTAGAACAAATTACCA
Ca_GpSSR01174	scaffold01835	Dinucleotide	(AT)8	16	3680	3695	Ca6	Dinucleotide	(AT)9	18	30594887	30594904	Intergenic	CATATAAAATCACCATCACCAC	GAAAGACTTTTGTCCCATTTT
Ca_GpSSR01175	scaffold00079	Dinucleotide	(AT)12	24	69859	69882	Ca6	Dinucleotide	(AT)32	64	30738349	30738412	Intergenic	CATTTGTTCATCTACATGGATT	CGTATGCTTCAACGTCATAGT
Ca_GpSSR01176	scaffold00079	Dinucleotide	(AT)11	22	125224	125245	Ca6	Dinucleotide	(AT)19	38	30790799	30790836	Intergenic	CATTTGTTCATCTACATGGATT	CGTATGCTTCAACGTCATAGT
Ca_GpSSR01177	scaffold00079	Dinucleotide	(AT)21	42	184182	184223	Ca6	Dinucleotide	(AT)31	62	30855512	30855573	Intergenic		
Ca_GpSSR01178	scaffold00079	Dinucleotide	(AT)19	38	221792	221829	Ca6	Dinucleotide	(AT)23	46	30906455	30906500	Intergenic	CTAGAGACGTTTAGGTGGACA	GGCAATACCACCATCTAAGTA
Ca_GpSSR01179	scaffold00079	Dinucleotide	(TA)16	32	247902	247933	Ca6	Dinucleotide	(TA)18	36	30933302	30933337	Intergenic		
Ca_GpSSR01180	scaffold00079	Dinucleotide	(TA)24	48	289381	289428	Ca6	Dinucleotide	(TA)7	14	30981952	30981965	Intergenic		
Ca_GpSSR01181	scaffold06752	Dinucleotide	(TA)14	28	7947	7974	Ca6	Dinucleotide	(TA)9	18	31271189	31271206	Intergenic	GGGggCAtGTTTtATTTGtA	CGCTTCCCAACTACTTGTTT
Ca_GpSSR01182	scaffold02336	Dinucleotide	(TA)14	28	27991	28018	Ca6	Dinucleotide	(TA)12	24	31727075	31727098	Intergenic	GCGTTTTCTCTGAACAAATAG	TGAAGTTAAAGTCGAAGAAATG
Ca_GpSSR01183	scaffold00624	Dinucleotide	(AT)8	16	117341	117356	Ca6	Dinucleotide	(AT)9	18	32280884	32280901	Intergenic	TGGATATAATGTTTGTTCCGTA	AGACAAATGTTGTCACTCGAC
Ca_GpSSR01184	scaffold00624	Dinucleotide	(AT)15	30	95594	95623	Ca6	Dinucleotide	(AT)18	36	32300885	32300920	Intergenic		
Ca_GpSSR01185	scaffold01022	Dinucleotide	(AT)19	38	40234	40271	Ca6	Dinucleotide	(AT)16	32	32459602	32459633	Intergenic		
Ca_GpSSR01186	scaffold00658	Dinucleotide	(AT)12	24	111912	111935	Ca6	Dinucleotide	(AT)13	26	32650587	32650612	Intergenic		
Ca_GpSSR01187	scaffold01658	Dinucleotide	(AT)9	18	33734	33751	Ca6	Dinucleotide	(AT)8	16	32893838	32893853	gene=Ca_16235;mRNA=Ca_16235.1;intron;	AATGTTATCGACCTGCAAAT	CATCACAATACGTCATCATTTT
Ca_GpSSR01188	scaffold02281	Dinucleotide	(TA)10	20	39285	39304	Ca6	Dinucleotide	(TA)11	22	33864651	33864672	Intergenic	ACTCTTAATCATGACCATCCA	TGGTAGACCTTGTATCTCACC
Ca_GpSSR01189	scaffold04876	Hexanucleotide	(TTTATA)18	108	12060	12167	Ca6	Hexanucleotide	(TTTATA)9	54	33908470	33908523	upstream:Ca_19132;		
Ca_GpSSR01190	scaffold05124	Dinucleotide	(AT)6	12	6833	6844	Ca6	Dinucleotide	(AT)9	18	34206368	34206385	Intergenic		
Ca_GpSSR01191	scaffold01715	Dinucleotide	(TA)19	38	57483	57520	Ca6	Dinucleotide	(TA)6	12	34422100	34422111	Intergenic	GTGCCAATTTATTTGGAAAA	TGCTACGTCCATTTTTAGAAG
Ca_GpSSR01192	scaffold01566	Dinucleotide	(AT)17	34	157433	157466	Ca6	Dinucleotide	(AT)24	48	34770828	34770875	upstream:Ca_10319;	CTAGAGACGTTTAGGTGGACA	GGCAATACCACCATCTAAGTA
Ca_GpSSR01193	scaffold00693	Dinucleotide	(AT)11	22	41500	41521	Ca6	Dinucleotide	(AT)12	24	34856534	34856557	mRNA=Ca_13710.1;gene=Ca_13710;intron;		
Ca_GpSSR01194	Ca_LG_2	Dinucleotide	(TA)11	22	10367024	10367045	Ca6	Dinucleotide	(TA)29	58	35049925	35049982	Intergenic	ATTTGCACTCTTTGACTTTGA	TCAAATGTTTAACTCGTGGAC
Ca_GpSSR01195	scaffold02535	Dinucleotide	(AT)9	18	4982	4999	Ca6	Dinucleotide	(AT)10	20	35681525	35681544	Intergenic	ATCAGGTCTCTTTTGCTCTCT	CAGTTTACTCAGTACCTGTTCA
Ca_GpSSR01196	scaffold02574	Trinucleotide	(TTA)14	42	4257	4298	Ca6	Trinucleotide	(TTA)12	36	36951266	36951301	Intergenic		
Ca_GpSSR01197	scaffold03807	Tetranucleotide	(TTTA)5	20	22233	22252	Ca6	Tetranucleotide	(TTTA)6	24	37132272	37132295	Intergenic	CATGTGACTCTTTTTAATGTTTG	ACTACTTGTGTCCTAACAACTCA
Ca_GpSSR01198	scaffold00894	Trinucleotide	(TAT)7	21	22770	22790	Ca6	Trinucleotide	(TAT)6	18	37387170	37387187	Intergenic	AATCCAAAACCTTGATTGAA	GCAAATATGGTTTGATTTGAC
Ca_GpSSR01199	scaffold01712	Dinucleotide	(TA)15	30	13058	13087	Ca6	Dinucleotide	(TA)19	38	37497801	37497838	Intergenic	TTTTGCTATCCTCACAGTTTT	ATCTTGGAGGAACTCTCAATC
Ca_GpSSR01200	scaffold01974	Trinucleotide	(TTA)19	57	1004	1060	Ca6	Trinucleotide	(TTA)13	39	38318204	38318242	Intergenic		
Ca_GpSSR01201	scaffold02042	Dinucleotide	(AT)15	30	36549	36578	Ca6	Dinucleotide	(AT)18	36	39428374	39428409	Intergenic	AACAAAAATAAGAATGGCGTGAA	GCTGAACGGTGCTTTTTAGG
Ca_GpSSR01202	scaffold05841	Trinucleotide	(TTG)13	39	933	971	Ca6	Trinucleotide	(TTG)15	45	39475329	39475373	Intergenic		
Ca_GpSSR01203	scaffold132923	Dinucleotide	(AT)12	24	107	130	Ca6	Dinucleotide	(AT)11	22	39484510	39484531	Intergenic	ATTATTTAATCGACCGACGAG	GCCCCTAACTCAAATTTCTAC
Ca_GpSSR01204	scaffold03439	Dinucleotide	(TA)12	24	25362	25385	Ca6	Dinucleotide	(TA)11	22	39892557	39892578	Intergenic	TAATGGAAAAATGGAAAGCA	TGGAGTCAAAACGATTTTATG
Ca_GpSSR01205	scaffold00670	Dinucleotide	(AT)8	16	160290	160305	Ca6	Dinucleotide	(AT)9	18	40090063	40090080	Intergenic		
Ca_GpSSR01206	scaffold02846	Dinucleotide	(AT)6	12	2558	2569	Ca6	Dinucleotide	(AT)7	14	41203445	41203458	Intergenic	TTCCTTCTCTATCTCCCTCTT	AAGTAGTCGTAAGAAAAAGGTG
Ca_GpSSR01207	scaffold71214	Dinucleotide	(TA)17	34	1	34	Ca6	Dinucleotide	(TA)22	44	42015544	42015587	Intergenic	CTAGAGACGTTTAGGTGGACA	TATGGCAATACCACCATCTAA
Ca_GpSSR01208	scaffold03961	Trinucleotide	(ATA)23	69	12854	12922	Ca6	Trinucleotide	(ATA)20	60	42226507	42226566	Intergenic		
Ca_GpSSR01209	scaffold04235	Trinucleotide	(TGT)8	24	66548	66571	Ca6	Trinucleotide	(TGT)9	27	42737671	42737697	upstream:Ca_09486;	GTTTCATCGGAGATGTCAAT	CAATTTCCAAAACTTCACACT
Ca_GpSSR01210	scaffold02548	Dinucleotide	(TA)29	58	19193	19250	Ca6	Dinucleotide	(TA)10	20	42931488	42931507	Intergenic	TTTGAGATTTTTGGCTCACTA	TTTGTTGTGATTGTGTTGTTC
Ca_GpSSR01211	scaffold01119	Trinucleotide	(TTA)7	21	35	55	Ca6	Trinucleotide	(TTA)6	18	44169073	44169090	Intergenic		
Ca_GpSSR01212	scaffold01119	Dinucleotide	(TA)10	20	20164	20183	Ca6	Dinucleotide	(TA)11	22	44189388	44189409	Intergenic		

Ca_GpSSR01213	scaffold01257	Dinucleotide	(TA)9	18	2466	2483	Ca6	Dinucleotide	(TA)15	30	44479850	44479879	upstream:Ca_15367;	GAAGGAGGTGAGTTTTGAAAT	TGATTTTACTTGACTGACTTTGA
Ca_GpSSR01214	scaffold112824	Dinucleotide	(TA)13	26	224	249	Ca6	Dinucleotide	(TA)15	30	44822262	44822291	Intergenic	TTCAAAAGTTTTCACTCTTCCT	ACTAATTTATTGCGATACGA
Ca_GpSSR01215	scaffold22507	Dinucleotide	(AT)24	48	1401	1448	Ca6	Dinucleotide	(AT)27	54	44832957	44833010	Intergenic	AGCACTTGTTAACGTTTTCTT	GTCAATCTATTGTGATGTTTTTG
Ca_GpSSR01216	scaffold113178	Dinucleotide	(AT)22	44	6	49	Ca6	Dinucleotide	(AT)15	30	45279356	45279385	Intergenic		
Ca_GpSSR01217	Ca_LG_5	Dinucleotide	(TA)19	38	12364	12401	Ca6	Dinucleotide	(TA)16	32	45484769	45484800	Intergenic		
Ca_GpSSR01218	Ca_LG_5	Dinucleotide	(TA)19	38	12473	12510	Ca6	Dinucleotide	(TA)11	22	45485155	45485176	Intergenic		
Ca_GpSSR01219	Ca_LG_5	Dinucleotide	(AT)15	30	164047	164076	Ca6	Dinucleotide	(AT)27	54	45622795	45622848	Intergenic	AGCACTTGTTAACGTTTTCTT	GTCAATCTATTGTGATGTTTTTG
Ca_GpSSR01220	Ca_LG_5	Dinucleotide	(AT)7	14	187810	187823	Ca6	Dinucleotide	(AT)6	12	45649189	45649200	Intergenic	CTTCTTTACATCTGCTCTCCA	GCGCAAAATTCCTTTTAGTAT
Ca_GpSSR01221	scaffold05824	Dinucleotide	(TA)8	16	328240	328255	Ca6	Dinucleotide	(TA)9	18	45895265	45895282	Intergenic	TCCTTTCTTTGCCTTTTATG	AAAAGTGGTTTGTGATGTGAC
Ca_GpSSR01222	scaffold07820	Dinucleotide	(TA)13	26	7104	7129	Ca6	Dinucleotide	(TA)14	28	45900189	45900216	Intergenic		
Ca_GpSSR01223	scaffold05824	Dinucleotide	(GT)7	14	447689	447702	Ca6	Dinucleotide	(GT)8	16	46026054	46026069	upstream:Ca_08944;		
Ca_GpSSR01224	scaffold00612	Dinucleotide	(AT)12	24	214143	214166	Ca6	Dinucleotide	(AT)17	34	46533446	46533479	Intergenic	CGAACCTTCATCTATTACACG	ACTATGTCTTCTTGGTTGTATTT
Ca_GpSSR01225	scaffold00612	Dinucleotide	(AC)8	16	33404	33419	Ca6	Dinucleotide	(AC)9	18	46657224	46657241	Intergenic	TTTTGGTCTTTGATACACGAT	TCTTGAAAAATTTGTGTCTCC
Ca_GpSSR01226	scaffold00781	Dinucleotide	(AT)12	24	4770	4793	Ca6	Dinucleotide	(AT)11	22	46852237	46852258	Intergenic		
Ca_GpSSR01227	scaffold00781	Dinucleotide	(AT)13	26	47903	47928	Ca6	Dinucleotide	(AT)9	18	46895130	46895147	Intergenic		
Ca_GpSSR01228	scaffold00781	Dinucleotide	(AT)7	14	82277	82290	Ca6	Dinucleotide	(AT)12	24	46930086	46930109	Intergenic		
Ca_GpSSR01229	scaffold00664	Dinucleotide	(TA)9	18	41595	41612	Ca6	Dinucleotide	(TA)16	32	47274788	47274819	Intergenic		
Ca_GpSSR01230	scaffold03419	Trinucleotide	(AAT)27	81	27662	27742	Ca6	Trinucleotide	(AAT)33	99	47490301	47490399	Intergenic	TTTGGAAGTTTGGAAGTTGCT	GGGTGAGGTGGTCCAATAAT
Ca_GpSSR01231	scaffold02694	Dinucleotide	(AT)12	24	19850	19873	Ca6	Dinucleotide	(AT)11	22	47594118	47594139	Intergenic		
Ca_GpSSR01232	scaffold02694	Dinucleotide	(TA)14	28	29806	29833	Ca6	Dinucleotide	(TA)6	12	47604155	47604166	Intergenic	TGTCAACAAAAGaCTAGGCTAGGA	AATTTTGTAACGACCAAcAACA
Ca_GpSSR01233	scaffold03024	Pentanucleotide	(CTCTT)9	45	4747	4791	Ca6	Pentanucleotide	(CTCTT)12	60	47687503	47687562	Intergenic	ATTTCTCCCCTTTTATTTCCT	GTTTGATATGGTTTGTTAGCC
Ca_GpSSR01234	scaffold01578	Dinucleotide	(TA)11	22	9329	9350	Ca6	Dinucleotide	(TA)12	24	47840259	47840282	upstream:Ca_16082;		
Ca_GpSSR01235	scaffold13920	Dinucleotide	(TA)10	20	1067	1086	Ca6	Dinucleotide	(TA)9	18	47962228	47962245	Intergenic	TTTCATTAGGGTGATAACCAA	GGGAACATAATCTTAACACAAAA
Ca_GpSSR01236	scaffold02134	Dinucleotide	(TA)18	36	41609	41644	Ca6	Dinucleotide	(TA)11	22	48659871	48659892	Intergenic	CTTGCTTTCATTTTTCCTACA	AGTTTACACAACCATAAACAAGA
Ca_GpSSR01237	scaffold04173	Dinucleotide	(AT)9	18	14232	14249	Ca6	Dinucleotide	(AT)11	22	48730440	48730461	Intergenic	CCGAAAAGATTGAAATGAAG	GTTTTAAGAGGGGATTTTTCA
Ca_GpSSR01238	scaffold05830	Dinucleotide	(AT)15	30	3302	3331	Ca6	Dinucleotide	(AT)25	50	49174584	49174633	Intergenic	TGAAAACATCTAAATTTCTACCG	CAGCTAACAATTAAACCTCCA
Ca_GpSSR01239	Ca_LG_2	Trinucleotide	(TAT)26	78	13223880	13223957	Ca6	Trinucleotide	(TAT)17	51	49337554	49337604	Intergenic	GACGATTCTCCATAACTGTGA	ACAAAGGTTGTGTTTCCTAAA
Ca_GpSSR01240	Ca_LG_2	Dinucleotide	(AT)17	34	13345118	13345151	Ca6	Dinucleotide	(AT)27	54	49485417	49485470	Intergenic	AGCACTTGTTAACGTTTTCTT	GTCAATCTATTGTGATGTTTTTG
Ca_GpSSR01241	Ca_LG_2	Trinucleotide	(TAA)8	24	13363335	13363358	Ca6	Trinucleotide	(TAA)9	27	49507544	49507570	Intergenic		
Ca_GpSSR01242	Ca_LG_2	Dinucleotide	(TA)10	20	13448318	13448337	Ca6	Dinucleotide	(TA)11	22	49593059	49593080	upstream:Ca_01877;	ATTTGCACTCTTTGACTTTGA	TCAAATGTTTAACTCGTGGAC
Ca_GpSSR01243	scaffold01526	Dinucleotide	(AT)9	18	5045	5062	Ca6	Dinucleotide	(AT)10	20	49615010	49615029	Intergenic	TGAGGAACATATTTTGGACAC	AACCGTATAGATGCTACACCA
Ca_GpSSR01244	scaffold02923	Trinucleotide	(ATA)5	15	31292	31306	Ca6	Trinucleotide	(ATA)6	18	49702455	49702472	Intergenic		
Ca_GpSSR01245	scaffold00944	Dinucleotide	(AT)10	20	58253	58272	Ca6	Dinucleotide	(AT)14	28	49742480	49742507	Intergenic	GCAAAATAGGATAGCGTTGTT	AGATGCGACCATATACACAGT
Ca_GpSSR01246	scaffold00156	Dinucleotide	(AT)9	18	242203	242220	Ca6	Dinucleotide	(AT)8	16	49884640	49884655	gene=Ca_11030;mRNA=Ca_11030.1;intron;		
Ca_GpSSR01247	scaffold57484	Dinucleotide	(AT)22	44	583	626	Ca6	Dinucleotide	(AT)10	20	50254786	50254805	Intergenic		
Ca_GpSSR01248	Ca_LG_2	Dinucleotide	(TA)9	18	12083046	12083063	Ca6	Dinucleotide	(TA)8	16	50488404	50488419	Intergenic	ATTTGCACTCTTTGACTTTGA	TCAAATGTTTAACTCGTGGAC
Ca_GpSSR01249	scaffold18225	Dinucleotide	(AT)18	36	1822	1857	Ca6	Dinucleotide	(AT)25	50	50516807	50516856	Intergenic	TGAAAACATCTAAATTTCTACCG	CAGCTAACAATTAAACCTCCA
Ca_GpSSR01250	scaffold01684	Dinucleotide	(AT)9	18	31939	31956	Ca6	Dinucleotide	(AT)8	16	50591641	50591656	Intergenic	TCACACTGCACACAAATTAAA	TCATCATTACTAACGGTTTGG
Ca_GpSSR01251	scaffold08735	Dinucleotide	(TA)12	24	1821	1844	Ca6	Dinucleotide	(TA)21	42	50906889	50906930	Intergenic	CATAAAGAAATTACCTACTGTGA	GAAGAGAAACAAGAGGAAGAAA
Ca_GpSSR01252	scaffold00357	Trinucleotide	(TTA)29	87	67301	67387	Ca6	Trinucleotide	(TTA)30	90	51282231	51282320	Intergenic		
Ca_GpSSR01253	scaffold00357	Trinucleotide	(ATT)8	24	132346	132369	Ca6	Trinucleotide	(ATT)7	21	51350137	51350157	Intergenic		
Ca_GpSSR01254	scaffold26868	Dinucleotide	(TA)6	12	1209	1220	Ca6	Dinucleotide	(TA)11	22	51458907	51458928	Intergenic		
Ca_GpSSR01255	scaffold01328	Dinucleotide	(TA)9	18	621	638	Ca6	Dinucleotide	(TA)8	16	51863374	51863389	Intergenic	TGTTCCTCAAACTATCCAATG	GAGGCATTTAGCCAATTTTAT
Ca_GpSSR01256	scaffold04010	Dinucleotide	(TA)23	46	11543	11588	Ca6	Dinucleotide	(TA)31	62	51946540	51946601	gene=Ca_18727;mRNA=Ca_18727.1;intron;	TGCAAAGACTAAAATCACACA	CCGCATATACTTTAGATGGAA
Ca_GpSSR01257	scaffold07676	Dinucleotide	(TA)21	42	7291	7332	Ca6	Dinucleotide	(TA)11	22	52306606	52306627	upstream:Ca_19937;		
Ca_GpSSR01258	scaffold02105	Dinucleotide	(AT)9	18	47060	47077	Ca6	Dinucleotide	(AT)12	24	52670437	52670460	Intergenic	CATCCCTAAAACGATTGTTAAT	GTATAAGTACCCGCGAGTTTA
Ca_GpSSR01259	scaffold10252	Dinucleotide	(TA)11	22	1912	1933	Ca6	Dinucleotide	(TA)17	34	52894507	52894540	Intergenic	CCGCACCTGCAAGTAATA	AGAATTTAGACCCTCATCTCC
Ca_GpSSR01260	Ca_LG_2	Trinucleotide	(TTA)20	60	12463666	12463725	Ca6	Trinucleotide	(TTA)17	51	53069214	53069264	Intergenic		
Ca_GpSSR01261	scaffold07706	Dinucleotide	(AT)11	22	3066	3087	Ca6	Dinucleotide	(AT)10	20	53076749	53076768	Intergenic	GTCCCAATAATTCATTACTAAAC	TGCTGACATCTTAAAGGTGAA
Ca_GpSSR01262	scaffold00398	Dinucleotide	(AT)20	40	124752	124791	Ca6	Dinucleotide	(AT)24	48	53398579	53398626	Intergenic	CTAGAGACGTTTAGGTGGACA	GGCAATACCACCATCTAAGTA
Ca_GpSSR01263	scaffold132483	Dinucleotide	(TA)18	36	246	281	Ca6	Dinucleotide	(TA)12	24	53407010	53407033	Intergenic		
Ca_GpSSR01264	Ca_LG_2	Dinucleotide	(TA)12	24	8260815	8260838	Ca6	Dinucleotide	(TA)14	28	53650550	53650577	Intergenic		
Ca_GpSSR01265	scaffold43450	Dinucleotide	(AT)6	12	1	12	Ca6	Dinucleotide	(AT)10	20	53942134	53942153	Intergenic		
Ca_GpSSR01266	scaffold01122	Dinucleotide	(AT)9	18	50770	50787	Ca6	Dinucleotide	(AT)10	20	54123012	54123031	Intergenic	CAACGAGATAGTACGAAAGGA	TGCACATGTTCCTCATATACA
Ca_GpSSR01267	Ca_LG_2	Dinucleotide	(TA)11	22	9829709	9829730	Ca6	Dinucleotide	(TA)10	20	54193025	54193044	Intergenic	ATTTGCACTCTTTGACTTTGA	TCAAATGTTTAACTCGTGGAC
Ca_GpSSR01268	Ca_LG_2	Dinucleotide	(AT)8	16	9839053	9839068	Ca6	Dinucleotide	(AT)9	18	54202310	54202327	downstream:Ca_01702;	ATTTGCACTCTTTGACTTTGA	TCAAATGTTTAACTCGTGGAC
Ca_GpSSR01269	scaffold00806	Dinucleotide	(AT)12	24	22953	22976	Ca6	Dinucleotide	(AT)13	26	54342059	54342084	Intergenic		
Ca_GpSSR01270	scaffold01333	Trinucleotide	(TAA)5	15	82320	82334	Ca6	Trinucleotide	(TAA)6	18	54912176	54912193	Intergenic	TTTTTGGCGTAGTGGATATAG	AAGACCGAGTTTGATTACTCC
Ca_GpSSR01271	scaffold01333	Dinucleotide	(AT)10	20	82959	82978	Ca6	Dinucleotide	(AT)9	18	54912818	54912835	Intergenic		
Ca_GpSSR01272	scaffold01333	Trinucleotide	(TAA)11	33	95083	95115	Ca6	Trinucleotide	(TAA)12	36	54927391	54927426	Intergenic		
Ca_GpSSR01273	scaffold01333	Dinucleotide	(TA)12	24	150230	150253	Ca6	Dinucleotide	(TA)6	12	54983871	54983882	Intergenic	TGACGTGTCATGCATCAAATAA	TGAATCGTTCATCAAACACGA
Ca_GpSSR01274	scaffold00907	Dinucleotide	(TA)8	16	141878	141893	Ca6	Dinucleotide	(TA)10	20	55109999	55110018	Intergenic		
Ca_GpSSR01275	scaffold00907	Dinucleotide	(AT)11	22	3734	3755	Ca6	Dinucleotide	(AT)12	24	55168294	55168317	Intergenic		
Ca_GpSSR01276	scaffold00907	Dinucleotide	(TA)24	48	7374	7421	Ca6	Dinucleotide	(TA)8	16	55170938	55170953	Intergenic		
Ca_GpSSR01277	scaffold89817	Trinucleotide	(ATA)5	15	1	15	Ca6	Trinucleotide	(ATA)6	18	55406873	55406890	Intergenic		
Ca_GpSSR01278	Ca_LG_2	Dinucleotide	(TA)15	30	777203	777232	Ca6	Dinucleotide	(TA)25	50	56537564	56537613	gene=Ca_01075;mRNA=Ca_01075.1;intron;	TCAATTCGAACAAAGTAACGGA	TTGGACCAAATTAGGAATATCCA
Ca_GpSSR01279	Ca_LG_2	Trinucleotide	(AAT)10	30	1103481	1103510	Ca6	Trinucleotide	(AAT)12	36	56874983	56875018	Intergenic	GAAATCGAAGATGATTGACAC	AAGAAAAGGGAAAAGTTGGTT
Ca_GpSSR01280	Ca_LG_2	Dinucleotide	(AT)22	44	854374	854417	Ca6	Dinucleotide	(AT)19	38	56991351	56991388	Intergenic		
Ca_GpSSR01281	scaffold02407	Dinucleotide	(TA)17	34	42363	42396	Ca6	Dinucleotide	(TA)15	30	57061119	57061148	Intergenic		
Ca_GpSSR01282	Ca_LG_2	Dinucleotide	(TA)10	20	5974179	5974198	Ca6	Dinucleotide	(TA)12	24	57588555	57588578	Intergenic	ATTTGCACTCTTTGACTTTGA	TCAAATGTTTAACTCGTGGAC
Ca_GpSSR01283	Ca_LG_2	Trinucleotide	(ATT)34	102	5996147	5996248	Ca6	Trinucleotide	(ATT)13	39	57610529	57610567	upstream:Ca_01452;	AAGTTGTGCAACAAATAGGAA	ATACCGAACAACAACAACAAC
Ca_GpSSR01284	Ca_LG_2	Trinucleotide	(ATT)20	60	5996271	5996330	Ca6	Trinucleotide	(ATT)19	57	57610646	57610702	upstream:Ca_01452;		
Ca_GpSSR01285	scaffold40501	Dinucleotide	(TA)12	24	813	836	Ca6	Dinucleotide	(TA)11	22	57717132	57717153	Intergenic		
Ca_GpSSR01286	Ca_LG_2	Trinucleotide	(CTT)8	24	5693940	5693963	Ca6	Trinucleotide	(CTT)7	21	57830865	57830885	mRNA=Ca_01432.1;5'-UTR=Ca_01432.1.5'-UTR1;exor	GCACAAATACTGAAACCCTTA	GAGGAAGTTGAAATCGAAAAT
Ca_GpSSR01287	Ca_LG_2	Dinucleotide	(TA)7	14	5557641	5557654	Ca6	Dinucleotide	(TA)13	26	57952561	57952586	upstream:Ca_01420;	ATTTGCACTCTTTGACTTTGA	TCAAATGTTTAACTCGTGGAC
Ca_GpSSR01288	Ca_LG_2	Trinucleotide	(TTG)6	18	5583513	5583530	Ca6	Trinucleotide	(TTG)5	15	57975402	57975416	mRNA=Ca_01422.1;gene=Ca_01422;intron;	TTTGAGATGAGACCACCTATG	AACATCACAACCGTAAACAAC
Ca_GpSSR01289	Ca_LG_2	Dinucleotide	(GA)8	16	5603850	5603865	Ca6	Dinucleotide	(GA)7	14	57995975	57995988	gene=Ca_01425;mRNA=Ca_01425.1;intron;	AGCGAAATGTATTTCAGTGAG	CTTTCATTCTTCTCTCCCATT
Ca_GpSSR01290	Ca_LG_2	Dinucleotide	(TA)10	20	16793833	16793852	Ca6	Dinucleotide	(TA)11	22	58523124	58523145	Intergenic	ATTTGCACTCTTTGACTTTGA	TCAAATGTTTAACTCGTGGAC
Ca_GpSSR01291	scaffold22515	Dinucleotide	(GA)19	38	1426	1463	Ca6	Dinucleotide	(GA)47	94	58686896	58686989	upstream:Ca_20822;	AATGTCTGAGTGTTTCCTTCA	CAAACATTAAAGCCAAAAACC
Ca_GpSSR01292	Ca_LG_7	Dinucleotide	(AC)7	14	1912759	1912772	Ca6	Dinucleotide	(AC)6	12	58783050	58783061	Intergenic	AGTTTTAGGGCGATTCTTATG	CCATTCACAACCAATTTCTAA
Ca_GpSSR01293	Ca_LG_7	Dinucleotide	(AT)12	24	2026131	2026154	Ca6	Dinucleotide	(AT)10	20	58886584	58886603	Intergenic		

Ca_GpSSR01294	Ca_LG_7	Dinucleotide	(AT)13	26	2026159	2026184	Ca6	Dinucleotide	(AT)15	30	58886632	58886661	Intergenic		
Ca_GpSSR01295	scaffold00851	Dinucleotide	(AT)10	20	45610	45629	Ca6	Dinucleotide	(AT)12	24	58988840	58988863	Intergenic		
Ca_GpSSR01296	scaffold07835	Dinucleotide	(AT)10	20	4750	4769	Ca6	Dinucleotide	(AT)23	46	59017134	59017179	Intergenic	CTAGAGACGTTTAGGTGGACA	GGCAATACCACCATCTAAGTA
Ca_GpSSR01297	scaffold07835	Dinucleotide	(TA)23	46	6	51	Ca6	Dinucleotide	(TA)14	28	59021882	59021909	Intergenic		
Ca_GpSSR01298	Ca_LG_2	Dinucleotide	(AT)9	18	204946	204963	Ca6	Dinucleotide	(AT)7	14	59222237	59222250	upstream:Ca_01042;	ATTTGCACTCTTTGACTTTGA	TCAAATGTTTAACTCGTGGAC
Ca_GpSSR01299	Ca_LG_2	Trinucleotide	(ATT)31	93	5114761	5114853	Ca6	Trinucleotide	(ATT)30	90	59294342	59294431	Intergenic	AAGTTGTGCAACAAATAGGAA	ATACCGAACAACAACAACAAC
Ca_GpSSR01300	Ca_LG_2	Dinucleotide	(TA)7	14	5146613	5146626	Ca6	Dinucleotide	(TA)8	16	59332874	59332889	Intergenic	ATTTGCACTCTTTGACTTTGA	TCAAATGTTTAACTCGTGGAC
Ca_GpSSR01301	Ca_LG_2	Dinucleotide	(TA)20	40	5228049	5228088	Ca6	Dinucleotide	(TA)11	22	59422589	59422610	Intergenic		
Ca_GpSSR01302	scaffold02677	Dinucleotide	(TA)6	12	9615	9626	Ca7	Dinucleotide	(TA)11	22	395210	395231	Intergenic	CCAAAGTCCTTCAGGTAAAAT	AAACTTAAAAACACACGCAAC
Ca_GpSSR01303	scaffold04112	Trinucleotide	(TAA)39	117	5653	5769	Ca7	Trinucleotide	(TAA)26	78	2512042	2512119	Intergenic		
Ca_GpSSR01304	scaffold07183	Dinucleotide	(TA)7	14	5222	5235	Ca7	Dinucleotide	(TA)8	16	3339954	3339969	Intergenic	GTTTCCGAGTTCATTTTCTTT	GCTAGCGTGTTATATTTCCAA
Ca_GpSSR01305	Ca_LG_8	Trinucleotide	(AAT)45	135	3688927	3689061	Ca7	Trinucleotide	(AAT)32	96	3551397	3551492	Intergenic		
Ca_GpSSR01306	Ca_LG_8	Dinucleotide	(AT)10	20	4263892	4263911	Ca7	Dinucleotide	(AT)9	18	4161324	4161341	Intergenic	CCACTAGTATTATGGACAAATCA	GTGGTTTGCAAGGTTTTATATT
Ca_GpSSR01307	Ca_LG_8	Dinucleotide	(GA)8	16	4611654	4611669	Ca7	Dinucleotide	(GA)9	18	4508978	4508995	mRNA=Ca_08273.1;gene=Ca_08273;intron;		
Ca_GpSSR01308	Ca_LG_8	Dinucleotide	(AT)10	20	4818999	4819018	Ca7	Dinucleotide	(AT)9	18	4722840	4722857	gene=Ca_08294;mRNA=Ca_08294.1;intron;	CCACTAGTATTATGGACAAATCA	GTGGTTTGCAAGGTTTTATATT
Ca_GpSSR01309	Ca_LG_8	Dinucleotide	(TA)19	38	4931040	4931077	Ca7	Dinucleotide	(TA)22	44	4834678	4834721	upstream:Ca_08311;	CCTCCCTCCAATTTATACCTA	CACTTTTGCCCAACTATTTTA
Ca_GpSSR01310	scaffold09237	Trinucleotide	(AAT)8	24	3927	3950	Ca7	Trinucleotide	(AAT)17	51	5100972	5101022	Intergenic		
Ca_GpSSR01311	scaffold01398	Trinucleotide	(TTA)9	27	32384	32410	Ca7	Trinucleotide	(TTA)30	90	5102749	5102838	Intergenic		
Ca_GpSSR01312	Ca_LG_8	Dinucleotide	(TA)11	22	6040663	6040684	Ca7	Dinucleotide	(TA)30	60	5927407	5927466	upstream:Ca_08414;	CCACTAGTATTATGGACAAATCA	GTGGTTTGCAAGGTTTTATATT
Ca_GpSSR01313	Ca_LG_8	Dinucleotide	(TA)23	46	5909306	5909351	Ca7	Dinucleotide	(TA)8	16	6059605	6059620	gene=Ca_08404;mRNA=Ca_08404.1;intron;	ACACACAATGAAGATTGAACA	TTCATCTTTGTTTCTTTCACG
Ca_GpSSR01314	Ca_LG_8	Dinucleotide	(TA)6	12	5909025	5909036	Ca7	Dinucleotide	(TA)12	24	6059790	6059813	gene=Ca_08404;mRNA=Ca_08404.1;intron;	ACACACAATGAAGATTGAACA	TTCATCTTTGTTTCTTTCACG
Ca_GpSSR01315	Ca_LG_8	Dinucleotide	(AT)10	20	5526700	5526719	Ca7	Dinucleotide	(AT)11	22	6444785	6444806	Intergenic	CCACTAGTATTATGGACAAATCA	GTGGTTTGCAAGGTTTTATATT
Ca_GpSSR01316	Ca_LG_8	Dinucleotide	(AT)10	20	5337996	5338015	Ca7	Dinucleotide	(AT)9	18	6635524	6635541	Intergenic	CCACTAGTATTATGGACAAATCA	GTGGTTTGCAAGGTTTTATATT
Ca_GpSSR01317	Ca_LG_8	Dinucleotide	(AT)11	22	5301069	5301090	Ca7	Dinucleotide	(AT)10	20	6674040	6674059	Intergenic	CCACTAGTATTATGGACAAATCA	GTGGTTTGCAAGGTTTTATATT
Ca_GpSSR01318	scaffold00630	Dinucleotide	(AT)17	34	98205	98238	Ca7	Dinucleotide	(AT)19	38	6918589	6918626	Intergenic	ACACACAATGAAGATTGAACA	TTCATCTTTGTTTCTTTCACG
Ca_GpSSR01319	scaffold21377	Dinucleotide	(AG)10	20	1	20	Ca7	Dinucleotide	(AG)12	24	7213674	7213697	Intergenic	AAGTTGGGTGATTAATTTGGT	TAACAGAGGGAAGTGAAGTGA
Ca_GpSSR01320	scaffold01708	Dinucleotide	(AT)21	42	38349	38390	Ca7	Dinucleotide	(AT)23	46	7793060	7793105	upstream:Ca_16316;	CCTCCCTCCAATTTATACCTA	CACTTTTGCCCAACTATTTTA
Ca_GpSSR01321	scaffold01708	Dinucleotide	(AT)22	44	38769	38812	Ca7	Dinucleotide	(AT)23	46	7794482	7794527	upstream:Ca_16316;	CCTCCCTCCAATTTATACCTA	CACTTTTGCCCAACTATTTTA
Ca_GpSSR01322	Ca_LG_8	Dinucleotide	(AT)11	22	6817198	6817219	Ca7	Dinucleotide	(AT)10	20	7862329	7862348	Intergenic	CCACTAGTATTATGGACAAATCA	GTGGTTTGCAAGGTTTTATATT
Ca_GpSSR01323	scaffold01506	Trinucleotide	(ATT)18	54	19740	19793	Ca7	Trinucleotide	(ATT)17	51	8044763	8044813	upstream:Ca_15947;		
Ca_GpSSR01324	scaffold01506	Trinucleotide	(ATT)10	30	69432	69461	Ca7	Trinucleotide	(ATT)11	33	8097201	8097233	Intergenic		
Ca_GpSSR01325	Ca_LG_8	Dinucleotide	(AT)11	22	6529039	6529060	Ca7	Dinucleotide	(AT)10	20	8412209	8412228	Intergenic	CCACTAGTATTATGGACAAATCA	GTGGTTTGCAAGGTTTTATATT
Ca_GpSSR01326	Ca_LG_8	Dinucleotide	(AT)8	16	6550522	6550537	Ca7	Dinucleotide	(AT)9	18	8434059	8434076	upstream:Ca_08464;	ACACACAATGAAGATTGAACA	TTCATCTTTGTTTCTTTCACG
Ca_GpSSR01327	scaffold06808	Dinucleotide	(AT)13	26	2	27	Ca7	Dinucleotide	(AT)11	22	8868007	8868028	Intergenic	CGCATATACAATTCAGCCTAT	ACAAAAATTCAAGCGACACTA
Ca_GpSSR01328	Ca_LG_8	Dinucleotide	(AT)10	20	7741641	7741660	Ca7	Dinucleotide	(AT)9	18	9408183	9408200	Intergenic	CCACTAGTATTATGGACAAATCA	GTGGTTTGCAAGGTTTTATATT
Ca_GpSSR01329	Ca_LG_8	Dinucleotide	(TA)6	12	8105733	8105744	Ca7	Dinucleotide	(TA)8	16	9630834	9630849	downstream:Ca_08576;	ACACACAATGAAGATTGAACA	TTCATCTTTGTTTCTTTCACG
Ca_GpSSR01330	scaffold65919	Dinucleotide	(AT)27	54	505	558	Ca7	Dinucleotide	(AT)25	50	10038170	10038219	Intergenic	CCTCCCTCCAATTTATACCTA	CACTTTTGCCCAACTATTTTA
Ca_GpSSR01331	scaffold15076	Dinucleotide	(AT)19	38	10	47	Ca7	Dinucleotide	(AT)18	36	10136595	10136630	upstream:Ca_20601;	ACACACAATGAAGATTGAACA	TTCATCTTTGTTTCTTTCACG
Ca_GpSSR01332	scaffold20437	Dinucleotide	(AT)9	18	1616	1633	Ca7	Dinucleotide	(AT)8	16	10173319	10173334	Intergenic	ACACACAATGAAGATTGAACA	TTCATCTTTGTTTCTTTCACG
Ca_GpSSR01333	Ca_LG_7	Dinucleotide	(TA)8	16	7046667	7046682	Ca7	Dinucleotide	(TA)7	14	10297701	10297714	Intergenic	ACACACAATGAAGATTGAACA	TTCATCTTTGTTTCTTTCACG
Ca_GpSSR01334	Ca_LG_7	Dinucleotide	(AT)6	12	7150572	7150583	Ca7	Dinucleotide	(AT)24	48	10403718	10403765	Intergenic	CCTCCCTCCAATTTATACCTA	CACTTTTGCCCAACTATTTTA
Ca_GpSSR01335	scaffold00872	Dinucleotide	(AT)8	16	96154	96169	Ca7	Dinucleotide	(AT)9	18	10604036	10604053	upstream:Ca_14352;	ACACACAATGAAGATTGAACA	TTCATCTTTGTTTCTTTCACG
Ca_GpSSR01336	scaffold00872	Dinucleotide	(AT)10	20	66156	66175	Ca7	Dinucleotide	(AT)11	22	10634278	10634299	Intergenic	CCCTCCTGTTTTATAGAAAGTT	CCACCTGATTTTGAATATACG
Ca_GpSSR01337	scaffold01885	Dinucleotide	(TA)15	30	45406	45435	Ca7	Dinucleotide	(TA)16	32	10933681	10933712	Intergenic	TCATGTCGTCTATGAACAAAT	TTCTTTTATTTTGGTCGAGAGT
Ca_GpSSR01338	Ca_LG_7	Trinucleotide	(ATA)21	63	6924239	6924301	Ca7	Trinucleotide	(ATA)19	57	11166452	11166508	downstream:Ca_07847;		
Ca_GpSSR01339	scaffold71852	Trinucleotide	(TTA)32	96	401	496	Ca7	Trinucleotide	(TTA)13	39	11312079	11312117	Intergenic		
Ca_GpSSR01340	scaffold11781	Dinucleotide	(AT)6	12	4072	4083	Ca7	Dinucleotide	(AT)25	50	11316509	11316558	Intergenic	CGATTTCTCGAAGGTAAAAA	ATGCTAATGGATATGTTGGTG
Ca_GpSSR01341	Ca_LG_7	Dinucleotide	(AT)13	26	6711804	6711829	Ca7	Dinucleotide	(AT)14	28	11435091	11435118	Intergenic	ACACACAATGAAGATTGAACA	TTCATCTTTGTTTCTTTCACG
Ca_GpSSR01342	Ca_LG_7	Dinucleotide	(TA)7	14	6614295	6614308	Ca7	Dinucleotide	(TA)8	16	11536695	11536710	mRNA=Ca_07821.1;gene=Ca_07821;intron;	ACACACAATGAAGATTGAACA	TTCATCTTTGTTTCTTTCACG
Ca_GpSSR01343	Ca_LG_7	Dinucleotide	(TA)9	18	6589791	6589808	Ca7	Dinucleotide	(TA)7	14	11568341	11568354	Intergenic	ACACACAATGAAGATTGAACA	TTCATCTTTGTTTCTTTCACG
Ca_GpSSR01344	Ca_LG_6	Dinucleotide	(TA)22	44	7059393	7059436	Ca7	Dinucleotide	(TA)11	22	12090643	12090664	Intergenic	ACACACAATGAAGATTGAACA	TTCATCTTTGTTTCTTTCACG
Ca_GpSSR01345	scaffold00308	Trinucleotide	(TAT)18	54	17466	17519	Ca7	Trinucleotide	(TAT)19	57	12128763	12128819	Intergenic	GCAATTCAGTGTTCAGTAGGT	GAGTCTCTATGACGTTGCACT
Ca_GpSSR01346	scaffold00308	Dinucleotide	(AT)19	38	21874	21911	Ca7	Dinucleotide	(AT)23	46	12133180	12133225	Intergenic	CCTCCCTCCAATTTATACCTA	CACTTTTGCCCAACTATTTTA
Ca_GpSSR01347	scaffold00308	Dinucleotide	(TA)21	42	162665	162706	Ca7	Dinucleotide	(TA)17	34	12275029	12275062	Intergenic	ACACACAATGAAGATTGAACA	TTCATCTTTGTTTCTTTCACG
Ca_GpSSR01348	scaffold00308	Dinucleotide	(TA)20	40	163021	163060	Ca7	Dinucleotide	(TA)7	14	12275492	12275505	Intergenic	ACACACAATGAAGATTGAACA	TTCATCTTTGTTTCTTTCACG
Ca_GpSSR01349	scaffold00308	Dinucleotide	(AT)19	38	200893	200930	Ca7	Dinucleotide	(AT)22	44	12311731	12311774	Intergenic	CCTCCCTCCAATTTATACCTA	CACTTTTGCCCAACTATTTTA
Ca_GpSSR01350	scaffold02660	Trinucleotide	(AAT)18	54	30951	31004	Ca7	Trinucleotide	(AAT)16	48	12347572	12347619	Intergenic	TGTGCACAAAAaGGTAGTAACAAAA	TGGTGGTGTAGTTGCATGATT
Ca_GpSSR01351	scaffold02660	Trinucleotide	(TCT)9	27	37157	37183	Ca7	Trinucleotide	(TCT)11	33	12362228	12362260	upstream:Ca_17720;	AGTCCCGTGCAAGAATTAAGA	CCCAATCCCAGCAATAAAAA
Ca_GpSSR01352	scaffold00046	Trinucleotide	(TTA)9	27	166438	166464	Ca7	Trinucleotide	(TTA)12	36	12786615	12786650	Intergenic		
Ca_GpSSR01353	scaffold00046	Dinucleotide	(TA)12	24	179309	179332	Ca7	Dinucleotide	(TA)11	22	12798399	12798420	Intergenic	CACGTCCATTCTACCAGTAAT	TTTGAATATCTCCTTTCACACA
Ca_GpSSR01354	scaffold00046	Dinucleotide	(AT)8	16	189522	189537	Ca7	Dinucleotide	(AT)11	22	12808769	12808790	Intergenic	AGCATTCTTAGTCCCTATTCC	TCCCATCTTAGCTTCTTTGAT
Ca_GpSSR01355	scaffold00046	Dinucleotide	(AT)12	24	190506	190529	Ca7	Dinucleotide	(AT)14	28	12809837	12809864	Intergenic	CACGTCCATTCTACCAGTAAT	TTTGAATATCTCCTTTCACACA
Ca_GpSSR01356	scaffold00046	Dinucleotide	(AT)7	14	240689	240702	Ca7	Dinucleotide	(AT)6	12	12854466	12854477	upstream:Ca_08758;	AATAGTCTTTGTTCTCGTCTGTT	CGCACTAGCTCTTTTTAATTT
Ca_GpSSR01357	scaffold00046	Dinucleotide	(AT)10	20	326227	326246	Ca7	Dinucleotide	(AT)9	18	12940318	12940335	Intergenic	CACGTCCATTCTACCAGTAAT	TTTGAATATCTCCTTTCACACA
Ca_GpSSR01358	scaffold00046	Dinucleotide	(AT)10	20	494517	494536	Ca7	Dinucleotide	(AT)9	18	13254525	13254542	Intergenic	CACGTCCATTCTACCAGTAAT	TTTGAATATCTCCTTTCACACA
Ca_GpSSR01359	Ca_LG_7	Dinucleotide	(TA)15	30	6115799	6115828	Ca7	Dinucleotide	(TA)16	32	13800048	13800079	upstream:Ca_07774;	GCCTAAAAGATATACAATTCACT	TCAAAGTGTTCGATGATTGTC
Ca_GpSSR01360	scaffold00553	Dinucleotide	(AT)10	20	133725	133744	Ca7	Dinucleotide	(AT)9	18	13909362	13909379	Intergenic	ACGGAGACAACTTGTGTTTTA	GTAATACGTGCACCCACAA
Ca_GpSSR01361	scaffold00644	Trinucleotide	(TTA)19	57	3688	3744	Ca7	Trinucleotide	(TTA)22	66	14123268	14123333	Intergenic	TGCACTCATCACTACTTACCA	GTTATGGTGGAGACAGATTGA
Ca_GpSSR01362	scaffold00644	Dinucleotide	(TA)15	30	46781	46810	Ca7	Dinucleotide	(TA)14	28	14165327	14165354	Intergenic	ACACACAATGAAGATTGAACA	TTCATCTTTGTTTCTTTCACG
Ca_GpSSR01363	scaffold00644	Dinucleotide	(AT)12	24	88377	88400	Ca7	Dinucleotide	(AT)13	26	14206905	14206930	upstream:Ca_13559;	ACACACAATGAAGATTGAACA	TTCATCTTTGTTTCTTTCACG
Ca_GpSSR01364	scaffold50860	Dinucleotide	(AT)14	28	594	621	Ca7	Dinucleotide	(AT)13	26	14788272	14788297	Intergenic	CGATGAAATGTGTTGGTTATT	TGGTAAAAACAAGTACGTAGAAA
Ca_GpSSR01365	scaffold02443	Dinucleotide	(AT)13	26	28002	28027	Ca7	Dinucleotide	(AT)15	30	14857993	14858022	Intergenic	AAGAATTATCAAGAGTGTCACAA	CAAAATTTCACAAGCATTAGC
Ca_GpSSR01366	scaffold28133	Trinucleotide	(TTA)10	30	352	381	Ca7	Trinucleotide	(TTA)11	33	15631378	15631410	Intergenic		
Ca_GpSSR01367	Ca_LG_7	Dinucleotide	(TA)10	20	5896938	5896957	Ca7	Dinucleotide	(TA)8	16	15902474	15902489	Intergenic	ACACACAATGAAGATTGAACA	TTCATCTTTGTTTCTTTCACG
Ca_GpSSR01368	Ca_LG_7	Dinucleotide	(TA)18	36	7375596	7375631	Ca7	Dinucleotide	(TA)21	42	16031089	16031130	Intergenic	GTGGTCGAAGATGAAAATAAA	GAGTCATTGGATGATGAAGAA
Ca_GpSSR01369	scaffold36755	Dinucleotide	(AT)17	34	891	924	Ca7	Dinucleotide	(AT)11	22	16191172	16191193	Intergenic	ACACACAATGAAGATTGAACA	TTCATCTTTGTTTCTTTCACG
Ca_GpSSR01370	Ca_LG_7	Dinucleotide	(AT)11	22	5430148	5430169	Ca7	Dinucleotide	(AT)6	12	16193831	16193842	Intergenic	ACACACAATGAAGATTGAACA	TTCATCTTTGTTTCTTTCACG
Ca_GpSSR01371	scaffold06114	Dinucleotide	(AT)6	12	600	611	Ca7	Dinucleotide	(AT)29	58	16578888	16578945	Intergenic	GTTTTCTTGGTCTCGATTTTT	TGAGTATATGCAACACACTACAA
Ca_GpSSR01372	scaffold06114	Dinucleotide	(AT)12	24	5428	5451	Ca7	Dinucleotide	(AT)11	22	16583326	16583347	mRNA=Ca_19579.1;gene=Ca_19579;intron;	TAGGCTAATAAGGCGAAACA	AAAGAATAGTCGTTTGGGTTC
Ca_GpSSR01373	scaffold71232	Dinucleotide	(TA)8	16	505	520	Ca7	Dinucleotide	(TA)12	24	16878381	16878404	Intergenic	ACACACAATGAAGATTGAACA	TTCATCTTTGTTTCTTTCACG
Ca_GpSSR01374	scaffold04454	Dinucleotide	(AT)8	16	14816	14831	Ca7	Dinucleotide	(AT)7	14	17171776	17171789	Intergenic	ACACACAATGAAGATTGAACA	TTCATCTTTGTTTCTTTCACG

Ca_GpSSR01375	scaffold25910	Dinucleotide	(TA)18	36	52	87	Ca7	Dinucleotide	(TA)16	32	17550569	17550600	Intergenic	ACACACAATGAAGATTGAACA	TTCATCTTTGTTTCTTTCACG
Ca_GpSSR01376	scaffold09259	Dinucleotide	(TA)18	36	5623	5658	Ca7	Dinucleotide	(TA)16	32	18441081	18441112	Intergenic	ACACACAATGAAGATTGAACA	TTCATCTTTGTTTCTTTCACG
Ca_GpSSR01377	scaffold04717	Dinucleotide	(AT)19	38	8989	9026	Ca7	Dinucleotide	(AT)21	42	18898574	18898615	Intergenic	ACACACAATGAAGATTGAACA	TTCATCTTTGTTTCTTTCACG
Ca_GpSSR01378	Ca_LG_7	Dinucleotide	(AT)13	26	5655354	5655379	Ca7	Dinucleotide	(AT)18	36	19179527	19179562	gene=Ca_07738;mRNA=Ca_07738.1;intron;	TGAGTGTGTACTGGGGTGAAA	AACACGCCCCTTCTTATCCT
Ca_GpSSR01379	Ca_LG_7	Dinucleotide	(AT)12	24	5474320	5474343	Ca7	Dinucleotide	(AT)31	62	19371993	19372054	Intergenic	GTTTTCTTGGTCTCGATTTTT	TGAGTATATGCAACACACTACAA
Ca_GpSSR01380	scaffold00797	Dinucleotide	(AT)13	26	36918	36943	Ca7	Dinucleotide	(AT)10	20	19534300	19534319	gene=Ca_14088;mRNA=Ca_14088.1;intron;	ACACACAATGAAGATTGAACA	TTCATCTTTGTTTCTTTCACG
Ca_GpSSR01381	scaffold00797	Dinucleotide	(TA)6	12	36836	36847	Ca7	Dinucleotide	(TA)15	30	19534403	19534432	gene=Ca_14088;mRNA=Ca_14088.1;intron;	TCGATATCCAGATGACATAAAA	CCCAAATATGACCTTACAAGA
Ca_GpSSR01382	scaffold00797	Dinucleotide	(AT)9	18	5112	5129	Ca7	Dinucleotide	(AT)10	20	19566080	19566099	Intergenic	ACACACAATGAAGATTGAACA	TTCATCTTTGTTTCTTTCACG
Ca_GpSSR01383	scaffold01116	Dinucleotide	(AT)9	18	131117	131134	Ca7	Dinucleotide	(AT)8	16	19656205	19656220	Intergenic	ACGTCTATCCTAAAATCAACTT	ACAGTGAAAGTGTGAAATGGA
Ca_GpSSR01384	scaffold01116	Dinucleotide	(AT)12	24	96584	96607	Ca7	Dinucleotide	(AT)11	22	19690949	19690970	Intergenic	ACGTCTATCCTAAAATCAACTT	ACAGTGAAAGTGTGAAATGGA
Ca_GpSSR01385	scaffold08707	Dinucleotide	(AT)8	16	2537	2552	Ca7	Dinucleotide	(AT)7	14	19747090	19747103	gene=Ca_20088;mRNA=Ca_20088.1;intron;	TTCTTGGTCTTCGTTATTCAA	CCAACCACATTACCTGAAATA
Ca_GpSSR01386	scaffold16000	Dinucleotide	(AT)14	28	758	785	Ca7	Dinucleotide	(AT)17	34	19871505	19871538	Intergenic	CTGTCACATCAATATTTAACATC	TTTTGAGTTAGTTGTTCTCCAA
Ca_GpSSR01387	scaffold01200	Dinucleotide	(AG)11	22	37142	37163	Ca7	Dinucleotide	(AG)12	24	20814618	20814641	mRNA=Ca_15198.1;gene=Ca_15198;intron;	GGAAAGAAGCATTTATCAAGA	GCGACGGTTAGAGGTTTATTA
Ca_GpSSR01388	Ca_LG_7	Dinucleotide	(TA)6	12	5038054	5038065	Ca7	Dinucleotide	(TA)28	56	20922821	20922876	Intergenic	GAAATTGAACCCACAATTCCA	AATTGGCAACGGTGAGAAAA
Ca_GpSSR01389	scaffold13433	Dinucleotide	(TA)14	28	6	33	Ca7	Dinucleotide	(TA)15	30	21140752	21140781	Intergenic	ACACACAATGAAGATTGAACA	TTCATCTTTGTTTCTTTCACG
Ca_GpSSR01390	scaffold00681	Trinucleotide	(ATT)17	51	37979	38029	Ca7	Trinucleotide	(ATT)13	39	21308542	21308580	Intergenic		
Ca_GpSSR01391	scaffold00681	Trinucleotide	(ATT)28	84	38033	38116	Ca7	Trinucleotide	(ATT)21	63	21308584	21308646	Intergenic		
Ca_GpSSR01392	scaffold00681	Dinucleotide	(TA)10	20	88019	88038	Ca7	Dinucleotide	(TA)13	26	21359434	21359459	Intergenic	ACACACAATGAAGATTGAACA	TTCATCTTTGTTTCTTTCACG
Ca_GpSSR01393	scaffold00374	Dinucleotide	(TA)11	22	120311	120332	Ca7	Dinucleotide	(TA)33	66	21454803	21454868	mRNA=Ca_12349.1;gene=Ca_12349;intron;	GTTTTCTTGGTCTCGATTTTT	TGAGTATATGCAACACACTACAA
Ca_GpSSR01394	scaffold02103	Trinucleotide	(TTA)39	117	20982	21098	Ca7	Trinucleotide	(TTA)29	87	21760762	21760848	upstream:Ca_16929;		
Ca_GpSSR01395	scaffold02103	Dinucleotide	(AT)10	20	45110	45129	Ca7	Dinucleotide	(AT)11	22	21772694	21772715	Intergenic	TAATACCTTCGCCTTACGTTT	GGTGTTAACGTAACAAATTGAA
Ca_GpSSR01396	scaffold04310	Trinucleotide	(AAT)7	21	20247	20267	Ca7	Trinucleotide	(AAT)5	15	21905049	21905063	Intergenic		
Ca_GpSSR01397	scaffold04310	Dinucleotide	(AT)21	42	17861	17902	Ca7	Dinucleotide	(AT)16	32	21907420	21907451	upstream:Ca_18878;	ACACACAATGAAGATTGAACA	TTCATCTTTGTTTCTTTCACG
Ca_GpSSR01398	scaffold02243	Trinucleotide	(ATT)43	129	45542	45670	Ca7	Trinucleotide	(ATT)20	60	22136905	22136964	Intergenic		
Ca_GpSSR01399	scaffold45893	Dinucleotide	(AT)18	36	2	37	Ca7	Dinucleotide	(AT)22	44	22487945	22487988	Intergenic	CCTCCCTCCAATTTATACCTA	CACTTTTGCCCAACTATTTTA
Ca_GpSSR01400	scaffold14943	Trinucleotide	(TAA)10	30	8073	8102	Ca7	Trinucleotide	(TAA)11	33	22519525	22519557	Intergenic	CCTTTTGTGTAACATCCCTCT	AGCTTAGTAATTCCCCTTTTT
Ca_GpSSR01401	scaffold02451	Dinucleotide	(AT)14	28	19603	19630	Ca7	Dinucleotide	(AT)11	22	23195330	23195351	Intergenic	TCAATTTTGAACGAGAAGTGAATC	CCAAAGAGTCTACTAGCGGAACA
Ca_GpSSR01402	scaffold02989	Dinucleotide	(AT)10	20	18323	18342	Ca7	Dinucleotide	(AT)11	22	23264425	23264446	upstream:Ca_18045;	GCCCATTATATGGATATGAAAC	GGTCCTCCCATATCTATTTTC
Ca_GpSSR01403	scaffold02989	Dinucleotide	(AT)11	22	8603	8624	Ca7	Dinucleotide	(AT)10	20	23274012	23274031	Intergenic	GCCCATTATATGGATATGAAAC	GGTCCTCCCATATCTATTTTC
Ca_GpSSR01404	scaffold00325	Dinucleotide	(AT)6	12	189823	189834	Ca7	Dinucleotide	(AT)17	34	23463268	23463301	Intergenic	TTTTGGTCAAACAAATGACA	CAAGAGGCAAGGTACAATTAC
Ca_GpSSR01405	scaffold03647	Dinucleotide	(AT)11	22	403	424	Ca7	Dinucleotide	(AT)10	20	24012521	24012540	Intergenic	ACACACAATGAAGATTGAACA	TTCATCTTTGTTTCTTTCACG
Ca_GpSSR01406	scaffold03162	Dinucleotide	(AT)10	20	71391	71410	Ca7	Dinucleotide	(AT)13	26	24530702	24530727	Intergenic	TTtGTTTACAATGATATTtATGCcA	TCATTGtTCTGTGCCACttTTT
Ca_GpSSR01407	scaffold04685	Trinucleotide	(ATT)20	60	10374	10433	Ca7	Trinucleotide	(ATT)19	57	25036602	25036658	Intergenic		
Ca_GpSSR01408	scaffold07786	Dinucleotide	(AT)14	28	3600	3627	Ca7	Dinucleotide	(AT)13	26	25771321	25771346	Intergenic	ACACACAATGAAGATTGAACA	TTCATCTTTGTTTCTTTCACG
Ca_GpSSR01409	scaffold01722	Dinucleotide	(AT)17	34	60548	60581	Ca7	Dinucleotide	(AT)23	46	26097787	26097832	Intergenic	CCTCCCTCCAATTTATACCTA	CACTTTTGCCCAACTATTTTA
Ca_GpSSR01410	scaffold01722	Dinucleotide	(TA)9	18	39884	39901	Ca7	Dinucleotide	(TA)10	20	26118671	26118690	Intergenic	GCAAAAACTCAAAGGATAAAAC	AGTTAGCGGTTTGAAAAACTT
Ca_GpSSR01411	scaffold10522	Pentanucleotide	(ATTTA)7	35	3044	3078	Ca7	Pentanucleotide	(ATTTA)6	30	26907201	26907230	Intergenic		
Ca_GpSSR01412	scaffold03716	Dinucleotide	(AT)18	36	1	36	Ca7	Dinucleotide	(AT)15	30	28040578	28040607	Intergenic	ACACACAATGAAGATTGAACA	TTCATCTTTGTTTCTTTCACG
Ca_GpSSR01413	scaffold02179	Dinucleotide	(AT)31	62	1	62	Ca7	Dinucleotide	(AT)6	12	28420852	28420863	Intergenic	ACACACAATGAAGATTGAACA	TTCATCTTTGTTTCTTTCACG
Ca_GpSSR01414	scaffold00316	Dinucleotide	(TA)8	16	140503	140518	Ca7	Dinucleotide	(TA)9	18	28648460	28648477	Intergenic	CTTAAAGTTGCGAATATGGAA	CACGAACTTAGAACCGAAAC
Ca_GpSSR01415	scaffold00686	Dinucleotide	(TA)11	22	40435	40456	Ca7	Dinucleotide	(TA)10	20	29326466	29326485	Intergenic	ACACACAATGAAGATTGAACA	TTCATCTTTGTTTCTTTCACG
Ca_GpSSR01416	scaffold00143	Dinucleotide	(TA)8	16	78334	78349	Ca7	Dinucleotide	(TA)7	14	30151628	30151641	Intergenic	GGGAGCAATTAAATCGTTGT	CCTAATAAGGCTACTCCCAAG
Ca_GpSSR01417	scaffold00307	Dinucleotide	(AT)14	28	128726	128753	Ca7	Dinucleotide	(AT)9	18	30235563	30235580	Intergenic	TATCTGATGCTTGTGGATCTT	TTAGTCAGCACGAATCTTCAA
Ca_GpSSR01418	scaffold00307	Dinucleotide	(TA)11	22	75929	75950	Ca7	Dinucleotide	(TA)12	24	30287990	30288013	Intergenic	AATCAGGTTTGGTTTTGAACT	GTCATCCCCTCGTTATAAACT
Ca_GpSSR01419	scaffold00539	Dinucleotide	(TA)12	24	93728	93751	Ca7	Dinucleotide	(TA)9	18	30631979	30631996	Intergenic	ACGAACTCTTGACCCCAAAT	CCGTCATTACATAACAATTGACC
Ca_GpSSR01420	scaffold00539	Dinucleotide	(TA)19	38	29550	29587	Ca7	Dinucleotide	(TA)17	34	30700884	30700917	Intergenic	ACACACAATGAAGATTGAACA	TTCATCTTTGTTTCTTTCACG
Ca_GpSSR01421	scaffold00515	Dinucleotide	(TA)18	36	7963	7998	Ca7	Dinucleotide	(TA)6	12	31262184	31262195	Intergenic	TTACGGTATCACTTTTAGATCA	ATTAAGGCCACAAAATGAATG
Ca_GpSSR01422	scaffold02485	Dinucleotide	(AT)10	20	21511	21530	Ca7	Dinucleotide	(AT)8	16	31588638	31588653	downstream:Ca_17507;	ACACACAATGAAGATTGAACA	TTCATCTTTGTTTCTTTCACG
Ca_GpSSR01423	scaffold00494	Dinucleotide	(TA)9	18	46066	46083	Ca7	Dinucleotide	(TA)10	20	31656528	31656547	Intergenic	ACTTCGATAATTCATGGCATA	CAAGACAAATAAGAAGCAAGC
Ca_GpSSR01424	Ca_LG_7	Trinucleotide	(ATT)7	21	7669112	7669132	Ca7	Trinucleotide	(ATT)5	15	32857739	32857753	Intergenic	TTGTTGTTGATGTTGCTATTG	TCGTCTCACCATTTATCTCAT
Ca_GpSSR01425	Ca_LG_7	Dinucleotide	(TA)8	16	7683068	7683083	Ca7	Dinucleotide	(TA)9	18	32870134	32870151	Intergenic	ACACACAATGAAGATTGAACA	TTCATCTTTGTTTCTTTCACG
Ca_GpSSR01426	Ca_LG_7	Dinucleotide	(AT)14	28	8044730	8044757	Ca7	Dinucleotide	(AT)18	36	33293814	33293849	Intergenic	ACACACAATGAAGATTGAACA	TTCATCTTTGTTTCTTTCACG
Ca_GpSSR01427	scaffold35561	Dinucleotide	(TA)13	26	777	802	Ca7	Dinucleotide	(TA)12	24	33308647	33308670	Intergenic	ACACACAATGAAGATTGAACA	TTCATCTTTGTTTCTTTCACG
Ca_GpSSR01428	scaffold01531	Dinucleotide	(AT)12	24	5145	5168	Ca7	Dinucleotide	(AT)14	28	33324655	33324682	Intergenic	ACACACAATGAAGATTGAACA	TTCATCTTTGTTTCTTTCACG
Ca_GpSSR01429	scaffold01121	Trinucleotide	(AGA)5	15	65377	65391	Ca7	Trinucleotide	(AGA)6	18	33466314	33466331	CDS=Ca_15003.1.cds7;gene=Ca_15003;mRNA=Ca_15	GATCCACACCATCTAAAACAA	TGGTGTGAAAAGTAATGTGGT
Ca_GpSSR01430	scaffold00290	Dinucleotide	(TG)6	12	152226	152237	Ca7	Dinucleotide	(TG)7	14	33654420	33654433	Intergenic	TTTTCTCCCCTATGTATGTCA	GGATGATATGCTTGGCTATTA
Ca_GpSSR01431	scaffold02424	Dinucleotide	(AT)10	20	35602	35621	Ca7	Dinucleotide	(AT)32	64	33990489	33990552	Intergenic	CATGTTTATTGGGTTTTTGTC	TACTTCATGTCATTTTGCTGA
Ca_GpSSR01432	scaffold07155	Trinucleotide	(AAT)37	111	5732	5842	Ca7	Trinucleotide	(AAT)18	54	34038902	34038955	Intergenic		
Ca_GpSSR01433	Ca_LG_8	Dinucleotide	(AT)7	14	6891838	6891851	Ca7	Dinucleotide	(AT)6	12	34317536	34317547	Intergenic	ACACACAATGAAGATTGAACA	TTCATCTTTGTTTCTTTCACG
Ca_GpSSR01434	Ca_LG_8	Dinucleotide	(AT)12	24	6873098	6873121	Ca7	Dinucleotide	(AT)11	22	34340998	34341019	Intergenic	CCACTAGTATTATGGACAAATCA	GTGGTTTGCAAGGTTTTATATT
Ca_GpSSR01435	scaffold02957	Dinucleotide	(TA)17	34	35610	35643	Ca7	Dinucleotide	(TA)20	40	34520696	34520735	Intergenic	ACACACAATGAAGATTGAACA	TTCATCTTTGTTTCTTTCACG
Ca_GpSSR01436	scaffold01143	Dinucleotide	(TA)30	60	12505	12564	Ca7	Dinucleotide	(TA)10	20	34646403	34646422	Intergenic	ACACACAATGAAGATTGAACA	TTCATCTTTGTTTCTTTCACG
Ca_GpSSR01437	scaffold15585	Trinucleotide	(TAT)10	30	1067	1096	Ca7	Trinucleotide	(TAT)11	33	34672458	34672490	Intergenic	CAGGCAAGCCTTAATGTTAT	GGCAGAGTTTGTCAATAAAAC
Ca_GpSSR01438	scaffold49660	Dinucleotide	(AT)8	16	703	718	Ca7	Dinucleotide	(AT)14	28	34741876	34741903	Intergenic	ACACACAATGAAGATTGAACA	TTCATCTTTGTTTCTTTCACG
Ca_GpSSR01439	scaffold01366	Dinucleotide	(AT)8	16	54816	54831	Ca7	Dinucleotide	(AT)9	18	34766456	34766473	Intergenic	GTATGGAATCCAATTGTCAAA	TTGAGAATCCTCATAAACCAA
Ca_GpSSR01440	scaffold00538	Dinucleotide	(AT)11	22	54585	54606	Ca7	Dinucleotide	(AT)17	34	34897369	34897402	Intergenic	TGTGAGTCGTGTCACAATAAAAA	TGTTTATCAGGTTCAAGCCG
Ca_GpSSR01441	scaffold00731	Dinucleotide	(TA)17	34	114043	114076	Ca7	Dinucleotide	(TA)22	44	35384554	35384597	Intergenic	CCTCCCTCCAATTTATACCTA	CACTTTTGCCCAACTATTTTA
Ca_GpSSR01442	scaffold02924	Trinucleotide	(TAA)45	135	35990	36124	Ca7	Trinucleotide	(TAA)30	90	35521008	35521097	Intergenic		
Ca_GpSSR01443	scaffold00055	Dinucleotide	(AT)10	20	294496	294515	Ca7	Dinucleotide	(AT)11	22	35661774	35661795	mRNA=Ca_08801.1;gene=Ca_08801;intron;	GCACGGAAATAACGATTTTA	CATGGTGTTGGAGTCATACTT
Ca_GpSSR01444	scaffold14747	Trinucleotide	(ATA)19	57	1120	1176	Ca7	Trinucleotide	(ATA)15	45	35846360	35846404	Intergenic		
Ca_GpSSR01445	scaffold02656	Dinucleotide	(AT)14	28	22536	22563	Ca7	Dinucleotide	(AT)7	14	36051091	36051104	Intergenic	TGATCATTAAAGAGTGTTGCTATCAG	CGTACGTTGCACGGaAATAA
Ca_GpSSR01446	scaffold01453	Trinucleotide	(AAT)18	54	38536	38589	Ca7	Trinucleotide	(AAT)17	51	36119714	36119764	Intergenic		
Ca_GpSSR01447	scaffold09401	Trinucleotide	(TAT)6	18	27	44	Ca7	Trinucleotide	(TAT)8	24	36202771	36202794	Intergenic	TTGTTGTTGATGTTGCTATTG	TCGTCTCACCATTTATCTCAT
Ca_GpSSR01448	scaffold01284	Dinucleotide	(AT)11	22	4727	4748	Ca7	Dinucleotide	(AT)12	24	36476588	36476611	Intergenic	TGACGTTTGTATGTCTTTGTTT	CACCACTTAAGAAAAGTTTTGA
Ca_GpSSR01449	scaffold02663	Trinucleotide	(TAT)8	24	31693	31716	Ca7	Trinucleotide	(TAT)7	21	36562201	36562221	upstream:Ca_17724;	AGTGGAAAAGGTTAGCAGAAT	TGAATTATGATGGACTCCTAAG
Ca_GpSSR01450	scaffold02402	Trinucleotide	(ATT)12	36	33388	33423	Ca7	Trinucleotide	(ATT)10	30	36639639	36639668	Intergenic	GTCGTTAATTTTGCATCAATAG	AATCCTTGCTCAAAGAATATGT
Ca_GpSSR01451	scaffold03384	Dinucleotide	(AT)8	16	550	565	Ca7	Dinucleotide	(AT)7	14	36678952	36678965	Intergenic	CTTATCCATGGAAACCATTTT	TGATAAATTTGCAAGGATTC
Ca_GpSSR01452	Ca_LG_7	Dinucleotide	(AT)17	34	3374926	3374959	Ca7	Dinucleotide	(AT)19	38	36822433	36822470	Intergenic	GTGGTCGAAGATGAAAATAAA	GAGTCATTGGATGATGAAGAA
Ca_GpSSR01453	scaffold00429	Dinucleotide	(AT)21	42	69327	69368	Ca7	Dinucleotide	(AT)12	24	36963649	36963672	Intergenic	ACACACAATGAAGATTGAACA	TTCATCTTTGTTTCTTTCACG
Ca_GpSSR01454	scaffold01625	Dinucleotide	(TA)9	18	20739	20756	Ca7	Dinucleotide	(TA)10	20	37654059	37654078	Intergenic	ACACACAATGAAGATTGAACA	TTCATCTTTGTTTCTTTCACG
Ca_GpSSR01455	scaffold01997	Dinucleotide	(AT)11	22	203244	203265	Ca7	Dinucleotide	(AT)21	42	38446039	38446080	Intergenic	ACACACAATGAAGATTGAACA	TTCATCTTTGTTTCTTTCACG

Ca_GpSSR01456	scaffold05844	Dinucleotide	(TA)11	22	6190	6211	Ca7	Dinucleotide	(TA)10	20	39083005	39083024	Intergenic	ACACACAATGAAGATTGAACA	TTCATCTTTGTTTCTTTCACG
Ca_GpSSR01457	scaffold00356	Dinucleotide	(AT)11	22	214319	214340	Ca7	Dinucleotide	(AT)15	30	39118971	39119000	Intergenic	TCTTTCTTCCTTTTTCCTTTC	CGAGTATTCCTTTCTTCTTCC
Ca_GpSSR01458	scaffold00197	Trinucleotide	(ATT)25	75	34552	34626	Ca7	Trinucleotide	(ATT)21	63	39389067	39389129	Intergenic	GAAGGAAATATATCCCTTACAAGTGTG	ACGGTAAACTATAAGACGTGTCG
Ca_GpSSR01459	scaffold01546	Dinucleotide	(AT)18	36	64301	64336	Ca7	Dinucleotide	(AT)17	34	39660102	39660135	Intergenic	ACACACAATGAAGATTGAACA	TTCATCTTTGTTTCTTTCACG
Ca_GpSSR01460	scaffold03863	Dinucleotide	(AT)9	18	3680	3697	Ca7	Dinucleotide	(AT)10	20	39974352	39974371	Intergenic	ACACACAATGAAGATTGAACA	TTCATCTTTGTTTCTTTCACG
Ca_GpSSR01461	scaffold00777	Dinucleotide	(AT)11	22	13546	13567	Ca7	Dinucleotide	(AT)10	20	40151265	40151284	Intergenic	GCAAGAGTTCATAGCTGTGTC	CAGAACAAATTGTGCAATAATAG
Ca_GpSSR01462	scaffold26059	Dinucleotide	(AT)16	32	1105	1136	Ca7	Dinucleotide	(AT)14	28	40719727	40719754	Intergenic	ACACACAATGAAGATTGAACA	TTCATCTTTGTTTCTTTCACG
Ca_GpSSR01463	scaffold03073	Dinucleotide	(TA)8	16	82496	82511	Ca7	Dinucleotide	(TA)14	28	41558224	41558251	Intergenic	CACTTAGTCACACCCATTGTC	CAAAATTTGGTTTCACATACC
Ca_GpSSR01464	scaffold03073	Dinucleotide	(TA)7	14	379201	379214	Ca7	Dinucleotide	(TA)8	16	41869119	41869134	Intergenic	CACTTAGTCACACCCATTGTC	CAAAATTTGGTTTCACATACC
Ca_GpSSR01465	scaffold08002	Dinucleotide	(AG)11	22	1	22	Ca7	Dinucleotide	(AG)10	20	42585115	42585134	Intergenic	GAAAATGCAACCAATAATCTG	GCTTTGTCAATGAAGAGAATG
Ca_GpSSR01466	scaffold00561	Trinucleotide	(ATA)28	84	142108	142191	Ca7	Trinucleotide	(ATA)27	81	43273980	43274060	Intergenic	CATCGTTTATCAGTTTCATTTC	CCAACCGTTCTTAACAATATG
Ca_GpSSR01467	scaffold01716	Dinucleotide	(TA)11	22	2447	2468	Ca7	Dinucleotide	(TA)12	24	44007346	44007369	Intergenic	TTTCCATTTAGTTTGATGCT	AGCATACAATACGCATAAAT
Ca_GpSSR01468	scaffold01716	Dinucleotide	(TA)18	36	53911	53946	Ca7	Dinucleotide	(TA)17	34	44064843	44064876	Intergenic	TTTCCATTTAGTTTGATGCT	AGCATACAATACGCATAAAT
Ca_GpSSR01469	Ca_LG_7	Dinucleotide	(AT)11	22	4231995	4232016	Ca7	Dinucleotide	(AT)23	46	44752656	44752701	upstream:Ca_07656;	CCTCCCTCCAATTTATACCTA	CACTTTTGCCCAACTATTTTA
Ca_GpSSR01470	Ca_LG_7	Dinucleotide	(AT)13	26	4157545	4157570	Ca7	Dinucleotide	(AT)18	36	44863063	44863098	Intergenic	ACACACAATGAAGATTGAACA	TTCATCTTTGTTTCTTTCACG
Ca_GpSSR01471	scaffold07043	Dinucleotide	(AT)19	38	3915	3952	Ca7	Dinucleotide	(AT)17	34	45248870	45248903	Intergenic	GATATGTCAAAACACAAGATATG	TGATGGGACGATCTACTGTTA
Ca_GpSSR01472	scaffold00791	Dinucleotide	(TA)21	42	115575	115616	Ca7	Dinucleotide	(TA)17	34	45370072	45370105	Intergenic	ACACACAATGAAGATTGAACA	TTCATCTTTGTTTCTTTCACG
Ca_GpSSR01473	scaffold02077	Dinucleotide	(TA)14	28	2383	2410	Ca7	Dinucleotide	(TA)40	80	45467985	45468064	Intergenic	TGTGATATGCACGATTTGATA	TCCCTGAGAAGGATATAGACG
Ca_GpSSR01474	scaffold02077	Trinucleotide	(TAA)6	18	26040	26057	Ca7	Trinucleotide	(TAA)7	21	45491038	45491058	Intergenic		
Ca_GpSSR01475	scaffold00736	Dinucleotide	(AT)13	26	135994	136019	Ca7	Dinucleotide	(AT)7	14	45759545	45759558	Intergenic	TTTtGCTTCAAGTCATCGCA	CCTTTACGCACCCTATCGAG
Ca_GpSSR01476	scaffold01435	Dinucleotide	(AT)11	22	42087	42108	Ca7	Dinucleotide	(AT)12	24	46897489	46897512	Intergenic	ACACACAATGAAGATTGAACA	TTCATCTTTGTTTCTTTCACG
Ca_GpSSR01477	scaffold09650	Trinucleotide	(TAT)13	39	3215	3253	Ca7	Trinucleotide	(TAT)12	36	47396378	47396413	Intergenic	CAGGCAAGCCTTAATGTTAT	GGCAGAGTTTGTCAATAAAAC
Ca_GpSSR01478	scaffold01619	Dinucleotide	(AT)8	16	31714	31729	Ca7	Dinucleotide	(AT)12	24	47681276	47681299	Intergenic	TTCTTGTGTTTCTCTTTCTCG	AATTGAGTTGCAACGAGAGTA
Ca_GpSSR01479	scaffold13743	Dinucleotide	(AT)21	42	3149	3190	Ca7	Dinucleotide	(AT)17	34	47854351	47854384	Intergenic	ACACACAATGAAGATTGAACA	TTCATCTTTGTTTCTTTCACG
Ca_GpSSR01480	scaffold02888	Dinucleotide	(TA)17	34	26596	26629	Ca7	Dinucleotide	(TA)14	28	48352764	48352791	Intergenic	ACACACAATGAAGATTGAACA	TTCATCTTTGTTTCTTTCACG
Ca_GpSSR01481	Ca_LG_3	Dinucleotide	(TA)12	24	19729380	19729403	Ca8	Dinucleotide	(TA)13	26	728619	728644	upstream:Ca_03596;	ACCTCGATAAACAGTACAGACC	TGAATTTGAGTGTTAGCGTTT
Ca_GpSSR01482	Ca_LG_3	Dinucleotide	(AT)11	22	19758252	19758273	Ca8	Dinucleotide	(AT)13	26	757271	757296	Intergenic	AATCCACAAACACAACAAAAC	CCAATCAACTCATTGAGACC
Ca_GpSSR01483	Ca_LG_3	Dinucleotide	(TA)16	32	19760649	19760680	Ca8	Dinucleotide	(TA)26	52	759587	759638	Intergenic	TCAAGAACATTTCTCAAACAGT	ACTTTTTCCATCTCTTTCGTT
Ca_GpSSR01484	Ca_LG_3	Trinucleotide	(ATA)6	18	19951090	19951107	Ca8	Trinucleotide	(ATA)21	63	956530	956592	upstream:Ca_03626;		
Ca_GpSSR01485	scaffold08896	Dinucleotide	(AT)18	36	5972	6007	Ca8	Dinucleotide	(AT)8	16	1101103	1101118	Intergenic	CTAAACCCTAAACCCAACATT	TTAATCTATCTACCCACATGGT
Ca_GpSSR01486	Ca_LG_3	Dinucleotide	(TA)18	36	20147669	20147704	Ca8	Dinucleotide	(TA)21	42	1183082	1183123	Intergenic	GTATGGAGATGAAATGTGGAA	CGTAAATCAACTTCTAAATTCG
Ca_GpSSR01487	Ca_LG_3	Dinucleotide	(TA)10	20	20175269	20175288	Ca8	Dinucleotide	(TA)18	36	1402760	1402795	upstream:Ca_03658;	AATCCACAAACACAACAAAAC	CCAATCAACTCATTGAGACC
Ca_GpSSR01488	Ca_LG_3	Dinucleotide	(TA)17	34	17869439	17869472	Ca8	Dinucleotide	(TA)28	56	2055758	2055813	gene=Ca_03398;mRNA=Ca_03398.1;intron;	AGCCCCTCTTACTCTCACATA	TTGTATGTTTGCTCCGTGTAT
Ca_GpSSR01489	scaffold20257	Dinucleotide	(TA)17	34	1	34	Ca8	Dinucleotide	(TA)24	48	2315576	2315623	Intergenic	TCAAGAACATTTCTCAAACAGT	ACTTTTTCCATCTCTTTCGTT
Ca_GpSSR01490	Ca_LG_3	Dinucleotide	(AT)21	42	14980351	14980392	Ca8	Dinucleotide	(AT)13	26	2325799	2325824	Intergenic	ACCTCGATAAACAGTACAGACC	TGAATTTGAGTGTTAGCGTTT
Ca_GpSSR01491	scaffold09276	Trinucleotide	(AAT)20	60	2396	2455	Ca8	Trinucleotide	(AAT)19	57	2584041	2584097	Intergenic		
Ca_GpSSR01492	Ca_LG_3	Dinucleotide	(TA)24	48	16284451	16284498	Ca8	Dinucleotide	(TA)22	44	3119464	3119507	Intergenic	AATTTACCGCTGAAGAGTTTC	AGGGTAAACTTTAAACTCAAATG
Ca_GpSSR01493	Ca_LG_3	Trinucleotide	(ATT)29	87	16428093	16428179	Ca8	Trinucleotide	(ATT)23	69	3267938	3268006	Intergenic	TCACAGGTCCAATCATATTTAATCA	AAAAGaAATCTTAATTGAGGACAACA
Ca_GpSSR01494	Ca_LG_3	Dinucleotide	(AT)9	18	16474845	16474862	Ca8	Dinucleotide	(AT)12	24	3309930	3309953	Intergenic	AATCCACAAACACAACAAAAC	CCAATCAACTCATTGAGACC
Ca_GpSSR01495	Ca_LG_3	Dinucleotide	(TA)11	22	16478197	16478218	Ca8	Dinucleotide	(TA)10	20	3313291	3313310	Intergenic	AATCCACAAACACAACAAAAC	CCAATCAACTCATTGAGACC
Ca_GpSSR01496	Ca_LG_3	Dinucleotide	(AT)13	26	16501286	16501311	Ca8	Dinucleotide	(AT)14	28	3333008	3333035	Intergenic	ACCTCGATAAACAGTACAGACC	TGAATTTGAGTGTTAGCGTTT
Ca_GpSSR01497	Ca_LG_3	Dinucleotide	(TA)19	38	15926083	15926120	Ca8	Dinucleotide	(TA)18	36	3353457	3353492	Intergenic	GTATGGAGATGAAATGTGGAA	CGTAAATCAACTTCTAAATTCG
Ca_GpSSR01498	Ca_LG_3	Dinucleotide	(AT)12	24	15917112	15917135	Ca8	Dinucleotide	(AT)31	62	3361828	3361889	Intergenic	AGCCCCTCTTACTCTCACATA	TTGTATGTTTGCTCCGTGTAT
Ca_GpSSR01499	scaffold13022	Dinucleotide	(TA)13	26	3484	3509	Ca8	Dinucleotide	(TA)14	28	3405964	3405991	Intergenic	CAGATTCATCATCCTTAAACG	GAGTGATATGCTTAGCCCTTT
Ca_GpSSR01500	Ca_LG_3	Trinucleotide	(TAA)9	27	16667237	16667263	Ca8	Trinucleotide	(TAA)10	30	3619066	3619095	upstream:Ca_03290;		
Ca_GpSSR01501	Ca_LG_3	Dinucleotide	(TC)7	14	16959668	16959681	Ca8	Dinucleotide	(TC)8	16	3903450	3903465	upstream:Ca_03317;	TGATCCTTCAATCAAGTTTTG	TCTCTCTCTGTAAATCCGTTT
Ca_GpSSR01502	Ca_LG_3	Trinucleotide	(TTA)14	42	17053740	17053781	Ca8	Trinucleotide	(TTA)15	45	3994367	3994411	Intergenic	TATGGCCAATCTCTATGACAC	AATTTATTTTGCACCACACA
Ca_GpSSR01503	Ca_LG_3	Trinucleotide	(AAT)10	30	18287640	18287669	Ca8	Trinucleotide	(AAT)7	21	4186335	4186355	upstream:Ca_03449;	AATCTGACCATAAGTTGACGA	AGAACTATTACTGCATACCTCCA
Ca_GpSSR01504	Ca_LG_3	Dinucleotide	(TA)19	38	18380371	18380408	Ca8	Dinucleotide	(TA)27	54	4281630	4281683	upstream:Ca_03459;	AGCCCCTCTTACTCTCACATA	TTGTATGTTTGCTCCGTGTAT
Ca_GpSSR01505	Ca_LG_3	Dinucleotide	(TA)9	18	18423316	18423333	Ca8	Dinucleotide	(TA)18	36	4329872	4329907	Intergenic	GGGAGGGATGGAACCTTTTA	TCATTTGTGTGGTTGCTCTTG
Ca_GpSSR01506	scaffold20838	Dinucleotide	(AT)11	22	105	126	Ca8	Dinucleotide	(AT)14	28	4426154	4426181	Intergenic	ACCTCGATAAACAGTACAGACC	TGAATTTGAGTGTTAGCGTTT
Ca_GpSSR01507	Ca_LG_7	Dinucleotide	(AT)8	16	851006	851021	Ca8	Dinucleotide	(AT)7	14	4987846	4987859	upstream:Ca_07451;	CTAAACCCTAAACCCAACATT	TTAATCTATCTACCCACATGGT
Ca_GpSSR01508	Ca_LG_7	Dinucleotide	(TA)14	28	881518	881545	Ca8	Dinucleotide	(TA)16	32	5020792	5020823	Intergenic	GTATGGAGATGAAATGTGGAA	CGTAAATCAACTTCTAAATTCG
Ca_GpSSR01509	Ca_LG_7	Hexanucleotide	(TATATT)6	36	894166	894201	Ca8	Hexanucleotide	(TATATT)5	30	5033227	5033256	Intergenic	CACCGTAGAACTCAATGTGAT	ACTTTCACTTGATTGGTTCAA
Ca_GpSSR01510	scaffold01253	Dinucleotide	(AT)18	36	6642	6677	Ca8	Dinucleotide	(AT)20	40	5099902	5099941	Intergenic	GTATGGAGATGAAATGTGGAA	CGTAAATCAACTTCTAAATTCG
Ca_GpSSR01511	scaffold94234	Dinucleotide	(TA)7	14	1	14	Ca8	Dinucleotide	(TA)20	40	5170666	5170705	Intergenic	GTATGGAGATGAAATGTGGAA	CGTAAATCAACTTCTAAATTCG
Ca_GpSSR01512	scaffold01516	Trinucleotide	(AAT)14	42	70973	71014	Ca8	Trinucleotide	(AAT)5	15	5368882	5368896	Intergenic	CCGAATCACGCTTTAATTTAT	TTCATTTTTCTCAAACTGCAC
Ca_GpSSR01513	scaffold02163	Dinucleotide	(TA)22	44	13724	13767	Ca8	Dinucleotide	(TA)21	42	5625109	5625150	Intergenic	AGTCAGTTGATTGAATTTGAAGT	CACTTGGTTGTTAATTTTGGA
Ca_GpSSR01514	scaffold00510	Dinucleotide	(CT)8	16	99551	99566	Ca8	Dinucleotide	(CT)9	18	5666220	5666237	Intergenic	ATCCCTTCACACATTCCTAAC	TGAACGATATAAGCTCACCTG
Ca_GpSSR01515	scaffold00510	Dinucleotide	(TC)9	18	100720	100737	Ca8	Dinucleotide	(TC)10	20	5667392	5667411	upstream:Ca_12969;	TCTGAAAACACTTGTCACCTT	ACAAAACGAGGATGTGATATG
Ca_GpSSR01516	Ca_LG_3	Dinucleotide	(AT)11	22	19025144	19025165	Ca8	Dinucleotide	(AT)12	24	5780817	5780840	Intergenic	AATCCACAAACACAACAAAAC	CCAATCAACTCATTGAGACC
Ca_GpSSR01517	Ca_LG_3	Dinucleotide	(TC)6	12	19075098	19075109	Ca8	Dinucleotide	(TC)7	14	5831790	5831803	upstream:Ca_03531;	CTTAAGGTTTGGTGGAATTG	CCCTTTGTGGAATAAGTTGAT
Ca_GpSSR01518	scaffold00105	Dinucleotide	(TG)8	16	124804	124819	Ca8	Dinucleotide	(TG)7	14	6763804	6763817	Intergenic	AAGTGAATAAGAAGCACGATG	CCAAGACAGCTTTTTCCTAAT
Ca_GpSSR01519	scaffold00105	Trinucleotide	(TAA)14	42	304589	304630	Ca8	Trinucleotide	(TAA)10	30	6976160	6976189	gene=Ca_10857;mRNA=Ca_10857.1;intron;	GCGAAAAAGACAACGAAAGC	TCATCGGATTGGTCCTCTTC
Ca_GpSSR01520	Ca_LG_3	Dinucleotide	(AT)13	26	21248396	21248421	Ca8	Dinucleotide	(AT)9	18	7337941	7337958	Intergenic	ACCTCGATAAACAGTACAGACC	TGAATTTGAGTGTTAGCGTTT
Ca_GpSSR01521	Ca_LG_3	Dinucleotide	(AT)10	20	21248240	21248259	Ca8	Dinucleotide	(AT)12	24	7338133	7338156	Intergenic	AATCCACAAACACAACAAAAC	CCAATCAACTCATTGAGACC
Ca_GpSSR01522	scaffold00977	Pentanucleotide	(TAATA)30	150	111975	112124	Ca8	Pentanucleotide	(TAATA)21	105	7460728	7460832	upstream:Ca_09146;	AACGTGAGACTTCAATTTTCTT	GTCCAACCATATTCACACAAC
Ca_GpSSR01523	scaffold00977	Dinucleotide	(AT)9	18	167710	167727	Ca8	Dinucleotide	(AT)14	28	7524540	7524567	upstream:Ca_09155;	CCATAATTCATTTGCTTTGA	CGCATAACACGAACAATAGTC
Ca_GpSSR01524	scaffold00977	Trinucleotide	(AAT)5	15	200162	200176	Ca8	Trinucleotide	(AAT)14	42	7556337	7556378	upstream:Ca_09158;	ACAACAACAACAACAACAACA	TACACCGAAGTGAGATAGAGG
Ca_GpSSR01525	scaffold08412	Dinucleotide	(AT)14	28	2767	2794	Ca8	Dinucleotide	(AT)11	22	7652512	7652533	Intergenic	ACACACGAGCACATTATCTTT	AATTGATATTCCAGATGCTTG
Ca_GpSSR01526	scaffold38373	Dinucleotide	(AT)17	34	854	887	Ca8	Dinucleotide	(AT)14	28	8423479	8423506	Intergenic	ACCTCGATAAACAGTACAGACC	TGAATTTGAGTGTTAGCGTTT
Ca_GpSSR01527	scaffold02493	Dinucleotide	(TC)33	66	88	153	Ca8	Dinucleotide	(TC)29	58	8622694	8622751	Intergenic	CTCTCTCTCTCTCTCTCTCCT	CCAGTCAAACGTCAATAACAT
Ca_GpSSR01528	scaffold07930	Dinucleotide	(AG)10	20	1	20	Ca8	Dinucleotide	(AG)6	12	8740976	8740987	Intergenic	AGAaCgTTGGAAGcGGtAGA	TTCGGAGCTAGGTTTGGTTG
Ca_GpSSR01529	scaffold01260	Dinucleotide	(AT)7	14	3812	3825	Ca8	Dinucleotide	(AT)18	36	8748038	8748073	Intergenic	CTTAATCGTCCTTGTATAGTCG	GTGGATCACATGCATATTTTT
Ca_GpSSR01530	scaffold00940	Dinucleotide	(AT)23	46	9456	9501	Ca8	Dinucleotide	(AT)20	40	9102167	9102206	downstream:Ca_14529;	GTATGGAGATGAAATGTGGAA	CGTAAATCAACTTCTAAATTCG
Ca_GpSSR01531	scaffold01439	Dinucleotide	(AT)7	14	12248	12261	Ca8	Dinucleotide	(AT)16	32	9202398	9202429	Intergenic	GTATGGAGATGAAATGTGGAA	CGTAAATCAACTTCTAAATTCG
Ca_GpSSR01532	scaffold01162	Dinucleotide	(AT)16	32	37949	37980	Ca8	Dinucleotide	(AT)19	38	10010441	10010478	Intergenic	TTCCATACATCGGTCTAAATG	CCACTTATTTGTGTGATTTTCA
Ca_GpSSR01533	scaffold01999	Dinucleotide	(AT)10	20	27484	27503	Ca8	Dinucleotide	(AT)9	18	10925344	10925361	Intergenic	TTCGTAACCTAGATCAGCAAG	AAGACTTAATCAAGGAAACAGG
Ca_GpSSR01534	scaffold04957	Trinucleotide	(AAT)15	45	9040	9084	Ca8	Trinucleotide	(AAT)14	42	11469098	11469139	Intergenic	CCGAATCACGCTTTAATTTAT	TTCATTTTTCTCAAACTGCAC
Ca_GpSSR01535	scaffold01033	Dinucleotide	(TA)8	16	23695	23710	Ca8	Dinucleotide	(TA)9	18	11571713	11571730	Intergenic	ACCTCGATAAACAGTACAGACC	TGAATTTGAGTGTTAGCGTTT
Ca_GpSSR01536	scaffold01033	Dinucleotide	(CT)24	48	40372	40419	Ca8	Dinucleotide	(CT)29	58	11587246	11587303	mRNA=Ca_14797.1;gene=Ca_14797;intron;		

Ca_GpSSR01537	scaffold05539	Dinucleotide	(AT)12	24	7781	7804	Ca8	Dinucleotide	(AT)11	22	11858011	11858032	Intergenic	CACATGACATTCTTTTTAACG	AGGTGTGCATAGGTGTACAAA
Ca_GpSSR01538	scaffold04506	Dinucleotide	(TC)19	38	8462	8499	Ca8	Dinucleotide	(TC)10	20	11875732	11875751	upstream:Ca_18982;	AGCTTTTAAAGGGTTCTTTTG	TTGTGTGTATGTGTGTGAGTGT
Ca_GpSSR01539	scaffold00911	Hexanucleotide	(AAATAT)6	36	140201	140236	Ca8	Hexanucleotide	(AAATAT)7	42	12406708	12406749	Intergenic	GAAACCAAcATGATATgATAAGAaCAA	TGAAAAAGACAAAAATACCCTTATAGT
Ca_GpSSR01540	scaffold00911	Trinucleotide	(TTA)46	138	195177	195314	Ca8	Trinucleotide	(TTA)19	57	12460561	12460617	Intergenic	GGTTCTTATATTTCCAATTCCA	GACCACACTAGCAACAAAAAC
Ca_GpSSR01541	scaffold01834	Dinucleotide	(AT)15	30	16055	16084	Ca8	Dinucleotide	(AT)14	28	12614257	12614284	Intergenic	ACCTCGATAAACAGTACAGACC	TGAATTTGAGTGTTAGCGTTT
Ca_GpSSR01542	scaffold00938	Dinucleotide	(AT)14	28	36301	36328	Ca8	Dinucleotide	(AT)13	26	12723729	12723754	Intergenic	ACCTCGATAAACAGTACAGACC	TGAATTTGAGTGTTAGCGTTT
Ca_GpSSR01543	Ca_LG_3	Dinucleotide	(AT)10	20	1581794	1581813	Ca8	Dinucleotide	(AT)17	34	13899613	13899646	Intergenic	AATCCACAAACACAACAAAAC	CCAATCAACTCATTGAGACC
Ca_GpSSR01544	scaffold02527	Dinucleotide	(AT)16	32	14827	14858	Ca8	Dinucleotide	(AT)24	48	14211657	14211704	Intergenic	GAGAATAAAAaTGTTACAGCAAAATCA	AAAGGTTCAAGCAGGGTGAAT
Ca_GpSSR01545	scaffold00119	Dinucleotide	(AT)10	20	72332	72351	Ca8	Dinucleotide	(AT)9	18	14317520	14317537	Intergenic	ACCTCGATAAACAGTACAGACC	TGAATTTGAGTGTTAGCGTTT
Ca_GpSSR01546	scaffold00119	Dinucleotide	(AT)15	30	84062	84091	Ca8	Dinucleotide	(AT)16	32	14329930	14329961	Intergenic	GTATGGAGATGAAATGTGGAA	CGTAAATCAACTTCTAAATTCG
Ca_GpSSR01547	scaffold00119	Dinucleotide	(AT)7	14	276894	276907	Ca8	Dinucleotide	(AT)14	28	14524418	14524445	downstream:Ca_10941;	GCTTGTTTTCTTAAGTTGCCACA	tTGAATcATTaAACaAAAtTTCCTG
Ca_GpSSR01548	scaffold23092	Dinucleotide	(TA)21	42	2	43	Ca8	Dinucleotide	(TA)23	46	14552823	14552868	Intergenic	TCAAGAACATTTCTCAAACAGT	ACTTTTTCCATCTCTTTCGTT
Ca_GpSSR01549	scaffold04799	Dinucleotide	(AT)9	18	2656	2673	Ca8	Dinucleotide	(AT)10	20	14909790	14909809	Intergenic	TGGTGAGGTGAAATTTTCTAA	TTGGTTCACATATAGCATTCA
Ca_GpSSR01550	scaffold13511	Dinucleotide	(TA)6	12	220	231	Ca8	Dinucleotide	(TA)7	14	15024009	15024022	Intergenic	GCATTTAAAACTTGGACTTCTT	TTGTAATGGAGATTCCTCAAA
Ca_GpSSR01551	scaffold01523	Dinucleotide	(AT)12	24	68116	68139	Ca8	Dinucleotide	(AT)20	40	15234735	15234774	upstream:Ca_15996;	TGCAATCTATTGCTACACAAATCA	CATGGCTAGTGCAATTCTCA
Ca_GpSSR01552	scaffold00497	Dinucleotide	(TA)11	22	75549	75570	Ca8	Dinucleotide	(TA)13	26	15437503	15437528	upstream:Ca_12906;	GTGGGAAGCATCTAGAAAAAT	CTTCTAACCCTTTTCAAAACC
Ca_GpSSR01553	scaffold02932	Trinucleotide	(TAA)41	123	15	137	Ca8	Trinucleotide	(TAA)31	93	15551214	15551306	Intergenic		
Ca_GpSSR01554	scaffold02932	Dinucleotide	(TA)12	24	27957	27980	Ca8	Dinucleotide	(TA)14	28	15578939	15578966	upstream:Ca_18004;	TTTTGACCAGACTTTACTTGC	TTTCTCTTGAACAGCATGAGT
Ca_GpSSR01555	Ca_LG_3	Dinucleotide	(AT)10	20	22861426	22861445	Ca8	Dinucleotide	(AT)9	18	15722416	15722433	upstream:Ca_03835;mRNA=Ca_03834.1;gene=Ca_03	AATCCACAAACACAACAAAAC	CCAATCAACTCATTGAGACC
Ca_GpSSR01556	Ca_LG_3	Dinucleotide	(AT)20	40	22876510	22876549	Ca8	Dinucleotide	(AT)21	42	15737489	15737530	mRNA=Ca_03834.1;gene=Ca_03834;intron;	GTATGGAGATGAAATGTGGAA	CGTAAATCAACTTCTAAATTCG
Ca_GpSSR01557	scaffold02701	Trinucleotide	(TAT)19	57	28679	28735	Ca8	Trinucleotide	(TAT)7	21	16098448	16098468	Intergenic	TAAAaCCTtATTTtgCCGGA	GGGATAACGCCAAGGACATa
Ca_GpSSR01558	Ca_LG_2	Dinucleotide	(AT)16	32	2176264	2176295	C11070128	Dinucleotide	(AT)17	34	1357	1390	mRNA=Ca_01190.1;gene=Ca_01190;intron;		
Ca_GpSSR01559	Ca_LG_5	Trinucleotide	(TTG)8	24	15912666	15912689	C11132224	Trinucleotide	(TTG)6	18	3717	3734	Intergenic	ATTTCCCTTTGTAAAGCAATC	TAACAACAACAACAACGACAA
Ca_GpSSR01560	scaffold04019	Dinucleotide	(TA)18	36	69	104	C11145012	Dinucleotide	(TA)17	34	20	53	Intergenic		
Ca_GpSSR01561	scaffold00492	Dinucleotide	(TA)15	30	143138	143167	C11145408	Dinucleotide	(TA)13	26	228	253	Intergenic	CGTAGGGCTAAAATTAAAAGC	TACGGGGACAAAATTACAATA
Ca_GpSSR01562	scaffold05266	Dinucleotide	(TA)13	26	3942	3967	scaffold10227	Dinucleotide	(TA)26	52	3116	3167	Intergenic	CCATTTATGTATGGTCAAGATTT	GTAAAATAGACACTGCCCTCA
Ca_GpSSR01563	scaffold01092	Dinucleotide	(AT)16	32	12172	12203	scaffold1047	Dinucleotide	(AT)19	38	512148	512185	Intergenic	GAGGGAGGACATACACTTGAT	AGGCTAGGTTTGTAATTGTTG
Ca_GpSSR01564	scaffold01092	Trinucleotide	(GAA)12	36	39479	39514	scaffold1047	Trinucleotide	(GAA)11	33	540316	540348	Intergenic	AAAGAGTAAAACTCCGAGTGC	AAAGGGTGGAAACTGTTACAT
Ca_GpSSR01565	scaffold00820	Trinucleotide	(TAT)28	84	109084	109167	scaffold1050	Trinucleotide	(TAT)19	57	56913	56969	Intergenic	TTGGTTTCCAAATCATGTTA	TGTTGAAGGTGAAGTAGTAGACA
Ca_GpSSR01566	scaffold01027	Dinucleotide	(AT)15	30	49523	49552	scaffold1055	Dinucleotide	(AT)14	28	256218	256245	Intergenic	TTGATGAATTTATGGTCACA	AAGTTTTGGGAAAGGACAATA
Ca_GpSSR01567	scaffold15734	Dinucleotide	(TA)12	24	902	925	scaffold1060	Dinucleotide	(TA)15	30	87056	87085	Intergenic	GTCGTGCATCATAAAGGTACT	CACCGAAACTTTATTTGATCTT
Ca_GpSSR01568	scaffold01488	Dinucleotide	(TA)24	48	51264	51311	scaffold1060	Dinucleotide	(TA)13	26	410385	410410	Intergenic	CAAGCTTTTCAACCATAAGAT	CGTCACCACATGTAAGACTG
Ca_GpSSR01569	scaffold02612	Dinucleotide	(TA)18	36	1367	1402	scaffold1067	Dinucleotide	(TA)22	44	187698	187741	Intergenic	ATAAATGGCCCACTTGATG	GCATTAGAAAATTGAATGTTAGG
Ca_GpSSR01570	scaffold05105	Dinucleotide	(AT)22	44	203038	203081	scaffold1067	Dinucleotide	(AT)20	40	70309	70348	upstream:Ca_09407;	GGATCGCATATGTAATTTAACC	AGTTAACTTCAGTTCTCGAAT
Ca_GpSSR01571	scaffold00472	Dinucleotide	(TA)20	40	133126	133165	scaffold1089	Dinucleotide	(TA)10	20	140862	140881	Intergenic	TAATGCGTTATGGGTGAAAT	TTCTTCCTTTTTCCTTCTTTC
Ca_GpSSR01572	scaffold06282	Dinucleotide	(AT)9	18	3781	3798	scaffold10	Dinucleotide	(AT)12	24	13761	13784	Intergenic	GTCTCATCCGTTGAATCTAAA	ACATGCACCACTTTAATGTTT
Ca_GpSSR01573	scaffold62284	Dinucleotide	(AT)21	42	416	457	scaffold1115	Dinucleotide	(AT)18	36	19678	19713	Intergenic		
Ca_GpSSR01574	scaffold01449	Dinucleotide	(TA)19	38	34912	34949	scaffold111	Dinucleotide	(TA)13	26	62883	62908	Intergenic	CTTGGTTGGACATATTGTTTC	GGCGTTTGACTACATGATAAA
Ca_GpSSR01575	scaffold28057	Trinucleotide	(TAA)46	138	1	138	scaffold118	Trinucleotide	(TAA)12	36	194051	194086	Intergenic		
Ca_GpSSR01576	scaffold01024	Dinucleotide	(AT)7	14	1	14	scaffold118	Dinucleotide	(AT)17	34	216115	216148	Intergenic	AATTGGGTGTAACCAATAAGC	TGATAGGAAGAACTAAATATTGG
Ca_GpSSR01577	scaffold01024	Tetranucleotide	(ATTT)8	32	27023	27054	scaffold118	Tetranucleotide	(ATTT)9	36	243248	243283	Intergenic	GGTGGTAAGGGATTAATTGAC	AGACAAAAATGTTGAACGAGA
Ca_GpSSR01578	scaffold03837	Dinucleotide	(AT)12	24	18204	18227	scaffold1196	Dinucleotide	(AT)18	36	17355	17390	Intergenic	AAAGACTATCTTCGACACGTAA	CTTCGAGATCAGAAAACTTGT
Ca_GpSSR01579	Ca_LG_3	Dinucleotide	(AT)11	22	14164397	14164418	scaffold1202	Dinucleotide	(AT)13	26	54723	54748	Intergenic	AATCCACAAACACAACAAAAC	CCAATCAACTCATTGAGACC
Ca_GpSSR01580	scaffold38814	Trinucleotide	(TTA)26	78	809	886	scaffold1231	Trinucleotide	(TTA)16	48	148830	148877	Intergenic		
Ca_GpSSR01581	scaffold03295	Trinucleotide	(TTC)14	42	11509	11550	scaffold1281	Trinucleotide	(TTC)33	99	12719	12817	Intergenic		
Ca_GpSSR01582	scaffold00252	Dinucleotide	(AT)23	46	117368	117413	scaffold1281	Dinucleotide	(AT)24	48	234317	234364	Intergenic	TATGAGTTGGATGATGTGTCA	ATGGTTTATGATTGGTTCTCA
Ca_GpSSR01583	scaffold00252	Dinucleotide	(AT)11	22	82151	82172	scaffold1281	Dinucleotide	(AT)10	20	263983	264002	Intergenic	TCATATGCTTTGTGTCATGTG	TTTCACTCGTACTTTAATCATCA
Ca_GpSSR01584	scaffold00966	Trinucleotide	(TTA)19	57	28174	28230	scaffold1281	Trinucleotide	(TTA)20	60	405120	405179	upstream:Ca_14635;	GGTTTAAATGGGTCTATACGG	GGACCATCTTGAACCTTATGT
Ca_GpSSR01585	scaffold79759	Trinucleotide	(TTA)46	138	331	468	scaffold128	Trinucleotide	(TTA)27	81	583820	583900	Intergenic		
Ca_GpSSR01586	scaffold09324	Dinucleotide	(AT)8	16	1466	1481	scaffold1324	Dinucleotide	(AT)9	18	728299	728316	Intergenic	CCAAATCTAGCATGCATTAGT	AATAATTGGGAATCGAGGTTA
Ca_GpSSR01587	scaffold00346	Dinucleotide	(TA)13	26	92394	92419	scaffold1324	Dinucleotide	(TA)12	24	91239	91262	Intergenic	AACGGGTAACGAACTAACTAA	ACTTGTGTTCACACACACACA
Ca_GpSSR01588	scaffold00346	Dinucleotide	(AT)6	12	35321	35332	scaffold1324	Dinucleotide	(AT)7	14	36550	36563	Intergenic	TGACTAACAAATTTTCCTTCG	CCTCCATTTCAATCTCTACATC
Ca_GpSSR01589	scaffold01321	Dinucleotide	(TA)19	38	80458	80495	scaffold134	Dinucleotide	(TA)8	16	120786	120801	Intergenic	TGTGAGACGAAGAGCAATTAT	CACATCATATAAAAGCGATCA
Ca_GpSSR01590	scaffold01265	Trinucleotide	(ATA)19	57	5606	5662	scaffold134	Trinucleotide	(ATA)18	54	328783	328836	Intergenic	TCGGTGCTTTTGTCAATATAA	TATACATGCATTACAGCCAAA
Ca_GpSSR01591	scaffold01265	Dinucleotide	(TA)7	14	22439	22452	scaffold134	Dinucleotide	(TA)25	50	344773	344822	gene=Ca_15387;mRNA=Ca_15387.1;intron;	CAAATCGTGAAATGAAATACG	GAAGGGATAATTGGAAAGAGA
Ca_GpSSR01592	scaffold00469	Dinucleotide	(AT)19	38	14135	14172	scaffold1351	Dinucleotide	(AT)24	48	209543	209590	upstream:Ca_12755;		
Ca_GpSSR01593	scaffold01508	Trinucleotide	(ATA)17	51	48250	48300	scaffold1439	Trinucleotide	(ATA)14	42	181874	181915	upstream:Ca_15958;	TGGTTTAACACAAAGTACCAAA	TACGATCGAGACTAGACCAAA
Ca_GpSSR01594	scaffold03595	Trinucleotide	(ATT)26	78	13070	13147	scaffold1439	Trinucleotide	(ATT)19	57	24764	24820	Intergenic		
Ca_GpSSR01595	scaffold13666	Dinucleotide	(AT)9	18	2611	2628	scaffold1439	Dinucleotide	(AT)8	16	229155	229170	Intergenic	CGCAAATGCTTGATAAAAAT	TTTTGGTGACCGTATAGAAAA
Ca_GpSSR01596	scaffold13666	Trinucleotide	(ATA)6	18	3210	3227	scaffold1439	Trinucleotide	(ATA)19	57	229751	229807	Intergenic		
Ca_GpSSR01597	scaffold01155	Dinucleotide	(AT)15	30	131106	131135	scaffold1466	Dinucleotide	(AT)22	44	109389	109432	Intergenic	GCATATAAGCATTGGTGTTTT	TGAAGCATAACATGACATGAA
Ca_GpSSR01598	scaffold01155	Trinucleotide	(AAT)22	66	31347	31412	scaffold1466	Trinucleotide	(AAT)27	81	208515	208595	mRNA=Ca_10039.1;gene=Ca_10039;intron;	TCAACTCATAAATAAACGGATTTACC	TcCCTAAtTTTAAGaAAGGaAgGAG
Ca_GpSSR01599	scaffold02104	Dinucleotide	(TA)10	20	1303	1322	scaffold1466	Dinucleotide	(TA)11	22	17607	17628	Intergenic	GAATTCCTTTAGCCACTTTTC	TCAAATGTTACAGGCAACTCT
Ca_GpSSR01600	scaffold00588	Dinucleotide	(AT)17	34	112003	112036	scaffold1467	Dinucleotide	(AT)10	20	52804	52823	Intergenic	TGGAATACACCTTGTGCTAAT	CCGTCTATTCATTTTTACTACCA
Ca_GpSSR01601	scaffold00430	Dinucleotide	(TA)11	22	173928	173949	scaffold1475	Dinucleotide	(TA)12	24	93866	93889	Intergenic	TAGAACCAACCTTGAAACAAA	AAGTGGATTTTAGTCGATTGG
Ca_GpSSR01602	scaffold01175	Dinucleotide	(AT)8	16	25688	25703	scaffold1506	Dinucleotide	(AT)9	18	47395	47412	Intergenic	AAAGAGACATGACTTTGTCCA	AATATTGGCAGATGAATGAT
Ca_GpSSR01603	scaffold00792	Dinucleotide	(AT)12	24	8248	8271	scaffold1545	Dinucleotide	(AT)10	20	208549	208568	Intergenic	CTTTTCCTTTTCTTTCCAAAC	CCTTTACGGAGAATTTTATGC
Ca_GpSSR01604	scaffold06434	Trinucleotide	(AAT)12	36	1986	2021	scaffold157	Trinucleotide	(AAT)29	87	182844	182930	Intergenic	GGGATTAAAATTGAAAGGTT	GGGACTATATATGACCCTAGTT
Ca_GpSSR01605	scaffold00664	Dinucleotide	(AT)6	12	69978	69989	scaffold157	Dinucleotide	(AT)10	20	11933	11952	mRNA=Ca_13610.1;gene=Ca_13610;intron;	TTACAAGTCGCTATTTAGAACG	GTTACAGCCAGCTCTAATCAA
Ca_GpSSR01606	scaffold06356	Trinucleotide	(TTA)18	54	2365	2418	scaffold1626	Trinucleotide	(TTA)17	51	132025	132075	Intergenic	CGAAAACACTTGTGATTGTTA	ACACCTAATTTTTGTCCGATT
Ca_GpSSR01607	scaffold01382	Trinucleotide	(ATT)33	99	60773	60871	scaffold1631	Trinucleotide	(ATT)19	57	78628	78684	Intergenic	CTATGGAATTAGGGATTTTCC	TGTGCTTGTTATGGTGTACCT
Ca_GpSSR01608	scaffold24549	Dinucleotide	(AT)15	30	316	345	scaffold1631	Dinucleotide	(AT)12	24	182111	182134	Intergenic	ACACTCCATTCTAATTTGCAG	AGACAAAACAAACGTGAACAT
Ca_GpSSR01609	scaffold02253	Trinucleotide	(TTA)10	30	33972	34001	scaffold1632	Trinucleotide	(TTA)12	36	19873	19908	Intergenic		
Ca_GpSSR01610	scaffold02164	Trinucleotide	(AAT)12	36	15661	15696	scaffold1633	Trinucleotide	(AAT)11	33	15744	15776	Intergenic	TGAACGTCATCATACTACGTG	TGGGGATGTGTTATATCATTG
Ca_GpSSR01611	scaffold03774	Dinucleotide	(TA)9	18	745	762	scaffold1643	Dinucleotide	(TA)7	14	147215	147228	Intergenic	AGACGGATAATTTGTTTTTG	TCCATTCACCTTTTCTAGTGA
Ca_GpSSR01612	scaffold02333	Trinucleotide	(ATA)8	24	25760	25783	scaffold166	Trinucleotide	(ATA)6	18	35140	35157	Intergenic	ACACCTAATTTTGTCCGATTT	CGAAAACACTTGTGATTGTTA
Ca_GpSSR01613	scaffold04855	Dinucleotide	(AT)8	16	6928	6943	scaffold167	Dinucleotide	(AT)22	44	228304	228347	Intergenic	GAAACAAATGAGCTTAAAATCC	GTTCCACAAGCTTCACTACTAA
Ca_GpSSR01614	scaffold05735	Dinucleotide	(TA)13	26	1914	1939	scaffold167	Dinucleotide	(TA)8	16	307472	307487	Intergenic		
Ca_GpSSR01615	scaffold04069	Dinucleotide	(AT)11	22	6120	6141	scaffold1708	Dinucleotide	(AT)16	32	8925	8956	Intergenic		
Ca_GpSSR01616	Ca_LG_4	Dinucleotide	(AT)16	32	2587028	2587059	scaffold1710	Dinucleotide	(AT)17	34	50885	50918	Intergenic	TCTTATCACTGAATTGACACA	CAAACTTCACCAATACCACAT
Ca_GpSSR01617	scaffold02201	Dinucleotide	(TA)9	18	7794	7811	scaffold171	Dinucleotide	(TA)8	16	217309	217324	Intergenic	AATGAAATTCGGTGTCAAAT	AGGGACATAGCTCTTATTTCAA

Ca_GpSSR01618	Ca_LG_7	Dinucleotide	(AG)8	16	3127778	3127793	scaffold174	Dinucleotide	(AG)7	14	461834	461847	gene=Ca_07599;mRNA=Ca_07599.1;intron;	ATAAAGGCATAAGCAGAAGGT	TACAATTCAAAACGAACTTGG
Ca_GpSSR01619	scaffold03902	Trinucleotide	(TTA)28	84	10	93	scaffold174	Trinucleotide	(TTA)31	93	465106	465198	Intergenic		
Ca_GpSSR01620	scaffold00880	Dinucleotide	(AT)7	14	71955	71968	scaffold1751	Dinucleotide	(AT)9	18	470875	470892	Intergenic	ATACCGTTTTTCTTCGTTTTC	GAGAGGTTATGGGTAAAATGG
Ca_GpSSR01621	scaffold14054	Dinucleotide	(AT)18	36	3029	3064	scaffold1751	Dinucleotide	(AT)12	24	518155	518178	Intergenic	AAATGGTTTGAGAAGTGATGA	TCTGTTCCTTCTTTCCTTTCT
Ca_GpSSR01622	scaffold08277	Dinucleotide	(AT)10	20	518	537	scaffold1757	Dinucleotide	(AT)9	18	2888	2905	Intergenic	TATCGATATGGCTGGAATGTA	CCAAATCACCCTCTAATTTTT
Ca_GpSSR01623	scaffold01790	Dinucleotide	(AT)10	20	40078	40097	scaffold1757	Dinucleotide	(AT)9	18	86967	86984	Intergenic	AATGCTCATAACAAAGCAAAC	CAAAAATTGTTTTTCCCTCT
Ca_GpSSR01624	scaffold01286	Trinucleotide	(TAT)39	117	7394	7510	scaffold1771	Trinucleotide	(TAT)33	99	30146	30244	Intergenic	AGAAAAACCTATTAGGCATGA	AACTAGCAAGAATGCAACAAC
Ca_GpSSR01625	Ca_LG_3	Trinucleotide	(TAT)19	57	13265737	13265793	scaffold1777	Trinucleotide	(TAT)18	54	193677	193730	downstream:Ca_03071;		
Ca_GpSSR01626	scaffold02775	Dinucleotide	(TA)12	24	27233	27256	scaffold17	Dinucleotide	(TA)14	28	188503	188530	Intergenic	CCATAGCAAAGGTCATAGAAA	CCTCCCTATTTTTGTGTTTTT
Ca_GpSSR01627	scaffold03874	Dinucleotide	(AT)32	64	1942	2005	scaffold1804	Dinucleotide	(AT)24	48	23957	24004	Intergenic	CCTTTGGTTTTGAAATAGAATAG	AAAAACCATTGGGATCAAA
Ca_GpSSR01628	scaffold00747	Trinucleotide	(ATA)24	72	31121	31192	scaffold1867	Trinucleotide	(ATA)21	63	188245	188307	Intergenic	TCAATCATTAACACTAACACTCC	GCTGTTTTCTGTAACATCCTAA
Ca_GpSSR01629	scaffold01303	Dinucleotide	(TA)12	24	15837	15860	scaffold1922	Dinucleotide	(TA)17	34	17271	17304	Intergenic	AAATAGCAAGTCTGCAACAAC	TGATCTGATTTGGTCTAGCAT
Ca_GpSSR01630	scaffold03229	Dinucleotide	(TA)8	16	22257	22272	scaffold1928	Dinucleotide	(TA)7	14	109331	109344	Intergenic	TCCTTTTCCTTTCTTTTTCC	GGTTATGGGTGAAATGGTAAT
Ca_GpSSR01631	Ca_LG_4	Dinucleotide	(AT)9	18	6801135	6801152	scaffold1943	Dinucleotide	(AT)10	20	192566	192585	Intergenic	CTTAGGCATTTAGCCTTATTTT	ATGTCCGGTATTAAACTTTCC
Ca_GpSSR01632	Ca_LG_4	Trinucleotide	(AAT)13	39	6868048	6868086	scaffold1943	Trinucleotide	(AAT)8	24	271962	271985	Intergenic		
Ca_GpSSR01633	Ca_LG_4	Dinucleotide	(TA)16	32	6931512	6931543	scaffold1943	Dinucleotide	(TA)21	42	338677	338718	Intergenic		
Ca_GpSSR01634	Ca_LG_4	Dinucleotide	(AC)18	36	6945890	6945925	scaffold1943	Dinucleotide	(AC)7	14	354278	354291	Intergenic	TTAGTGAAGCGATGGGTATAA	CTCTTCCTTTCTCAGTTTTCC
Ca_GpSSR01635	scaffold02107	Dinucleotide	(AT)9	18	1391	1408	scaffold1962	Dinucleotide	(AT)10	20	60885	60904	gene=Ca_16935;mRNA=Ca_16935.1;intron;	GCTAGTGGGTATGCTTGTGTA	ATAGGGAGCCTATCAAAAGAG
Ca_GpSSR01636	scaffold01979	Dinucleotide	(TA)24	48	35618	35665	scaffold1964	Dinucleotide	(TA)12	24	132537	132560	gene=Ca_10415;mRNA=Ca_10415.1;intron;	AAGAAAATTTTGCCCTATACC	TACTTCTGTTCCGGAAATGTT
Ca_GpSSR01637	scaffold40894	Trinucleotide	(TTA)36	108	1	108	scaffold1981	Trinucleotide	(TTA)20	60	99417	99476	Intergenic	TAGGTGTCAACAGCCTAACTG	CAATTGTAATTTTTGCGACA
Ca_GpSSR01638	scaffold01340	Dinucleotide	(AT)19	38	24804	24841	scaffold198	Dinucleotide	(AT)6	12	572816	572827	Intergenic	GAAATGCCAATTTTTCAAAG	CATTGTCCAAATGTTATAGCC
Ca_GpSSR01639	scaffold00259	Dinucleotide	(TA)7	14	19677	19690	scaffold198	Dinucleotide	(TA)18	36	76414	76449	Intergenic	TTCACTAGCATCTCGCTATTT	TCAACTTACCATATTGTGTTTTC
Ca_GpSSR01640	scaffold01110	Trinucleotide	(TAT)26	78	10473	10550	scaffold198	Trinucleotide	(TAT)21	63	112567	112629	upstream:Ca_14973;	TGATGTTAATTTGTGTCCGTA	TGCATTCTGGTGAGAGTATTT
Ca_GpSSR01641	scaffold01792	Trinucleotide	(TTA)23	69	36357	36425	scaffold198	Trinucleotide	(TTA)24	72	40486	40557	Intergenic	TCGTCGAATTCATCACTAACT	TTTTCTCAATTTTCTCCAGGT
Ca_GpSSR01642	scaffold02269	Dinucleotide	(AT)19	38	47206	47243	scaffold1991	Dinucleotide	(AT)26	52	30381	30432	Intergenic		
Ca_GpSSR01643	scaffold02269	Dinucleotide	(TA)20	40	46736	46775	scaffold1991	Dinucleotide	(TA)15	30	31230	31259	Intergenic	TTAAGCGAAGTAACTCAAACAA	AGCGCACAGTTGATAAATTC
Ca_GpSSR01644	scaffold90149	Dinucleotide	(TA)15	30	364	393	scaffold202	Dinucleotide	(TA)16	32	7033	7064	Intergenic		
Ca_GpSSR01645	Ca_LG_5	Dinucleotide	(AT)9	18	14994482	14994499	scaffold2039	Dinucleotide	(AT)8	16	147936	147951	Intergenic	CTTCTTTACATCTGCTCTCCA	GCGCAAAATTCCTTTTAGTAT
Ca_GpSSR01646	scaffold03276	Dinucleotide	(TA)24	48	19	66	scaffold2051	Dinucleotide	(TA)20	40	230684	230723	Intergenic	TCTTGGCACGTTTTTAATTT	TGTGAGAACACCTTCAACACT
Ca_GpSSR01647	scaffold21594	Dinucleotide	(AT)14	28	726	753	scaffold206	Dinucleotide	(AT)15	30	61033	61062	Intergenic	CAACTCACTACTCGTATCCATTT	GTTTTGAGGTTCAATGTGTGT
Ca_GpSSR01648	scaffold04730	Dinucleotide	(AT)19	38	15261	15298	scaffold208	Dinucleotide	(AT)17	34	94372	94405	Intergenic	CAAGTATGAAACAACCTTCCA	TCTATTAATCGTGATAGGTCAAC
Ca_GpSSR01649	scaffold00526	Dinucleotide	(TA)23	46	35739	35784	scaffold208	Dinucleotide	(TA)56	112	359974	360085	Intergenic	TAAACCATACTTGCTGCAAAC	TGTCTAACAACCAATCCAGTT
Ca_GpSSR01650	scaffold03519	Trinucleotide	(AAT)8	24	26222	26245	scaffold212	Trinucleotide	(AAT)19	57	403268	403324	Intergenic		
Ca_GpSSR01651	scaffold02707	Dinucleotide	(TA)18	36	3861	3896	scaffold2152	Dinucleotide	(TA)6	12	321221	321232	Intergenic	AAACCGTAACCCTATGAACTC	AGCTCGGAAAACTTGTAAAAC
Ca_GpSSR01652	scaffold03223	Trinucleotide	(TAA)15	45	26117	26161	scaffold221	Trinucleotide	(TAA)16	48	88187	88234	Intergenic	CCCTCTTTAGCATAAATAATTG	ACATCAAAACAGCATCACAA
Ca_GpSSR01653	scaffold11766	Dinucleotide	(GA)6	12	2555	2566	scaffold223	Dinucleotide	(GA)7	14	38498	38511	Intergenic	GCCAGCACAACCTAGTAGTTA	CAATTATATGATCAAACCGTTC
Ca_GpSSR01654	scaffold01165	Trinucleotide	(ATT)6	18	85466	85483	scaffold2269	Trinucleotide	(ATT)11	33	137298	137330	Intergenic		
Ca_GpSSR01655	scaffold02325	Trinucleotide	(AAT)21	63	47139	47201	scaffold2311	Trinucleotide	(AAT)35	105	57210	57314	Intergenic		
Ca_GpSSR01656	Ca_LG_2	Dinucleotide	(TA)21	42	14794030	14794071	scaffold2330	Dinucleotide	(TA)8	16	92875	92890	Intergenic	TTACCGAATATGGAACGACTA	TAGGCCTGACAGACCAAC
Ca_GpSSR01657	Ca_LG_2	Dinucleotide	(AG)13	26	14766404	14766429	scaffold2330	Dinucleotide	(AG)17	34	65266	65299	mRNA=Ca_01972.1;gene=Ca_01972;intron;	GACAAGCCTCCGTGTGATTT	ATCGACGTCGCTAAATGCTT
Ca_GpSSR01658	scaffold03279	Trinucleotide	(AAT)7	21	11249	11269	scaffold2371	Trinucleotide	(AAT)6	18	10397	10414	Intergenic	TGAAACGGTTACCGAATATAG	CCTAACTTGATAGACCGACCT
Ca_GpSSR01659	scaffold91156	Dinucleotide	(AT)9	18	267	284	scaffold2392	Dinucleotide	(AT)10	20	228894	228913	Intergenic	CTTAACGGTAGCAAAAACGTA	CCACCCCTAGTACTTTTACGA
Ca_GpSSR01660	scaffold01743	Trinucleotide	(TAT)19	57	30255	30311	scaffold2392	Trinucleotide	(TAT)17	51	252027	252077	Intergenic		
Ca_GpSSR01661	scaffold00634	Dinucleotide	(AT)18	36	173597	173632	scaffold242	Dinucleotide	(AT)6	12	677494	677505	Intergenic	TCCTTTCTTTCTATTCTCTCCA	GAGAATTTAATGCGTTACGG
Ca_GpSSR01662	scaffold00634	Dinucleotide	(TA)19	38	174367	174404	scaffold242	Dinucleotide	(TA)16	32	677643	677674	Intergenic	CGTTCTCATATCAACCAAAAA	TGATTATGTTCTCCTTCCTTG
Ca_GpSSR01663	scaffold00634	Dinucleotide	(TA)12	24	192481	192504	scaffold242	Dinucleotide	(TA)7	14	696951	696964	upstream:Ca_09503;	TCCTTTCTTTCTATTCTCTCCA	GAGAATTTAATGCGTTACGG
Ca_GpSSR01664	scaffold01614	Dinucleotide	(TA)24	48	30853	30900	scaffold242	Dinucleotide	(TA)21	42	369943	369984	Intergenic	GAGTTGCGACGAGAGTAGATA	TTTCAGCTTTCTGTTTCCTTA
Ca_GpSSR01665	scaffold49416	Dinucleotide	(TA)20	40	53	92	scaffold242	Dinucleotide	(TA)13	26	410541	410566	Intergenic	CGTTCTCATATCAACCAAAAA	TGATTATGTTCTCCTTCCTTG
Ca_GpSSR01666	scaffold01142	Dinucleotide	(TA)22	44	64182	64225	scaffold2516	Dinucleotide	(TA)7	14	54148	54161	Intergenic	AGTTTGCGTAGCTGTGTTAAT	AATTTGAGGATTGTACGTGAA
Ca_GpSSR01667	scaffold06513	Trinucleotide	(AAT)9	27	7897	7923	scaffold2534	Trinucleotide	(AAT)8	24	40898	40921	Intergenic		
Ca_GpSSR01668	scaffold06513	Trinucleotide	(AAT)18	54	7996	8049	scaffold2534	Trinucleotide	(AAT)21	63	40994	41056	Intergenic		
Ca_GpSSR01669	scaffold00636	Trinucleotide	(TTA)23	69	2851	2919	scaffold2558	Trinucleotide	(TTA)19	57	8292	8348	Intergenic	CGACACCTATTCCTCTTTTCT	AGTTAGTTGAGTCTTGTGAAAAA
Ca_GpSSR01670	scaffold01317	Dinucleotide	(AT)19	38	6352	6389	scaffold2617	Dinucleotide	(AT)18	36	202060	202095	Intergenic	CTCAAATCAAACCCTTATCTTT	TTTATTGATCTGACGGTTATGA
Ca_GpSSR01671	scaffold01873	Trinucleotide	(TTA)15	45	8898	8942	scaffold263	Trinucleotide	(TTA)13	39	101812	101850	Intergenic	CTCGGAGTCTGATAAAAGGTT	TGATGTTGTAACACCCAATTT
Ca_GpSSR01672	scaffold02520	Dinucleotide	(AT)9	18	35644	35661	scaffold2720	Dinucleotide	(AT)8	16	160033	160048	Intergenic	GGAACCCTAATATCCTCTACG	CAACAAGTTTCAAAAAGAAACC
Ca_GpSSR01673	scaffold02468	Dinucleotide	(TA)11	22	8628	8649	scaffold275	Dinucleotide	(TA)7	14	98523	98536	Intergenic	ATTACCACATCGTAGAAAGCA	GCTTGGTGTACCAGTTCAGTA
Ca_GpSSR01674	scaffold05661	Trinucleotide	(ATA)32	96	11409	11504	scaffold284	Trinucleotide	(ATA)14	42	744186	744227	Intergenic	AACAATGCAGTACTATCATCCA	ATTATGGTACGAGTGTGTTGG
Ca_GpSSR01675	scaffold01662	Dinucleotide	(TA)10	20	39490	39509	scaffold287	Dinucleotide	(TA)23	46	301261	301306	gene=Ca_16247;mRNA=Ca_16247.1;intron;	TACCCCTTCTTTGTAGCTTTT	TGTTCATATGTGTATTGGTGAA
Ca_GpSSR01676	scaffold00969	Dinucleotide	(TA)11	22	63887	63908	scaffold290	Dinucleotide	(TA)18	36	88867	88902	Intergenic	GCTTTCAAATCTTAGTCGCTAT	ACAATACTCCATTTGGTCACA
Ca_GpSSR01677	scaffold18408	Dinucleotide	(AT)35	70	12	81	scaffold2916	Dinucleotide	(AT)16	32	44462	44493	Intergenic		
Ca_GpSSR01678	scaffold18408	Dinucleotide	(TA)13	26	1855	1880	scaffold2916	Dinucleotide	(TA)20	40	46203	46242	Intergenic		
Ca_GpSSR01679	scaffold00888	Trinucleotide	(TAA)17	51	47507	47557	scaffold2916	Trinucleotide	(TAA)23	69	51940	52008	Intergenic	TGGAATTCATTGTATTTTGG	AATGAATTGAGATGAGCCTTT
Ca_GpSSR01680	scaffold00888	Dinucleotide	(TA)11	22	61856	61877	scaffold2916	Dinucleotide	(TA)10	20	65934	65953	downstream:Ca_14392;		
Ca_GpSSR01681	scaffold15459	Trinucleotide	(TTA)30	90	2399	2488	scaffold2916	Trinucleotide	(TTA)32	96	23152	23247	Intergenic		
Ca_GpSSR01682	scaffold03800	Dinucleotide	(AT)13	26	10517	10542	scaffold2955	Dinucleotide	(AT)10	20	32718	32737	Intergenic	GTGGTTTAATGAAAAACAAGC	TTCTCAACCATTAGAGTTTGG
Ca_GpSSR01683	scaffold22657	Dinucleotide	(AT)15	30	7	36	scaffold296	Dinucleotide	(AT)20	40	298042	298081	Intergenic	TGCTCGTGGAAGTTAACTAAT	CTTTTTCAAGGGAAAAGTGTT
Ca_GpSSR01684	scaffold01391	Dinucleotide	(CT)9	18	69833	69850	scaffold296	Dinucleotide	(CT)12	24	474416	474439	gene=Ca_15688;mRNA=Ca_15688.1;intron;	TGCGTTGTCGTTTTTGtGTT	AAATCTAATCGATTCCCGGC
Ca_GpSSR01685	scaffold05316	Dinucleotide	(AT)9	18	70767	70784	scaffold2	Dinucleotide	(AT)25	50	433707	433756	downstream:Ca_10154;	TGGAGTTGTTTCAAATTGTTC	TGCTGCAGATATGGTTAAGTT
Ca_GpSSR01686	scaffold01266	Dinucleotide	(AT)9	18	53181	53198	scaffold3016	Dinucleotide	(AT)10	20	50453	50472	Intergenic	TTTCAAGTTTGAGTGTGTGGT	CAGATAATTCATCTCGCTAGG
Ca_GpSSR01687	scaffold01266	Dinucleotide	(AT)15	30	69328	69357	scaffold3016	Dinucleotide	(AT)14	28	59265	59292	Intergenic	GTCATTCCAAATGAAGACTTG	TACCAACGATTTTAGGTTCAA
Ca_GpSSR01688	scaffold03690	Trinucleotide	(TAA)8	24	17825	17848	scaffold303	Trinucleotide	(TAA)9	27	74642	74668	Intergenic		
Ca_GpSSR01689	scaffold08262	Dinucleotide	(TA)9	18	6286	6303	scaffold306	Dinucleotide	(TA)22	44	425182	425225	Intergenic	TCAAGTGTAAGCATGGTGAAT	CAAGTTTCAAATGGTGACTTT
Ca_GpSSR01690	Ca_LG_2	Dinucleotide	(CT)9	18	7928914	7928931	scaffold306	Dinucleotide	(CT)10	20	50571	50590	Intergenic	CAAAAGAAGTGGTGGTTGTAG	TATTATGCTACTTGGGACGTT
Ca_GpSSR01691	scaffold03341	Dinucleotide	(TA)7	14	30422	30435	scaffold314	Dinucleotide	(TA)38	76	142048	142123	Intergenic	CGCTTTAGGTTTTGTTCATTA	GTTATTTGTCAGTTGATTTAAGC
Ca_GpSSR01692	scaffold03331	Dinucleotide	(AT)15	30	7045	7074	scaffold3321	Dinucleotide	(AT)9	18	64428	64445	Intergenic	TCCTTTAGCATAACTCTCATTG	AAAAGGAATGCTTCGTCTTTA
Ca_GpSSR01693	scaffold01571	Dinucleotide	(AT)15	30	1698	1727	scaffold334	Dinucleotide	(AT)20	40	173797	173836	Intergenic	AACTTACCACACTAGCATAGAA	TGTGATTGGAGATAGACAAAGA
Ca_GpSSR01694	scaffold25543	Trinucleotide	(ATT)16	48	1015	1062	scaffold336	Trinucleotide	(ATT)14	42	616162	616203	Intergenic		
Ca_GpSSR01695	scaffold05182	Dinucleotide	(AT)9	18	1143	1160	scaffold336	Dinucleotide	(AT)8	16	617876	617891	upstream:Ca_19251;	CAACGTGCATAAACAAAAAT	CTTTCGTGACTTTAAGGAACA
Ca_GpSSR01696	scaffold06672	Dinucleotide	(TA)12	24	3296	3319	scaffold340	Dinucleotide	(TA)9	18	175478	175495	Intergenic	AGCCTTGTGAACAGATTGATA	ACCTTACGACGAATTACGG
Ca_GpSSR01697	scaffold09898	Dinucleotide	(AT)8	16	2387	2402	scaffold342	Dinucleotide	(AT)9	18	45646	45663	Intergenic	AAGCGAAGTAACTCATCACAA	CCCAAGAGTTATTAGATGGAGA
Ca_GpSSR01698	scaffold00371	Tetranucleotide	(ATAA)7	28	20078	20105	scaffold3432	Tetranucleotide	(ATAA)6	24	119514	119537	Intergenic	TGTGTGTGTGAATAGCTTGAA	TTAAGGGACTGCGGATATAAT

Ca_GpSSR01699	scaffold32236	Trinucleotide	(TAT)27	81	961	1041	scaffold3500	Trinucleotide	(TAT)15	45	31496	31540	Intergenic		
Ca_GpSSR01700	scaffold05976	Dinucleotide	(TG)11	22	5254	5275	scaffold3520	Dinucleotide	(TG)10	20	11868	11887	Intergenic	GTCTGCCTCCTATTTTATGGT	TCGGGTAACCAATATAGTCTG
Ca_GpSSR01701	scaffold15871	Dinucleotide	(AT)17	34	1	34	scaffold362	Dinucleotide	(AT)10	20	316446	316465	Intergenic	TTCTTCACCTTTTCATTATGG	TGGTGATTCTCACAACAAAA
Ca_GpSSR01702	Ca_LG_5	Dinucleotide	(TA)15	30	16251775	16251804	scaffold362	Dinucleotide	(TA)23	46	342876	342921	Intergenic	GTTGAATTGGAGTTGTCAAAG	TCCAGAAACGGTACTTTATTTT
Ca_GpSSR01703	scaffold03542	Dinucleotide	(TA)11	22	16118	16139	scaffold363	Dinucleotide	(TA)10	20	69948	69967	Intergenic	GTACCGTCGAAATATTTTCCT	GAGAAATGTTTTGTTATGAAGTG
Ca_GpSSR01704	scaffold03542	Trinucleotide	(AAT)22	66	2528	2593	scaffold363	Trinucleotide	(AAT)21	63	56097	56159	Intergenic		
Ca_GpSSR01705	scaffold115517	Trinucleotide	(TAA)8	24	1	24	scaffold363	Trinucleotide	(TAA)10	30	63947	63976	Intergenic		
Ca_GpSSR01706	scaffold22485	Dinucleotide	(AT)6	12	1458	1469	scaffold36	Dinucleotide	(AT)8	16	309428	309443	Intergenic	ATAAAGAATATGCTCGACACG	ATAAAGAATATGCTCGACACG
Ca_GpSSR01707	scaffold02627	Trinucleotide	(AAT)24	72	27472	27543	scaffold374	Trinucleotide	(AAT)20	60	106806	106865	Intergenic	GGTCCATGTGTGTAAATGAGT	AAGCGATAGCTTGTAACACTTA
Ca_GpSSR01708	scaffold28254	Dinucleotide	(TA)7	14	1007	1020	scaffold377	Dinucleotide	(TA)9	18	153066	153083	Intergenic	ATGCAAGCATTTTGTGTAAGT	CCTGAACCCAAATTGAAATA
Ca_GpSSR01709	scaffold01079	Trinucleotide	(ACA)9	27	27792	27818	scaffold379	Trinucleotide	(ACA)5	15	30328	30342	Intergenic	CTGAATATCGGACATCAATTT	TGTTGTTGTTGTTGTTGTTGT
Ca_GpSSR01710	scaffold03051	Tetranucleotide	(TGAA)5	20	14961	14980	scaffold379	Tetranucleotide	(TGAA)7	28	193230	193257	Intergenic	GGCTCTGCTAGGTACATGAAT	GATTTGAATTGTCGTATCCAA
Ca_GpSSR01711	scaffold03051	Trinucleotide	(TAA)19	57	29201	29257	scaffold379	Trinucleotide	(TAA)15	45	210499	210543	Intergenic	TAGAAAACAACGTCACGAAAT	AAAAGATACAATCCGTGAACA
Ca_GpSSR01712	scaffold09056	Dinucleotide	(AT)22	44	43	86	scaffold38	Dinucleotide	(AT)14	28	157748	157775	Intergenic	CCATTCTCTAAAGATAGTGGTA	GAAAGGAAAATCTTGAGTCTGA
Ca_GpSSR01713	scaffold00573	Trinucleotide	(TAT)12	36	97855	97890	scaffold38	Trinucleotide	(TAT)16	48	192533	192580	Intergenic		
Ca_GpSSR01714	scaffold00573	Dinucleotide	(AG)32	64	132593	132656	scaffold38	Dinucleotide	(AG)7	14	224552	224565	Intergenic		
Ca_GpSSR01715	scaffold07595	Dinucleotide	(AT)17	34	7391	7424	scaffold38	Dinucleotide	(AT)12	24	343516	343539	Intergenic	CCATTCTCTAAAGATAGTGGTA	GAAAGGAAAATCTTGAGTCTGA
Ca_GpSSR01716	scaffold01737	Dinucleotide	(AT)9	18	36481	36498	scaffold38	Dinucleotide	(AT)8	16	466084	466099	gene=Ca_10373;mRNA=Ca_10373.1;intron;	GGGTTTTGAATTGACATGTTT	GATTATCAGCTTTGTTGGTTG
Ca_GpSSR01717	scaffold03098	Dinucleotide	(TA)11	22	6714	6735	scaffold395	Dinucleotide	(TA)10	20	273212	273231	Intergenic	AGGGCTAGTTGTACACATGAA	TGTGGTCGAAATGTTCTTAAT
Ca_GpSSR01718	scaffold02875	Dinucleotide	(TA)16	32	11755	11786	scaffold397	Dinucleotide	(TA)24	48	28648	28695	Intergenic	AAATAATTGTCTTGAGATAGTGG	CTCACATTAAAATACTACCATCA
Ca_GpSSR01719	Ca_LG_3	Dinucleotide	(TA)22	44	1789989	1790032	scaffold398	Dinucleotide	(TA)13	26	109981	110006	Intergenic	AATTTACCGCTGAAGAGTTTC	AGGGTAAACTTTAAACTCAAATG
Ca_GpSSR01720	Ca_LG_3	Dinucleotide	(TC)9	18	1816633	1816650	scaffold398	Dinucleotide	(TC)8	16	135160	135175	Intergenic	GGGGAGGACTAGATGTAACAG	TGCTCAACCAAAAATAAGTTC
Ca_GpSSR01721	Ca_LG_3	Trinucleotide	(AAT)19	57	1900350	1900406	scaffold398	Trinucleotide	(AAT)12	36	252118	252153	Intergenic	GCAGACTGTGAAGATTCTTTG	CACATATTATCTGGAAAAACCA
Ca_GpSSR01722	Ca_LG_3	Dinucleotide	(TA)18	36	1909396	1909431	scaffold398	Dinucleotide	(TA)12	24	261123	261146	Intergenic	GGTGCCTTCTTATTCAGACTT	GTTGCAGGGAAAAGAGATTC
Ca_GpSSR01723	scaffold06108	Dinucleotide	(TA)24	48	54	101	scaffold398	Dinucleotide	(TA)7	14	300436	300449	Intergenic	TGATGTGTTGTGAAGTGACAT	TTGCTTCCTTTCTTCTTTTCT
Ca_GpSSR01724	scaffold01188	Trinucleotide	(TAT)14	42	54721	54762	scaffold40	Trinucleotide	(TAT)11	33	496476	496508	downstream:Ca_15169;	TGTTGTTTACACACCGTAAGA	AAGGAAAATCATTCTTAGTCCA
Ca_GpSSR01725	scaffold00212	Dinucleotide	(AT)23	46	188136	188181	scaffold40	Dinucleotide	(AT)16	32	601181	601212	downstream:Ca_08886;	TGTAAATGTAGATGATTGTTTGC	ACAAACTGATTCACGAAATTG
Ca_GpSSR01726	scaffold00212	Dinucleotide	(AT)10	20	154130	154149	scaffold40	Dinucleotide	(AT)11	22	635144	635165	Intergenic	ACTTTCTCTTTGGTTGTAACG	ATCAACTTACCTCCCTAGCAT
Ca_GpSSR01727	scaffold03156	Dinucleotide	(TA)9	18	3073	3090	scaffold40	Dinucleotide	(TA)10	20	326092	326111	Intergenic	TCTTGTGTTAATGTGTTGTGC	CAAAAAGTAAGGGTCATCTCC
Ca_GpSSR01728	scaffold03780	Dinucleotide	(AT)19	38	5577	5614	scaffold411	Dinucleotide	(AT)28	56	221703	221758	Intergenic	TATTTCCTTTTCCTTTCCCTA	TATCATTGGCATACCCTTCTA
Ca_GpSSR01729	Ca_LG_4	Trinucleotide	(ATA)10	30	16683910	16683939	scaffold418	Trinucleotide	(ATA)9	27	107400	107426	Intergenic	GTGTAACATCCCTCTTTAGCA	TCCCCTTACTCTTTTCTTTTC
Ca_GpSSR01730	scaffold05848	Dinucleotide	(AT)11	22	6673	6694	scaffold418	Dinucleotide	(AT)15	30	158677	158706	Intergenic	TAACATTGATCCCGTAAGAAA	AAACAAACGGACGATCTTAAT
Ca_GpSSR01731	scaffold20127	Dinucleotide	(TA)8	16	422	437	scaffold419	Dinucleotide	(TA)10	20	263512	263531	Intergenic	GGTTATGGGTGAAATGGTAAT	TTTCCTTTTCCTTCCTTCTAA
Ca_GpSSR01732	scaffold02975	Dinucleotide	(AT)6	12	26269	26280	scaffold419	Dinucleotide	(AT)12	24	277899	277922	Intergenic	GGTTATGGGTGAAATGGTAAT	TTTCCTTTTCCTTCCTTCTAA
Ca_GpSSR01733	Ca_LG_2	Trinucleotide	(TTA)18	54	3891070	3891123	scaffold420	Trinucleotide	(TTA)17	51	109390	109440	Intergenic	AATTTCCTCCCTTTCTTTTCT	GATGCAACATCATTCTTTAGC
Ca_GpSSR01734	scaffold04853	Dinucleotide	(AT)19	38	1049	1086	scaffold433	Dinucleotide	(AT)12	24	96087	96110	Intergenic	TTCTTTTATCCCCAAACAAA	CGAAGAATTTAATGCGTTATG
Ca_GpSSR01735	scaffold02314	Dinucleotide	(TA)14	28	21244	21271	scaffold435	Dinucleotide	(TA)6	12	32700	32711	Intergenic	TCTCACTCTCTCATTGTAAAGC	CATCTCTACTTTGTATATGCTTC
Ca_GpSSR01736	scaffold01194	Dinucleotide	(TA)12	24	63466	63489	scaffold4452	Dinucleotide	(TA)11	22	44783	44804	Intergenic	GAAAAAGCAATGTTTTGAAC	TCACTCTAAATGAATTCTTCTCT
Ca_GpSSR01737	scaffold02672	Dinucleotide	(TA)11	22	19272	19293	scaffold44	Dinucleotide	(TA)12	24	148630	148653	Intergenic	TCTATTCTCTCCAAGCAAACA	GAGAATTTAATGCGTTACGG
Ca_GpSSR01738	scaffold13468	Dinucleotide	(AT)9	18	1486	1503	scaffold4508	Dinucleotide	(AT)12	24	21692	21715	Intergenic	AAATGCCGACTTTCTGAAG	TCGATGTCACATTCAACATTA
Ca_GpSSR01739	scaffold00587	Dinucleotide	(AT)6	12	39455	39466	scaffold450	Dinucleotide	(AT)7	14	299665	299678	Intergenic	GTGTGAAATAATCAAATGCGATA	ATGTGTCAACCAAATAATCA
Ca_GpSSR01740	scaffold04778	Trinucleotide	(AAT)13	39	14165	14203	scaffold451	Trinucleotide	(AAT)19	57	324313	324369	Intergenic	AGGGTTTTGTTGACACCTAAT	CACTTGTGATTGTCTAAAGCA
Ca_GpSSR01741	scaffold03453	Dinucleotide	(AT)9	18	854	871	scaffold453	Dinucleotide	(AT)10	20	357849	357868	Intergenic	GAATATTTTCACCCACTTCATT	TGGAGTGACTAGAGTTTATTCGT
Ca_GpSSR01742	Ca_LG_7	Trinucleotide	(ATA)19	57	4328997	4329053	scaffold470	Trinucleotide	(ATA)23	69	179090	179158	Intergenic		
Ca_GpSSR01743	scaffold02160	Dinucleotide	(TA)7	14	32902	32915	scaffold4759	Dinucleotide	(TA)8	16	19725	19740	gene=Ca_17025;mRNA=Ca_17025.1;intron;	GTTGTGTAATTGCCAGAAGTC	AAATTCTTATGCGTCACTCAA
Ca_GpSSR01744	scaffold17909	Dinucleotide	(AT)11	22	71	92	scaffold4837	Dinucleotide	(AT)10	20	350	369	Intergenic	TGTGCAACATATTCTGTCTCT	TGTGACACATGATCACTTAAAA
Ca_GpSSR01745	scaffold03686	Trinucleotide	(ATT)24	72	16430	16501	scaffold484	Trinucleotide	(ATT)25	75	765758	765832	Intergenic	TCCTCGAGAATTTATTTTACCT	GAATTTGACAATGCAATACT
Ca_GpSSR01746	scaffold06271	Trinucleotide	(TAA)12	36	2060	2095	scaffold48	Trinucleotide	(TAA)11	33	114311	114343	Intergenic	TGTTTGTTCCCTTTCTTTTAAC	CCATTATCATTCTCGTTTGAA
Ca_GpSSR01747	scaffold00376	Dinucleotide	(AT)6	12	122951	122962	scaffold48	Dinucleotide	(AT)14	28	268202	268229	Intergenic	CCTTGTTTTCACATAAATCCA	AATGAATTTTAACCGAGTGGT
Ca_GpSSR01748	scaffold06526	Dinucleotide	(AT)10	20	401	420	scaffold4923	Dinucleotide	(AT)13	26	9446	9471	Intergenic	CCGTTAAACACGCATATTACT	TTATTATTTGCGTTCATGTG
Ca_GpSSR01749	scaffold05278	Dinucleotide	(AT)12	24	12374	12397	scaffold495	Dinucleotide	(AT)9	18	97174	97191	Intergenic		
Ca_GpSSR01750	scaffold02661	Dinucleotide	(AT)11	22	22377	22398	scaffold498	Dinucleotide	(AT)13	26	81128	81153	Intergenic	GGAAAATGCGAAATTTTTGA	GGCCAATATATTTAACGTTTGTA
Ca_GpSSR01751	scaffold01973	Dinucleotide	(TC)7	14	10440	10453	scaffold498	Dinucleotide	(TC)10	20	16265	16284	Intergenic	AGCCAAATTTTTATTGTACCC	GAAGGAAAGAGAGAGTGAGAAA
Ca_GpSSR01752	scaffold04464	Dinucleotide	(TA)18	36	2666	2701	scaffold506	Dinucleotide	(TA)26	52	37656	37707	Intergenic		
Ca_GpSSR01753	scaffold02368	Trinucleotide	(TTA)13	39	21932	21970	scaffold50	Trinucleotide	(TTA)12	36	86755	86790	Intergenic		
Ca_GpSSR01754	scaffold02368	Dinucleotide	(TC)8	16	33104	33119	scaffold50	Dinucleotide	(TC)7	14	97926	97939	mRNA=Ca_17337.1;exon=Ca_17337.1.exon1;5'-UTR=	AAAAGGGTTTTTCTCTCTCAA	ACTCATTTCTCAACTGTCTCG
Ca_GpSSR01755	Ca_LG_2	Trinucleotide	(ATA)7	21	11597899	11597919	scaffold50	Trinucleotide	(ATA)9	27	113244	113270	Intergenic		
Ca_GpSSR01756	scaffold03485	Dinucleotide	(AT)15	30	16208	16237	scaffold50	Dinucleotide	(AT)19	38	467449	467486	Intergenic	AAATCACACATCAATTTCACC	GGGAATTAAGTACCAAAACTTG
Ca_GpSSR01757	scaffold00370	Dinucleotide	(AT)12	24	75516	75539	scaffold528	Dinucleotide	(AT)11	22	53141	53162	Intergenic	GTAGTGCTCTTGGGTTTGTTA	CATATGTTTCGATGATGTTGA
Ca_GpSSR01758	Ca_LG_3	Dinucleotide	(AT)16	32	18580721	18580752	scaffold528	Dinucleotide	(AT)25	50	238824	238873	upstream:Ca_03474;	GCATGTTGATTTTGATAAGGA	CTTCTTCTGATGCCATGACT
Ca_GpSSR01759	scaffold05948	Dinucleotide	(AG)47	94	8494	8587	scaffold528	Dinucleotide	(AG)35	70	308678	308747	Intergenic	TCTTGTTTTGTGTTTTCGAGT	TGTGTCATCATCATCTCTCTCT
Ca_GpSSR01760	scaffold07074	Trinucleotide	(AAT)23	69	4207	4275	scaffold531	Trinucleotide	(AAT)17	51	66441	66491	Intergenic	ACAGCACGTAAAAACAACAAT	CCAGTCCTTCCAAGTTGTTA
Ca_GpSSR01761	scaffold34995	Trinucleotide	(AAT)13	39	458	496	scaffold5376	Trinucleotide	(AAT)15	45	525	569	Intergenic	TTCTTATACATGTGTTTTTGGTG	ATATGATGCCTAGCAATTCAA
Ca_GpSSR01762	scaffold54924	Trinucleotide	(ATT)34	102	1	102	scaffold537	Trinucleotide	(ATT)20	60	129099	129158	Intergenic	CTGGAATGTAATAATGTGTTGC	TTTTACACATTTACTCAACCGTA
Ca_GpSSR01763	Ca_LG_2	Dinucleotide	(TA)18	36	14300409	14300444	scaffold537	Dinucleotide	(TA)13	26	137443	137468	Intergenic	GGGATTTAAGGTTTCACACTT	CGTAATTTATGGGTTTGGTTT
Ca_GpSSR01764	Ca_LG_3	Dinucleotide	(AT)17	34	4494216	4494249	scaffold545	Dinucleotide	(AT)22	44	60503	60546	Intergenic	AACACAATCATGTGTAACCAA	CACAAGGTAGTTTTGTCTTTTG
Ca_GpSSR01765	scaffold15970	Trinucleotide	(AAT)33	99	2260	2358	scaffold548	Trinucleotide	(AAT)26	78	121628	121705	Intergenic		
Ca_GpSSR01766	scaffold03023	Trinucleotide	(AAT)5	15	73573	73587	scaffold553	Trinucleotide	(AAT)13	39	657792	657830	Intergenic		
Ca_GpSSR01767	scaffold03023	Dinucleotide	(AT)10	20	104385	104404	scaffold553	Dinucleotide	(AT)9	18	757202	757219	Intergenic	TGTCTCTGTGACACCCTAAAC	TTTTGTGAAACGTGATATCCT
Ca_GpSSR01768	scaffold05177	Dinucleotide	(AT)6	12	10060	10071	scaffold553	Dinucleotide	(AT)13	26	880451	880476	Intergenic	GCAAGCCAATTATTGTAGGTA	TGTACGACTATTGGGTTGTTT
Ca_GpSSR01769	scaffold00498	Tetranucleotide	(AAAT)9	36	224903	224938	scaffold553	Tetranucleotide	(AAAT)7	28	247367	247394	Intergenic	CGAAAAATAATTTGCTGTAGG	ACGATATCTTCTACATTCTCTTT
Ca_GpSSR01770	scaffold07038	Dinucleotide	(AT)18	36	4262	4297	scaffold553	Dinucleotide	(AT)11	22	281670	281691	Intergenic	GGTAATAAAGAACAAGGAAAGAA	TCGGTTTAGCATCATGTTATC
Ca_GpSSR01771	scaffold00874	Dinucleotide	(AT)11	22	40620	40641	scaffold562	Dinucleotide	(AT)10	20	58652	58671	upstream:Ca_09308;	CATAATCACATTTTAACACATGC	TTCATAAGGAACAAGGTGAAA
Ca_GpSSR01772	Ca_LG_7	Dinucleotide	(TA)10	20	3839496	3839515	scaffold5714	Dinucleotide	(TA)20	40	22394	22433	Intergenic	CAACGGGTTTCGAGTTTC	GTGACATCCACCCCACAC
Ca_GpSSR01773	scaffold118654	Trinucleotide	(TAT)10	30	2	31	scaffold583	Trinucleotide	(TAT)5	15	13727	13741	Intergenic		
Ca_GpSSR01774	scaffold19721	Dinucleotide	(TA)12	24	1586	1609	scaffold584	Dinucleotide	(TA)14	28	74750	74777	Intergenic	AACACATTTTCAAACGAGAGA	TTATTGTTATCGTCGTCTTTCA
Ca_GpSSR01775	Ca_LG_4	Dinucleotide	(AT)10	20	6716699	6716718	scaffold598	Dinucleotide	(AT)8	16	517369	517384	mRNA=Ca_04294.1;gene=Ca_04294;intron;	TGCATATGGATCTGAATGTAGT	ACCATAAGCAGTTCACACAAT
Ca_GpSSR01776	scaffold01023	Dinucleotide	(TA)8	16	153065	153080	scaffold599	Dinucleotide	(TA)7	14	183544	183557	Intergenic	GGTGAAGGCAAAATCTAAAAT	TACACACACACACACACAC
Ca_GpSSR01777	scaffold25880	Trinucleotide	(AAT)18	54	142	195	scaffold6055	Trinucleotide	(AAT)7	21	279	299	Intergenic	CGCATGTGTAAGAAAATATCA	GGTTTATTTTAATCCATTGTGG
Ca_GpSSR01778	scaffold30415	Trinucleotide	(TAA)5	15	545	559	scaffold6304	Trinucleotide	(TAA)6	18	816	833	Intergenic	CTGTAGTACCTAATTTTTGTCCA	CTGGTTGCAGATCAATTAAAA
Ca_GpSSR01779	scaffold09785	Trinucleotide	(TAT)32	96	5076	5171	scaffold642	Trinucleotide	(TAT)34	102	567390	567491	Intergenic		

Ca_GpSSR01780	scaffold31231	Trinucleotide	(TAT)56	168	901	1068	scaffold661	Trinucleotide	(TAT)33	99	284166	284264	Intergenic		TGGAACCCACCAATTTTAC	CAAGCCCATTAATTTTGTTT
Ca_GpSSR01781	scaffold00378	Dinucleotide	(AT)10	20	168761	168780	scaffold674	Dinucleotide	(AT)6	12	268478	268489	mRNA=Ca_12359.1;gene=Ca_12359;intron;		ATGATTTGGGCATTGTTTAG	GGATGCACAAGATTGATTAAA
Ca_GpSSR01782	scaffold81997	Dinucleotide	(TA)14	28	1	28	scaffold674	Dinucleotide	(TA)21	42	390116	390157	Intergenic		GTTGTGCTAAATGAGGTGAAG	GATCATCCAATTCATCCATAA
Ca_GpSSR01783	scaffold00401	Dinucleotide	(AT)9	18	142094	142111	scaffold674	Dinucleotide	(AT)7	14	457377	457390	Intergenic		TGGGTTCTTCAGCAATAAGTA	ATTAGGATTCGGTCCAATTAC
Ca_GpSSR01784	scaffold02968	Dinucleotide	(TA)6	12	9228	9239	scaffold674	Dinucleotide	(TA)22	44	621657	621700	Intergenic		GTTGTGCTAAATGAGGTGAAG	GATCATCCAATTCATCCATAA
Ca_GpSSR01785	scaffold14615	Trinucleotide	(ATA)10	30	2803	2832	scaffold674	Trinucleotide	(ATA)13	39	682206	682244	Intergenic			
Ca_GpSSR01786	scaffold00378	Trinucleotide	(ATA)11	33	34745	34777	scaffold674	Trinucleotide	(ATA)19	57	118047	118103	Intergenic			
Ca_GpSSR01787	scaffold03282	Dinucleotide	(TA)11	22	18002	18023	scaffold716	Dinucleotide	(TA)10	20	304231	304250	Intergenic		CTCACAACACAACCATTTTTC	GGTGCTCAACTTGTTTGTTTA
Ca_GpSSR01788	scaffold22120	Dinucleotide	(TA)6	12	139	150	scaffold766	Dinucleotide	(TA)7	14	175236	175249	Intergenic		TTTCCTTTCTATTTCCTTTCC	TGGGGGTAAGAGTTGTTAGAT
Ca_GpSSR01789	scaffold03406	Dinucleotide	(TA)17	34	12315	12348	scaffold7800	Dinucleotide	(TA)12	24	12007	12030	Intergenic		CAAAATGAACGCATGATTAAC	TTATGTAGATCAGAATCAGACTT
Ca_GpSSR01790	scaffold04854	Dinucleotide	(AT)10	20	779	798	scaffold793	Dinucleotide	(AT)9	18	254315	254332	Intergenic		AAAAACCTTTGAGAAATCGAG	GGCATTTCGAATGAGTTATATT
Ca_GpSSR01791	scaffold03802	Trinucleotide	(TTC)13	39	19844	19882	scaffold7	Trinucleotide	(TTC)196	588	70037	70624	Intergenic		GAGCTCTCAAAAGTTTTATCG	TCCATACAATCGAGAAGAAGA
Ca_GpSSR01792	scaffold03839	Dinucleotide	(AT)9	18	1268	1285	scaffold801	Dinucleotide	(AT)11	22	198017	198038	Intergenic		AGACGAGACTAGTATCACTCCTT	GTAGTACCCCAATTTTGTGTG
Ca_GpSSR01793	scaffold39046	Dinucleotide	(TA)16	32	2	33	scaffold80	Dinucleotide	(TA)11	22	451133	451154	Intergenic		GTAATGTTCTGTTTGATTTCCTT	CATTAGAGGATGGTCGAAACT
Ca_GpSSR01794	scaffold13257	Trinucleotide	(ATT)13	39	940	978	scaffold812	Trinucleotide	(ATT)11	33	311739	311771	Intergenic		AGTGGGTTTTATGAATATGTTG	GCAAAATTCATTGGTAATGATG
Ca_GpSSR01795	scaffold02693	Trinucleotide	(ATT)5	15	14986	15000	scaffold845	Trinucleotide	(ATT)6	18	47663	47680	upstream:Ca_17755;		GCGGCTAGTTTTAGAATGAGT	AGAAAGTAAAGAAGGGAGTTT
Ca_GpSSR01796	scaffold03053	Dinucleotide	(TA)7	14	2456	2469	scaffold845	Dinucleotide	(TA)8	16	1277	1292	Intergenic		TTGGGTATGAGTCTAATTGTCA	GCGACTTGACTCGATCTATTA
Ca_GpSSR01797	scaffold00776	Dinucleotide	(TA)6	12	71061	71072	scaffold845	Dinucleotide	(TA)7	14	152652	152665	gene=Ca_13996;mRNA=Ca_13996.1;intron;		TTTGCGAGTACTCATTGGATA	GCGACTTGACTCGATCTATTA
Ca_GpSSR01798	scaffold00776	Dinucleotide	(TA)9	18	48198	48215	scaffold845	Dinucleotide	(TA)13	26	177019	177044	Intergenic		AATTAGATTTCAACCCACACA	CTCGTTTATCAATCACTGTCG
Ca_GpSSR01799	scaffold33320	Dinucleotide	(TA)17	34	1	34	scaffold845	Dinucleotide	(TA)11	22	12342	12363	Intergenic		CGCTGCGTATTATTTGTTATT	TCTCCTCATTGAATGTGAACT
Ca_GpSSR01800	scaffold03254	Trinucleotide	(TAA)52	156	31324	31479	scaffold845	Trinucleotide	(TAA)20	60	312256	312315	Intergenic			
Ca_GpSSR01801	scaffold56091	Trinucleotide	(TTA)44	132	1	132	scaffold845	Trinucleotide	(TTA)12	36	367845	367880	Intergenic			
Ca_GpSSR01802	scaffold23028	Dinucleotide	(TA)15	30	39	68	scaffold845	Dinucleotide	(TA)12	24	368715	368738	Intergenic		CGCTGCGTATTATTTGTTATT	TCTCCTCATTGAATGTGAACT
Ca_GpSSR01803	scaffold04926	Dinucleotide	(AT)25	50	6871	6920	scaffold845	Dinucleotide	(AT)9	18	378553	378570	Intergenic		CGCTGCGTATTATTTGTTATT	TCTCCTCATTGAATGTGAACT
Ca_GpSSR01804	scaffold11629	Dinucleotide	(AT)16	32	97	128	scaffold866	Dinucleotide	(AT)12	24	38306	38329	Intergenic		TTGTGTGTTGCTATGTGTGAT	CTTTATCGAAGGCACTCATTA
Ca_GpSSR01805	scaffold04497	Dinucleotide	(AT)21	42	18734	18775	scaffold876	Dinucleotide	(AT)30	60	595319	595378	Intergenic		GTGATATTGTGTTTGATTTCCTT	CTTGTGTTTCCAAATACTCAAA
Ca_GpSSR01806	scaffold02298	Dinucleotide	(TA)19	38	2746	2783	scaffold87	Dinucleotide	(TA)11	22	581533	581554	Intergenic		TTCTATTTCCTTTTCCTTTCC	TATGCCTTATGAGTGAAATGG
Ca_GpSSR01807	scaffold02044	Dinucleotide	(AT)13	26	30708	30733	scaffold87	Dinucleotide	(AT)11	22	110621	110642	Intergenic		TTTCCTCTTAGGTACGCCTAT	TTGTGTGAGGTCAACTAATTT
Ca_GpSSR01808	scaffold49409	Dinucleotide	(AT)26	52	1	52	scaffold87	Dinucleotide	(AT)24	48	349983	350030	Intergenic		AAGGAAAAGTGAAAAGGAATG	AAGTGTTGTGTGTGAGAGTGA
Ca_GpSSR01809	scaffold04561	Dinucleotide	(AT)9	18	3941	3958	scaffold87	Dinucleotide	(AT)8	16	377128	377143	gene=Ca_18999;mRNA=Ca_18999.1;intron;		CGCTAGAGCTAGAAGACATTG	TGGTTCATAACACTAACATAACA
Ca_GpSSR01810	scaffold03572	Dinucleotide	(AT)11	22	4025	4046	scaffold87	Dinucleotide	(AT)12	24	451514	451537	Intergenic		TTCTATTTCCTTTTCCTTTCC	TATGCCTTATGAGTGAAATGG
Ca_GpSSR01811	scaffold07439	Dinucleotide	(AT)11	22	563	584	scaffold882	Dinucleotide	(AT)14	28	103091	103118	Intergenic		TGGAATATGAGAATTTTGAG	ATTGGATTTTAGTTGGAGTT
Ca_GpSSR01812	scaffold00519	Dinucleotide	(TA)18	36	96049	96084	scaffold882	Dinucleotide	(TA)15	30	135979	136008	Intergenic		CGGGTGTCTTGAGATTTATTT	TGATTGCATTTATGCTACTAAGA
Ca_GpSSR01813	scaffold02905	Dinucleotide	(TA)18	36	19758	19793	scaffold882	Dinucleotide	(TA)28	56	483532	483587	Intergenic		CTTTCTTGGTTCCTTTTGAAT	ACAACATTAGTTATTGTCTTGG
Ca_GpSSR01814	scaffold36935	Dinucleotide	(AT)11	22	1	22	scaffold882	Dinucleotide	(AT)30	60	497112	497171	Intergenic		CGAAGATCCAAAGGTTAAAAA	TCAATGCCATGTCAATAAT
Ca_GpSSR01815	scaffold00592	Trinucleotide	(TTA)7	21	70266	70286	scaffold88	Trinucleotide	(TTA)6	18	47775	47792	gene=Ca_13316;mRNA=Ca_13316.1;exon=Ca_13316.		TTAGGATGCATGCTTTAATCT	CTGTGTGAATATTTGGTATTACT
Ca_GpSSR01816	scaffold00592	Dinucleotide	(AT)13	26	120041	120066	scaffold88	Dinucleotide	(AT)10	20	102499	102518	Intergenic		AGTGAAGGCGTGTTTTTATTT	TCAAGACCGTTCAGACATTAT
Ca_GpSSR01817	scaffold04106	Dinucleotide	(AT)6	12	22418	22429	scaffold88	Dinucleotide	(AT)21	42	156039	156080	upstream:Ca_18781;		AGTGAAGGCGTGTTTTTATTT	TCAAGACCGTTCAGACATTAT
Ca_GpSSR01818	scaffold01197	Trinucleotide	(TTA)31	93	59282	59374	scaffold913	Trinucleotide	(TTA)34	102	194518	194619	Intergenic			
Ca_GpSSR01819	scaffold01197	Dinucleotide	(AT)12	24	77182	77205	scaffold913	Dinucleotide	(AT)16	32	212650	212681	Intergenic		ACTACATCTCTTTTGGGGAGA	AATTAATGGACCGCATATGTA
Ca_GpSSR01820	scaffold01046	Dinucleotide	(AT)6	12	23363	23374	scaffold913	Dinucleotide	(AT)14	28	346362	346389	Intergenic		ACTACATCTCTTTTGGGGAGA	AATTAATGGACCGCATATGTA
Ca_GpSSR01821	scaffold00985	Dinucleotide	(AT)6	12	62637	62648	scaffold914	Dinucleotide	(AT)9	18	127061	127078	Intergenic		CAATGAACTCGAAGGAGTGTA	AATAAAACGTGTGTGTGTGTG
Ca_GpSSR01822	scaffold00985	Dinucleotide	(AT)12	24	100597	100620	scaffold914	Dinucleotide	(AT)10	20	152007	152026	Intergenic		GTCTATGGCGTTGATTATGAC	AGATCTGACGAAACAAATTGA
Ca_GpSSR01823	scaffold03914	Trinucleotide	(ATA)6	18	4821	4838	scaffold916	Trinucleotide	(ATA)5	15	139777	139791	Intergenic		GATCTCCAACAAAACTGTCAA	TGATGTCATATACCACATAACG
Ca_GpSSR01824	scaffold00415	Dinucleotide	(TA)11	22	234871	234892	scaffold916	Dinucleotide	(TA)10	20	282553	282572	Intergenic		CATTATGGGTGAAATGGTAAT	TCTCTTCTTCTTCTTCTTTTCC
Ca_GpSSR01825	scaffold00415	Dinucleotide	(AT)8	16	46529	46544	scaffold916	Dinucleotide	(AT)7	14	444844	444857	upstream:Ca_09196;		GAGTGGAACCAATAAAATTGA	AATAATTCTCAATCCAAAGC
Ca_GpSSR01826	scaffold00415	Dinucleotide	(AT)12	24	25371	25394	scaffold916	Dinucleotide	(AT)11	22	466016	466037	mRNA=Ca_09195.1;gene=Ca_09195;intron;		CATTATGGGTGAAATGGTAAT	TCTCTTCTTCTTCTTCTTTTCC
Ca_GpSSR01827	scaffold01401	Trinucleotide	(TTA)11	33	48251	48283	scaffold931	Trinucleotide	(TTA)10	30	37674	37703	Intergenic			
Ca_GpSSR01828	scaffold70197	Dinucleotide	(TA)20	40	482	521	scaffold9367	Dinucleotide	(TA)6	12	212	223	Intergenic		TGCAGCGGATAAATACAAATA	CGCTGCAAATTTTTAAGG
Ca_GpSSR01829	scaffold02056	Dinucleotide	(TA)8	16	20435	20450	scaffold953	Dinucleotide	(TA)10	20	138251	138270	Intergenic		AAGACCATGGAGCTTATTTTC	CAAAGTGGCTGAATACTTTGT
Ca_GpSSR01830	scaffold02242	Trinucleotide	(TTA)13	39	47426	47464	scaffold959	Trinucleotide	(TTA)14	42	52509	52550	Intergenic		GTCATTTACAGGCTAAAAAGAAA	TAGTGTGCGCAAACAATAAA
Ca_GpSSR01831	scaffold02950	Trinucleotide	(TAA)7	21	35723	35743	scaffold962	Trinucleotide	(TAA)8	24	202261	202284	Intergenic		TATATGTAAAACCCATTTGC	CGAAGAAGAAGAAGAAGAAGG
Ca_GpSSR01832	scaffold01364	Trinucleotide	(TAA)8	24	2986	3009	scaffold964	Trinucleotide	(TAA)16	48	81360	81407	Intergenic			
Ca_GpSSR01833	scaffold02918	Dinucleotide	(TA)24	48	14473	14520	scaffold98	Dinucleotide	(TA)16	32	444203	444234	Intergenic		CCGACAAATAACTTTTATCGAC	TGCAGTTTTAGGATATTTTGTG
Ca_GpSSR01834	scaffold00508	Dinucleotide	(TA)7	14	9296	9309	scaffold996	Dinucleotide	(TA)8	16	37799	37814	Intergenic		TGATTTTTAACGCTGTTTGA	TAATACCAGAACCCAGGATTT
Ca_GpSSR01835	scaffold13150	Trinucleotide	(TAT)16	48	3397	3444	scaffold9	Trinucleotide	(TAT)34	102	80354	80455	Intergenic			
