# Supplementary material for: Development of genome-wide informative simple sequence repeat markers for large-scale genotyping applications in chickpea and development of web resource
Source: Front Plant Sci. 2015 Aug 21;6:645. doi: 10.3389/fpls.2015.00645 (PMC4543896; doi:10.3389/fpls.2015.00645)
Supplement: Supplementary file 2 [file Table_2.PDF]

Supplementary Table S2. List of 341 polymorphic simple sequence repeats validated by polymerase chain reaction.

SSR ID	*Desi*				*Kabuli*				Annotation	Forward primer	Reverse primer	Monomorphic (M)/ Polymorphic (P)	Number of alleles amplified	Polymorphism information content (PIC)
	Linkage group/Scaffold ID	SSR motif	Start position (bp)	Stop position (bp)	Linkage group/Scaffold ID	SSR motif	Start position (bp)	Stop position (bp)						
Ca_GpSSR00009	Ca_LG_1	(AT)16	1907876	1907907	Ca1	(AT)10	1086807	1086826	Intron	TGAATGGAGTAAATGATTACTTG	TCGCATATAGACGGATAATTT	N	N	0.00
Ca_GpSSR00010	Ca_LG_1	(AG)7	2590893	2590906	Ca1	(AG)9	2129374	2129391	exon, 5'-UTR	ATAGTGGACCCCACAATCCA	TCGCTGTTGTGTTTTTGCTT	M	1	0.00
Ca_GpSSR00015	Ca_LG_1	(TA)23	3163612	3163657	Ca1	(TA)14	3286600	3286627		ATCGCGTTAAATACTTGGACT	CATATGTGGAATGGACTATGC	M	1	0.00
Ca_GpSSR00017	Ca_LG_1	(TA)10	3509348	3509367	Ca1	(TA)6	3677591	3677602	Intron	ATCGCGTTAAATACTTGGACT	CATATGTGGAATGGACTATGC	M	1	0.00
Ca_GpSSR00028	Ca_LG_1	(AT)19	5529971	5530008	Ca1	(AT)9	6514446	6514463		ATCGCGTTAAATACTTGGACT	CATATGTGGAATGGACTATGC	P	3	0.65
Ca_GpSSR00038	Ca_LG_1	(AT)11	2813153	2813174	Ca1	(AT)25	8377748	8377797		CTGTTACGTGCAATGGATGC	TCGGTATGACACAAAAATGTGA	P	6	0.78
Ca_GpSSR00039	Ca_LG_1	(TA)16	2981371	2981402	Ca1	(TA)6	8556463	8556474		TGAATGGAGTAAATGATTACTTG	TCGCATATAGACGGATAATTT	N	N	0.00
Ca_GpSSR00045	Ca_LG_1	(AT)13	12006995	12007020	Ca1	(AT)10	12092744	12092763		ATCGCGTTAAATACTTGGACT	CATATGTGGAATGGACTATGC	P	4	0.84
Ca_GpSSR00081	Ca_LG_1	(AT)11	6783655	6783676	Ca1	(AT)16	17729267	17729298		TCATGCAACAGACGAATTGA	AACATGTTATAAAACGTAAGGCG	P	5	0.77
Ca_GpSSR00114	Ca_LG_1	(TTA)25	7150246	7150320	Ca1	(TTA)12	22326881	22326916		TGTGACAAAaCAAaTAACGTGTCA	CGTCAAAGTTAACGCAGTTGAG	P	4	0.82
Ca_GpSSR00173	Ca_LG_1	(AT)16	9787142	9787173	Ca1	(AT)22	47415169	47415212		tGtCAcAAaCATGTAaGaCAGTtGTT	AAGATTTGGCTGGCTTCATC	P	7	0.80
Ca_GpSSR00002	Ca_LG_1	(AT)14	718312	718339	Ca1	(AT)12	623176	623199	Upstream	ATCGCGTTAAATACTTGGACT	CATATGTGGAATGGACTATGC	P	2	0.70
Ca_GpSSR00056	Ca_LG_1	(AT)8	10223659	10223674	Ca1	(AT)7	15037302	15037315	Upstream	ATCGCGTTAAATACTTGGACT	CATATGTGGAATGGACTATGC	P	2	0.72
Ca_GpSSR00040	Ca_LG_1	(AT)10	13639938	13639957	Ca1	(AT)13	9758418	9758443	Upstream	ATCGCGTTAAATACTTGGACT	CATATGTGGAATGGACTATGC	P	2	0.65
Ca_GpSSR00003	Ca_LG_1	(AT)17	728566	728599	Ca1	(AT)22	633426	633469		TGAATGGAGTAAATGATTACTTG	TCGCATATAGACGGATAATTT	P	3	0.63
Ca_GpSSR00008	Ca_LG_1	(ATA)11	1757091	1757123	Ca1	(ATA)20	939741	939800	Upstream	TGACCAACTTTCCTTTTAAGAT	ATTGGAAAATTCTGGTAAAGC	N	N	0.00
Ca_GpSSR00479	Ca_LG_1	(AT)13	5252128	5252153	Ca3	(AT)7	35371166	35371179		CAAATATGCAATAACCTTCCA	AAAAATCTATTTGGTGCTGGT	N	N	0.00
Ca_GpSSR00050	Ca_LG_1	(TA)8	11064897	11064912	Ca1	(TA)14	13417844	13417871		ATCGCGTTAAATACTTGGACT	CATATGTGGAATGGACTATGC	M	1	0.00
Ca_GpSSR00155	Ca_LG_1	(TA)7	9009356	9009369	Ca1	(TA)19	42003292	42003329		AAAATTTCAATTCACGGTTG	CTTGCCAATTTATCATGTTTG	M	1	0.00
Ca_GpSSR00224	Ca_LG_2	(TA)12	2464854	2464877	Ca2	(TA)9	9921390	9921407		TTTCTTCCTCATTTCAACAA	GAGTGACGTATTTTCTCAACTG	M	1	0.00
Ca_GpSSR00227	Ca_LG_2	(TA)10	2550179	2550198	Ca2	(TA)7	10013936	10013949		ATTTGCACTCTTTGACTTTGA	TCAAATGTTTAACTCGTGGAC	P	3	0.70
Ca_GpSSR00231	Ca_LG_2	(AT)7	5419221	5419234	Ca2	(AT)11	10240969	10240990		ATCTGGGGCCAAAAaGTAGG	CATTCAGCGACAAAAGGTCA	P	3	0.68
Ca_GpSSR00272	Ca_LG_2	(AT)13	3515543	3515568	Ca2	(AT)17	28157708	28157741		TcATTTTTGAATGTCCATGCTT	TCAATTTCTTAtACTTTTCGCAACA	M	1	0.00
Ca_GpSSR00280	Ca_LG_2	(TA)15	3679983	3680012	Ca2	(TA)12	30291446	30291469		TTTCTTCCTCATTTCAACAA	GAGTGACGTATTTTCTCAACTG	M	1	0.00
Ca_GpSSR00296	Ca_LG_2	(TTA)5	6529337	6529351	Ca2	(TTA)13	32478386	32478424		TGATGTTCTCTGTCTTTCCTT	TCCATATGCAAGAGTAAAAGC	P	4	0.83
Ca_GpSSR00318	Ca_LG_2	(GTT)5	16696532	16696546	Ca2	(GTT)7	35998700	35998720	exon, CDS	TGGCCCAATATTCAGATTCC	TCACCAAACCAGGTACTGCTC	P	6	0.72
Ca_GpSSR01239	Ca_LG_2	(TAT)26	13223880	13223957	Ca6	(TAT)17	49337554	49337604		GACGATTCTCCATAACTGTGA	ACAAAGGTTGTGTTTCCTAAA	P	5	0.78
Ca_GpSSR01278	Ca_LG_2	(TA)15	777203	777232	Ca6	(TA)25	56537564	56537613	Intron	TCAATTCGAACAAAGTAACGGA	TTGGACCAAATTAGGAATATCCA	N	N	0.00
Ca_GpSSR01283	Ca_LG_2	(ATT)34	5996147	5996248	Ca6	(ATT)13	57610529	57610567		AAGTTGTGCAACAAATAGGAA	ATACCGAACAACAACAACAAC	N	N	0.00
Ca_GpSSR01657	Ca_LG_2	(AG)13	14766404	14766429	scaffold2330	(AG)17	65266	65299	Intron	GACAAGCCTCCGTGTGATTT	ATCGACGTCGCTAAATGCTT	P	7	0.82
Ca_GpSSR01242	Ca_LG_2	(TA)10	13448318	13448337	Ca6	(TA)11	49593059	49593080	Upstream	ATTTGCACTCTTTGACTTTGA	TCAAATGTTTAACTCGTGGAC	P	3	0.80
Ca_GpSSR01116	Ca_LG_2	(AT)11	14452258	14452279	Ca6	(AT)18	19439669	19439704	Upstream	ATTTGCACTCTTTGACTTTGA	TCAAATGTTTAACTCGTGGAC	P	3	0.81
Ca_GpSSR01289	Ca_LG_2	(GA)8	5603850	5603865	Ca6	(GA)7	57995975	57995988	Intron	AGCGAAATGTATTTCAGTGAG	CTTTCATTCTTCTCTCCCATT	P	4	0.80
Ca_GpSSR00297	Ca_LG_2	(TC)8	6872427	6872442	Ca2	(TC)9	32745344	32745361	Intron	TCTGGCAGATGAAGATGTAAC	TAACAGACCAAAAACTCCAAA	P	2	0.69
Ca_GpSSR00128	Ca_LG_2	(AT)18	5284151	5284186	Ca1	(AT)12	26624005	26624028		ATCGCGTTAAATACTTGGACT	CATATGTGGAATGGACTATGC	P	2	0.55
Ca_GpSSR00195	Ca_LG_2	(AT)19	1975086	1975123	Ca2	(AT)28	4014917	4014972		ACAATTCCACTTAATCTTTGC	AATTAGCCTACAGACACACACA	P	3	0.79
Ca_GpSSR01240	Ca_LG_2	(AT)17	13345118	13345151	Ca6	(AT)27	49485417	49485470		AGCACTTGTTAACGTTTTCTT	GTCAATCTATTGTGATGTTTTTG	P	2	0.58
Ca_GpSSR01156	Ca_LG_2	(AT)11	8611652	8611673	Ca6	(AT)25	27273612	27273661		ATTTGCACTCTTTGACTTTGA	TCAAATGTTTAACTCGTGGAC	P	2	0.55
Ca_GpSSR01194	Ca_LG_2	(TA)11	10367024	10367045	Ca6	(TA)29	35049925	35049982		ATTTGCACTCTTTGACTTTGA	TCAAATGTTTAACTCGTGGAC	P	3	0.77
Ca_GpSSR00228	Ca_LG_2	(AT)6	2573258	2573269	Ca2	(AT)7	10032702	10032715	Upstream	ATTTGCACTCTTTGACTTTGA	TCAAATGTTTAACTCGTGGAC	N	N	0.00
Ca_GpSSR01287	Ca_LG_2	(TA)7	5557641	5557654	Ca6	(TA)13	57952561	57952586	Upstream	ATTTGCACTCTTTGACTTTGA	TCAAATGTTTAACTCGTGGAC	N	N	0.00
Ca_GpSSR00295	Ca_LG_2	(AT)6	6418959	6418970	Ca2	(AT)9	32334667	32334684	Upstream	ATTTGCACTCTTTGACTTTGA	TCAAATGTTTAACTCGTGGAC	N	N	0.00
Ca_GpSSR00303	Ca_LG_2	(AT)16	7642719	7642750	Ca2	(AT)14	34235262	34235289	Upstream	TTTCTTCCTCATTTCAACAA	GAGTGACGTATTTTCTCAACTG	N	N	0.00
Ca_GpSSR01268	Ca_LG_2	(AT)8	9839053	9839068	Ca6	(AT)9	54202310	54202327	exon, 3'-UTR	ATTTGCACTCTTTGACTTTGA	TCAAATGTTTAACTCGTGGAC	N	N	0.00
Ca_GpSSR01298	Ca_LG_2	(AT)9	204946	204963	Ca6	(AT)7	59222237	59222250	Upstream	ATTTGCACTCTTTGACTTTGA	TCAAATGTTTAACTCGTGGAC	M	1	0.00
Ca_GpSSR00158	Ca_LG_2	(TA)20	132909	132948	Ca1	(TA)9	43101961	43101978		ATCGCGTTAAATACTTGGACT	CATATGTGGAATGGACTATGC	M	1	0.00
Ca_GpSSR01143	Ca_LG_2	(AT)9	13185166	13185183	Ca6	(AT)18	23107468	23107503		ATTTGCACTCTTTGACTTTGA	TCAAATGTTTAACTCGTGGAC	M	1	0.00
Ca_GpSSR00229	Ca_LG_2	(TA)9	2626750	2626767	Ca2	(TA)22	10086460	10086503		ATTTGCACTCTTTGACTTTGA	TCAAATGTTTAACTCGTGGAC	M	1	0.00
Ca_GpSSR00381	Ca_LG_3	(AT)13	12984678	12984703	Ca3	(AT)9	16563840	16563857	Intron	TGCAATAGTACAAAatAaTTGTAaTCc	tTTGTTTGtTTGACACATTGCT	N	N	0.00
Ca_GpSSR00407	Ca_LG_3	(TA)13	12053446	12053471	Ca3	(TA)21	21125541	21125582		GAGCACCAATTAAAGAGGGC	GGCATTTAAAACATAAATCCCAA	P	7	0.74
Ca_GpSSR00419	Ca_LG_3	(ATA)7	6257604	6257624	Ca3	(ATA)5	22850419	22850433		TCATCTCCTGCTCCAGTATTA	TTGTTGAGGTCACTCACTTCT	M	1	0.00
Ca_GpSSR00437	Ca_LG_3	(AT)16	9885008	9885039	Ca3	(AT)6	26388749	26388760		TGATTTAATTATCGATGTGAGTTTCAA	TTGAGTTAATGAAACAAATAAGTCGAA	M	1	0.00
Ca_GpSSR00451	Ca_LG_3	(AT)15	7054559	7054588	Ca3	(AT)9	30841155	30841172		CAAATATGCAATAACCTTCCA	AAAAATCTATTTGGTGCTGGT	N	N	0.00
Ca_GpSSR00458	Ca_LG_3	(TA)9	6598132	6598149	Ca3	(TA)14	31474732	31474759		TCAAAGGCAATTTTGATTGAA	TTTTTGATAGTCGCAGGAcCTT	P	3	0.68
Ca_GpSSR00481	Ca_LG_3	(TTA)25	3805284	3805358	Ca3	(TTA)33	35840378	35840476		TtAattttgTTTTTGttCTTtcgTt	GAGAAGGAGGCTGAGCAAAA	N	N	0.00
Ca_GpSSR00483	Ca_LG_3	(AT)8	3907503	3907518	Ca3	(AT)11	35943336	35943357		CGAACACTGATGCCAAACAA	AAAATGTATTTTATGAGTTTGGCCTAT	P	4	0.65
Ca_GpSSR00487	Ca_LG_3	(AG)8	4194481	4194496	Ca3	(AG)6	36225055	36225066	exon, 5'-UTR	TGAAATTCAGCAACGAAATGA	cCCAGTAACCGCTCTCTCTG	P	4	0.63
Ca_GpSSR00488	Ca_LG_3	(TA)9	4217827	4217844	Ca3	(TA)12	36248881	36248904	exon, 3'-UTR	GCAGGGgcTATATGGATTTG	GCATGAATTCAGCAGAAGAAAA	P	4	0.23
Ca_GpSSR00499	Ca_LG_3	(AT)15	3313388	3313417	Ca3	(AT)11	38406108	38406129		TTTTtCTCCCTTGACTAATTGATTTT	TGGTTGATTTGGTAACATTTTTCA	M	1	0.00
Ca_GpSSR00500	Ca_LG_3	(AT)19	2692579	2692616	Ca3	(AT)16	38784891	38784922		CAAATATGCAATAACCTTCCA	AAAAATCTATTTGGTGCTGGT	N	N	0.00
Ca_GpSSR00568	Ca_LG_3	(TAT)58	3027986	3028159	Ca4	(TAT)18	11669951	11670004		CATTCCTTCAGCATTTCTTTT	CGTAGAATTTACCGTTGTTCT	P	10	0.79
Ca_GpSSR01493	Ca_LG_3	(ATT)29	16428093	16428179	Ca8	(ATT)23	3267938	3268006		TCACAGGTCCAATCATATTTAATCA	AAAAGaAATCTTAATTGAGGACAACA	N	N	0.00
Ca_GpSSR01503	Ca_LG_3	(AAT)10	18287640	18287669	Ca8	(AAT)7	4186335	4186355		AATCTGACCATAAGTTGACGA	AGAACTATTACTGCATACCTCCA	P	10	0.85
Ca_GpSSR01505	Ca_LG_3	(TA)9	18423316	18423333	Ca8	(TA)18	4329872	4329907		GGGAGGGATGGAACCTTTTA	TCATTTGTGTGGTTGCTCTTG	P	6	0.71
Ca_GpSSR01487	Ca_LG_3	(TA)10	20175269	20175288	Ca8	(TA)18	1402760	1402795	Upstream	AATCCACAAACACAACAAAAC	CCAATCAACTCATTGAGACC	P	3	0.81
Ca_GpSSR01555	Ca_LG_3	(AT)10	22861426	22861445	Ca8	(AT)9	15722416	15722433	Upstream	AATCCACAAACACAACAAAAC	CCAATCAACTCATTGAGACC	P	2	0.65
Ca_GpSSR01481	Ca_LG_3	(TA)12	19729380	19729403	Ca8	(TA)13	728619	728644	Upstream	ACCTCGATAAACAGTACAGACC	TGAATTTGAGTGTTAGCGTTT	P	3	0.78
Ca_GpSSR01504	Ca_LG_3	(TA)19	18380371	18380408	Ca8	(TA)27	4281630	4281683	Upstream	AGCCCCTCTTACTCTCACATA	TTGTATGTTTGCTCCGTGTAT	P	5	0.86
Ca_GpSSR00477	Ca_LG_3	(TA)18	4824557	4824592	Ca3	(TA)21	35051582	35051623	Upstream	CAAATATGCAATAACCTTCCA	AAAAATCTATTTGGTGCTGGT	P	3	0.83
Ca_GpSSR01758	Ca_LG_3	(AT)16	18580721	18580752	scaffold528	(AT)25	238824	238873	Upstream	GCATGTTGATTTTGATAAGGA	CTTCTTCTGATGCCATGACT	P	2	0.69
Ca_GpSSR01488	Ca_LG_3	(TA)17	17869439	17869472	Ca8	(TA)28	2055758	2055813	Intron	AGCCCCTCTTACTCTCACATA	TTGTATGTTTGCTCCGTGTAT	P	2	0.54
Ca_GpSSR00459	Ca_LG_3	(TA)12	6355894	6355917	Ca3	(TA)11	31540732	31540753	Intron	CAAATATGCAATAACCTTCCA	AAAAATCTATTTGGTGCTGGT	P	4	0.79
Ca_GpSSR00343	Ca_LG_3	(AT)23	14475055	14475100	Ca3	(AT)28	5431014	5431069		AATTTACCGCTGAAGAGTTTC	AGGGTAAACTTTAAACTCAAATG	P	4	0.81
Ca_GpSSR01543	Ca_LG_3	(AT)10	1581794	1581813	Ca8	(AT)17	13899613	13899646		AATCCACAAACACAACAAAAC	CCAATCAACTCATTGAGACC	P	4	0.80
Ca_GpSSR01498	Ca_LG_3	(AT)12	15917112	15917135	Ca8	(AT)31	3361828	3361889		AGCCCCTCTTACTCTCACATA	TTGTATGTTTGCTCCGTGTAT	P	3	0.78
Ca_GpSSR01517	Ca_LG_3	(TC)6	19075098	19075109	Ca8	(TC)7	5831790	5831803	Upstream	CTTAAGGTTTGGTGGAATTG	CCCTTTGTGGAATAAGTTGAT	N	N	0.00
Ca_GpSSR00428	Ca_LG_3	(AAT)20	11236544	11236603	Ca3	(AAT)21	24112136	24112198	Upstream	TTAAGCTCAAACAAACGAATC	ACCTTTTTAGACAATAGATGTCA	N	N	0.00
Ca_GpSSR01490	Ca_LG_3	(AT)21	14980351	14980392	Ca8	(AT)13	2325799	2325824		ACCTCGATAAACAGTACAGACC	TGAATTTGAGTGTTAGCGTTT	N	N	0.00
Ca_GpSSR00330	Ca_LG_3	(AT)10	14009997	14010016	Ca3	(AT)15	3013495	3013524		AATCCACAAACACAACAAAAC	CCAATCAACTCATTGAGACC	N	N	0.00
Ca_GpSSR00460	Ca_LG_3	(AT)8	6378639	6378654	Ca3	(AT)13	31576728	31576753		AATCCACAAACACAACAAAAC	CCAATCAACTCATTGAGACC	N	N	0.00
Ca_GpSSR00445	Ca_LG_3	(TA)13	8546278	8546303	Ca3	(TA)14	28932041	28932068	Upstream	CAAATATGCAATAACCTTCCA	AAAAATCTATTTGGTGCTGGT	M	1	0.00
Ca_GpSSR01501	Ca_LG_3	(TC)7	16959668	16959681	Ca8	(TC)8	3903450	3903465	Upstream	TGATCCTTCAATCAAGTTTTG	TCTCTCTCTGTAAATCCGTTT	M	1	0.00
Ca_GpSSR00489	Ca_LG_3	(CT)8	4298387	4298402	Ca3	(CT)9	36329058	36329075	Intron	CAACTCTCAACTCCCTAATCC	AGTGAGTGAGGTAGGGAGAAA	M	1	0.00
Ca_GpSSR00453	Ca_LG_3	(TA)8	6953286	6953301	Ca3	(TA)18	30941968	30942003		AATCCACAAACACAACAAAAC	CCAATCAACTCATTGAGACC	M	1	0.00
Ca_GpSSR01483	Ca_LG_3	(TA)16	19760649	19760680	Ca8	(TA)26	759587	759638		TCAAGAACATTTCTCAAACAGT	ACTTTTTCCATCTCTTTCGTT	M	1	0.00
Ca_GpSSR00504	Ca_LG_4	(ATA)21	18982147	18982209	Ca4	(ATA)13	1104792	1104830		TCACTTTTACATTACACAcAAGGATt	CATGTCTTTTGATATACTACCTCCACA	P	4	0.80
Ca_GpSSR00521	Ca_LG_4	(TA)10	16984989	16985008	Ca4	(TA)16	4062811	4062842	Intron	AATGACGCAGGTTCAACACA	ATGTCATTATGTTGCATTAAGATCTAC	P	6	0.83
Ca_GpSSR00527	Ca_LG_4	(AT)11	16754699	16754720	Ca4	(AT)23	4295796	4295841		TCTTCACGCTCCCTACAAAA	TGAGCGTTATAGGGGAGAAAAA	P	6	0.75
Ca_GpSSR00529	Ca_LG_4	(TAA)5	20448344	20448358	Ca4	(TAA)14	4516085	4516126		CTACGTTTCACAATATTTTCGTT	ACCCCTTTTCTCTTTCTTCTA	P	2	0.82
Ca_GpSSR00534	Ca_LG_4	(AT)9	20533227	20533244	Ca4	(AT)6	4594656	4594667	exon, 5'-UTR	TGATCGATTGCTCCTCCTTT	ACCCATCATCCCCACTCATA	M	1	0.00
Ca_GpSSR00536	Ca_LG_4	(AG)9	20788615	20788632	Ca4	(AG)12	4842166	4842189	exon, 5'-UTR	TTATCCCGTAAGCCTCGCTA	CCACGTCGGTTCTCTTTGAT	P	5	0.59
Ca_GpSSR00578	Ca_LG_4	(ATA)12	10552513	10552548	Ca4	(ATA)18	12681613	12681666		CCAAGCTATAGTGCAATCATCGT	AGGAGGCCTTTGAAAAATCA	P	7	0.76
Ca_GpSSR00603	Ca_LG_4	(AT)10	7748875	7748894	Ca4	(AT)26	17341313	17341364		AAAACATCATGTGCTGGTGC	CAGCTCATCCTCCTACTGTGG	M	1	0.00
Ca_GpSSR00604	Ca_LG_4	(TA)6	7681073	7681084	Ca4	(TA)10	17395507	17395526	Intron	tCAAAGGTTGAGAACGAGTCC	CAACTTTCATTTTCTCACGAAGA	N	N	0.00
Ca_GpSSR00641	Ca_LG_4	(TAA)37	5895990	5896100	Ca4	(TAA)6	27313204	27313221		TAAATCAATCTACACCGCAAT	ATATGGGACAAGATGATGAGA	M	1	0.00
Ca_GpSSR00648	Ca_LG_4	(AT)13	5673428	5673453	Ca4	(AT)25	28087405	28087454		TTTGCATTGAGATTCATTGTTTTT	AGCGAGGATCGAGACTAAACa	N	N	0.00
Ca_GpSSR00715	Ca_LG_4	(TA)16	4607604	4607635	Ca4	(TA)11	38674437	38674458	Intron	CACCAAGCCACCcTTATGAC	TGGGATGACTGCTATTCCAA	N	N	0.00
Ca_GpSSR00728	Ca_LG_4	(AT)12	3824144	3824167	Ca4	(AT)21	40760314	40760355		TgCCcTTTtAaGgTTtGTTTG	TGATTTCTCATTTTAAACGGtTTTT	P	4	0.74
Ca_GpSSR00729	Ca_LG_4	(AT)19	3825049	3825086	Ca4	(AT)11	40761337	40761358		AGAAAATCAGAAGTTGCATT	AAAGTTTGGTGGTGAGTTTTT	N	N	0.00
Ca_GpSSR00742	Ca_LG_4	(TA)15	1057355	1057384	Ca4	(TA)11	44015358	44015379		AGAAAATCAGAAGTTGCATT	AAAGTTTGGTGGTGAGTTTTT	N	N	0.00
Ca_GpSSR01634	Ca_LG_4	(AC)18	6945890	6945925	scaffold1943	(AC)7	354278	354291		TTAGTGAAGCGATGGGTATAA	CTCTTCCTTTCTCAGTTTTCC	M	1	0.00
Ca_GpSSR00736	Ca_LG_4	(AT)12	3370477	3370500	Ca4	(AT)11	41748174	41748195	Upstream	AGAAAATCAGAAGTTGCATT	AAAGTTTGGTGGTGAGTTTTT	P	4	0.85
Ca_GpSSR00543	Ca_LG_4	(TA)22	16002374	16002417	Ca4	(TA)20	5655210	5655249	Upstream	ATCATTTCCTTCGGTTCATC	TTCTTTTCTTCCTAAAAGGACA	P	3	0.80
Ca_GpSSR00741	Ca_LG_4	(AT)17	1024068	1024101	Ca4	(AT)18	43981124	43981159	Upstream	GCCTAAAATCATAAAACCAAAG	ATGCAAGGGTTATTTTATGTC	P	4	0.80
Ca_GpSSR00538	Ca_LG_4	(ATT)7	20834957	20834977	Ca4	(ATT)6	4891738	4891755	Intron	AAAATAAAAGCTAACGGTCA	TCGAAGTTGTGTCAAACTGTA	P	2	0.71
Ca_GpSSR00552	Ca_LG_4	(TC)10	12035781	12035800	Ca4	(TC)11	9736708	9736729	Intron	ACCATTTATGAATTACGGTCA	CACACACACATTCACTCTCAA	P	2	0.66
Ca_GpSSR00716	Ca_LG_4	(AT)10	4611897	4611916	Ca4	(AT)8	38678558	38678573	Intron	AGAAAATCAGAAGTTGCATT	AAAGTTTGGTGGTGAGTTTTT	P	3	0.69

Ca GpSSR00556	Ca LG 4	(TA)9	12332683	12332700	Ca4	(TA)7	10047177	10047190	Intron	AGAAAATCAGAAGTTGCATT	AAAGTTTGGTGGTGAGTTTTT	P	3	0.70
Ca GpSSR00537	Ca LG 4	(TA)10	20822750	20822769	Ca4	(TA)8	4874271	4874286	Intron	AGAAAATCAGAAGTTGCATT	AAAGTTTGGTGGTGAGTTTTT	P	2	0.51
Ca GpSSR00535	Ca LG 4	(AAT)40	20535959	20536078	Ca4	(AAT)18	4597377	4597430		AAAATGGTAGGTCATTCATTACT	TACTCTTTCCGCCACAATAA	P	3	0.66
Ca GpSSR00512	Ca LG 4	(TA)25	18464251	18464300	Ca4	(TA)8	1962951	1962966		TTAAAGACCCTTTTTGGAATC	TCAATCTTTTACTCTCGCAAT	P	3	0.64
Ca GpSSR00593	Ca LG 4	(AT)17	8874342	8874375	Ca4	(AT)6	15924759	15924770		AGAAAATCAGAAGTTGCATT	AAAGTTTGGTGGTGAGTTTTT	P	2	0.71
Ca GpSSR00661	Ca LG 4	(TA)12	6167585	6167608	Ca4	(TA)6	30087569	30087580		AGAAAATCAGAAGTTGCATT	AAAGTTTGGTGGTGAGTTTTT	P	3	0.57
Ca GpSSR00560	Ca LG 4	(AT)11	16377652	16377673	Ca4	(AT)18	10738319	10738354		GCCTAAAATCATAAAACCAAAG	ATGCAAGGGTTATTTTATGTC	P	3	0.77
Ca GpSSR00720	Ca LG 4	(TA)8	4703375	4703390	Ca4	(TA)10	38772350	38772369	Upstream	AGAAAATCAGAAGTTGCATT	AAAGTTTGGTGGTGAGTTTTT	N	N	0.00
Ca GpSSR00591	Ca LG 4	(TA)8	9027366	9027381	Ca4	(TA)9	15473255	15473272	Upstream	AGAAAATCAGAAGTTGCATT	AAAGTTTGGTGGTGAGTTTTT	N	N	0.00
Ca GpSSR00718	Ca LG 4	(TATT)5	4645602	4645621	Ca4	(TATT)6	38712506	38712529	Upstream	TAATTTTGTGGATCTGGCTTA	GGTTTGTGAGTCATGGTTTTA	N	N	0.00
Ca GpSSR00602	Ca LG 4	(AT)14	21639011	21639038	Ca4	(AT)8	17043303	17043318		AGAAAATCAGAAGTTGCATT	AAAGTTTGGTGGTGAGTTTTT	N	N	0.00
Ca GpSSR00588	Ca LG 4	(AT)21	8395710	8395751	Ca4	(AT)15	14821172	14821201		ATCATTTCCTTCGGTTCATC	TTCTTTTCTTCCTAAAAGGACA	N	N	0.00
Ca GpSSR00511	Ca LG 4	(TA)8	18432941	18432956	Ca4	(TA)17	1927537	1927570		ACGATATCGACATTTTGTCA	CGTACATATGCACCGAAAT	N	N	0.00
Ca GpSSR00533	Ca LG 4	(TA)11	20468878	20468899	Ca4	(TA)17	4527521	4527554	Upstream	ACGATATCGACATTTTGTCA	CGTACATATGCACCGAAAT	M	1	0.00
Ca GpSSR00580	Ca LG 4	(AT)15	10710704	10710733	Ca4	(AT)16	12836742	12836773	Upstream	TCTTATCACTGAATTGACACA	CAAACTTCACCAATACCACAT	M	1	0.00
Ca GpSSR00694	Ca LG 4	(AT)7	4881993	4882006	Ca4	(AT)6	36642951	36642962	Intron	AGAAAATCAGAAGTTGCATT	AAAGTTTGGTGGTGAGTTTTT	M	1	0.00
Ca GpSSR00579	Ca LG 4	(TA)7	10621458	10621471	Ca4	(TA)8	12748854	12748869	Intron	AGATGTACAATCTTTTGAGACAA	GCTGCAAAATGGTACATAGAC	M	1	0.00
Ca GpSSR00730	Ca LG 4	(TA)20	3853392	3853431	Ca4	(TA)8	40789270	40789285		AGAAAATCAGAAGTTGCATT	AAAGTTTGGTGGTGAGTTTTT	M	1	0.00
Ca GpSSR00917	Ca LG 4	(TA)7	8086803	8086816	Ca5	(TA)12	34466075	34466098		AGATGTACAATCTTTTGAGACAA	GCTGCAAAATGGTACATAGAC	M	1	0.00
Ca GpSSR00559	Ca LG 4	(AT)7	16368737	16368750	Ca4	(AT)13	10729912	10729937		CTTCTTTATTCGCAGTACCAA	GGGCTTCTGTGACACTATAAA	M	1	0.00
Ca GpSSR00693	Ca LG 4	(TA)6	4863426	4863437	Ca4	(TA)23	36627420	36627465		AGATGTACAATCTTTTGAGACAA	GCTGCAAAATGGTACATAGAC	M	1	0.00
Ca GpSSR00799	Ca LG 5	(AT)16	15438916	15438947	Ca5	(AT)12	9708217	9708240		AGAGTTGTCAATGGATCGGA	TCGCTTTTGCAAATGACAAC	N	N	0.00
Ca GpSSR00834	Ca LG 5	(TAA)8	15138160	15138183	Ca5	(TAA)10	20176966	20176995		AACGCTTTCCTACCAATTTTT	GGAGGAAGTCGTTAACAAAAGA	P	5	0.81
Ca GpSSR00893	Ca LG 5	(AT)20	1404766	1404805	Ca5	(AT)14	31101286	31101313		GTCCCAAAGCAATTTATTATC	CACTGCCACCGTATTATTAAA	P	5	0.84
Ca GpSSR00897	Ca LG 5	(AT)17	1796552	1796585	Ca5	(AT)13	31503890	31503915		GTCCCAAAGCAATTTATTATC	CACTGCCACCGTATTATTAAA	P	7	0.85
Ca GpSSR00904	Ca LG 5	(TC)9	2592222	2592239	Ca5	(TC)24	32627922	32627969	Intron	TTCAGGATTTGCTTGATGGA	CATAACCGCATAAGGGCATT	N	N	0.00
Ca GpSSR00907	Ca LG 5	(AT)21	2783303	2783344	Ca5	(AT)9	32879761	32879778		CATGCAACCTTCTTTTCAA	AGACATGGTAGATTAAAAGTGTG	P	2	0.59
Ca GpSSR00931	Ca LG 5	(AAT)35	4673759	4673863	Ca5	(AAT)20	35607530	35607589		TCATCTGATCACTAATACACCAA	GAAGAAAAAGAACCATTTGACT	P	3	0.79
Ca GpSSR00943	Ca LG 5	(TTA)7	5265718	5265738	Ca5	(TTA)5	37521123	37521137		CTTGGTGTGATCTTCACGGA	AGACAAAAACGGGCTAGTTAAA	P	8	0.84
Ca GpSSR00972	Ca LG 5	(AT)10	7983757	7983776	Ca5	(AT)13	43242240	43242265		TTTTtGTTACAACCATTTTTGGAT	GAAGCtATTTTGAAtTCTTTTGTTAAA	P	9	0.80
Ca GpSSR00974	Ca LG 5	(AT)20	8219412	8219451	Ca5	(AT)9	43474700	43474717		CATGCAACCTTCTTTTCAA	AGACATGGTAGATTAAAAGTGTG	M	1	0.00
Ca GpSSR00976	Ca LG 5	(AAT)23	8384037	8384105	Ca5	(AAT)18	43627432	43627485		TGGTTTTtAACAAAAaTATGTGGC	TCCCTCTTAAATTGATGGCAA	P	6	0.79
Ca GpSSR00980	Ca LG 5	(AT)10	10024504	10024523	Ca5	(AT)14	45557068	45557095		TTCAAATGGAAAAAGGTAATGAAA	CCTATGAATTTTTCATGATATCCaA	N	N	0.00
Ca GpSSR00955	Ca LG 5	(TA)23	6579859	6579904	Ca5	(TA)25	39836174	39836223	Upstream	GTCCCAAAGCAATTTATTATC	CACTGCCACCGTATTATTAAA	P	4	0.82
Ca GpSSR00954	Ca LG 5	(AT)11	6600947	6600968	Ca5	(AT)10	39815598	39815617	Intron	CTTCTTTACATCTGCTCTCCA	GCGCAAAATTCCTTTTAGTAT	P	3	0.71
Ca GpSSR00938	Ca LG 5	(TA)26	5771063	5771114	Ca5	(TA)13	36873482	36873507		GTCCCAAAGCAATTTATTATC	CACTGCCACCGTATTATTAAA	P	3	0.67
Ca GpSSR00914	Ca LG 5	(TA)11	3500799	3500820	Ca5	(TA)16	34084354	34084385	Upstream	CTTCTTTACATCTGCTCTCCA	GCGCAAAATTCCTTTTAGTAT	N	N	0.00
Ca GpSSR00992	Ca LG 5	(TA)11	7020694	7020715	Ca5	(TA)12	47689134	47689157	Upstream	CTTCTTTACATCTGCTCTCCA	GCGCAAAATTCCTTTTAGTAT	N	N	0.00
Ca GpSSR00952	Ca LG 5	(AT)13	11577667	11577692	Ca5	(AT)15	39537233	39537262	Intron	CTTCTTTACATCTGCTCTCCA	GCGCAAAATTCCTTTTAGTAT	N	N	0.00
Ca GpSSR00934	Ca LG 5	(TA)6	4949032	4949043	Ca5	(TA)7	35886784	35886797	Intron	GCTTTCAAAACATATCAACAAG	CCATCTACGTTGTCTCAGGTA	N	N	0.00
Ca GpSSR00937	Ca LG 5	(TA)17	5609103	5609136	Ca5	(TA)10	36723236	36723255		TGCGTAAGAGAGGTATATGGA	GGGAGTCAGCACCATTTAT	N	N	0.00
Ca GpSSR00832	Ca LG 5	(AT)19	14320433	14320470	Ca5	(AT)24	19863173	19863220		GTCCCAAAGCAATTTATTATC	CACTGCCACCGTATTATTAAA	N	N	0.00
Ca GpSSR00465	Ca LG 5	(AT)8	16100890	16100905	Ca3	(AT)25	32394131	32394180		CTTCTTTACATCTGCTCTCCA	GCGCAAAATTCCTTTTAGTAT	N	N	0.00
Ca GpSSR00982	Ca LG 5	(AT)8	10072741	10072756	Ca5	(AT)26	45603419	45603470		CTTCTTTACATCTGCTCTCCA	GCGCAAAATTCCTTTTAGTAT	N	N	0.00
Ca GpSSR00902	Ca LG 5	(TA)11	2317836	2317857	Ca5	(TA)9	32360829	32360846	Upstream	CTTCTTTACATCTGCTCTCCA	GCGCAAAATTCCTTTTAGTAT	M	1	0.00
Ca GpSSR00924	Ca LG 5	(AT)9	4380108	4380125	Ca5	(AT)10	35157633	35157652	Upstream	CTTCTTTACATCTGCTCTCCA	GCGCAAAATTCCTTTTAGTAT	M	1	0.00
Ca GpSSR00932	Ca LG 5	(TA)9	4834792	4834809	Ca5	(TA)11	35770233	35770254	Upstream	CTTCTTTACATCTGCTCTCCA	GCGCAAAATTCCTTTTAGTAT	M	1	0.00
Ca GpSSR00971	Ca LG 5	(AT)9	7947853	7947870	Ca5	(AT)8	43206162	43206177	Upstream	CTTCTTTACATCTGCTCTCCA	GCGCAAAATTCCTTTTAGTAT	M	1	0.00
Ca GpSSR00978	Ca LG 5	(AT)11	9818919	9818940	Ca5	(AT)10	45350530	45350549	Upstream	CTTCTTTACATCTGCTCTCCA	GCGCAAAATTCCTTTTAGTAT	M	1	0.00
Ca GpSSR00898	Ca LG 5	(AT)10	1955721	1955740	Ca5	(AT)9	31663883	31663900	exon, 3'-UTR	CTTCTTTACATCTGCTCTCCA	GCGCAAAATTCCTTTTAGTAT	M	1	0.00
Ca GpSSR00958	Ca LG 5	(ATT)6	10989647	10989664	Ca5	(ATT)5	40661717	40661731	Intron	CCAAACTAAATATTGCAAAC	GCACAGGACAATAAAATGAAC	M	1	0.00
Ca GpSSR00960	Ca LG 5	(TA)18	11151147	11151182	Ca5	(TA)7	40819373	40819386		CATGCAACCTTCTTTTCAA	AGACATGGTAGATTAAAAGTGTG	M	1	0.00
Ca GpSSR01219	Ca LG 5	(AT)15	164047	164076	Ca6	(AT)27	45622795	45622848		AGCACTTGTTAACGTTTTCTT	GTCAATCTATTGTGATGTTTTTG	M	1	0.00
Ca GpSSR01019	Ca LG 6	(AT)11	10946387	10946408	Ca6	(AT)14	6113644	6113671	Intron	TCAAATCAACGTTGCAAAAA	GTGCGATCGTACATAACCCT	N	N	0.00
Ca GpSSR01024	Ca LG 6	(AT)13	5756686	5756711	Ca6	(AT)25	7639034	7639083	Intron	AACCACATTTGTTGTCGCTG	CAAAATAAATGAAGGAGAGAATAAAGC	P	6	0.84
Ca GpSSR01030	Ca LG 6	(AAT)14	6020547	6020588	Ca6	(AAT)17	7901085	7901135		TGAATGACAATTTTAACAAATGGA	TTTCTTCTATGTCTTTTGTGTGTCAA	N	N	0.00
Ca GpSSR01045	Ca LG 6	(TA)9	6752543	6752560	Ca6	(TA)29	9795289	9795346		TTTGTTGAATAGGTCTTGTTTTGAA	CTGATGTGGGAAAATTCCAG	P	3	0.61
Ca GpSSR01067	Ca LG 6	(ATA)29	7442254	7442340	Ca6	(ATA)19	11038836	11038892		GAGCTCAATAATTGGATTAGATTTATG	TGGTACAAAATACCAGGATCAA	P	5	0.85
Ca GpSSR01089	Ca LG 6	(TAT)21	8487238	8487300	Ca6	(TAT)17	16372440	16372490		TTCTGCTTTCCAACCAAaGA	AGTCAAAGTGAATCCGTGATGA	N	N	0.00
Ca GpSSR01090	Ca LG 6	(AT)15	8512479	8512508	Ca6	(AT)12	16397769	16397792		CAtGcGAaTttAGgAagaGAGA	aGACATGGAGAGACCAAACAA	P	8	0.78
Ca GpSSR01084	Ca LG 6	(AT)18	10727121	10727156	Ca6	(AT)25	14786648	14786697	Upstream	TGAAAACATCTAAATTTCTACCG	CAGCTAACAATTAAACCTCCA	P	3	0.78
Ca GpSSR01344	Ca LG 6	(TA)22	7059393	7059436	Ca7	(TA)11	12090643	12090664		ACACACAATGAAGATTGAACA	TTCATCTTTGTTTCTTTCACG	P	3	0.72
Ca GpSSR01080	Ca LG 6	(TA)6	8715974	8715985	Ca6	(TA)26	13550243	13550294	Upstream	AAAGGTCGAACTTGGAGTTAC	GAATTAAGGAGCCAAGACAAC	N	N	0.00
Ca GpSSR00310	Ca LG 6	(TA)9	820927	820944	Ca2	(TA)10	35158756	35158775	Upstream	TTTCTTCCTCATTTCAACAA	GAGTGACGTATTTTCTCAACTG	N	N	0.00
Ca GpSSR01028	Ca LG 6	(AT)16	5843570	5843601	Ca6	(AT)25	7733096	7733145		TGAAAACATCTAAATTTCTACCG	CAGCTAACAATTAAACCTCCA	N	N	0.00
Ca GpSSR01003	Ca LG 6	(AT)6	3984494	3984505	Ca6	(AT)23	2902039	2902084		AAAGGTCGAACTTGGAGTTAC	GAATTAAGGAGCCAAGACAAC	N	N	0.00
Ca GpSSR01091	Ca LG 6	(TA)6	10491425	10491436	Ca6	(TA)30	16527609	16527668		AAAGGTCGAACTTGGAGTTAC	GAATTAAGGAGCCAAGACAAC	N	N	0.00
Ca GpSSR00332	Ca LG 6	(TA)13	4302942	4302967	Ca3	(TA)16	3272994	3273025	Upstream	CAAATATGCAATAACCTTCCA	AAAAATCTATTTGGTGCTGGT	M	1	0.00
Ca GpSSR01076	Ca LG 6	(TC)8	7917964	7917979	Ca6	(TC)7	12935212	12935225	Upstream	TCCAGATTCGTCTTCTCTGTA	AGGTGAAGATGAAGAGCTACC	M	1	0.00
Ca GpSSR00185	Ca LG 6	(AT)11	4288001	4288022	Ca2	(AT)10	1657272	1657291	Upstream	TTTCTTCCTCATTTCAACAA	GAGTGACGTATTTTCTCAACTG	M	1	0.00
Ca GpSSR01046	Ca LG 6	(TC)9	6793081	6793098	Ca6	(TC)8	9834493	9834508	Intron	TCCAGATTCGTCTTCTCTGTA	AGGTGAAGATGAAGAGCTACC	M	1	0.00
Ca GpSSR00997	Ca LG 6	(AT)13	2022244	2022269	Ca6	(AT)23	620795	620840		CTAGAGACGTTTAGGTGGACA	GGCAATACCACCATCTAAGTA	M	1	0.00
Ca GpSSR01378	Ca LG 7	(AT)13	5655354	5655379	Ca7	(AT)18	19179527	19179562	Intron	TGAGTGTGTACTGGGGTGAAA	AACACGCCCCTTCTTATCCT	P	8	0.85
Ca GpSSR01388	Ca LG 7	(TA)6	5038054	5038065	Ca7	(TA)28	20922821	20922876		GAAATTGAACCCAcAATTCCA	AATTGGCAACGGTGAGAAAA	M	1	0.00
Ca GpSSR01469	Ca LG 7	(AT)11	4231995	4232016	Ca7	(AT)23	44752656	44752701	Upstream	CCTCCCTCCAATTTATACCTA	CACTTTTGCCCAACTATTTTA	P	3	0.67
Ca GpSSR01359	Ca LG 7	(TA)15	6115799	6115828	Ca7	(TA)16	13800048	13800079	Upstream	GCCTAAAAGATATACAATTCACT	TCAAAGTGTTCGATGATTGTC	P	3	0.74
Ca GpSSR01470	Ca LG 7	(AT)13	4157545	4157570	Ca7	(AT)18	44863063	44863098		ACACACAATGAAGATTGAACA	TTCATCTTTGTTTCTTTCACG	P	5	0.80
Ca GpSSR01507	Ca LG 7	(AT)8	851006	851021	Ca8	(AT)7	4987846	4987859	Upstream	CTAAACCCTAAACCCAACATT	TTAATCTATCTACCCACATGGT	N	N	0.00
Ca GpSSR01334	Ca LG 7	(AT)6	7150572	7150583	Ca7	(AT)24	10403718	10403765		CCTCCCTCCAATTTATACCTA	CACTTTTGCCCAACTATTTTA	N	N	0.00
Ca GpSSR00099	Ca LG 7	(TAT)11	2721252	2721284	Ca1	(TAT)10	20300791	20300820	Upstream	AAGTTGTTCTTGTTGTTACCG	GAACATTTCTGCGAAAATAA	M	1	0.00
Ca GpSSR00613	Ca LG 7	(TA)8	450058	450073	Ca4	(TA)7	18980290	18980303	Upstream	AGAAAATCAGAAGTTGCATT	AAAGTTTGGTGGTGAGTTTTT	M	1	0.00
Ca GpSSR00691	Ca LG 7	(AC)9	1755599	1755616	Ca4	(AC)8	36460460	36460475	Intron	ACTTCAAAATAGAGGCCATTC	GGTGTGTCTCAATTCAGTGTT	M	1	0.00
Ca GpSSR01370	Ca LG 7	(AT)11	5430148	5430169	Ca7	(AT)6	16193831	16193842		ACACACAATGAAGATTGAACA	TTCATCTTTGTTTCTTTCACG	M	1	0.00
Ca GpSSR01379	Ca LG 7	(AT)12	5474320	5474343	Ca7	(AT)31	19371993	19372054		GTTTTCTTGGTCTCGATTTTT	TGAGTATATGCAACACACTACAA	M	1	0.00
Ca GpSSR00106	Ca LG 8	(TAT)28	9092309	9092392	Ca1	(TAT)17	21529812	21529862		CCTACGTGGACCCTCTTATTA	GCTTGTGTGGTTGATTTTATC	P	6	0.81
Ca GpSSR00765	Ca LG 8	(AG)11	306000	306021	Ca5	(AG)36	182413	182484		GGTGAAAATCAAAAGTAGGAGA	GGTATTCCTTCCATCCTCTT	P	10	0.80
Ca GpSSR01313	Ca LG 8	(TA)23	5909306	5909351	Ca7	(TA)8	6059605	6059620	Intron	ACACACAATGAAGATTGAACA	TTCATCTTTGTTTCTTTCACG	P	4	0.71
Ca GpSSR01312	Ca LG 8	(TA)11	6040663	6040684	Ca7	(TA)30	5927407	5927466	Upstream	CCACTAGTATTATGGACAAATCA	GTGGTTTGCAAGGTTTTATATT	P	2	0.66
Ca GpSSR01308	Ca LG 8	(AT)10	4818999	4819018	Ca7	(AT)9	4722840	4722857	Intron	CCACTAGTATTATGGACAAATCA	GTGGTTTGCAAGGTTTTATATT	P	5	0.85
Ca GpSSR00764	Ca LG 8	(AT)14	231831	231858	Ca5	(AT)15	105148	105177	Intron	TTTACCGGTGTAAATGGAC	TGGTATCATGTGTTTGATGTG	P	2	0.59
Ca GpSSR00105	Ca LG 8	(TA)14	9073186	9073213	Ca1	(TA)20	21511797	21511836		TTTACCGGTGTAAATGGAC	TGGTATCATGTGTTTGATGTG	P	3	0.59
Ca GpSSR01326	Ca LG 8	(AT)8	6550522	6550537	Ca7	(AT)9	8434059	8434076	Upstream	ACACACAATGAAGATTGAACA	TTCATCTTTGTTTCTTTCACG	N	N	0.00
Ca GpSSR01329	Ca LG 8	(TA)6	8105733	8105744	Ca7	(TA)8	9630834	9630849	exon, 3'-UTR	ACACACAATGAAGATTGAACA	TTCATCTTTGTTTCTTTCACG	N	N	0.00
Ca GpSSR01309	Ca LG 8	(TA)19	4931040	4931077	Ca7	(TA)22	4834678	4834721	Upstream	CCTCCCTCCAATTTATACCTA	CACTTTTGCCCAACTATTTTA	M	1	0.00
Ca GpSSR01314	Ca LG 8	(TA)6	5909025	5909036	Ca7	(TA)12	6059790	6059813	Intron	ACACACAATGAAGATTGAACA	TTCATCTTTGTTTCTTTCACG	M	1	0.00
Ca GpSSR00191	scaffold00078	(TA)20	92569	92608	Ca2	(TA)25	3282129	3282178		ACAATTCCACTTAATCTTTGC	AATTAGCCTACAGACACACACA	N	N	0.00
Ca GpSSR01519	scaffold00105	(TAA)14	304589	304630	Ca8	(TAA)10	6976160	6976189	Intron	GCGAAAAAGACAACGAAAGC	TCATCGGATTGGTCCTCTTC	P	5	0.78
Ca GpSSR01547	scaffold00119	(AT)7	276894	276907	Ca8	(AT)14	14524418	14524445		GCTTGTTTTCTTAAGTTGCCACA	tTGAATcATTaAACaAAAtTTCCTG	N	N	0.00
Ca GpSSR01049	scaffold00157	(AT)23	23949	23994	Ca6	(AT)7	9927325	9927338	Intron	CCTCTTTTCACACTTCCTTTT	ATTTCTGATATGGGTCACTCC	M	1	0.00
Ca GpSSR00213	scaffold00166	(TA)7	48977	48990	Ca2	(TA)14	8239573	8239600		TTTTAACATCCAGAaGTAAAAGAAAa	ATcCCATAcACCgaCGACAT	M	1	0.00
Ca GpSSR00649	scaffold00167	(TA)12	203749	203772	Ca4	(TA)8	28591976	28591991		AGAAAATCAGAAGTTGCATT	AAAGTTTGGTGGTGAGTTTTT	N	N	0.00
Ca GpSSR01141	scaffold00173	(ATA)23	208703	208771	Ca6	(ATA)19	23003198	23003254		AAGATACCAAACCTTTCCTTG	GTGTTAATGAAGGGGTATTTTT	N	N	0.00
Ca GpSSR01458	scaffold00197	(ATT)25	34552	34626	Ca7	(ATT)21	39389067	39389129		GAAGGAAATATATCCCTTACAAGTGTG	ACGGTAAACTATAAGACGTGTCG	P	5	0.79
Ca GpSSR00464	scaffold00198	(AT)10	252116	252135	Ca3	(AT)11	31982547	31982568	exon, 3'-UTR	CAAATATGCAATAACCTTCCA	AAAAATCTATTTGGTGCTGGT	P	2	0.74
Ca GpSSR00154	scaffold00206	(TAA)26	181997	182074	Ca1	(TAA)19	41069980	41070036		CCCTCAAGCAACCCATAAAT	TTGACACCATATGTGTTCTCCC	P	5	0.73
Ca GpSSR01725	scaffold00212	(AT)23	188136	188181	scaffold40	(AT)16	601181	601212		TGTAAATGTAGATGATTGTTTGC	ACAAACTGATTCACGAAATTG	N	N	0.00
Ca GpSSR00286	scaffold00225	(AAT)19	202872	202928	Ca2	(AAT)29	30988922	30989008		TTGAAGATATATACATGAGCAAC	GGAGCTTTCTTTGATTTTTCT	M	1	0.00
Ca GpSSR00849	scaffold00225	(AT)16	81661	81692	Ca5	(AT)21	25756857	25756898		TtATATAAAaTTCGATATGCAGGATCT	tCTcAATTCaAACTTCCAcTCAA	M	1	0.00
Ca GpSSR00880	scaffold00245	(TA)9	38632	38649	Ca5	(TA)15	29479495	29479524		CTCGTACTGTGAGAAAACCTcG	AGCTTGCAAATGCGATTCTT	P	6	0.67
Ca GpSSR00882	scaffold00245	(ATA)21	117729	117791	Ca5	(ATA)18	29568805	29568858		TTTTTCTTTTCCATAATGTGCG	CACCGGTTCAATTTTCGATT	P	4	0.79
Ca GpSSR00383	scaffold00246	(TAA)29	151828	151914	Ca3	(TAA)6	16728236	16728253		TTTGTTAACCATTGGATTGAG	CATCCACTGACCAAAAATAAA	M	1	0.00
Ca GpSSR00654	scaffold00248	(TAT)11	62270	62302	Ca4	(TAT)9	28939194	28939220		CACCCATGCCTTATAGATACA	TTTAAATTGAGTTCACGTAGTCT	P	6	0.82

Ca_GpSSR00655	scaffold00248	(AT)9	160687	160704	Ca4	(AT)8	29038167	29038182	exon, 3'-UTR	GACTGTAGAGGAGCACTGATG	GCACACAACAACAAAATTTATC	M	1	0.00
Ca_GpSSR00749	scaffold00259	(AT)16	189743	189774	Ca4	(AT)12	45811549	45811572		AGACGTCCTTAGGGgATTTTT	TTTAAGGaAGTCGCCTTTGC	M	1	0.00
Ca_GpSSR00268	scaffold00297	(AT)19	18188	18225	Ca2	(AT)12	26470801	26470824		TTTCTTCCTCATTTCAACAA	GAGTGACGTATTTTCTCAACTG	P	3	0.81
Ca_GpSSR01348	scaffold00308	(TA)20	163021	163060	Ca7	(TA)7	12275492	12275505		ACACACAATGAAGATTGAACA	TTCATCTTTGTTTCTTTCACG	P	3	0.69
Ca_GpSSR00023	scaffold00341	(AT)12	16023	16046	Ca1	(AT)18	5270952	5270987		TTTGATTCCCTCGTTAAATTATGA	AAAGGCTAGATCAACAACGTACaA	N	N	0.00
Ca_GpSSR00762	scaffold00351	(ATAGTA)19	127986	128099	Ca4	(ATAGTA)11	49097626	49097691		AAAAGTATGAGCAACGCACT	TCTAAAAAGACTACATCCTAACA	N	N	0.00
Ca_GpSSR00064	scaffold00360	(TTA)14	145166	145207	Ca1	(TTA)17	15928923	15928973		CGACCATCGTCATATATTTTT	TTGTGTTTCCCAAATTTTTC	M	1	0.00
Ca_GpSSR01781	scaffold00378	(AT)10	168761	168780	scaffold674	(AT)6	268478	268489	Intron	ATGATTTGGGCATTGTTTAG	GGATGCACAAGATTGATTAAA	N	N	0.00
Ca_GpSSR00709	scaffold00423	(TA)7	24842	24855	Ca4	(TA)12	38232543	38232566		CAACGACAAATCGACAATGG	CGAATTCGACAAAACTACAATAAATG	P	7	0.71
Ca_GpSSR00699	scaffold00495	(AT)9	121129	121146	Ca4	(AT)12	36887863	36887886	Intron	GACTAGTCCAACATTTGACTTTTCTT	TCACATAAGTGGTCCTGCCA	M	1	0.00
Ca_GpSSR00627	scaffold00511	(TA)12	126554	126577	Ca4	(TA)9	21770744	21770761	Intron	AATTTGCATCTATCTCAAGAACT	CGTTCTTTTAATACTGATTTGAC	P	3	0.68
Ca_GpSSR01421	scaffold00515	(TA)18	7963	7998	Ca7	(TA)6	31262184	31262195		TTACGGTATCACTTTTAGATCA	ATTAAGGCCACAAAATGAATG	M	1	0.00
Ca_GpSSR01102	scaffold00526	(TTA)26	150872	150949	Ca6	(TTA)18	17385162	17385215		TTCTCTTTTTCCCATTAATCC	CATCCCTCGTTAGCATAAATA	P	4	0.78
Ca_GpSSR01440	scaffold00538	(AT)11	54585	54606	Ca7	(AT)17	34897369	34897402		TGTGAGTCGTGTCACAATAAAAA	TGTTTATCAGGTTCAAGCCG	P	6	0.74
Ca_GpSSR01419	scaffold00539	(TA)12	93728	93751	Ca7	(TA)9	30631979	30631996		ACGAACTCTTGACCCCAAAT	CCGTCATTACATAACAATTGACC	P	5	0.85
Ca_GpSSR00209	scaffold00541	(TTA)35	67182	67286	Ca2	(TTA)20	7620184	7620243		CCTTGCGTGAGTTTGACGTA	CATCGCGGTCATGTGaGTTA	P	8	0.85
Ca_GpSSR01020	scaffold00582	(TTA)25	60330	60404	Ca6	(TTA)23	6333208	6333276		CTGAAAATGGAAAATCAATCAG	TCAACCTAATAAGAGAATGAACA	P	8	0.83
Ca_GpSSR01663	scaffold00634	(TA)12	192481	192504	scaffold242	(TA)7	696951	696964		TCCTTTCTTTCTATTCTCTCCA	GAGAATTTAATGCGTTACGG	P	2	0.66
Ca_GpSSR00096	scaffold00647	(AT)16	30709	30740	Ca1	(AT)26	20115911	20115962		CAATCAAGCTCGTATGGAACA	CATCAATTATTCAACTACCAAtTTTC	P	10	0.78
Ca_GpSSR00617	scaffold00669	(ATA)29	18822	18908	Ca4	(ATA)7	20009819	20009839		TCTGATAAATCAATCACAAGC	TTACAATTGCAGCTTAAAACG	P	6	0.82
Ca_GpSSR00574	scaffold00690	(TAA)17	11781	11831	Ca4	(TAA)20	12613373	12613432		AAGTCAGAAGGTCATACTCAAA	TGGTCTCCAATTATACAGAAAA	M	1	0.00
Ca_GpSSR00633	scaffold00690	(TA)18	63692	63727	Ca4	(TA)10	24106872	24106891		AGAAAATCAGAAGTTGCATT	AAAGTTTGGTGGTGAGTTTTT	M	1	0.00
Ca_GpSSR00625	scaffold00703	(TAT)27	113509	113589	Ca4	(TAT)18	21528401	21528454		CATTCCTTCAGCATTTCTTTT	CGTAGAATTTACCGTTGTTCT	P	11	0.75
Ca_GpSSR00357	scaffold00726	(TTTA)8	101734	101765	Ca3	(TTTA)6	9241047	9241070		GGATCACATGTGGTATTTAGCC	GCACATGTTCACCGACATTC	P	8	0.78
Ca_GpSSR01475	scaffold00736	(AT)13	135994	136019	Ca7	(AT)7	45759545	45759558		TTTtGCTTCAAGTCATCGCA	CCTTTACGCACCCTATCGAG	M	1	0.00
Ca_GpSSR00957	scaffold00748	(TA)22	111999	112042	Ca5	(TA)25	40544921	40544970	Intron	TGCTAAAGTAGCAAGCCaCAAA	cGGaAgAAGATgGTgaAGATG	N	N	0.00
Ca_GpSSR00472	scaffold00767	(TA)27	52777	52830	Ca3	(TA)19	33953817	33953854		CAAATATGCAATAACCTTCCA	AAAAATCTATTTGGTGCTGGT	P	5	0.80
Ca_GpSSR00371	scaffold00772	(AAT)15	89080	89124	Ca3	(AAT)17	11701006	11701056		GCCCACGATATCCCctATTT	GGGAGAGCAAAGAAGAAAGG	P	3	0.83
Ca_GpSSR00090	scaffold00795	(GA)11	52302	52323	Ca1	(GA)15	19550771	19550800	Intron	aGCGAAAATTCGAGAAACGA	ACTCCCCACCACtTTCCTCT	P	9	0.75
Ca_GpSSR00091	scaffold00795	(AG)10	73052	73071	Ca1	(AG)12	19574561	19574584	exon, 5'-UTR	CCAAAGCAAGAATTACACGC	CTTTACATTTACGCCCCCAA	M	1	0.00
Ca_GpSSR01380	scaffold00797	(AT)13	36918	36943	Ca7	(AT)10	19534300	19534319	Intron	ACACACAATGAAGATTGAACA	TTCATCTTTGTTTCTTTCACG	P	8	0.84
Ca_GpSSR00134	scaffold00845	(AT)16	38444	38475	Ca1	(AT)10	30828736	30828755		TCACACATTTTAACCTAACCGC	TGTGTGTGTGtGTTTGTGTGTG	N	N	0.00
Ca_GpSSR00217	scaffold00846	(AAT)15	26456	26500	Ca2	(AAT)11	9298700	9298732	Intron	TTGGTAGGAAGGTTGATCTGTTC	TGGCCCTAAATATTAGTATCATTTCAA	P	6	0.80
Ca_GpSSR00823	scaffold00857	(TTA)24	65131	65202	Ca5	(TTA)27	17774911	17774991		tGTGTGTGGACGTGTGATTG	TTCGTTTACCAATAcCAAtTTTCa	P	4	0.82
Ca_GpSSR01539	scaffold00911	(AAATAT)6	140201	140236	Ca8	(AAATAT)7	12406708	12406749		GAAACCAAcATGATATgATAAGAaCAA	TGAAAAAGACAAAAATACCCTTATAGT	M	1	0.00
Ca_GpSSR01540	scaffold00911	(TTA)46	195177	195314	Ca8	(TTA)19	12460561	12460617		GGTTCTTATATTTCCAATTCCA	GACCACACTAGCAACAAAAAC	M	1	0.00
Ca_GpSSR00843	scaffold00924	(ATT)24	65927	65998	Ca5	(ATT)20	24073883	24073942		CCAAACTAAATATTGCAAAC	GCACAGGACAATAAAATGAAC	P	7	0.86
Ca_GpSSR01530	scaffold00940	(AT)23	9456	9501	Ca8	(AT)20	9102167	9102206		GTATGGAGATGAAATGTGGAA	CGTAAATCAACTTCTAAATTCG	P	3	0.86
Ca_GpSSR00609	scaffold00943	(TA)10	38367	38386	Ca4	(TA)9	18430227	18430244	exon, 3'-UTR	TGAGAGTGAGGTATTTTGGTG	CTCCTGTAGACAAAATTCGTG	P	3	0.81
Ca_GpSSR01522	scaffold00977	(TAATA)30	111975	112124	Ca8	(TAATA)21	7460728	7460832		AACGTGAGACTTCAATTTTCTT	GTCCAACCATATTCACACAAC	P	4	0.81
Ca_GpSSR00146	scaffold01007	(AT)26	43756	43807	Ca1	(AT)8	38750885	38750900		TCACAACAGTTGACATTATACTC	CGGAACAATCTCTCAAAAGTT	P	2	0.60
Ca_GpSSR00732	scaffold01081	(TAATA)7	63544	63578	Ca4	(TAATA)44	40920226	40920445	Intron	TGATCATAGGAAATAACTCAAGA	GTTGCTTTTCCATCCTTTTTA	P	5	0.84
Ca_GpSSR01640	scaffold01110	(TAT)26	10473	10550	scaffold198	(TAT)21	112567	112629		TGATGTTAATTTGTGTCCGTA	TGCATTCTGGTGAGAGTATTT	M	1	0.00
Ca_GpSSR01598	scaffold01155	(AAT)22	31347	31412	scaffold1466	(AAT)27	208515	208595	Intron	TCAACTCATAAATAAACGGATTTACC	TcCCTAAtTTTAAGaAAGGaAgGAG	N	N	0.00
Ca_GpSSR00161	scaffold01182	(AT)25	37708	37757	Ca1	(AT)10	43397528	43397547		ATCGCGTTAAATACTTGGACT	CATATGTGGAATGGACTATGC	M	1	0.00
Ca_GpSSR01724	scaffold01188	(TAT)14	54721	54762	scaffold40	(TAT)11	496476	496508		TGTTGTTTACACACCGTAAGA	AAGGAAAATCATTCTTAGTCCA	M	1	0.00
Ca_GpSSR00686	scaffold01247	(CT)7	4643	4656	Ca4	(CT)9	34309140	34309157	exon, 5'-UTR	ACATCCGGTTACCCCTCTCT	CGTAACGGTTTTCTTCACGC	M	1	0.00
Ca_GpSSR01273	scaffold01333	(TA)12	150230	150253	Ca6	(TA)6	54983871	54983882		TGACGTGTCATGCATCAAATAA	TGAATCGTTCATCAAACACGA	M	1	0.00
Ca_GpSSR00491	scaffold01341	(AT)19	8088	8125	Ca3	(AT)10	36484834	36484853		CAAATATGCAATAACCTTCCA	AAAAATCTATTTGGTGCTGGT	N	N	0.00
Ca_GpSSR00278	scaffold01369	(TA)18	1	36	Ca2	(TA)6	29878998	29879009		TTTCTTCCTCATTTCAACAA	GAGTGACGTATTTTCTCAACTG	M	1	0.00
Ca_GpSSR00723	scaffold01372	(TA)12	29391	29414	Ca4	(TA)9	39116041	39116058		GCAAACTATAATTCAAAAGCAC	TCTTGTTTGTTCAGGAAAGAG	M	1	0.00
Ca_GpSSR01684	scaffold01391	(CT)9	69833	69850	scaffold296	(CT)12	474416	474439	Intron	TGCGTTGTCGTTTTTGtGTT	AAATCTAATCGATTCCCGGC	P	2	0.75
Ca_GpSSR00339	scaffold01392	(AAT)13	47545	47583	Ca3	(AAT)19	4596676	4596732		GTGCACACGGGTTACACAGT	GTGCGGTGGTTGAAGGTAGT	P	7	0.81
Ca_GpSSR01593	scaffold01508	(ATA)17	48250	48300	scaffold1439	(ATA)14	181874	181915		TGGTTTAACACAAAGTACCAAA	TACGATCGAGACTAGACCAAA	M	1	0.00
Ca_GpSSR01512	scaffold01516	(AAT)14	70973	71014	Ca8	(AAT)5	5368882	5368896		CCGAATCACGCTTTAATTTAT	TTCATTTTTCTCAAACTGCAC	M	1	0.00
Ca_GpSSR01551	scaffold01523	(AT)12	68116	68139	Ca8	(AT)20	15234735	15234774		TGCAATCTATTGCTACACAAATCA	CATGGCTAGTGCAATTCTCA	M	1	0.00
Ca_GpSSR00121	scaffold01704	(AAT)5	167393	167407	Ca1	(AAT)21	23641483	23641545		TGTGTTTCATGGGTCAGGTC	TCGtAAGtTTTTATTTATCAACGGG	N	N	0.00
Ca_GpSSR00149	scaffold01844	(ATT)25	51397	51471	Ca1	(ATT)20	40048662	40048721		ACATATCCTTTGTGAAAGTACAA	TTTTGTGTGTGTACATCTTTCA	P	8	0.84
Ca_GpSSR01072	scaffold01886	(TTCT)19	6987	7062	Ca6	(TTCT)15	12269011	12269070		GCCCAGTCTCTCCCTCTTTC	ACGTCACTCCCTTCCACAAA	P	9	0.80
Ca_GpSSR00797	scaffold01959	(AT)13	31999	32024	Ca5	(AT)8	8949986	8950001	Intron	TTATTGGGGACATAATTAGCA	TCAAAAACTTCAACATCCAAG	N	N	0.00
Ca_GpSSR01636	scaffold01979	(TA)24	35618	35665	scaffold1964	(TA)12	132537	132560	Intron	AAGAAAATTTTGCCCTATACC	TACTTCTGTTCCGGAAATGTT	P	4	0.83
Ca_GpSSR00199	scaffold02025	(AT)15	38351	38380	Ca2	(AT)8	4978808	4978823		TTTCTTCCTCATTTCAACAA	GAGTGACGTATTTTCTCAACTG	P	5	0.62
Ca_GpSSR01201	scaffold02042	(AT)15	36549	36578	Ca6	(AT)18	39428374	39428409		AACAAAAATAAGAATGGCGTGAA	GCTGAACGGTGCTTTTTAGG	N	N	0.00
Ca_GpSSR00813	scaffold02275	(AAT)30	17172	17261	Ca5	(AAT)24	13120133	13120204		GCGACCTCGCACTAGAACTT	GACTCCCAATTTCCTTCCCT	P	5	0.80
Ca_GpSSR00666	scaffold02278	(AAT)28	381203	381286	Ca4	(AAT)13	30412466	30412504		TGTTGACACCTAATTTTATTCG	CGAAAACACTTGTGATTGTTA	N	N	0.00
Ca_GpSSR00647	scaffold02387	(GA)7	41885	41898	Ca4	(GA)11	28061216	28061237		TGTTGGTGTTGAAATCACATA	GTCACTGTAAACAAACGGTGT	P	7	0.80
Ca_GpSSR00546	scaffold02410	(TAT)43	16189	16317	Ca4	(TAT)23	7450500	7450568	Intron	GTGTAATTTTTGGGGTGATTT	ACAAGACATATACTACGCACCA	P	9	0.84
Ca_GpSSR01401	scaffold02451	(AT)14	19603	19630	Ca7	(AT)11	23195330	23195351		TCAATTTTGAACGAGAAGTGAATC	CCAAAGAGTCTACTAGCGGAACA	P	5	0.79
Ca_GpSSR00284	scaffold02476	(AAT)10	10536	10565	Ca2	(AAT)6	30847526	30847543		TTGTGTGGAGCTTAGGGTGTC	CCAATTAGTATATTGCTTCACAGACAA	N	N	0.00
Ca_GpSSR00285	scaffold02476	(TA)8	12599	12614	Ca2	(TA)17	30849550	30849583		GACCGAAATTTTAACCCTTGG	CGGACGGCATATCTAAGTTGA	P	4	0.62
Ca_GpSSR01422	scaffold02485	(AT)10	21511	21530	Ca7	(AT)8	31588638	31588653	exon, 3'-UTR	ACACACAATGAAGATTGAACA	TTCATCTTTGTTTCTTTCACG	P	3	0.75
Ca_GpSSR01544	scaffold02527	(AT)16	14827	14858	Ca8	(AT)24	14211657	14211704		GAGAATAAAAaTGTTACAGCAAAATCA	AAAGGTTCAAGCAGGGTGAAT	P	4	0.79
Ca_GpSSR00113	scaffold02568	(AT)28	15444	15499	Ca1	(AT)13	22226170	22226195	Intron	TCTTACCATCACGAAATGTTGC	GTTGAATGATAATTGGTCAGACTTT	N	N	0.00
Ca_GpSSR00401	scaffold02646	(AAT)10	10978	11007	Ca3	(AAT)13	20537462	20537500		TTGCCTACTTTTCATGAGTTTG	TCTCGTTTAAAATCAATTATTCCG	P	5	0.79
Ca_GpSSR01445	scaffold02656	(AT)14	22536	22563	Ca7	(AT)7	36051091	36051104		TGATCATTAAAGAGTGTTGCTATCAG	CGTACGTTGCACGGaAATAA	N	N	0.00
Ca_GpSSR01350	scaffold02660	(AAT)18	30951	31004	Ca7	(AAT)16	12347572	12347619		TGTGCACAAAAaGGTAGTAACAAAA	TGGTGGTGTAGTTGCATGATT	N	N	0.00
Ca_GpSSR01351	scaffold02660	(TCT)9	37157	37183	Ca7	(TCT)11	12362228	12362260		AGTCCCGTGCAAGAATTAAGA	CCCAATCCCAGCAATAAAAA	P	5	0.75
Ca_GpSSR01232	scaffold02694	(TA)14	29806	29833	Ca6	(TA)6	47604155	47604166		TGTCAACAAAAGaCTAGGCTAGGA	AATTTTGTAACGACCAAcAACA	M	1	0.00
Ca_GpSSR01557	scaffold02701	(TAT)19	28679	28735	Ca8	(TAT)7	16098448	16098468		TAAAaCCTtATTTtgCCGGA	GGGATAACGCCAAGGACATa	P	5	0.76
Ca_GpSSR00794	scaffold02788	(AT)14	68263	68290	Ca5	(AT)22	7882818	7882861		AAGTGGAGGTTGAAAAGCCC	ACGCAATTTAACGTTCCCaA	P	10	0.80
Ca_GpSSR01233	scaffold03024	(CTCTT)9	4747	4791	Ca6	(CTCTT)12	47687503	47687562		ATTTCTCCCCTTTTATTTCCT	GTTTGATATGGTTTGTTAGCC	M	1	0.00
Ca_GpSSR01406	scaffold03162	(AT)10	71391	71410	Ca7	(AT)13	24530702	24530727		TTtGTTTACAATGATATTtATGCcA	TCATTGtTCTGTGCCACttTTT	N	N	0.00
Ca_GpSSR01173	scaffold03171	(TA)11	21735	21756	Ca6	(TA)25	30333189	30333238		TTACCCCTTGTTCTATAATTATTTTGT	TCACCACAATTAGAACAAATTACCA	N	N	0.00
Ca_GpSSR01230	scaffold03419	(AAT)27	27662	27742	Ca6	(AAT)33	47490301	47490399		TTTGGAAGTTTGGAAGTTGCT	GGGTGAGGTGGTCCAATAAT	P	11	0.81
Ca_GpSSR00738	scaffold03544	(TA)11	18214	18235	Ca4	(TA)7	43437682	43437695		CCAAAAGGTTACGTTCAAAAAGA	GCTTTTTGCATTTGTTTCCAA	P	4	0.70
Ca_GpSSR01117	scaffold03622	(AAT)28	11728	11811	Ca6	(AAT)22	19583902	19583967		AATAGTTTCGGAATTTCCTTG	GTTAGTTAGTTAGGTTTTCGTTT	N	N	0.00
Ca_GpSSR00030	scaffold03683	(TTA)14	6258	6299	Ca1	(TTA)21	6978418	6978480	Intron	GCTCTCGGGATTGACATCAT	TTCACATCATGAACAAAAGGAAA	N	N	0.00
Ca_GpSSR00786	scaffold03730	(CCA)5	38043	38057	Ca5	(CCA)8	6286658	6286681	exon, CDS	GCCCCTcTTCCACCaTTCtA	GAGGTGGGGCCTTAGACATC	N	N	0.00
Ca_GpSSR00438	scaffold03779	(TA)8	19271	19286	Ca3	(TA)14	27573782	27573809		ATTTCTTATGGgCATGGgTG	GAATAGTCAAAGCGATAAAATAAATGA	N	N	0.00
Ca_GpSSR01032	scaffold03805	(TA)16	21081	21112	Ca6	(TA)12	7969856	7969879		TCAATAGTGTGTTTTGTGTAAGAGATT	TgGATATTAtTTgGtCAtGtTTTGA	N	N	0.00
Ca_GpSSR00819	scaffold03890	(AAT)21	22883	22945	Ca5	(AAT)18	16470096	16470149		TTGAACGTTATATCTTAGATTCG	GAAATAGAGAAAATCCCTACTCT	M	1	0.00
Ca_GpSSR01397	scaffold04310	(AT)21	17861	17902	Ca7	(AT)16	21907420	21907451		ACACACAATGAAGATTGAACA	TTCATCTTTGTTTCTTTCACG	P	10	0.80
Ca_GpSSR00644	scaffold04448	(TA)19	5777	5814	Ca4	(TA)8	27617346	27617361	Intron	TTACAACAATCACTCCGAAAT	GAGCTCGAAAAACTTTTAAGG	P	3	0.66
Ca_GpSSR01538	scaffold04506	(TC)19	8462	8499	Ca8	(TC)10	11875732	11875751		AGCTTTTAAAGGGTTCTTTTG	TTGTGTGTATGTGTGTGAGTGT	P	3	0.81
Ca_GpSSR00247	scaffold04790	(TA)24	12063	12110	Ca2	(TA)8	18676129	18676144		TTTCTTCCTCATTTCAACAA	GAGTGACGTATTTTCTCAACTG	M	1	0.00
Ca_GpSSR00076	scaffold04948	(TTA)29	8346	8432	Ca1	(TTA)21	17235425	17235487		CCTTTCCTTTTGATTAGGTTT	GGACTAATGACGTTAAAATCCA	N	N	0.00
Ca_GpSSR01685	scaffold05316	(AT)9	70767	70784	scaffold2	(AT)25	433707	433756	exon, 3'-UTR	TGGAGTTGTTTCAAATTGTTC	TGCTGCAGATATGGTTAAGTT	M	1	0.00
Ca_GpSSR00770	scaffold05350	(AT)22	8401	8444	Ca5	(AT)9	1433021	1433038		CATGCAACCTTCTTTTCAA	AGACATGGTAGATTAAAAGTGTG	M	1	0.00
Ca_GpSSR00824	scaffold05660	(TAA)21	6876	6938	Ca5	(TAA)25	17797031	17797105		CACATAGGTCAAACCCAATAA	ACTCACTCCTTAAGTGGAAGC	P	12	0.78
Ca_GpSSR00348	scaffold05887	(TTTA)5	8144	8163	Ca3	(TTTA)7	6672423	6672450	Intron	CCGTCTCTGCTAAGATTGCC	AATGGGATTAATGGTGGTGAA	P	6	0.85
Ca_GpSSR01759	scaffold05948	(AG)47	8494	8587	scaffold528	(AG)35	308678	308747		TCTTGTTTTGTGTTTTCGAGT	TGTGTCATCATCATCTCTCTCT	M	1	0.00
Ca_GpSSR00673	scaffold05983	(GA)53	5704	5809	Ca4	(GA)15	31448290	31448319		TAGAGGAAGCGTCGAATTTAC	TTCTCCCCCACTCTATTTTAC	P	8	0.81
Ca_GpSSR01181	scaffold06752	(TA)14	7947	7974	Ca6	(TA)9	31271189	31271206		GGGggCAtGTTTtATTTGtA	CGCTTCCCAACTACTTGTTT	N	N	0.00
Ca_GpSSR01528	scaffold07930	(AG)10	1	20	Ca8	(AG)6	8740976	8740987		AGAaCgTTGGAAGcGGtAGA	TTCGGAGCTAGGTTTGGTTG	M	1	0.00
Ca_GpSSR00307	scaffold08771	(AT)15	6108	6137	Ca2	(AT)7	35037307	35037320		TTTCTTCCTCATTTCAACAA	GAGTGACGTATTTTCTCAACTG	M	1	0.00
Ca_GpSSR01134	scaffold09107	(AT)6	3712	3723	Ca6	(AT)12	22163951	22163974		CAGTGACATTTCATTTTGTCTCC	TCGAGATTAGTTTGTTTATTCTTGGA	P	5	0.69
Ca_GpSSR00975	scaffold09195	(AT)10	3664	3683	Ca5	(AT)8	43551687	43551702	exon, 3'-UTR	ACGCCACTCTTTTGTTATTTT	CCAACTCATAAACACGTAACAC	P	2	0.77
Ca_GpSSR00316	scaffold10005	(TA)16	2405	2436	Ca2	(TA)8	35816147	35816162		GCAAAGTGGAATTTGTGGGT	aAAACATCAATTTACCATCATTCA	M	1	0.00
Ca_GpSSR00642	scaffold10216	(AT)25	1081	1130	Ca4	(AT)7	27363338	27363351		TGTCAACCTTAATGTTTTGGAA	TTgGGCTATTAGTCTTCCTATTCAA	P	6	0.80
Ca_GpSSR00036	scaffold10946	(TTA)23	16	84	Ca1	(TTA)16	8328466	8328513		AATTAACCAACCAATCCTACG	GCTTGTGTGGTTGATTTTATC	P	6	0.76
Ca_GpSSR00420	scaffold11189	(AT)7	759	772	Ca3	(AT)34	22930707	22930774		TcTCACAAGGATGGGAACAA	ATTAtCCAGGTTgACGCTGC	N	N	0.00
Ca_GpSSR00619	scaffold12694	(AT)16	3363	3394	Ca4	(AT)11	20884614	20884635		tCATTGTTtCTGAAAATGAAGGAT	CAGAAAATAGAAACGGAAAATTCG	N	N	0.00

Ca_GpSSR00587	scaffold14163	(AT)21	2894	2935	Ca4	(AT)11	14755594	14755615		AGAAAATCAGAAGTTGCATT	AAAGTTTGGTGGTGAGTTTTT	N	N	0.00
Ca_GpSSR00788	scaffold16375	(AT)24	2205	2252	Ca5	(AT)8	6371319	6371334		CATGCAACCTTCTTTTCAA	AGACATGGTAGATTAAAAGTGTG	M	1	0.00
Ca_GpSSR00672	scaffold16708	(AT)8	126	141	Ca4	(AT)25	31260052	31260101		CcACCAAAAtCCGTCAACTT	TGTTCCATCATAtTTTcAACCG	P	7	0.65
Ca_GpSSR00241	scaffold17215	(TA)20	21	60	Ca2	(TA)9	14283177	14283194		ACCCAATTTGGTTTCACTAA	TTGGACTTGAAAGCTTATTTC	N	N	0.00
Ca_GpSSR00059	scaffold21907	(AT)13	83	108	Ca1	(AT)28	15542865	15542920		GACCTAGTCCGCGACTCAAC	AgAcCCaAACATGTCGGTaAA	P	10	0.78
Ca_GpSSR00606	scaffold22996	(AT)21	1	42	Ca4	(AT)6	18163274	18163285		AGAAAGGTTTCACTACCCAAG	TCACTAGCCCTATTGTGTGAG	P	4	0.72
Ca_GpSSR00347	scaffold23582	(TA)29	12	69	Ca3	(TA)22	6377157	6377200		CAAATATGCAATAACCTTCCA	AAAAATCTATTTGGTGCTGGT	P	3	0.80
Ca_GpSSR00400	scaffold32929	(TA)18	8	43	Ca3	(TA)8	20272661	20272676		CAAATATGCAATAACCTTCCA	AAAAATCTATTTGGTGCTGGT	N	N	0.00
Ca_GpSSR00508	scaffold39578	(AT)17	838	871	Ca4	(AT)22	1732272	1732315		GGTGTCAAAAAGGTATAAAGGGG	CCTCGTGTCATTATTATATTTAACGGT	P	8	0.84
Ca_GpSSR00526	scaffold76751	(TAT)60	260	439	Ca4	(TAT)18	4266684	4266737		CATTCCTTCAGCATTTCTTTT	CGTAGAATTTACCGTTGTTCT	P	5	0.74
