# Supplementary material for: Development of genome-wide informative simple sequence repeat markers for large-scale genotyping applications in chickpea and development of web resource
Source: Front Plant Sci. 2015 Aug 21;6:645. doi: 10.3389/fpls.2015.00645 (PMC4543896; doi:10.3389/fpls.2015.00645)
Supplement: Supplementary file 3 [file Table_3.PDF]

Additional information

Genome-wide Development of Highly Polymorphic Simple Sequence Repeat Markers in Chickpea, and Development of Web Resource for Large-scale Genotyping Applications

Swarup Kumar Parida, Mohit Verma, Santosh Kumar Yadav, Supriya, Shouvik Das, Rohini Garg & Mukesh Jain

Functional and Applied Genomics Laboratory, National Institute of Plant Genome Research (NIPGR), Aruna Asaf Ali Marg, New Delhi - 110067, India

Figure S1. Genomic distribution of polymorphic simple sequence repeats on chickpea chromosomes. The polymorphic SSRs for which primers could be designed are highlighted in red color font. The validated SSRs are highlighted in green color font.

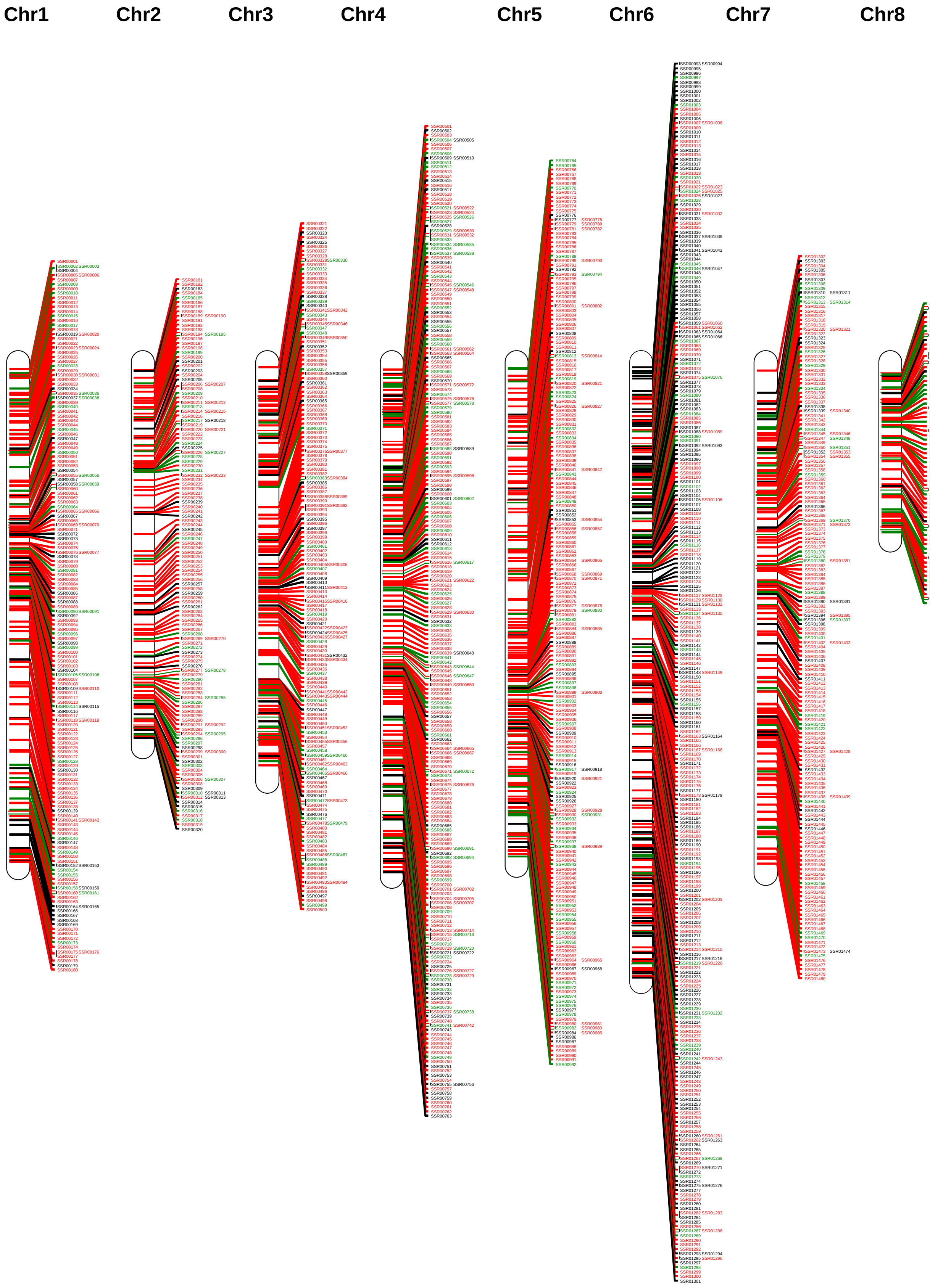

Supplementary Table S3. Chickpea genotypes used in the study for evaluating the amplification and polymorphic potential of SSR markers. The cultivar-specific classification and geographical origin of each genotype are also given.

Group	Genotype	Cultivar-specific classification	Geographical origin
**Ia**	ICC4958	*Cicer arietinum Desi*	Central India
	ICC1882	*Cicer arietinum Desi*	India
	ICC8261	*Cicer arietinum Kabuli*	Turkey
	ICC283	*Cicer arietinum Desi*	India
	SBD377	*Cicer arietinum Desi*	Northern India
	Pusa1103	*Cicer arietinum Desi*	Northern India
	Pusa362	*Cicer arietinum Desi*	Northern India
	ICCV10	*Cicer arietinum Desi*	India
	JG11	*Cicer arietinum Desi*	Southern India
	JG130	*Cicer arietinum Desi*	Central India
	ICCC4	*Cicer arietinum Desi*	Western India
**Ib**	Dilaji	*Cicer arietinum Desi*	Eastern India
	BGD112	*Cicer arietinum Desi*	Northern India
	Kranthi	*Cicer arietinum Desi*	Southern India
	Bharathi	*Cicer arietinum Desi*	Southern and Central India
	Pratap Chana1	*Cicer arietinum Desi*	Northern India
	JAKI9218	*Cicer arietinum Desi*	Central India
	CO4	*Cicer arietinum Desi*	Southern India
	Pusa256	*Cicer arietinum Desi*	Northern India
	Vishal	*Cicer arietinum Desi*	Central India
	GG2	*Cicer arietinum Desi*	Western India
**Ic**	Vaibhav	*Cicer arietinum Desi*	Central India
	JG14	*Cicer arietinum Desi*	Central India
	Pusa372	*Cicer arietinum Desi*	Northern India
	WR315	*Cicer arietinum Desi*	Northern India
	BGD1103	*Cicer arietinum Desi*	Northern India
	BG256	*Cicer arietinum Desi*	Northern India
	Pusa547	*Cicer arietinum Desi*	Northern India
	BG5028	*Cicer arietinum Desi*	Northern India
**Id**	Himchana2	*Cicer arietinum Desi*	Western India
	Himchana1	*Cicer arietinum Desi*	Western India
	JG16	*Cicer arietinum Desi*	Central India
**IIa**	JGK3	*Cicer arietinum Kabuli*	Central India
	ICCV2	*Cicer arietinum Kabuli*	Southern India
	JGK2	*Cicer arietinum Kabuli*	Central India
	KAK2	*Cicer arietinum Kabuli*	Central India
	LBeG7	*Cicer arietinum Kabuli*	Southern India
	JGK1	*Cicer arietinum Kabuli*	Central India
	Vihar	*Cicer arietinum Kabuli*	Southern India
	Swetha	*Cicer arietinum Kabuli*	Southern India
**IIb**	Pusa1108	*Cicer arietinum Kabuli*	Northern India
	BGD1105	*Cicer arietinum Kabuli*	Northern India

	BG5023	*Cicer arietinum Kabuli*	Northern India
	BGD2024	*Cicer arietinum Kabuli*	Northern India
	Pusa1088	*Cicer arietinum Kabuli*	Northern India
	PG515	*Cicer arietinum Kabuli*	Central India
